# The prognostic and clinicopathologic characteristics of CD147 and esophagus cancer: A meta-analysis

**DOI:** 10.1371/journal.pone.0180271

**Published:** 2017-07-11

**Authors:** Hui Li, Chunxiang Jiang, Dongwen Wu, Shupeng Shi, Mengting Liao, Jing Wang, Yanwen Li, Zihao Xu

**Affiliations:** 1 Reproductive Department, Xiangya Hospital, Central South University, Changsha, China; 2 Xiangya School of Medicine, Central South University, Changsha, China; 3 Oncology department of Xiangya Hospital, Central South University, Changsha, China; Sapporo Ika Daigaku, JAPAN

## Abstract

**Objective:**

The prognostic significance of CD147 expression in esophageal cancer patients remains controversial. Using a meta-analysis, we investigated the prognostic and clinicopathologic characteristics of CD147 in esophageal cancer.

**Methods:**

A comprehensive literature search of the PubMed (1966–2016), EMBASE (1980–2016), Cochrane Library (1996–2016), Web of Science (1945–2016), China National Knowledge Infrastructure (1982–2016), and Wanfang databases (1988–2016) was performed to identify studies of all esophageal cancer subtypes. Correlations between CD147 expression and survival outcomes and clinicopathological features were analyzed using meta-analysis methods.

**Results:**

Seventeen studies were included. High CD147 expression reduced the 3-year survival rate (OR = 3.26, 95% CI = (1.53, 6.93), p = 0.02) and 5-year survival rate(OR = 4.35, 95% CI = (2.13, 8.90), p < 0.0001). High CD147 expression reduced overall survival in esophageal cancer (HR = 1.60, 95% CI = (1.19, 2.15), p = 0.02). Additionally, higher CD147 expression was detected in esophageal cancer tissues than noncancerous tissues (OR = 9.45, 95% CI = (5.39, 16.59), p < 0.00001), normal tissues (OR = 12.73, 95% CI = (3.49, 46.46), p = 0.0001), para-carcinoma tissues (OR = 12.80, 95% CI = (6.57, 24.92), p < 0.00001), and hyperplastic tissues (OR = 3.27, 95% CI = (1.47, 7.29), p = 0.004). CD147 expression was associated with TNM stage (OR = 3.66, 95% CI = (2.20, 6.09), p < 0.00001), tumor depth (OR = 7.97, 95% CI = (4.13, 15.38), p < 0.00001), and lymph node status (OR = 5.14, 95% CI = (2.03,13.01), p = 0.0005), but not with tumor differentiation, age, or sex.

**Conclusion:**

Our meta-analysis suggests that CD147 is an efficient prognostic factor in esophageal cancer. High CD147 expression in patients with esophageal cancer was associated with worse survival outcomes and common clinicopathological indicators of poor prognosis.

## Introduction

Esophagus cancer (EC) is a malignant disease with the eighth incidence rate and the sixth mortality rate wordwide[1, 2]. The prognosis of EC is unfavorable, largely due to its unapparent symptom at the early stage and tumor infiltration and metastasis which makes it hard to completely remove the tumor by surgery[3]. Therefore, it is with great significance to study the molecular mechanism in the development, invasion and metastasis of EC. Up to now, it is proved that the expression of some molecules, such as NF-kappaB, MIF, CXCR4 and EGFR, are related to the treatment or the prognosis of EC[4–6].

CD147, also named extracellular matrix (ECM) metalloproteinase inducer, is a molecule highly expressed on the surface of cancer cells and promotes the secretion of matrix metalloproteinases (MMPs) from fibroblasts, degrading the matrix of cancer cell and thus facilitating the invasion and metastasis of cancer[7, 8]. Numerous papers showed that CD147 plays an important role in different sorts of cancer, including bladder cancer, prostate cancer, ovarian cancer, glioma, and esophageal cancer is also one of them[9–14].

Although evidence exists that CD147 is an important factor implicated in clinicopathological features and the prognosis of EC. Some conflicting results have been reported. Wan and Wu[15] reported the CD147 expression wasn’t associated with overall survival (OS), which contradictory with Zhu et al[16]. Some studies found that CD147 high Expression might be related to advanced clinical stage and lymph node metastasis[15, 17]. But other studies reported that there was no significant difference between CD147 and clinical stage and lymph node metastasis[17, 18]. Moreover, there are also arguments about relationship of the CD147 high expression with invasive depth, histological differentiation[16, 18–20].

This controversial issue could be results of differences in sample sizes and other factors, such as the criteria of the CD147 high expression, and unfortunately evidence-based confirmation by large-scale clinical trials is still lacking. Therefore, we conducted this meta-analysis to quantitatively inspect the relationship between CD147 and clinicopathological features and survival of EC patients.

## Methods and materials

### Search strategy

We searched PubMed (1966–2016), EMBASE (1980–2016), the Cochrane Library (1996–2016), Web of Science (1945–2016), China National Knowledge Infrastructure (1982–2016), and the WanFang databases (1988–2016). The studies were restricted to humans, but not restricted by date, language, or publication status. The following combined search term was used:(Esophageal Cancer, esophageal carcinoma, esophageal neoplasms, carcinoma of esophagus, esophageal tumor, Malignant Neoplasm of Oesophagus) AND (CD147, (extracellular AND matrix AND metalloproteinase AND inducer), extracellular MMP inducer, EMMPRIN, BSG) to identify relevant papers addressing all subtypes of esophageal cancer. We combined the term appropriately with MeSH Terms and used an appropriate adjustment for different databases. Details of the search strategies can be found in S1 File.

### Criteria for including studies

1. Published or unpublished case control study or cohort study in English or Chinese with the full text available;
2. All cases had survival or clinical pathological characteristic data, without radiotherapy or chemotherapy or biological therapy before sampling;
3. Diagnosis of esophagus cancer was proven by pathological methods;
4. Studies of CD147 expression based on primary esophagus cancer tissues, rather than serum or any other kinds of indirect specimen were included;
5. The best quality study was retained for dealing duplicated studies.

### Criteria for excluding studies

1. Cell or animal studies, case reports, letters, reviews;
2. The standard of pathological diagnosis was not clear.

### Assessment of included studies

The Newcastle-Ottawa quality assessment scale of case control studies (NOS)[21] was adopted to assess the quality of included studies, which has three categories (selection, comparability, and exposure) and eight items. The quality assessment values ranged from 0 to 9 stars. Studies scored more than 6 stars was included for our analysis.

### Statistical analysis

Records were independently scanned by two authors to exclude apparent irrelevant studies. Then, full text were independently reviewed by two authors, and controversial opinions about whether to include specific study were resolved by discussion. Data was extracted independently by two authors: Hui Li and Chunxiang Jiang. Excel was designed according to the Cochrane manual to extract data and the survival data from the Kaplan-Meier curve was obtained by using Engauge Digitizer software. The software Revman 5.3 and Stata 13.0 were applied to analyze the data. Results were showed with odds ratios (OR) or HR (hazard ratio) and 95% confidence intervals (95% CI). Fixed-effects model was adopted when there was no evidence of significant heterogeneity (p > 0.1 and *I*^2^ < 50%); otherwise, random-effects model was used. If possible, heterogeneity was explored and subgroup analyses were performed. All p values were 2-sided, and p < 0.05 was considered significant.

Sensitivity analysis was also performed to evaluate the influence of individual study on the final effect if the parameter has more than two data sets, and different model was used for no more than two data sets.

Begg’s test was used to assess publication bias (p <0.05 was considered statistically significant). If publication bias was confirmed, a trim-and-fill method developed by Duval and Tweedie[22] was implemented to adjust for this bias. Then, we replicated the funnel plot with their ‘‘missing” counterparts around the adjusted summary estimate.

## Results

### Literature search

**Fig 1:**
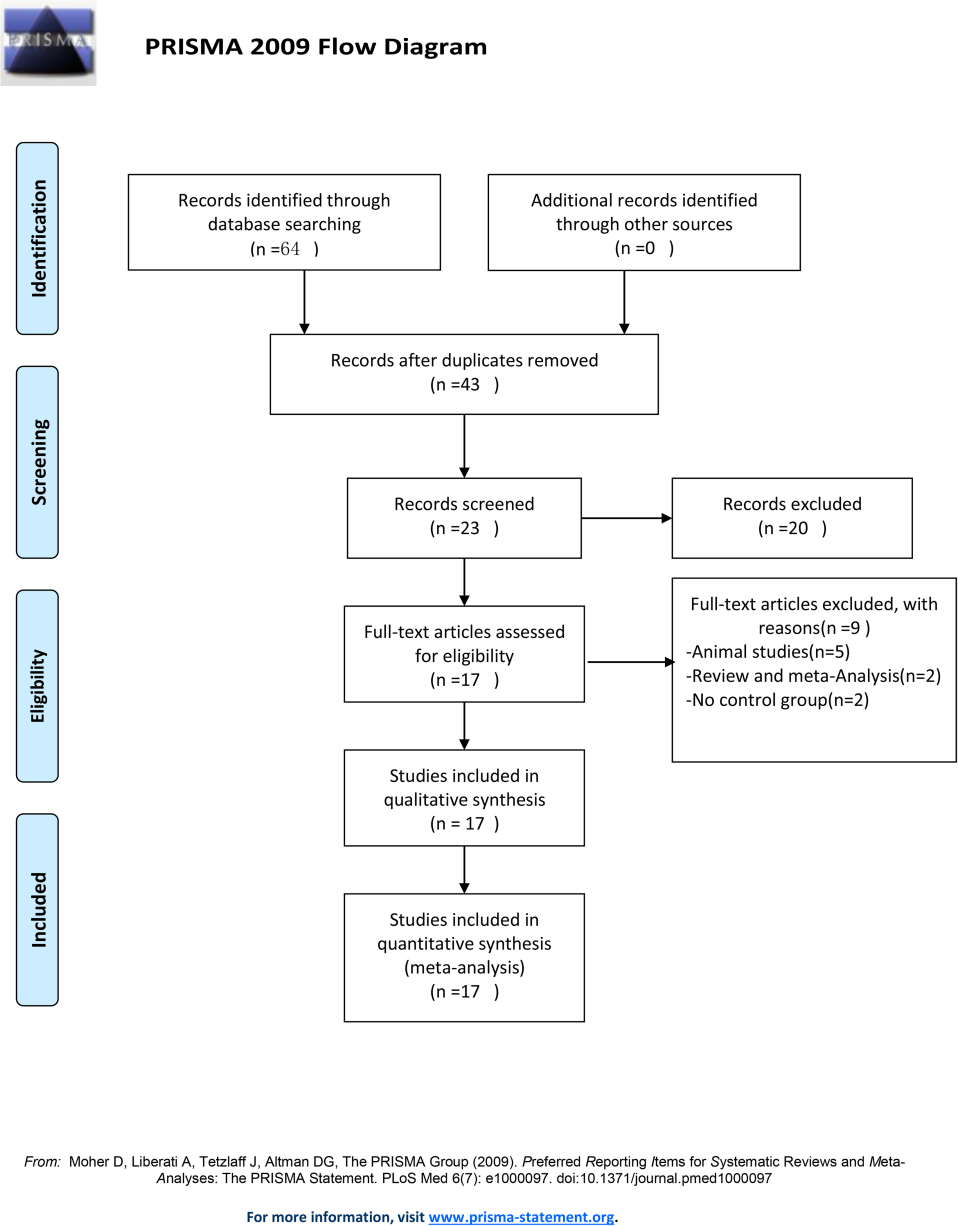
Flowchart of selection of studies for inclusion in meta-analysis. A total of 64 studies were identified, and 21 studies were excluded because of duplication. After reading the titles and abstracts, 20 studies were excluded. 23 possible full text studies were carefully reviewed (animal studies [n = 5]; review and meta-analysis [n = 2]; no control group [n = 2]). Finally, 17 trials were included for quantitative analysis.

The literature searches revealed 64 studies, of which 21 studies were excluded owing to duplication. After reading the titles and abstracts, 20 studies were excluded. The full-length texts of 23 candidate studies were carefully reviewed (animal studies [n = 5]; review and meta-analysis [n = 2]; no control group [n = 2]). Finally, 17 trials were included in the quantitative analysis (Fig 1). Only Huang et al.[7] reported CD147 expression in type II/III adenocarcinoma of the esophagogastric junction (Type II/III AEGs). An association between CD147 and esophageal squamous cell carcinoma (ESCC) was reported in 16 studies.

### Qualitative assessment

Study quality was assessed using the Newcastle–Ottawa quality assessment scale; scores ranged from 7 to 8 (with a mean of 7.35), and higher values indicated better methodology. The quality assessment results are shown in S1 Table and detailed information for this analysis is provided in Table 1.

**Table 1:** Characteristics of eligible studies.

First Author	Year	Origin	Median age	sample size	CD147 distribution	Type of cancer	Counting method	Definition of CD147 positive	NOS score
Yoshio Ishibashi	2004	Japan	61	101	-	ESCC	-	No staining, partial staining, and diffuse and strong staining	8
Zhao JH	2004	China	54.6	70	M and C	ESCC	A and B	>0% or weak intensity	7
Cheng, M. F	2006	Taiwan	62.5	41	M and C	ESCC	A and B	>0% or weak intensity	7
Zhang HZ	2006	China	58	85	M	ESCC	B	Brown	7
Xiong SongBai	2007	China	55.6	57	M and C	ESCC	A and B	>0% or weak intensity	7
Xie L	2008	China	53.2	87	M and C	ESCC	A and B	>5% or weak intensity	7
Qi Bo	2008	China	58.2	52	C	ESCC	A and B	>0% or weak intensity	8
Ma Guang	2009	China	59.5	70	M and C	ESCC	A and B	A+B>3	8
Chen JX	2009	China	42	50	M and C	ESCC	A and B	>10% or weak intensity	7
Liu HaiMing	2010	China	-	19	M and C	ESCC	A	>10%	7
Xiao XiangZhi	2011	China	50	60	M and C	ESCC	A and B	A2*B>1	7
Zhu ShaoJun	2011	China	-	108	M and C	ESCC	A	>5%	7
Xiong LN	2011	China	-	40	M and C	ESCC	A	>5%	8
Zhu, S	2011	China	-	86	M and C	ESCC	A	>5%	7
Wan, Y	2012	China	58.8	80	M	ESCC	A and B	A3*B≥5	8
Li ChangXiu	2013	China	63.4	60	M and C	ESCC	A and B	A4*B≥5	7
Huang, L	2015	China	62.2	74	M and C	Type II/III AEGs	A and B	A3*B≥3	8

Positive cell percentage (A). A1:0 point for positive cell percentage≤ 25%;1 point for 26%-50%; 2 points for 51%-75%;3 points for>75%. A2: point for positive cell percentage<5%;1 point for 5%-25%,26%-50%;3 points for >50%. A3:0 point for positive cell percentage≤ 5%, 1 point for 6%-25%, 2 points for 26%-50%, 3 points for 51%-75%, and 4 points for >75%. A4: point for positive cell percentage< 1%;1 point for1%-10%; 2 points for11%~50%;3 points for≥51%. Staining intensity (B):0 point for basically no coloration, 1 point for light yellow, 2 points for pale brown, and 3 points for dark brown. M:Membrane,C:Cytoplasm. ESCC:esophageal squamous cell carcinoma. Type II/III AEGs:type II/III adenocarcinoma of esophagogastric junction.

### CD147 and survival analysis

#### CD147 expression and overall survival

**Fig 2:**
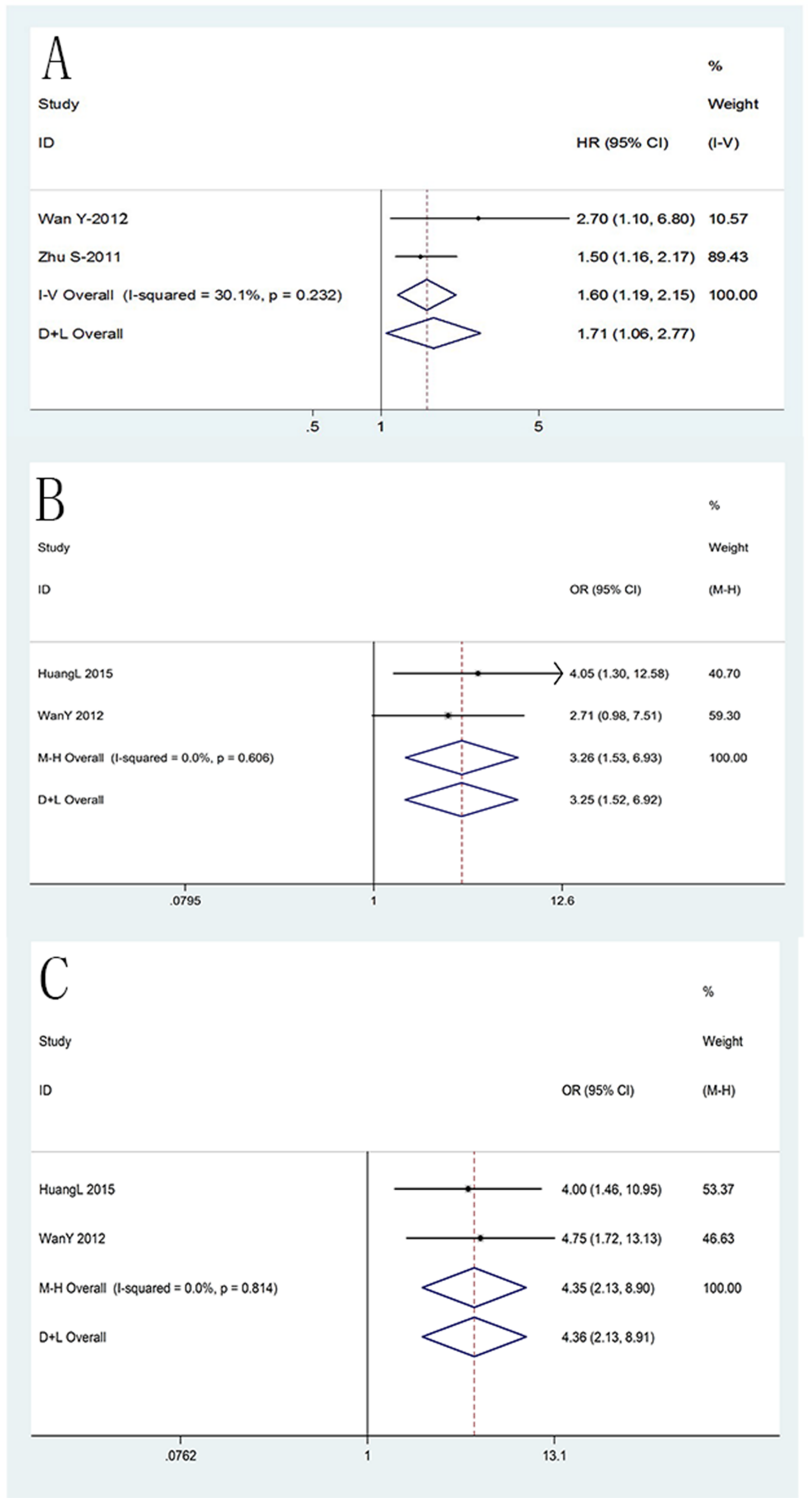
Survival analysis forest plot. The squares and horizontal lines correspond to the study- specific OR and 95%CI.The area of the squares reflects the study-specific weight (inverse of the variance). The diamonds represent the pooled OR and 95% CI. The solid vertical line is at the null value (OR = 1). **A** The relationship between CD147 expression and overall survival. CD147 expression was associated with overall survival (HR = 1.60, 95% CI = (1.19, 2.15), p = 0.02). **B** The relationship between CD147 expression and 3-year survival rate. CD147 expression was associated with 3-year survival rate (OR = 3.26, 95%CI = (1.53,6.93),p = 0.02). **C** The relationship between CD147 expression and 5-year survival rate.CD147 expression was associated with 5-year survival rate (OR = 4.35, 95%CI = (2.13, 8.90), p < 0.0001).

We analyzed the relationship between CD147 expression and overall survival in EC patients based on the results of two studies[9, 15]. Wan and Wu[15] used Kaplan–Meier survival curves and Zhu et al.[9] used Cox regression analyses. In both studies, high CD147 expression was related to poor OS. As shown in Fig 2A, the data did notexhibit heterogeneity (p = 0.23, *I*^2^ = 30.1%), and the fixed effects model showed thatthe combined HR was 1.60 (95% CI = (1.19, 2.15), p = 0.02).

#### Impact of CD147 on 3-year survival rate of EC

Two reports[7, 15] including a total of 154 patients reported an association between CD147 expression and the 3-year survival rate. Huang et al.[7] detected CD147 expression in Type II/III AEGs, and Wan and Wu[15] observed CD147 expression in ESCC. Without heterogeneity (p = 0.61, *I*^2^ = 0%), a fixed-effects model showed that high CD147 expression (57.95%) was statistically significantly associated with a lower 3-year survival rate than that of low expression (81.82%) (OR = 3.26, 95% CI = (1.53, 6.93), p = 0.02) (Fig 2B).

#### Impact of CD147 on the 5-year survival rate of EC

The association between CD147 and the 5-year survival rate of EC was reported in two studies[7, 15]. Both Huang et al.[7] (OR = 4.0, 95% CI = (1.46, 10.95)) and Wan and Wu[15] (OR = 4.75, 95% CI = (1.72, 13.13)) showed that high CD147 expression is statistically significantly associated with a lower 5-year survival rate. Without heterogeneity (p = 0.81, *I*^2^ = 0%), a significant difference in the 5-year survival rate was detected between groups with high CD147 expression (29.55%) and low CD147 expression (62.12%) assuming a fixed-effects model (OR = 4.35, 95% CI = (2.13, 8.90), p < 0.0001) (Fig 2C).

#### CD147 expression and disease-free survival (DFS)

Ishibashi et al.[17] reported an association between CD147 and DFS. The risk was 1.5 times higher for the high CD147 expression group than the low CD147 expression group (HR = 4.6, 95% CI = 1.55, 13.4), p = 0.006). The pooled HR for DFS showed that high expression of CD147 reduced DFS in EC.

### CD147 expression in different EC tissues

#### CD147 in esophageal cancer and noncancerous tissues

**Fig 3:**
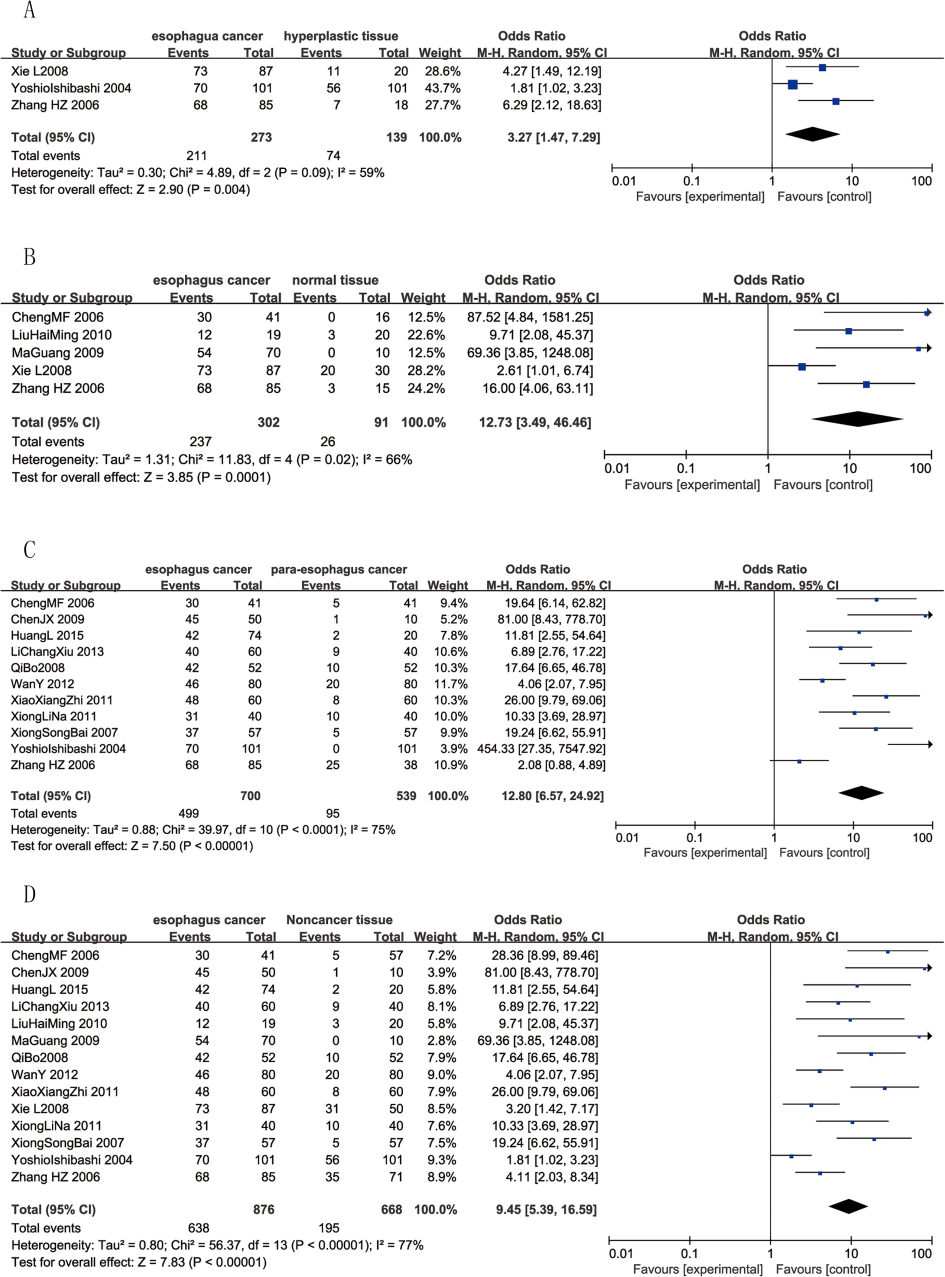
Forest plots of CD147 expression and different tissues. The squares and horizontal lines correspond to the study-specific OR and 95% CI.The area of the squares reflects the study-specific weight (inverse of the variance). The diamonds represent the pooled OR and 95% CI. The solid vertical line is at the null value (OR = 1). **A** CD147 positive expression between cancer and noncancer tissues.Significant difference was found between cancer and noncancer tissues (OR = 9.45, 95%CI = (5.39, 16.59), p < 0.00001). **B** CD147 positive expression between cancer and normal tissues.Significant difference was found between cancer and normal tissues (OR = 12.73, 95%CI = (3.49, 46.46), p = 0.0001). **C** CD147positive expression between cancer and para-carcinoma tissues.Significant difference was found between cancer and para-carcinoma tissues (OR = 12.80, 95%CI = (6.57, 24.92), p < 0.00001). **D** CD147 positive expression between cancer and hyperplastic tissues.Significant difference was found between cancer and hyperplastic tissues (OR = 3.27, 95% CI = (1.47, 7.29), p = 0.004).

CD147 expression in esophageal cancer and noncancerous tissues was investigated in 14 studies[7, 15, 17–20, 23–30] including 1544 patients. With significant heterogeneity (p < 0.00001, *I*^2^ = 77%), a random-effects model showed that CD147 expression in esophageal cancer (72.83%) was higher than that in noncancerous tissues (29.19%) (OR = 9.45, 95% CI = (5.39, 16.59), p < 0.00001) (Fig 3A).

#### CD147 in esophageal cancer and normal esophageal tissues

Five trials[18, 20, 24, 29, 30] reported the expression of CD147 in esophageal cancer tissues and normal esophageal cancer tissues, including 302 esophageal cancer tissues and 91 normal esophageal cancer tissues. With significant heterogeneity (p = 0.02, *I*^2^ = 66%), a random-effects model showed that CD147 expression was higher inesophageal cancer tissues (78.48%) than in normal tissues (28.57%) (OR = 12.73, 95%CI = (3.49, 46.46), p = 0.0001) (Fig 3B).

#### CD147 in esophageal cancer and para-carcinoma tissues

Eleven trials[7, 15, 17–19, 23, 25–28, 30] investigated the expression of CD147 in esophageal cancer tissues and para-carcinoma tissues, including 700 esophageal cancer tissues and 539 para-carcinoma tissues. A random-effects model showed that CD147 expression was higher in esophageal cancer tissues (71.29%) than in para-carcinoma tissues (17.63%) (OR = 12.80, 95% CI = (6.57, 24.92), p < 0.00001) with significant heterogeneity (p < 0.0001, *I*^2^ = 75%) (Fig 3C).

#### CD147 in esophageal cancer and hyperplastic tissues

Three trials[17, 18, 29] reported the expression of CD147 in esophageal cancer tissues and hyperplastic tissues, including 273 esophageal cancer tissues and adjacent hyperplastic tissues. A random-effects model showed a difference in the rate of high CD147 expression between the two groups (OR = 3.27, 95% CI = (1.47, 7.29), p = 0.004) with heterogeneity (*I*^2^ = 59%, p = 0.09) (Fig 3D).

### Correlation of CD147 with clinicopathological parameters

#### Correlation between CD147 and TNM stage of esophageal cancer tissues

**Fig 4:**
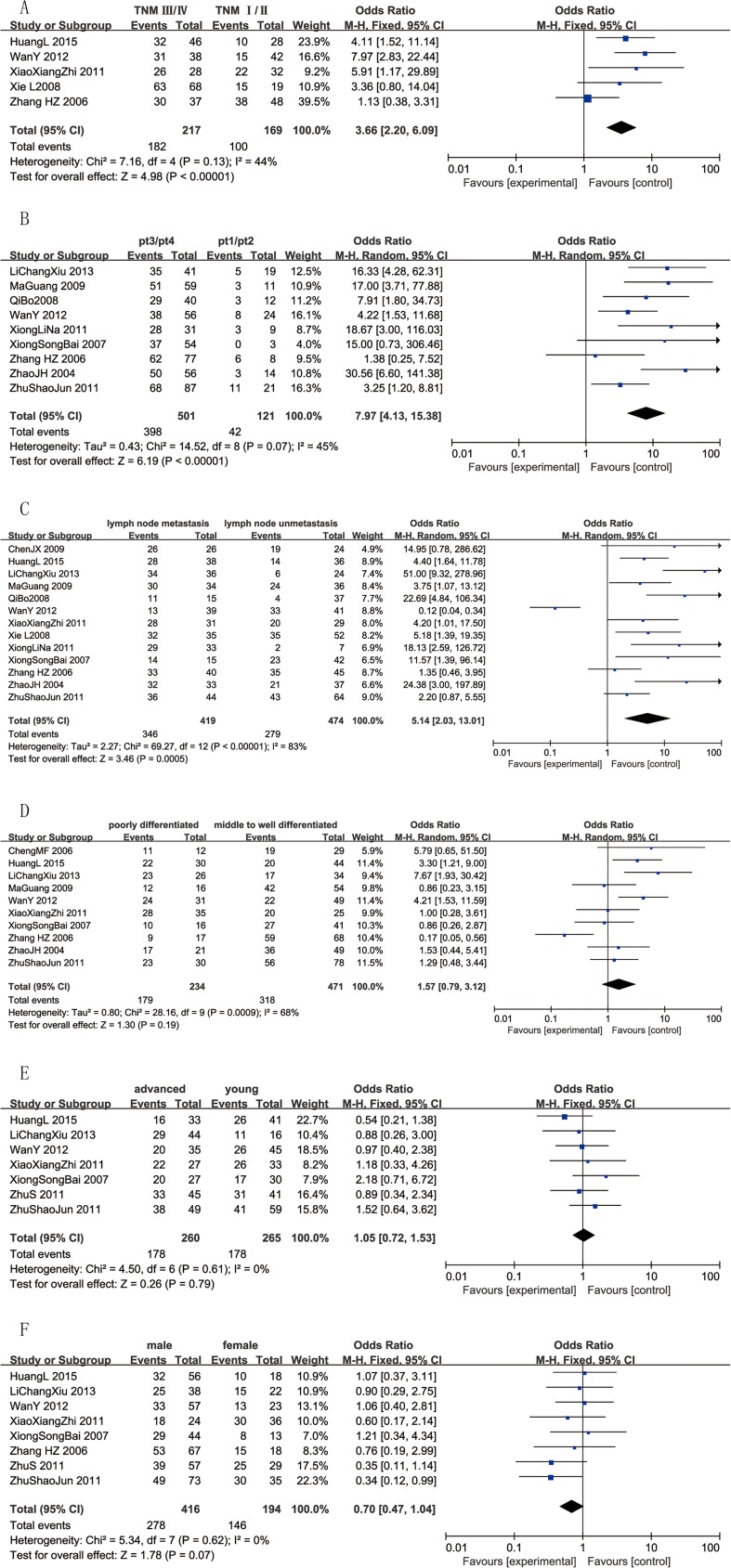
Forest plots of CD147 expression and the clinicopathological features of patients with esophagus cancer. The squares and horizontal lines correspond to the study- specific OR and 95% CI.The area of the squares reflects the study-specific weight (inverse of the variance). The diamonds represent the pooled OR and 95% CI. The solid vertical line is at the null value (OR = 1). **A** The relationship between CD147 expression and TNM staging. CD147 expression was associated with TNM staging of esophagus cancer(OR = 3.66, 95%CI = (2.20, 6.09), p < 0.00001). **B** The relationship between CD147 expression and tumor depth. CD147 expression was associated with tumor depth (OR = 7.97, 95%CI = (4.13, 15.38), p < 0.00001). **C** The relationship between CD147 expression and status of lymph node.CD147 expression was associated with status of lymph node (OR = 5.14, 95%CI = (2.03, 13.01), p = 0.0005). **D** The relationship between CD147 expression and tumor differentiation.CD147 expression wasn’t associated with tumor differentiation (OR = 1.57, 95%CI = (0.79, 3.12), p = 0.19). **E** The relationship between CD147 expression and age.CD147 expression wasn’t associated with age (OR = 1.05, 95%CI = (0.72,1.53), p = 0.79). **F** The relationship between CD147 expression and sex.CD147 expression wasn’t associated with sex (OR = 0.70, 95%CI = (0.47, 1.04), p = 0.07).

TNM stage is an international standard for tumor staging. TNM stage I–II has a better prognosis than TNM stage III–IV in EC. The association between CD147 and TNM stage was investigated in five studies[7, 15, 18, 23, 29]. A fixed-effects model wasused without heterogeneity (p = 0.13, *I*^2^ = 44%); it indicated a significant difference between the TNM stage III–IV group (83.87%) and TNM stage I–II group (59.17%) (OR = 3.66, 95% CI = (2.20, 6.09), p < 0.00001) (Fig 4A).

#### CD147 with invasive depth of esophageal cancer tissues

Nine studies[15, 16, 18–20, 26–28, 31] including 622 tissue samples investigated the relationship between CD147 expression and depth of tumor invasion. With significant heterogeneity (p = 0.07, *I*^2^ = 45%), a random-effects model showed a significant difference between the pt3/pt4 group (79.44%) and pt1/pt2 group (34.71%) (OR = 7.97, 95% CI = (4.13, 15.38), p < 0.00001) (Fig 4B).

#### CD147 with lymph node metastasis of esophageal cancer tissues

Prognosis is often not good when esophageal cancer patients develop lymph node metastasis; accordingly, it is very important to identify indicators of metastasis at an early stage. Thirteen studies[7, 15, 16, 18–20, 23, 25–29, 31] that examined metastasis were included. With significant heterogeneity (p < 0.00001, *I*^2^ = 83%), a random-effects model showed a significant difference between the lymph node metastasis group (82.58%) and the non-metastasis group (58.86%) (OR = 5.14, 95% CI = (2.03, 13.01), p = 0.0005) (Fig 4C).

#### CD147 with differentiation of esophageal cancer tissues

The association between CD147 and histological differentiation was investigated in ten studies[7, 15, 16, 18, 20, 23, 26, 28, 30, 31]. With significant heterogeneity(p = 0.0009, *I*^2^ = 68%), a random-effects model showed no difference between 234 poorly differentiated tissues (76.50%) and 471 moderately to well differentiated tissues (67.52%) (OR = 1.57, 95% CI = (0.79, 3.12), p = 0.19) (Fig 4D).

#### CD147 with age and sex of esophageal cancer tissues

Seven[7, 9, 15, 16, 23, 26, 28] and eight studies[7, 9, 15, 16, 18, 23, 26, 28] reported the relationship of CD147 expression with age and sex, respectively. Heterogeneity was not observed in the analysis of CD147 expression with respect to age (p = 0.61, *I*^2^ = 0%) and sex (p = 0.62, *I*^2^ = 0%); therefore, a fixed-effect model was used. The results showed that CD147 was not associated with age (OR = 1.05, 95%CI = (0.72, 1.53), p = 0.79) (Fig 4E) or sex (OR = 0.70, 95% CI = (0.47, 1.04), p = 0.07) (Fig 4F).

### Sensitivity analysis and publication bias

A sensitivity analysis was performed to evaluate the stability of the results. As the survival analysis included fewer than three data sets, a different model was used or the sensitivity analysis, i.e., a random effect model. The results indicated stability, as shown in Table 2.

**Table 2:** Summary of the sensitivity analysis of parameters with less than 3 data sets.

	Sample number	Fixed model	Random model	Heterogeneity	Publication bias(p value)
3-year survival rate	154	OR = 3.26, 95% CI = (1.53, 6.93)	OR = 3.25,95% CI = (1.52, 6.92)	*I*^2^ = 0.0%, p = 0.606	1.000
5-year survival rate	154	OR = 4.35, 95% CI = (2.13, 8.90)	OR = 4.35, 95% CI = (2.13, 8.90)	*I*^2^ = 0.0%, p = 0.814	1.000
Overall survival	166	HR = 1.60, 95% CI = (1.19, 2.15)	HR = 1.71, 95% CI = (1.06, 2.77)	*I*^2^ = 30.1%, p = 0.233	1.000

We excluded studies one-by-one for the sensitivity test for parameters with more than 3 data sets. The sensitivity analysis (S1 Fig and S2 File) showed that all parameters are stable, except for EC vs. hyperplastic tissues and poorly vs. middle to well differentiated states (Table 3). For publication bias, we used Begg’s test (S2 Fig and S3 File). Only two parameters (EC vs. noncancerous and lymph node metastasis group) showed publication bias (Table 3). We then used a trim-and-fill method, as described in the Materials and Methods, after omitting studies with small sample sizes. The pooled analysis results were the same as the original results based on all studies.

**Table 3:** Summary of sensitivity analysis of parameters with more than 2 data sets.

	OR Fluctuation	95%CI Fluctuation	Publication bias (p value)
**CD 147 Expression among different tissue**			
EC VS noncancer	8.55~10.73	4.87~19.43	0.024
EC vs normal tissue	9.27~18.43	2.37~90.29	1.000
EC vs para-EC	10.82~15.24	5.83~30.30	0.074
EC vs hyperplastic tissue	2.48~5.14	0.93~10.95	0.296
**CD 147 Expression with clinicopathologic characteristics**			
TNM I/II vs TNM III/IV	2.80~5.31	1.94~9.59	1.000
pt3/pt4 VS pt1/pt2	6.64~9.46	3.54~19.26	0.536
LNM vs LNUM	4.22~6.50	1.74~16.32	0.016
Poorly vs middle to well differentiated	1.33~2.01	0.67~3.55	0.721
Advance VS Young	0.95~1.08	0.63~1.82	1.000
Male vs Female	0.64~0.80	0.41~1.18	0.902

Note: p < 0.05, exist Publication Bias;EC means esophagus cancer; LNM means lymph node metastasis; LUNM means lymph node unmetastasis.

## Discussion

CD147 is a 55-kDa molecule found on the surface of tumor cells. It can stimulate the expression of MMPs, which facilitate the invasiveness of cancer cells[17, 32]. The correlation between CD147 expression and EC has been investigated extensively. However, the clinical relevance of CD147 remains controversial. Sample size, as a strong predictor in epidemiological studies, may play an important role in resolving this controversy. In the current meta-analysis, we pooled data from 17 studies and demonstrated a remarkable association between CD147 expression and EC. We evaluated the association between CD147 in cancer and other tissues. Based on our results, we concluded that high CD147 expression was significantly associated with malignant tissues.

CD147 stimulates adjacent interstitial normal cells to produce MMPs[33]. MMPs are proteases known to degrade the ECM[34]. Thus, carcinoma cells can interact with adjacent normal cells to produce MMPs via CD147 on their surface, and, in turn, invade lymphatic tissue and blood vessels and penetrate the ECM to reach adjacent organs, with the help of MMPs. Tumor invasion and metastasis are a major barrier to cancer treatment and a main cause of death[35]. The basement membrane and ECM form a histological barrier that can prevent the progression of malignant tumors, and its degradation facilitates tumor progression.

Efforts were made to conduct a comprehensive analysis, but some limitations need to be acknowledged. First, despite our efforts, we did not obtain unpublished data; therefore, the data included in the analyses were from only published data. However, most of the parameters showed no publication bias according to Begg’s test, with the exception of two indicators (EC vs. Noncancerous and lymph node metastasis). We obtained a stable result when studies with small sample sizes were removed. Second, only 4 studies[7, 9, 15, 17] including 341 patients reported survival data. Wan and Wu[15] and Zhu et al. [9]focused on overall survival and Ishibashi et al.[17] considered disease-free survival. Wan and Wu[15] and Huang et al.[7] reported impact of CD147 on the 3-year survival rate and 5-year survival rate. Accordingly, the small sample size is a limitation. Fortunately, we obtained stable results among models in a test of the sensitivity. Last,16 studies reported an association between high CD147 expression and ESCC; therefore, our results were particularly representative of ESCC. Further studies of adenocarcinoma of the esophagus are required to verify these results. However, Huang et al.[7] also showed that high CD147 expression in Type II/III AEGs was significantly associated with cancer tissue types (esophageal cancer versus noncancerous tissues (OR = 11.81, 95%CI = (2.55, 54.64)), poor 3-year survival (OR = 4.05, 95% CI = (1.30, 12.58)), poor 5-year survival (OR = 4.00, 95% CI = (1.46, 10.95)), TNM stage (OR = 4.11, 95% CI = (1.52, 11.14)), lymph node metastasis (OR = 4.40, 95% CI = (1.64, 11.78)), and histological differentiation (OR = 3.30, 95% CI = (1.21, 9.00)). Furthermore, the sensitivity analysis showed that the study of Huang et al.[7] had no influence on the results.

To our knowledge, this meta-analysis is the first study to systematically estimate the association between CD147 expression and the risk of EC and its clinicopathological parameters. Early diagnosis and early treatment are fundamental approaches to improve prognosis[36]. Our results indicated that high CD147 was significantly associated with EC tissues, supporting the notion that CD147 could potentially be applied as a clinical marker for the early diagnosis of EC. We demonstrated that high CD147 expression strongly predicted a poorer TNM stage, invasion depth, lymph node metastasis, and a worse survival rate in patients with EC. In conclusion, CD147 was an important molecule for the diagnosis and estimating the prognosis of patients with EC. Further studies using additional putative EC surface markers in combination with CD147 are required to evaluate their potential use in predicting patient outcomes.

## Supporting information

S1 Table NOS score. (DOCX)

S1 File Searching strategy. (DOCX)

S2 File Sensitivity analysis plot legend. (DOCX)

S3 File Begg's plot legend. (DOCX)

S4 File PRISMA 2009 checklist. (DOC)

S1 Fig Sensitivity analysis plot. (TIF)

S2 Fig Begg's plot. (TIF)
