# Expression of Concern: The prognostic and clinicopathologic characteristics of CD147 and esophagus cancer: A meta-analysis

**DOI:** 10.1371/journal.pone.0282229

**Published:** 2023-02-22

**Authors:** The PLOS ONE Editors

After this article was published, similarities were noted between this article [1] and submissions by other research groups, which call into question the validity and provenance of the reported results.

In response to queries about these concerns, the corresponding author provided the underlying data in S1 File. They also noted that there is an error in Fig 3 where Fig 3A and 3C should be swapped in order. During editorial follow-up, it was also noted that there are nine instances across the Abstract, Results, Fig 3A, Fig 3 caption, and Table 1 where ‘hyperplastic’ should be replaced by ‘dysplastic (atypical hyperplasia)’. An updated Fig 3 with Fig 3A and 3C swapped, ‘hyperplastic’ updated to ‘dysplastic (atypical hyperplasia)’, and the associated updated caption are provided below.

The authors commented on aspects of how data were collected and analyzed for this study, but overall their responses did not fully resolve the concerns.

The *PLOS ONE* Editors issue this Expression of Concern to notify readers of the unresolved concerns discussed above, and to provide the data and updated figure received from the authors.

**Fig 3:**
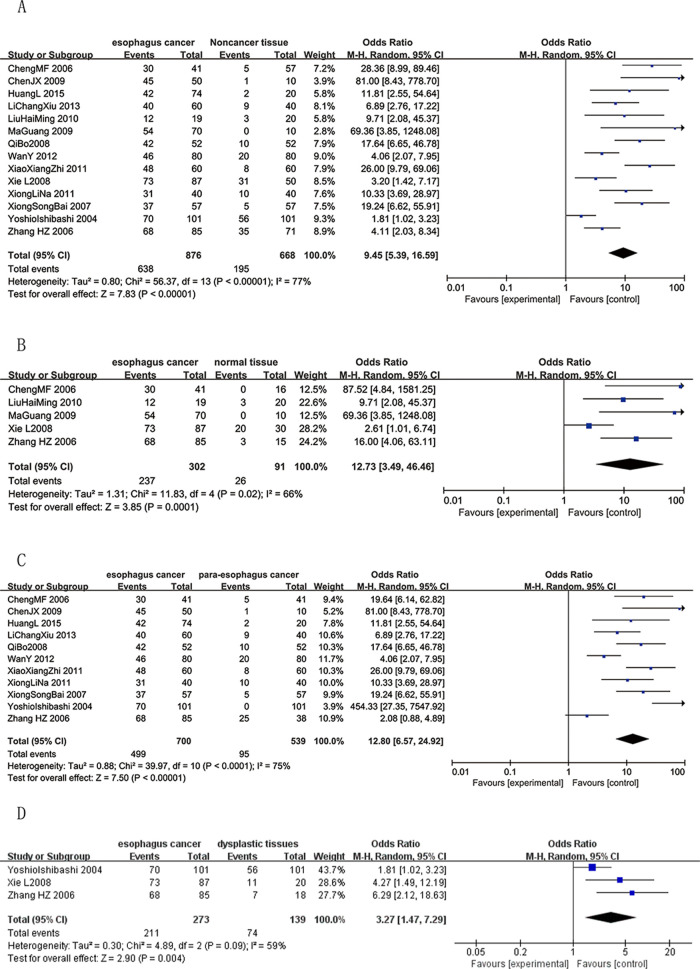
Forest plots of CD147 expression and different tissues. The squares and horizontal lines correspond to the study-specific OR and 95% CI. The area of the squares reflects the study-specific weight (inverse of the variance). The diamonds represent the pooled OR and 95% CI. The solid vertical line is at the null value (OR = 1). **A** CD147 positive expression between cancer and noncancer tissues. Significant difference was found between cancer and noncancer tissues (OR = 9.45,95%CI = (5.39,16.59), P<0.00001). **B** CD147 positive expression between cancer and normal tissues. Significant difference was found between cancer and normal tissues (OR = 12.73, 95%CI = (3.49,46.46), P = 0.0001). **C** CD147 positive expression between cancer and para-carcinoma tissues. Significant difference was found between cancer and para-carcinoma tissues (OR = 12.80, 95%CI = (6.57,24.92), P<0.00001). **D** CD147 positive expression between cancer and dysplastic tissues. Significant difference was found between cancer and dysplastic tissues (OR = 3.27, 95% CI = (1.47,7.29), P = 0.004).

## Supporting information

S1 File The underlying data used for the analysis in this article [1]. (ZIP)
