# Supplementary material for: The prognostic and clinicopathologic characteristics of CD147 and esophagus cancer: A meta-analysis
Source: PLoS One. 2017 Jul 11;12(7):e0180271. doi: 10.1371/journal.pone.0180271 (PMC5507401; doi:10.1371/journal.pone.0180271)
Supplement: S1 File — (DOCX) [file pone.0180271.s002.docx]

1.Pubmed

Eight studies

CD147

antigens, cd147[MeSH Terms] OR cd147[Title/Abstract] OR (extracellular[Title/Abstract] AND matrix[Title/Abstract] AND metalloproteinase[Title/Abstract] AND inducer[Title/Abstract]) OR "extracellular matrix metalloproteinase inducer"[Title/Abstract] OR EMMPRIN[Title/Abstract]

Tumor

Esophageal Neoplasms [MeSH Terms] OR (Esophageal[Title/Abstract] AND Neoplasms[Title/Abstract]) OR (Esophageal[Title/Abstract] AND tumor[Title/Abstract]) OR Esophageal[Title/Abstract]

2.Embase

Ten studies

(('CD147 antigen'/exp OR cd147:ti,ab OR (extracellular:ti,ab AND matrix:ti,ab AND metalloproteinase:ti,ab AND inducer:ti,ab) OR "extracellular matrix metalloproteinase inducer":ti,ab OR EMMPRIN:ti,ab) AND (' Esophageal Neoplasms'/exp OR Esophageal tumor:ti,ab OR Esophageal Neoplasms:ti,ab OR Esophageal:ti,ab)) AND [embase]/lim

3.Cochrane Library

Zero study

(Esophageal Neoplasms:ti,ab or Esophageal tumor:ti,ab or Esophageal:ti,ab) and ([mh "Antigens, CD147"] or CD147:ti,ab or (extracellular:ti,ab and matrix:ti,ab and metalloproteinase:ti,ab and inducer:ti,ab) or "extracellular matrix metalloproteinase inducer":ti,ab or EMMPRIN:ti,ab)

4.WOS

Six studies

(TS=Antigens, CD147 OR TS=cd147 OR (TS=extracellular AND TS=matrix AND TS=metalloproteinase AND TS=inducer) OR TS="extracellular matrix metalloproteinase inducer" OR TS=EMMPRIN) AND (TS= Esophageal Neoplasms OR TS= Esophageal tumor OR TS= Esophageal)

5.CNKI

Eighteen studies

CD147

SU='CD147' OR SU='antigen, CD147' OR SU='extracellular matrix metalloproteinase inducer'

tumor

SU='Esophageal tumor'

6.WANFANG

Twenty-two studies

(subject:(CD147) + subject:(antigen, CD147) + subject:(extracellular matrix metalloproteinase inducer)) * subject:(esophageal carcinoma)

Note:when searching the Chinese database,we used Chinese key words and in this article,we translated it.
