# Supplementary material for: The prognostic and clinicopathologic characteristics of CD147 and esophagus cancer: A meta-analysis
Source: PLoS One. 2017 Jul 11;12(7):e0180271. doi: 10.1371/journal.pone.0180271 (PMC5507401; doi:10.1371/journal.pone.0180271)
Supplement: S3 File — (DOCX) [file pone.0180271.s004.docx]

**Supplementary Begg’s Plot:**

Begg’s test was used to assess publication bias (p < 0.05 was considered statistically significant). If publication bias was confirmed, a trim-and-fill method developed by Duval and Tweedie was implemented to adjust for this bias.

A The publication bias between CD147 positive expression and 3-year survival rate. No publication bias was found with p = 1.000.

B The publication bias between CD147 positive expression and 5-year survival rate. No publication bias was found with p = 1.000.

C The publication bias between cancer and noncancer tissues. Publication bias was found with p = 0.024.

D The publication bias between cancer and normal tissues. No publication bias was found with p = 1.000.

E The publication bias between cancer and para-carcinoma tissues. No publication bias was found with p = 0.074.

F The publication bias between cancer and hyperplastic tissues. No publication bias was found with p = 0.296.

G The publication bias between CD147 expression and TNM staging. No publication bias was found with p = 1.000.

H The publication bias between CD147 expression and tumor depth. No publication bias was found with p = 0.536.

I The publication bias between CD147 expression and status of lymph node. Publication bias was found with p = 0.016.

J The publication bias between CD147 expression and tumor differentiation. No publication bias was found with p = 0.721.

K The publication bias between CD147 expression and age. No publication bias was found with p = 1.000.

L The publication bias between CD147 expression and sex.No publication bias was found with p = 0.902.

.
