# Supplementary figures and images for: The prognostic and clinicopathologic characteristics of CD147 and esophagus cancer: A meta-analysis
Source: PLoS One. 2017 Jul 11;12(7):e0180271. doi: 10.1371/journal.pone.0180271 (PMC5507401; doi:10.1371/journal.pone.0180271)

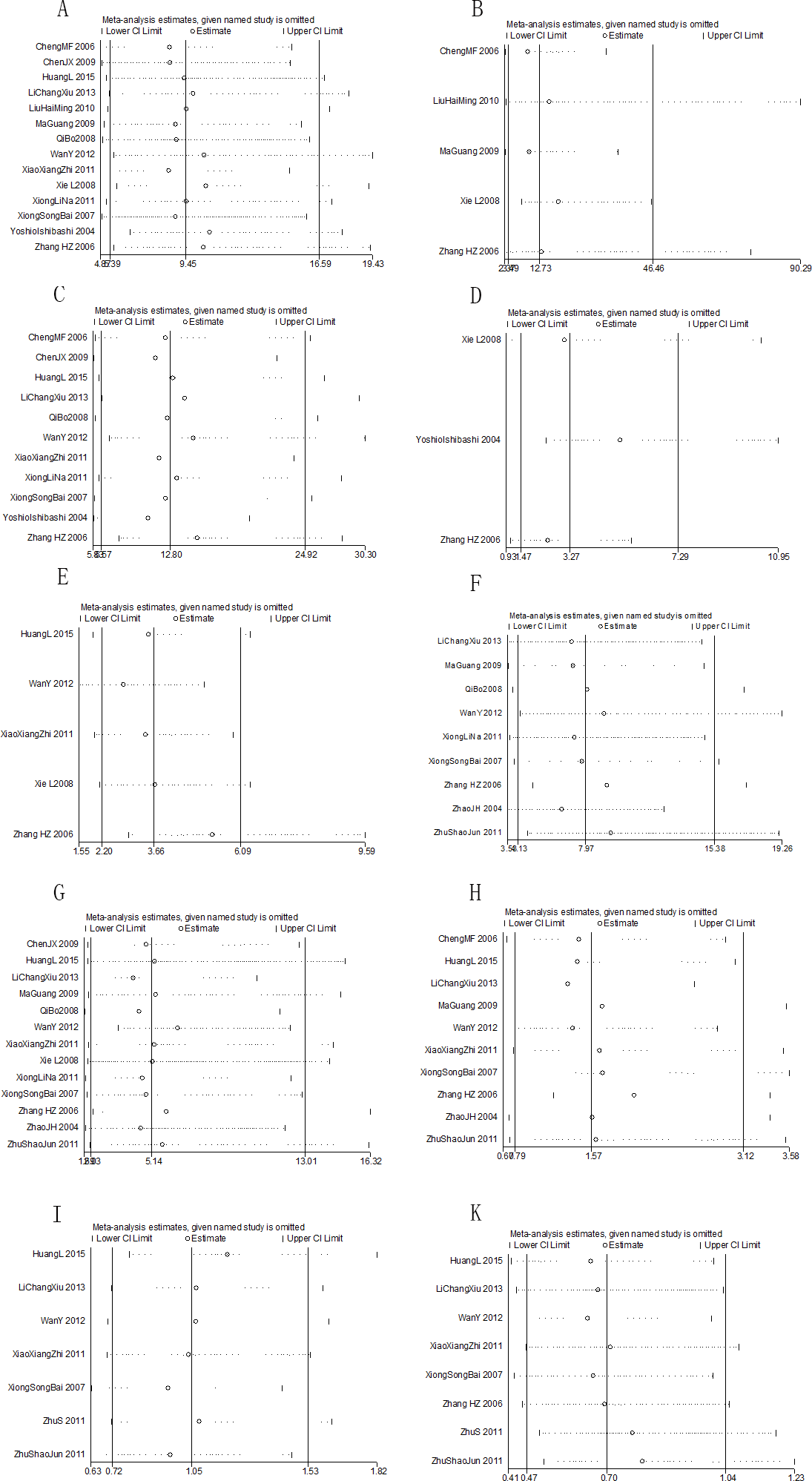

Supplement: S1 Fig — (TIF) [file pone.0180271.s006.tif]

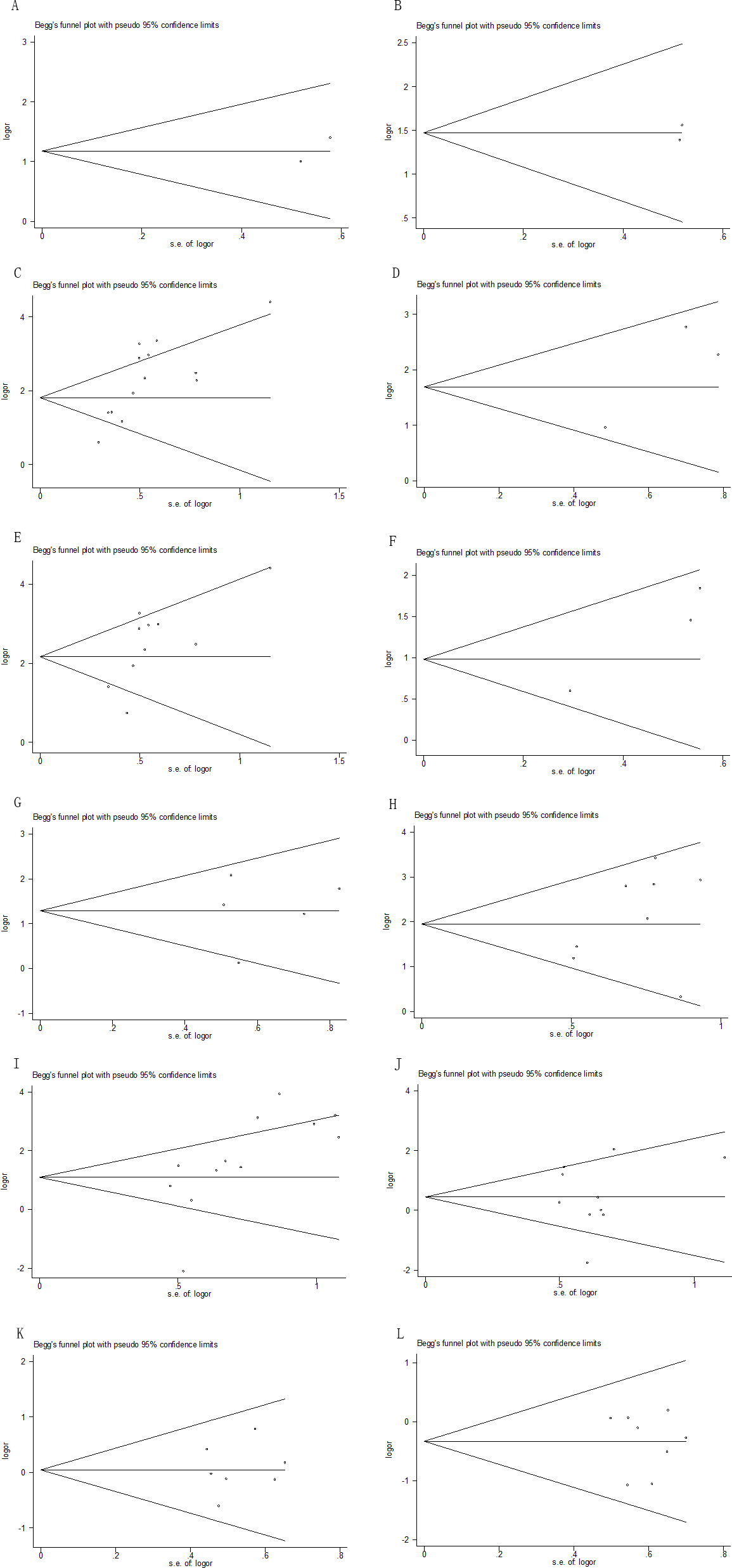

Supplement: S2 Fig — (TIF) [file pone.0180271.s007.tif]
