# Supplementary material for: Expression of Concern: The prognostic and clinicopathologic characteristics of CD147 and esophagus cancer: A meta-analysis
Source: PLoS One. 2023 Feb 22;18(2):e0282229. doi: 10.1371/journal.pone.0282229 (PMC9946197; doi:10.1371/journal.pone.0282229)
Supplement: S1 File — (ZIP) [file pone.0282229.s001.zip › CD147 and Clinical charicteristics plot.docx]

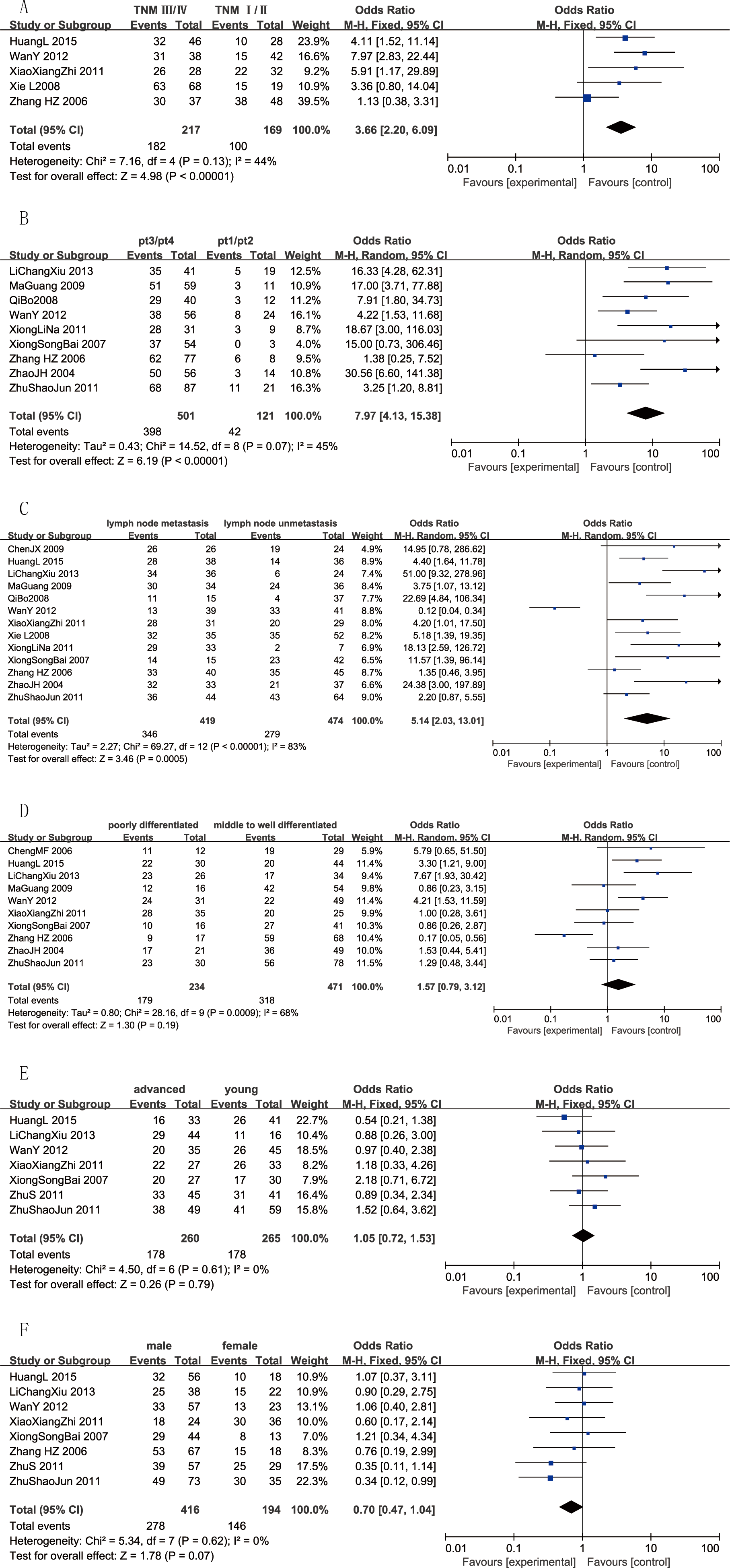


Fig.4. Forest plots of CD147 expression and the clinicopathological features of patients with esophagus cancer. The squares and horizontal lines correspond to the study- specific OR and 95% CI.The area of the squares reflects the study-specific weight (inverse of the variance). The diamonds represent the pooled OR and 95% CI. The solid vertical line is at the null value (OR=1).

A The relationship between CD147 expression and TNM staging. CD147 expression was associated with TNM staging of esophagus cancer(OR=3.66, 95%CI= (2.20,6.09), P<0.00001).

B The relationship between CD147 expression and tumor depth. CD147 expression was associated with tumor depth (OR=7.97, 95%CI= (4.13，15.38), P<0.00001).

C The relationship between CD147 expression and status of lymph node.CD147 expression was associated with status of lymph node(OR=5.14, 95%CI= (2.03,13.01), P=0.0005).

D The relationship between CD147 expression and tumor differentiation.CD147 expression wasn’t associated with tumor differentiation(OR=1.57, 95%CI= (0.79,3.12), P=0.19).

E The relationship between CD147 expression and age.CD147 expression wasn’t associated with age(OR=1.05, 95%CI= (0.72，1.53), P=0.79).

F The relationship between CD147 expression and sex.CD147 expression wasn’t associated with sex(OR=0.70,95%CI=(0.47,1.04),P=0.07).
