# Supplementary material for: Expression of Concern: The prognostic and clinicopathologic characteristics of CD147 and esophagus cancer: A meta-analysis
Source: PLoS One. 2023 Feb 22;18(2):e0282229. doi: 10.1371/journal.pone.0282229 (PMC9946197; doi:10.1371/journal.pone.0282229)
Supplement: S1 File — (ZIP) [file pone.0282229.s001.zip › CD147 and diff tissue type plot.docx]

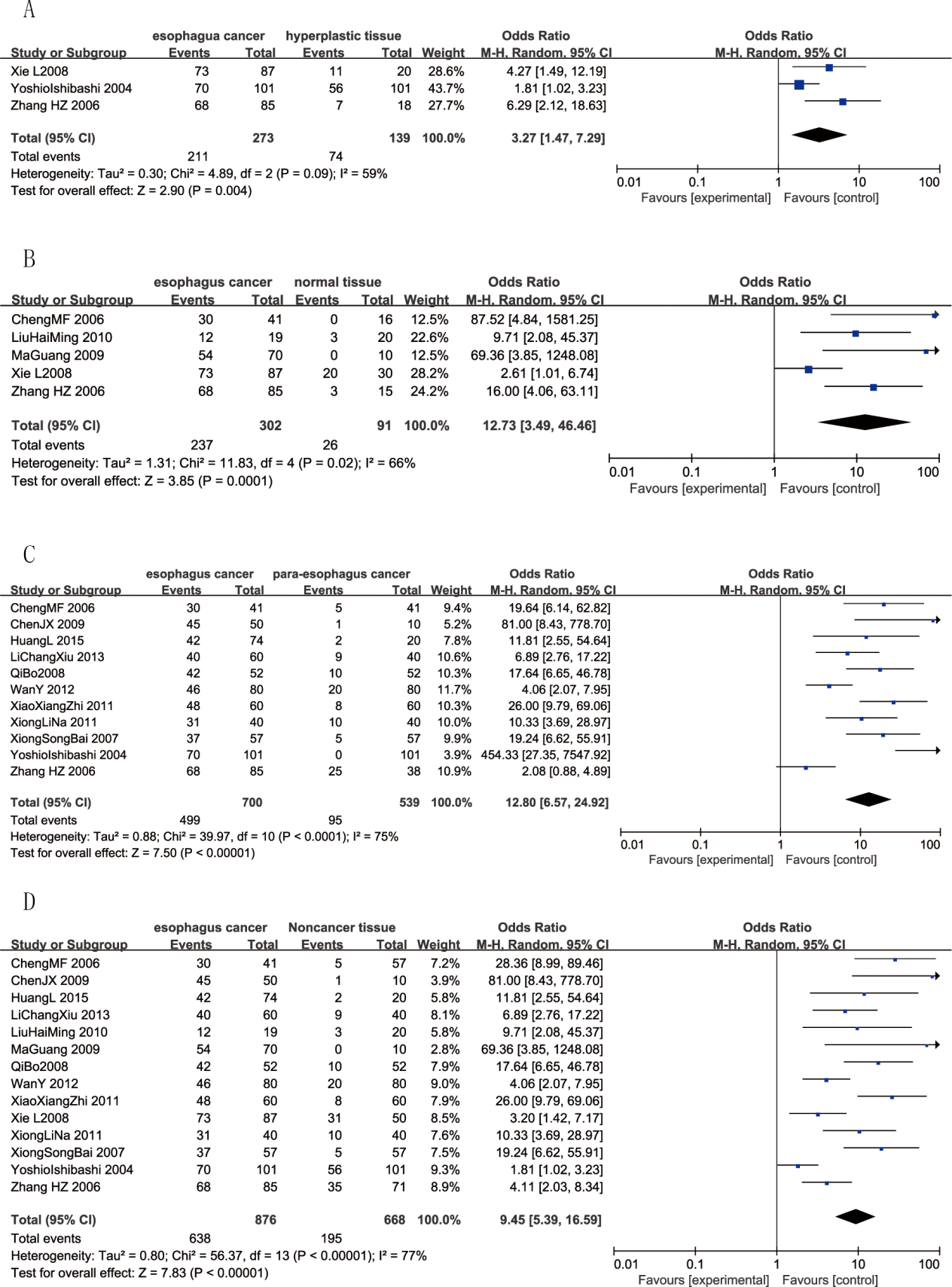


Fig.3. Forest plots of CD147 expression and different tissues. The squares and horizontal lines correspond to the study-specific OR and 95% CI.The area of the squares reflects the study-specific weight (inverse of the variance). The diamonds represent the pooled OR and 95% CI. The solid vertical line is at the null value (OR=1).

A CD147 positive expression between cancer and noncancer tissues.Significant difference was found between cancer and noncancer tissues(OR=9.45,95%CI=(5.39,16.59),P<0.00001).

B CD147 positive expression between cancer and normal tissues.Significant difference was found between cancer and normal tissues(OR= 12.73, 95%CI= (3.49,46.46), P=0.0001).

C CD147 positive expression between cancer and para-carcinoma tissues.Significant difference was found between cancer and para-carcinoma tissues(OR=12.80, 95%CI= (6.57,24.92), P<0.00001)

D CD147 positive expression between cancer and hyperplastic tissues.Significant difference was found between cancer and hyperplastic tissues(OR=3.27, 95% CI= (1.47,7.29), P=0.004).
