# Supplementary material for: Expression of Concern: The prognostic and clinicopathologic characteristics of CD147 and esophagus cancer: A meta-analysis
Source: PLoS One. 2023 Feb 22;18(2):e0282229. doi: 10.1371/journal.pone.0282229 (PMC9946197; doi:10.1371/journal.pone.0282229)
Supplement: S1 File — (ZIP) [file pone.0282229.s001.zip › CD147 and survival data plot.docx]

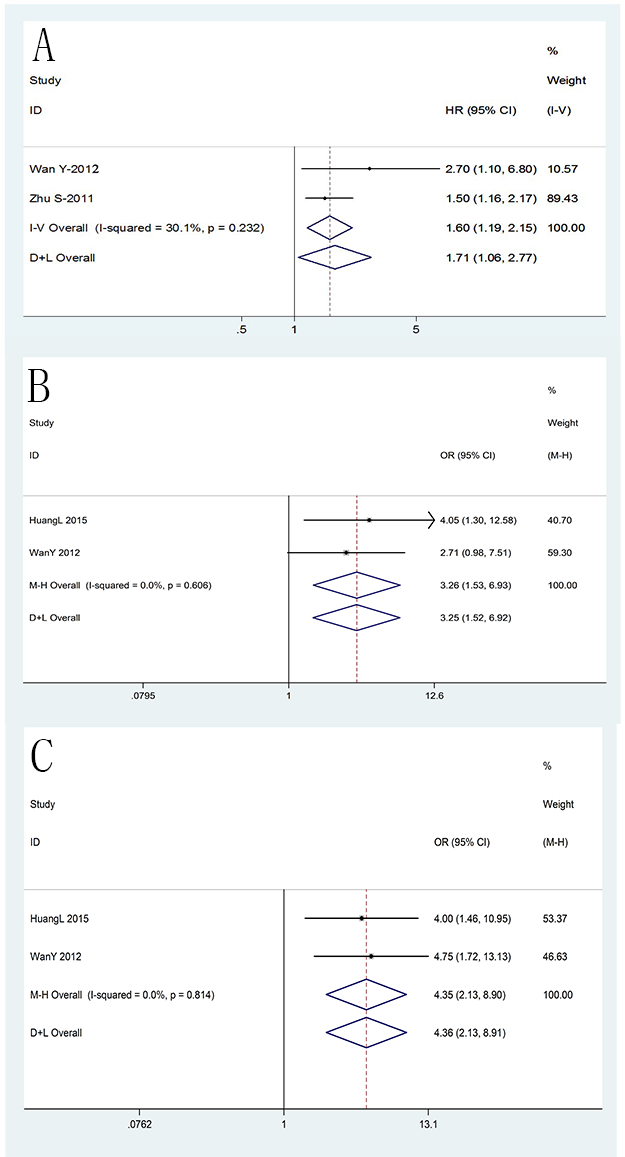


Fig.4. Forest plots of CD147 expression and the clinicopathological features of patients with esophagus cancer. The squares and horizontal lines correspond to the study- specific OR and 95% CI.The area of the squares reflects the study-specific weight (inverse of the variance). The diamonds represent the pooled OR and 95% CI. The solid vertical line is at the null value (OR=1).

A The relationship between CD147 expression and overall survival. CD147 expression was associated with overall survival（HR =1.60,95% CI =( 1.19,2.15),p=0.02).

B The relationship between CD147 expression and 3-year survival rate. CD147 expression was associated with 3-year survival rate (OR=3.26, 95%CI= (1.53,6.93),p=0.02).

C The relationship between CD147 expression and 5-year survival rate.CD147 expression was associated with 5-year survival rate(OR= 4.35,95%CI=(2.13,8.90),p<0.0001).
