# Supplementary material for: Expression of Concern: The prognostic and clinicopathologic characteristics of CD147 and esophagus cancer: A meta-analysis
Source: PLoS One. 2023 Feb 22;18(2):e0282229. doi: 10.1371/journal.pone.0282229 (PMC9946197; doi:10.1371/journal.pone.0282229)
Supplement: S1 File — (ZIP) [file pone.0282229.s001.zip › PDF of included paper/CD147 and MMP-9 expressions in typeó≥or ó≤ adenocarcinoma of esophagogastric junction and their clinicopathological significances.pdf]

Int J Clin Exp Pathol 2015;8(2):1929-1937
www.ijcep.com /ISSN:1936-2625/IJCEP0004256

Original Article

CD147 and MMP-9 expressions in type II/III adenocarcinoma of esophagogastric junction and their clinicopathological significances

Lei Huang[1,2], A-Man Xu[1,2], Qiang Peng[1]

[1]*Department of Gastrointestinal Surgery, The First Affiliated Hospital of Anhui Medical University, Hefei, China;* [2]*Department of Gastrointestinal Surgery, The Fourth Affiliated Hospital of Anhui Medical University, Hefei, China*

Received November 29, 2014; Accepted January 28, 2015; Epub February 1, 2015; Published February 15, 2015

Abstract: Objectives: To investigate CD147 and matrix metalloproteinase-9 (MMP-9) expressions in type II/III adenocarcinoma of esophagogastric junction (AEG), and their clinicopathological significances. Methods: Seventy-four patients clinically and pathologically diagnosed with type II/III AEG were analyzed, each undergoing radical total gastrectomy and Roux-en-Y esophagojejunostomy. The avidin streptavidin-perosidase immunohistochemistry technique was used to detect CD147 and MMP-9 in type II/III AEGs and 20 para-tumor controls, and their correlations with clinicopathological data and their reciprocal relationship were then analyzed. Kaplan-Meier survival analysis was conducted to reveal their prognostic significances. SPSS 20.0 was used for data analysis. A difference was statistically significant with $P < 0.05$, and very significant with $P < 0.01$. Results: In type II/III AEG CD147 and MMP-9 were mainly expressed on cellular membrane of in tumor cell cytoplasm. MMP-9 expression was significantly stronger at tumor-stroma junction and front edge of invasion. Their positive rates were significantly higher in malignant tissues than para-tumor tissues ($P < 0.01$ for both). There existed a significant positive correlation between both expressions ($P < 0.05$). They were significantly more highly expressed in cancers with lymphatic metastasis ($P < 0.01$ for both), at TNM III/IV stages ($P < 0.01$ for both), and with poor differentiation grade ($P < 0.05$ for both). Higher CD147 and MMP-9 expression rates were correlated with inferior postsurgical survivals ($P < 0.05$ for both). Conclusions: CD147 and MMP-9 could be novel biomarkers for type II/III AEG, and potentially predict tumor progression and prognosis. They are worth further investigation.

Keywords: Adenocarcinoma of esophagogastric junction, CD147, matrix metalloproteinase 9, tumor progression, prognosis

Introduction

Since the mid-1970s, the world has witnessed an increased incidence of adenocarcinoma of esophagogastric junction (AEG), while the morbidity of adenocarcinoma in distal gastric decreases year by year [1]. Due to its unique anatomic position and specific characteristics in epidemiology, etiology, clinical pathology, treatment and prognosis, AEG is gaining more and more attention as an independent disease [2]. For the moment the most effective treatment of AEG is radical resection, while the overall efficacy is not satisfactory due to the facts that the complex adjacent tissues are easily infiltrated, that tumors are mostly detected at advanced stage because of atypical early symptoms, and that there exist abundant complications after invasive surgical management [3]. Besides, tumor biological and anatomic features, including high incidence of tumor embolus, early and abundant lymphatic metastases, rich blood supply and lymphatic drainage of the esophagogastric junction (EGJ) facilitating malignant cell invasion and metastasis, high rate of poorly differentiated lesions, and easy local invasion or distant metastasis of tumor cells via vasculature due to gastric peristalsis, are also significant factors causing poor AEG prognosis [4].

Cancer patients mostly die of multiple organ failure caused by tumor progression, which also largely leads to failure of various therapeutic approaches including surgery and radiochemotherapy. Nowadays, early diagnosis and early

Table 1. Clinicopathological features of AEG[a] patients included

Item	Value
Gender (male/female)	56/18
Age (years)	62.2 ± 7.5 (41-76)[b]
Siewert classification (type II/III)	29/45
Location (great curvature/lesser curvature)	2/72
Tumor length (cm)	5.1 ± 3.0 (1.0-16.0)
Tumor width (cm)	3.9 ± 2.3 (1.0-11.0)
Tumor TNM classification (I and II/III and IV)	28/46
Differentiation (well/moderately/poorly)	19/25/30
Lymphatic metastasis (yes/no)	38/36
Abdominal metastasis (yes/no)	9/65
Liver metastasis (yes/no)	6/68

[a]AEG, adenocarcinoma of esophagogastric junction. [b]Measurement data are in mean ± standard deviation (range).

treatment are the fundamental approaches to improve prognosis [5]. Researchers worldwide are investigating deeply AEG genesis and progression, with the hope to come up with effective treatment.

Tumor invasion and metastasis are important complex multi-factor and multi-procedure processes causing recurrence and poor prognosis, with many genes and their products mediating malignant cell adhesion, migration, invasion, and angiogenesis involved in the regulation. In recent years, many AEG-associated prognostic molecular markers have been revealed, and it's a trend to assess prognosis based on clinical pathology and clinicobiochemistry [6]. Matrix metalloproteinase (MMP)-induced degradation of extracellular matrix (ECM) and basement membrane is a key factor promoting tumor progression. MMP-9 is one of the most important MMP members. CD147 is also called ECM metalloproteinase inducer, and can induce the expression and activation of MMPs, stimulate MMP secretion by tumor and stromal cells, and participate in the malignant-malignant cell and tumor cell-ECM interplays, thus degrading ECM and basement membrane, and promoting tumor progression [7]. Besides, CD147 could promote tumor angiogenesis via secreting vascular endothelial growth factor. CD147 paired with MMP-9 has been found to be highly expressed in various malignancies, and to have a positive correlation with malignant degree [8], suggesting their prognostic significances. However, relevant studies on AEG, which would have great significance due to the poor overall prognosis, have not been found.

This study examines CD147 and MMP-9 expressions in type II/III AEG, investigating their relationship with carcinogenesis, invasion and metastasis of AEG, and their inter-correlation, with the hope to provide gastroenterologists with novel evidences and directions facilitating early diagnosis, prognosis prediction and targeted therapy.

Materials and methods

Patients enrolled

A total of 78 potential patients clinically diagnosed with type II/III AEG [9] treated in the First Affiliated Hospital of Anhui Medical University from January 2004 to December 2006 were enrolled, each with detailed clinical data, operation record and follow-up results of more than 5 years. Three patients with squamous carcinoma and 1 with neuroendocrine carcinoma were excluded after pathological diagnosis, leaving 74 individuals as the experimental group. All patients did not receive any pre-surgical radiochemotherapy, and they all underwent total gastrectomy plus Roux-en-Y esophagojejunostomy. Tumors were categorized according to the TNM classification system proposed by American Joint Committee on Cancer (AJCC) in 2010 [10]. Information regarding the intra-abdominal findings with special reference to regional and distant metastasis was obtained from the surgical reports. Data regarding histological type and tumor penetration were gained from the pathological records. Follow-up was conducted via telephone. Characteristics of AEG patients in the experimental group are shown in **Table 1**. A group of 20 para-tumor tissues were selected as controls. Written informed consent was obtained from each patient. The procedures followed were approved by the institutional review board in accord with the ethical standards established by Ethics Committee of Anhui Medical University and are in accord with the Helsinki Declaration [11] and Good Clinical Practice [12].

Reagents

Mouse-anti-human monoclonal antibody of MMP-9 (MAB-0245) was purchased from Maixin Biotechnology Co. Ltd. in Fuzhou, China, and mouse-anti-human monoclonal antibody of

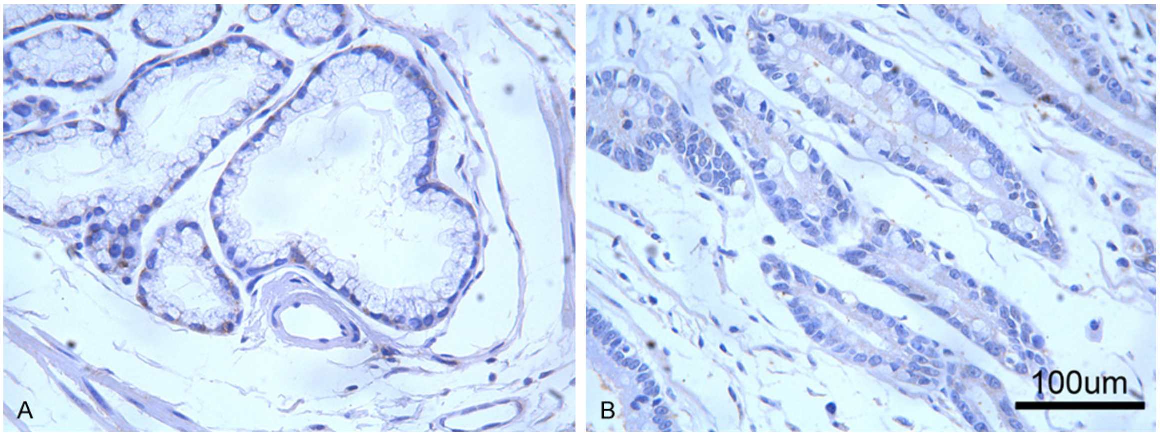

Figure 1. Expressions of CD147 and MMP-9 in para-AEG tissue. Immunohistochemical staining showed that CD147 (A) and MMP-9 (B) were very weakly or negatively expressed in para-AEG tissues (high power field, ×400). MMP-9, matrix metalloproteinase 9; AEG, adenocarcinoma of esophagogastric junction.

CD147 (ZM-0445) was obtained from Zhongshan Jinqiao Biological Product Co. Ltd. in Beijing, China. Diaminobenzidine (DAB) chromogenic and immunohistochemical kits were from Maixin Biotechnology Co. Ltd.

Immunohistochemistry

The avidin streptavidin-perosidase immunohistochemistry technique was used. Specimens were fixed in 4% neutral paraformaldehyde, conventionally embedded in paraffin, serially sectioned into 4 μm slices, and routinely stained using the HE method to further confirm the pathological diagnosis. Immunohistochemical staining was then performed. The paraffin sections were dewaxed by xylene, hydrated by gradient ethanol, emerged in 3% H_2O_2 solution, and incubated at room temperature (RT) for 10 minutes to inactivate endogenous peroxidases. Antigen was repaired in heated water bath using 0.01 mol/L citrate buffer (pH = 6.0, 95-100°C, 20 minutes). The specimens were washed by phosphate buffer saline (PBS) liquid after cooled at RT, added with mouse-anti-human CD147 monoclonal antibody, and mouse-anti-human MMP-9 monoclonal antibody, and then incubated at a constant temperature of 37°C for 2 hours. Then they were rinsed with PBS for 3 times (5 minutes per time), added with biotin-labeled secondary antibody, incubated at RT for 30 minutes, and washed again by PBS for 3 times (5 minutes per time). DAB was applied as the chromogenic agent, and the specimens were flushed with distilled water, stained by hematoxaylin, dehydrated by ethanol, made transparent by xylene, and sealed with neutral gum. The glioblastoma multiforme specimen with CD147 and MMP-9 positively expressed was selected as the positive control, and the negative control was obtained after replacing the primary antibody with PBS.

Assessment criteria

Positive CD147 expression was shown as yellow or brown granule in cytoplasm or on cellular membrane, and positive MMP-9 expression as brown granule in cytoplasm. Observed in high power field (HPF, × 400), randomly 5 fields of malignant cells were examined for each section, and the percentage of positively stained cells was calculated based on 1000 fields with 200 cells in each field. The result was determined using semi-quantitative scoring, based on both number of positively stained cells and color depth: 0 point for positive cell percentage ≤ 5%, 1 point for 6%-25%, 2 points for 26%-50%, 3 points for 51%-75%, and 4 points for > 75%. Concerned with color depth of positive cells, 0 point for basically no coloration, 1 point for light yellow, 2 points for pale brown, and 3 points for dark brown. After multiplying the 2 scores, we got a negative result (-) for 0-2 points (**Figure 1**), weakly positive (+) for 3-4 points, moderately positive (++) for 5-8 points, and strongly positive (+++) for 9-12 points (**Figure 2**). Positive expressions were marked as +, ++, and +++. Staining results were verified by 2 independent pathologists using the double blind procedure. In case of discrepancy, a third

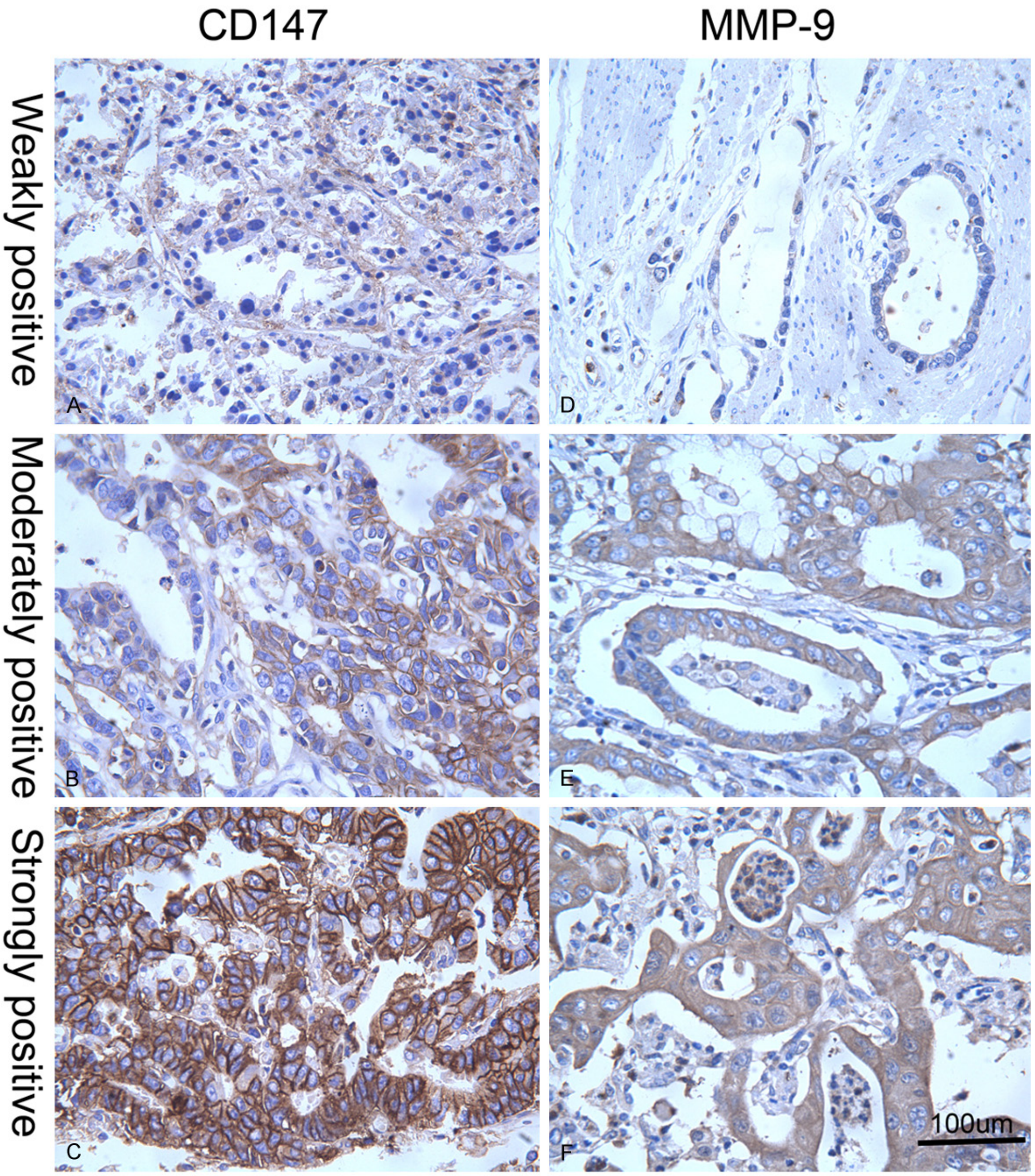

Figure 2. Different expression levels of CD147 and MMP-9 in AEG tissues. Revealed by immunohistochemical staining in AEG tissues, weakly positive (A), moderately positive (B), and strongly positive (C) CD147 expressions were indicated by yellow or brown color in cytoplasm or on cellular membrane, and weakly positive (D), moderately positive (E), and strongly positive (F) MMP-9 expressions were illustrated by brown color in cytoplasm (high power field, × 400). MMP-9, matrix metalloproteinase 9; AEG, adenocarcinoma of esophagogastric junction.

senior investigator was consulted and agreement was reached by consensus.

Statistical analyses

SPSS 20.0 software was applied. We compared differences of enumeration data between different groups using χ^2 test, and evaluated the correlations between expressions of CD147 and MMP-9 using Spearman rank correlation test with coefficient r_s calculated. A difference was statistically significant with $P < 0.05$, and very significant with $P < 0.01$.

Table 2. Expressions of CD147 and MMP-9[a] in type II/III AEG[b] and para-tumor tissues

Tissue type	CD147		MMP-9	
	-	+-+++	-	+-+++
Para-tumor tissue	18	2	17	3
Type II/III AEG tissue	32	42	24	50
χ^2	13.825		17.692	
P	0.000		0.000	

[a]MMP-9, matrix metalloproteinase 9; [b]AEG, adenocarcinoma of esophagogastric junction.

Results

Expressions and distributions of CD147 and MMP-9 in type II/III AEG and para-cancerous tissues

CD147 was mainly expressed on cellular membrane or in the cytoplasm of tumor cells, and MMP-9 was majorly expressed in the cytoplasm of malignant cells. In glands and stromal cells of para-tumor tissues, both were very weakly or negatively expressed (**Figure 1**). MMP-9 expression was significantly stronger at tumor-stroma junction and the front edge of malignant infiltration (**Figure 2**). Correlations of CD147 and MMP-9 expressions in type II/III AEG and paratumorous tissues are shown in **Table 2**. The positive rates of CD147 in para-tumor and type II/III AEG tissues were 10% (2/20) and 56.8% (42/74) respectively, and the positive rates of MMP-9 in para-tumor and malignant tissues were 15% (3/20) and 67.6% (50/74) respectively. Compared with para-tumor mucosa, positive expression rates of CD147 and MMP-9 in type II/III AEG tissue were significantly higher ($P = 0.000$ for both).

Correlations between CD147 expression in type II/III AEG tissue and clinicopathological parameters

The correlations are shown in **Table 3**. Positive rate of CD147 expression was not significantly correlated with patients' age, gender, tumor size or Siewert classification ($P > 0.05$ for all), but was significantly higher in tumors with lymphatic metastasis than those without metastasis ($P = 0.003$), and in TNM III/IV stage tumors than I/II ones ($P = 0.004$). The more poorly differentiated a tumor was, the higher possibly CD147 was positively expressed ($P = 0.036$). Besides, liver ($P = 0.072$) and abdominal metastases ($P = 0.086$) tended to be associated with higher rate of positive CD147 expression.

Correlations between MMP-9 expression in type II/III AEG tissue and clinicopathological parameters

The correlations are shown in **Table 3**. Positive rate of MMP-9 expression was not significantly correlated with patient's age, gender, tumor size or Siewert classification ($P > 0.05$ for all), but was significantly higher in tumors with lymphatic metastasis than those without metastasis ($P = 0.000$), and in TNM stage III/IV tumors than I/II ones ($P = 0.002$). The more poorly differentiated a tumor was, the higher possibly MMP-9 was positively expressed ($P = 0.021$).

Correlations between expressions of CD147 and MMP-9 and survival

The correlations are shown in **Table 4**. Positive rates of CD147 and MMP-9 expressions were significantly higher among patients with post-surgical survival time < 3 years than those ≥ 3 years ($P < 0.05$ for both), and with post-operational survival time < 5 years than those ≥ 5 years ($P < 0.01$ for both).

Correlation between CD147 and MMP-9 expressions in type II/III AEG tissue

CD147 and MMP-9 were mostly co-expressed in malignant tissue (**Table 5**). There existed a positive correlation between CD147 and MMP-9 expressions in type II/III AEG tissue ($r_s = 0.351$, $P = 0.03$).

Discussion

Though many controversies remain, Siewert's suggestion that adenocarcinomas locating within 5 cm from the cardia be defined as AEG which could be divided into 3 types has been widely accepted by International Gastric Cancer Association and International Society for Disease of Esophagus [13]. Type II/III is major, with type I uncommonly seen, and they differ greatly in etiology, epidemiology, origin, tumor biology, patient's medical history, and prognosis, making type II/III AEG considered an independent cancer [9]. Type II/III AEG originates from the cardiac mucosa in EGJ or the segmental intestinal epithelium metaplasia zone, and

Table 3. Correlation between CD147 and MMP-9[a] expressions in type II/III AEG[b] tissues and clinicopathological parameters

Clinicopathological indexes	CD147 expression		χ^2	*P*	MMP-9 expression		χ^2	*P*
	-	+-+++			-	+-+++		
Age (years)								
≤ 50	15	26			12	29		
> 50	17	16	1.660	0.198	12	21	0.420	0.517
Gender								
Male	24	32			18	38		
Female	8	10	0.014	0.906	6	12	0.009	0.925
Tumor length (cm)								
< 2	7	8			8	7		
2-5	14	24			10	28		
> 5	11	10	1.420	0.492	6	15	3.782	0.151
Lymphatic metastasis								
+	10	28			5	33		
-	22	14	9.119	0.003	19	17	13.242	0.000
Liver metastasis								
+	6	0			6	0		
-	36	32	3.242	0.072	44	24	1.730	0.188
Abdominal metastasis								
+	8	1			8	1		
-	34	31	2.949	0.086	42	23	1.162	0.281
Histological differentiation grade								
Well	12	7			12	7		
Moderately	12	13			10	15		
Poorly	8	22	6.659	0.036	7	23	7.752	0.021
TNM Classification								
Stage I/II	18	10			15	13		
Stage III/IV	14	32	8.126	0.004	9	37	9.185	0.002
Siewert classification								
Type II	12	17			11	18		
Type III	20	25	0.068	0.795	13	32	0.658	0.417

[a]MMP-9, matrix metalloproteinase 9; [b]AEG, adenocarcinoma of esophagogastric junction.

has greater invasion ability than tumors in other parts of a gastric [2]. Investigating the prognosticators, tumor biomarkers for early detection, and targeted drugs against AEG will show great significance in improving the overall survival.

Post-surgical prognosis of AEG is affected by various clinicopathological factors including tumor size, histological type, local invasion, lymphatic metastasis, and remnant cancer cells at cutting edge [14]. The molecular biological prognosticator has become a hot topic of research worldwide. Tumor invasion and metastasis are a major barrier of cancer treatment and a main reason causing most cancer deaths [15]. The basement membrane and ECM form the histological barrier to prevent the malignant processes, and their degradation facilitates tumor progression. Investigating the mechanism of malignant invasion and metastasis is essential to uncovering effective anti-tumor treatment and improving prognosis.

Studying functions of CD147 and MMP-9 during malignant progression is significant. This study, examining CD147 and MMP-9 in type II/III AEG tissues, revealed that their expressions were significantly higher among tumors with lymphatic metastasis, poor histological differentiation grade, and late TNM stage. Besides, higher

Table 4. Correlation between CD147 and MMP-9[a] expression and post-surgical survival

Survival years	n	CD147		X^2	P	MMP-9		X^2	P
		-	+-+++			-	+-+++		
≥ 5 years									
Yes	42	24	18			19	23		
No	32	8	24	7.645	0.006	5	27	7.268	0.007
≥ 3 years									
Yes	51	27	24			21	30		
No	23	5	18	6.288	0.012	3	20	5.725	0.017

[a]MMP-9, matrix metalloproteinase 9.

Table 5. Correlation between CD147 and MMP-9[a] expressions in type II/III AEG[b] tissue

MMP-9	CD147	
	-	+-+++
-	18	6
+-+++	14	36
X^2	14.595	
P	0.000	

[a]MMP-9, matrix metalloproteinase 9; [b]AEG, adenocarcinoma of esophagogastric junction.

CD147 expression rate tended to be correlated with liver and abdominal metastases. However, both expressions were not significantly correlated with patients' age, gender, tumor size or Siewert classification. Our study indicated that their expressions could suggest tumor cell invasive ability and prognosis. We further found that postoperative survivals less than 3 years and less than 5 years were both correlated with higher expression rates of CD147 and MMP-9.

MMPs could degrade almost all ECM components except polysaccharide, break the balance of matrix degradation, and promote tumor cells to surmount the histological barrier, facilitating local invasion and distal metastasis. CD147, the MMP inducer, could promote the secretion of MMPs, also facilitating tumor progression [16]. CD147 is involved in diverse physiological procedures via cell-cell and cell-stroma adhesions [17]. CD147 produced by malignant cells could induce angiogenesis by stimulating MMP secretion of fibroblasts and endothelial cells through paracrine, thus degrading various ECM proteins, and breaking the barrier which prevents tumor cell escape. Our study also revealed a significant positive correlation between CD147 and MMP-9 expressions in type II/III AEG tissues. Change of the intracellular C-terminal structure of CD147 would affect the polymerization and dissociation of the cytoskeleton proteins, participating cancer cell mobility. CD147 along with MMPs could be tightly attached onto surface of malignant cells, enhancing tumor stroma degradation via MMPs, creating a favorable tumor microenvironment for malignant infiltration and metastasis. Accordingly, we found CD147 and MMP-9 mainly expressed on cellular surface of in the cytoplasm of tumor cells.

In breast cancer, melanoma, lung cancer, and colorectal carcinoma, CD147 and MMP expression both increase, and correlate positively with malignant degree, tumor invasion and metastasis [7]. We detected great positive CD147 and MMP-9 expression rates in type II/III AEG, which was significantly higher than para-tumor mucosa. According to our study, CD147 was more highly expressed in tumors with metastasis, and interestingly, there was a significantly stronger MMP-9 expression at tumor-stroma junction and the front edge of tumor invasion than in the malignant tissue, which could be possibly explained by the facts that activated MMP-9 mainly locates in the protruding parts of tumor cell-penetrated matrix, and shares similar drill-like functions with MMP-2 in malignant tissues, degrading ECM and facilitating tumor invasion [18], and that the highly expressed CD147 in cancer cells could stimulate stromal cells to produce MMPs, facilitating tumor growth, invasion and metastasis [19]. However, how CD147 induces MMP generation remains obscure.

MMPs are usually produced by fibroblasts, macrophages, neutrophils and cancer cells. MMPs majorly promote tumor invasive growth via the following possible mechanisms. 1. They degrade the extracellular stroma, breaking the para-tumor barrier. 2. They remodel intercellular adhesive ability, facilitating tumor cell anchoring and metastasis. 3. They reconstruct ECM, promoting angiogenesis. 4. They could activate each other. In the family, MMP-9 is

generally thought to be most closely linked with tumor invasion and metastasis, and has a wide distribution of substrates, with type IV/V collagen and gelatin as the major ones. MMP-9 is also called type IV collagenase and is secreted as proenzyme. After activated, it could degrade various proteins in the para-tumor ECM, facilitating tumor progression. MMP-9 is rarely expressed in normal tissues, and is involved in various physiological and pathological processes. We also found it very weakly or negatively expressed in para-tumor tissues. However, during carcinogenesis and tumor progression, it's always over-expressed, and correlates positively with CD147 and malignant degree. In our study, we also found that CD147 and MMP-9 were largely co-expressed in malignant tissues. Based on the fact that CD147 and MMP-9 expressions might have significant correlations with tumor invasion and metastasis, discovery of blockers against them might effectively inhibit tumor progression and improve prognosis.

Enlightened by this study, how CD147 modulates expression of MMPs degrading ECM, what the key factors involved are, and how they interact with each other in the molecular biological level would be worth further investigation with the hope to further clarify how AEG evolves, develops, invades and migrates, facilitating diagnosis and treatment.

In conclusion, in type II/III AEG CD147 and MMP-9 are significantly more highly expressed than para-tumor tissue, and MMP-9 expression is stronger at the front edge of tumor invasion. They are positively correlated with each other. Both expressions correlate with lymphatic metastasis, TNM stage, and histological differentiation grade. Higher CD147 and MMP-9 expression rates are correlated with inferior postsurgical survival. It's worthy further investigating them which potentially indicate tumor progression and prognosis in AEG.

Acknowledgements

This work was supported by Foundation of Anhui Science and Technology Agency [grant number 12070403061]. The funder had no role in study design, data collection and analysis, decision to publish, or preparation of the manuscript.

Disclosure of conflict of interest

None.

Address correspondence to: Dr. A-Man Xu, Department of Gastrointestinal Surgery, The First Affiliated Hospital of Anhui Medical University, 218 Jixi Rd, Hefei 230022, China. Tel: +86-551-65334247; Fax: +86-551-63633742; E-mail: amanxu@163.com

References

[1] Jemal A, Bray F, Center MM, Ferlay J, Ward E and Forman D. Global cancer statistics. CA Cancer J Clin 2011; 61: 69-90.
[2] Suh YS, Han DS, Kong SH, Lee HJ, Kim YT, Kim WH, Lee KU and Yang HK. Should adenocarcinoma of the esophagogastric junction be classified as esophageal cancer? A comparative analysis according to the seventh AJCC TNM classification. Ann Surg 2012; 255: 908-915.
[3] Mine S, Sano T, Hiki N, Yamada K, Kosuga T, Nunobe S, Shigaki H and Yamaguchi T. Thoracic lymph node involvement in adenocarcinoma of the esophagogastric junction and lower esophageal squamous cell carcinoma relative to the location of the proximal end of the tumor. Ann Surg Oncol 2014; 21: 1596-1601.
[4] Hasegawa S, Yoshikawa T, Rino Y, Oshima T, Aoyama T, Hayashi T, Sato T, Yukawa N, Kameda Y, Sasaki T, Ono H, Tsuchida K, Cho H, Kunisaki C, Masuda M and Tsuburaya A. Priority of lymph node dissection for Siewert type II/III adenocarcinoma of the esophagogastric junction. Ann Surg Oncol 2013; 20: 4252-4259.
[5] Huang L, Xu A, Li T, Han W, Wu S and Wang Y. Detection of perioperative cancer antigen 72-4 in gastric juice pre- and post-distal gastrectomy and its significances. Med Oncol 2013; 30: 651.
[6] Xu AM, Huang L, Han WX and Wei ZJ. Monitoring of peri-distal gastrectomy carbohydrate antigen 19-9 level in gastric juice and its significance. Int J Clin Exp Med 2014; 7: 230-238.
[7] Kong LM, Liao CG, Zhang Y, Xu J, Li Y, Huang W, Zhang Y, Bian H and Chen ZN. A regulatory loop involving miR-22, Sp1, and c-Myc modulates CD147 expression in breast cancer invasion and metastasis. Cancer Res 2014; 74: 3764-3778.
[8] Omi Y, Shibata N, Okamoto T, Obara T and Kobayashi M. The role of CD147 in the invasiveness of follicular thyroid carcinoma cells. Thyroid 2012; 22: 383-394.
[9] Huang L and Xu AM. Adenocarcinoma of esophagogastric junction: controversial classi-

fication, surgical management, and clinicopathology. Chin J Cancer Res 2014; 26: 226-230.
[10] Edge SB and Compton CC. The American Joint Committee on Cancer: the 7th edition of the AJCC cancer staging manual and the future of TNM. Ann Surg Oncol 2010; 17: 1471-1474.
[11] Morris K. Revising the Declaration of Helsinki. Lancet 2013; 381: 1889-1890.
[12] Grimes DA, Hubacher D, Nanda K, Schulz KF, Moher D and Altman DG. The Good Clinical Practice guideline: a bronze standard for clinical research. Lancet 2005; 366: 172-174.
[13] Siewert JR and Stein HJ. Classification of adenocarcinoma of the oesophagogastric junction. Br J Surg 1998; 85: 1457-1459.
[14] Mariette C, Piessen G, Briez N, Gronnier C and Triboulet JP. Oesophagogastric junction adenocarcinoma: which therapeutic approach? Lancet Oncol 2011; 12: 296-305.
[15] Gilkes DM, Semenza GL and Wirtz D. Hypoxia and the extracellular matrix: drivers of tumour metastasis. Nat Rev Cancer 2014; 14: 430-439.
[16] Bernard SC, Simpson N, Join-Lambert O, Federici C, Laran-Chich MP, Maissa N, Bouzinba-Segard H, Morand PC, Chretien F, Taouji S, Chevet E, Janel S, Lafont F, Coureuil M, Segura A, Niedergang F, Marullo S, Couraud PO, Nassif X and Bourdoulous S. Pathogenic Neisseria meningitidis utilizes CD147 for vascular colonization. Nat Med 2014; 20: 725-731.
[17] Yurchenko V, Constant S, Eisenmesser E and Bukrinsky M. Cyclophilin-CD147 interactions: a new target for anti-inflammatory therapeutics. Clin Exp Immunol 2010; 160: 305-317.
[18] Huang Q, Lan F, Wang X, Yu Y, Ouyang X, Zheng F, Han J, Lin Y, Xie Y, Xie F, Liu W, Yang X, Wang H, Dong L, Wang L and Tan J. IL-1beta-induced activation of p38 promotes metastasis in gastric adenocarcinoma via upregulation of AP-1/c-fos, MMP2 and MMP9. Mol Cancer 2014; 13: 18.
[19] Wu J, Hao ZW, Zhao YX, Yang XM, Tang H, Zhang X, Song F, Sun XX, Wang B, Nan G, Chen ZN and Bian H. Full-length soluble CD147 promotes MMP-2 expression and is a potential serological marker in detection of hepatocellular carcinoma. J Transl Med 2014; 12: 190.
