# Supplementary material for: Expression of Concern: The prognostic and clinicopathologic characteristics of CD147 and esophagus cancer: A meta-analysis
Source: PLoS One. 2023 Feb 22;18(2):e0282229. doi: 10.1371/journal.pone.0282229 (PMC9946197; doi:10.1371/journal.pone.0282229)
Supplement: S1 File — (ZIP) [file pone.0282229.s001.zip › PDF of included paper/CD147PTEN E╥╗╕╞≡ñ╡░░╫╘┌╩│╣▄░⌐╫Θ╓»╓╨▒φ┤∩╙δ┴┘┤▓▓í└φ╡─╣╪╧╡.pdf]

・论　著・

CD147 PTEN E-钙黏蛋白在食管癌组织中表达与临床病理的关系*

李昌秀[1],徐红梅[1],杨达荣[1],杨　柳[1],吴时军[1],张　黎[1],罗治彬[2△](重庆市合川区人民医院:1.病理科;2.肿瘤科　401520)

【摘要】 **目的**　探讨 CD147 与 PTEN 及 E-钙黏蛋白(E-cadherin)在食管癌组织中的表达,以期了解三者在食管癌发生、发展及转移中的意义。**方法**　采用免疫组化法分别检测 CD147、PTEN 及 E-cadherin 在 60 例食管癌组织中的表达情况。**结果**　CD147 在食管癌组织中的表达明显高于在正常食管鳞状上皮中的表达($P<0.01$)。而 PTEN 及 E-cadherin 在食管癌中表达明显低于二者在正常食管组织中的表达($P<0.01$)。在食管癌中 CD147 的表达与 E-cadherin 的表达呈负相关,且与浸润深度及淋巴结转移呈正相关,而 CD147 与 PTEN 表达及分化程度无明显相关。PTEN 及 E-cadherin 的表达与淋巴结转移均呈负相关。**结论**　CD147、PTEN 及 E-cadherin 共同参与食管癌的发生且影响癌细胞转移。联合检测 CD147、PTEN 及 E-cadherin 有望成为食管癌预后评估的重要生物学指标。

【关键词】 食管癌;　CD147;　PTEN;　E-钙黏蛋白;　免疫组化

DOI:10.3969/j.issn.1672-9455.2013.02.001　文献标志码:A　文章编号:1672-9455(2013)02-0129-03

The expressions of CD147, PTEN and E-cadherin in esophageal carcinoma and their correlation with clinical-pathological characteristics　*LI Chang-xiu[1], XU Hong-mei[1], YANG Da-rong[1], YANG Liu[1], WU Shi-jun[1], ZHANG Li[1], LUO Zhi-bin[2△] (1. Department of Pathology; 2. Department of Oncology, People's Hospital of Hechuan District, He Chuan, Chongqing* 401520, *China*)

【Abstract】 **Objective**　To investigate the expressions of CD147, PTEN and E-cadherin in the esophageal carcinoma, and to explore their interactions in progression and metastasis of the esophageal carcinoma. **Methods**　The expressions of CD147, PTEN and E-cadherin were evaluated in 60 cases with esophageal carcinoma by immunohistochemical staining methods. **Results**　The positive rate of CD147 was significantly higher in esophageal carcinoma than that in normal squamous cell of esophagus($P<0.01$). The positive rate of PTEN and of E-cadherin was significantly lower in esophageal carcinoma than that in normal squamous cell of esophagus($P<0.01$). The expression of CD147 was negatively correlated with E-cadherin, and it was positively correlated with infiltration depth and the lymph-node metastasis. But the differentiating degree was not correlated with CD147 and E-cadherin. The expression of PTEN and E-cadherin were negatively correlated with the lymph-node metastasis. **Conclusion**　CD147, PTEN and E-cadherin may play the important roles in esophageal carcinoma development and lymph node metastasis. And combined detection of CD147, PTEN and E-cadherin may be helpful in evaluation of the biological prognosis of esophageal carcinoma.

【Key words】 esophageal carcinoma;　CD147;　PTEN;　E-cadherin;　immunohistochemical staining

CD147 又称促细胞外基质金属蛋白酶因子,高度表达在肿瘤细胞的表面,刺激肿瘤细胞周围的成纤维细胞分泌基质金属蛋白酶,降解基底膜及围绕肿瘤细胞的基质而促进肿瘤细胞侵袭、转移[1-2]。PTEN 是具有脂质磷酸酶活性的抑癌基因,对肿瘤细胞的增殖、迁移、侵袭、黏附有抑制作用。E-钙黏蛋白(E-cadherin)主要存在于人和动物的上皮细胞,介导细胞间的黏附作用,对维持正常的上皮细胞形态和结构完整有重要作用。近来研究表明,在一些恶性肿瘤中 CD147 与 PTEN 及 E-cadherin 存在一定的负相关[3-6]。作者采用免疫组织化学方法检测食管癌组织中 CD147、PTEN、E-cadherin 蛋白的表达,探讨三者在食管癌发生、发展及转移中的关系及意义。

1　资料与方法

1.1　一般资料　收集本院 2005～2011 年食管癌根治切除的石蜡标本 60 例。60 例食管癌组织标本经病理组织学检查证实为鳞状细胞癌,术前均未进行放疗及化疗。其中男 38 例,女 22 例;年龄 46～79 岁,平均(63.4±7.6)岁;有淋巴结转移 36 例,无淋巴结转移 24 例。癌旁正常黏膜组织(距癌组织 3 cm 以上,经病理检查证实为分化良好的鳞状上皮及少量固有层成分)40 例作为正常对照。

1.2　试剂与方法　CD147 单克隆抗体、PTEN 单克隆抗体、E-cadherin 单克隆抗体、SP 试剂盒均及 DAB 显色液购自福建迈新生物工程有限公司。组织经 10%甲醛固定,4 μm 连续石蜡切片。采用 SP 免疫组化染色法,按照 SP 检测试剂盒说明书的步骤操作。

1.3　结果的判定　参照文献[7],综合考虑切片中阳性细胞占所观察同类细胞数的百分比和阳性细胞着色强度两项指标,半

*　**基金项目:**重庆市合川区科技计划项目(201008)。　△　**通讯作者**,E-mail:luozhibincq@126.com。

定量判断结果。即先在低倍镜下选取抗体表达部位(细胞质、膜或核)的最强着色区,然后选 10 个高倍镜视野(×400),分别记数 100 个细胞,其颜色深浅参考文献评分:无棕黄色颗粒为 0 分、淡黄色为 1 分、棕黄色为 2 分、棕褐色为 3 分。染色细胞所占百分比评分:无阳性细胞为 0 分、阳性细胞 1%~10%为 1 分、11%~50%为 2 分、≥51%为 3 分。两项乘积得分大于或等于 5 分定为阳性。

1.4 统计学方法 采用 SPSS 13.0 软件进行分析,计数资料进行 χ^2 检验;两个变量相关性分析采用双变量相关分析,$P<0.05$ 为差异有统计学意义。

2 结 果

2.1 CD147、PTEN、E-cadherin 在食管癌中的表达 CD147 蛋白阳性产物为棕黄色颗粒,位于食管癌细胞胞质内或胞膜上,为强表达;E-cadherin 在正常食管鳞状上皮中表达于细胞膜。PTEN 在正常食管鳞状上皮中表达于细胞核,为强表达;CD147 在食管癌组织中的表达明显高于在正常食管鳞状上皮中的表达($P<0.05$),差异有统计学意义。而 PTEN 及 E-cadherin 在食管癌中表达分别为 43.33%和 38.33%,明显低于二者正常食管组织中的阳性率($P<0.01$),差异有统计学意义(表 1)。

2.2 CD147、PTEN、E-cadherin 在食管癌中的表达与临床病理的关系 在食管癌中 CD147 表达与 E-cadherin 的表达、分化程度呈一定的负相关,而与浸润深度及淋巴结转移呈正相关,且与 PTEN 无明显相关性。分化程度与 PTEN 及 E-cadherin 的表达无明显相关。PTEN 及 E-cadherin 与淋巴结转移的表达呈负相关。而 PTEN、E-cadherin 及 CD147 在食管癌中的表达与患者的性别及年龄无相关性($P>0.05$),差异无统计学意义(表 2)。

表 1 CD147、PTEN 及 E-cadherin 在食管癌中的表达

组别	n	CD147			PTEN			E-cadherin		
		阳性(n)	阴性(n)	阳性率(%)	阳性(n)	阴性(n)	阳性率(%)	阳性(n)	阴性(n)	阳性率(%)
正常食管组织	40	9	31	22.50	37	3	92.50	36	4	90.00
食管癌	60	40	20	66.67*	26	34	43.33*	23	37	38.33*

注:与正常食管组织比较,* $P<0.01$。

表 2 CD147 与 PTEN 及 E-cadherin 在食管癌中的表达与临床病理的关系

项目		n	PTEN 阳性	PTEN 阴性	χ^2	r	E-cadherin 阳性	E-cadherin 阴性	χ^2	r	CD147 阳性	CD147 阴性	χ^2	r
男		38	17	21	0.080	0.036△	16	22	0.618	0.101△	25	13	0.0314	0.023△
女		22	9	13			7	15			15	7		
年龄	≥50	44	19	25	2.570	0.203△	17	27	0.060	0.032△	29	15	0.0423	0.027△
	<50	16	7	9			6	10			11	5		
分化程度	高分化	19	13	6	7.170	0.327*	12	7	7.608	0.335*	6	13	16.377	−0.463*
	中分化	15	6	9			5	10			11	4		
	低分化	26	7	19			6	20			23	3		
浸润深度	黏膜/黏膜下层	19	17	2	23.817	−0.533*	15	4	19.445	−0.494*	5	14	20.372	0.503*
	肌层/外膜层	41	9	32			8	33			35	6		
有淋巴结转移		36	8	28	30.100	−0.584*	3	33	34.200	−0.603*	34	2	31.295	0.585*
无淋巴结转移		24	18	6			20	4			6	18		
CD147 阳性		40	19	21	0.848	0.118△	12	28	35.308	−0.609*	—	—	—	—
CD147 阴性		20	7	13			11	9			—	—		

注:* $P<0.01$;△ $P>0.05$;—表示无数据。

3 讨 论

食管鳞状细胞癌是食管恶性肿瘤中最常见的组织学类型,致死率较高,而肿瘤细胞的浸润转移是导致死亡的重要原因之一,也是治疗的关键。因此对肿瘤细胞浸润及转移的具体机制的研究对食管癌的治疗及预后具有重要意义。CD147 能促进肿瘤细胞及肿瘤间质成纤维细胞产生基质金属蛋白酶(MMPs),降解细胞外基质,参与肿瘤的浸润及转移。众多研究表明,CD147 在多种肿瘤细胞中高表达,与淋巴结转移呈正相关,并且可以作为肿瘤预后的指标之一[8-9]。本研究显示,食管癌组织中 CD147 的表达阳性率为 66.67%,较正常食管组织中 CD147 的表达明显升高,且与肿瘤的浸润深度($r=0.503$,$P<0.01$)、淋巴结转移($r=0.585$,$P<0.01$)呈正相关。这与 Zhu 等[10]对 86 例食管癌病例的研究结果基本一致。而且 Pan 等[11]通过 RNA 干扰技术对胰腺癌中 CD147 基因进行沉默后,胰腺癌细胞中 CD147 的 mRNA 及蛋白表达明显下降,同时细胞的转移能力降低。这进一步说明 CD147 参与肿瘤细胞的浸润及转移,并且可作为恶性肿瘤独立的预后因子及基因治疗的靶向因子。

肿瘤细胞脱离原发部位是肿瘤细胞发生浸润及转移的前提。E-cadherin 是细胞表面黏附分子,其表达量与细胞间的黏附能力呈正相关。在多种恶性肿瘤特别是有转移性癌细胞中 E-cadherin 的表达明显减少[12]。作者对 60 例食管癌组织中 E-cadherin 表达结果分析发现,食管癌中的 E-cadherin 阳性表达率(38.33%)显著低于正常食管组织(87.5%)($P<0.01$),且淋巴结转移组中 E-cadherin 表达明显低于无淋巴结转移组($P<0.01$),这提示 E-cadherin 参与了食管癌细胞浸润及转移。作者进一步发现 E-cadherin 与 CD147 呈负相关($r=-0.609$,$P<0.01$)。Wang 等[2]应用原位杂交技术检测 220 例胃癌组织中 CD147 及 E-cadherin mRNA 阳性率分别为 50.5%及 17.7%,CD147 与 E-cadherin 表达呈负相关。作者推测在促进肿瘤浸润及转移的过程中 E-cadherin 与 CD147 之间是否也存在某种相互调控的机制。而且 Nakamura 等[13]用 CD147 siRNA 作用于子宫内膜癌细胞株后细胞中 CD147 的表达降低,而 E-cadherin 的表达增加,但是其机制还不是很明确。

PTEN 基因作为第一个具有双特异性磷酸酶活性的抑癌基因,其在众多恶性肿瘤中存在突变、缺失或低表达,导致其抑癌功能减弱或丧失。本研究结果也显示食管癌中 PTEN 的阳性表达率(43.33%)显著低于正常食管组织(92.5%)($P<0.01$)。同时发现淋巴结转移组中 PTEN 表达明显低于无淋巴结转移组($P<0.01$),这与 Ettl 等[14]在涎腺癌中的研究结果一致。推测 PTEN 蛋白对肿瘤细胞的迁移有抑制作用,PTEN 可能是通过增强和稳定肿瘤细胞间黏附作用而抑制肿瘤细胞转移。Kim[15]用 PTEN siRNA 作用于恶性黑色素瘤细胞株后,细胞中 RAS 基因被激活引起 E-cadherin 降低,导致肿瘤细胞间连接及稳定性的破坏而发生浸润及转移。作者前期研究实验结果也显示在食管癌组织中 PTEN 与 E-cadherin 的表达呈正相关,这提示 PTEN 可能通过某种机制调节细胞 E-cadherin 的表达及细胞间的黏附力。Lau 等[16]在卵巢癌组织中发现 PTEN 与 E-cadherin 呈密切正相关,且 PTEN 通过 PI3K/AKT 途径调节 E-cadherin 的表达。也有学者报道,胃癌组织中 PTEN 表达与 CD147 表达呈负相关,PTEN 表达的降低及 CD147 的增加促进胃癌细胞的淋巴结转移[1]。但本研究中发现食管癌中 PTEN 表达与 CD147 表达无明显相关。

肿瘤细胞中抑癌基因 PTEN 的丧失导致肿瘤细胞的增殖,E-cadherin 的减少导致肿瘤细胞间的黏附能力下降以及 CD147 在肿瘤微环境中诱导基质蛋白酶的表达所导致的细胞外基质的溶解,是恶性肿瘤发生浸润及转移的必要因素。三者在肿瘤的发生、浸润及转移的不同阶段发挥不同的作用,而且又密切相关。所以联合检测 3 种指标,可作为正确判断食管癌患者预后,指导临床治疗的辅助分子生物学指标。但是关于三者在食管癌的发生、发展、浸润及转移中的具体机制还需进一步研究。

参考文献

[1] 许建民,董涛,田湘萍,等. PTEN 和 AKT2 及 CD147 在胃癌组织中的表达及意义[J]. 中国现代普通外科学进展,2010,13(9):681-684.

[2] Wang LL,Yao GY,Zhang BY,et al. Expression and significance of CD147 and E-cadherin in human gastric carcinoma[J]. Zhong Hua Zhong Liu Za Zhi,2009,31(7):515-519.

[3] 万勇,吴心愿. 死亡相关蛋白激酶 1 和 CD147 在食管鳞癌中的表达及其临床意义[J]. 中华肿瘤杂志,2012,34(1):44-48.

[4] 熊莉娜,熊枝繁,涂艳. CD147、EGFR 在食管鳞状细胞癌组织中的表达及意义[J]. 中国现代医学杂志,2011,21(20):2388-2391.

[5] 刘海明,窦俊峰. CD147 和 MMP-2 在早期食管癌中的表达[J]. 中外医疗,2010,29(29):3,5.

[6] 魏文启,赵国强. 沉默食管癌 EC9706 细胞 CD147 表达的研究[J]. 中国医学检验杂志,2010,11(2):78-80.

[7] 许良中,杨文涛. 免疫组织化学反应结果的判定标准[J]. 中国癌症杂志,1996,6(4):229.

[8] Sweeny L,Dean NR,Frederick JW,et al. CD147 expression in advanced cutaneous squamous cell carcinoma[J]. J Cutan Pathol,2012,39(6):603-609.

[9] Liu F,Cui L,Zhang Y,et al. Expression of HAb18G is associated with tumor progression and prognosis of breast carcinoma[J]. Breast Cancer Res Treat,2010,124(3):677-688.

[10] Zhu S,Li Y,Mi L,et al. Clinical impact of HAb18G/CD147 expression in esophageal squamous cell carcinoma[J]. Dig Dis Sci,2011,56(12):3569-3576.

[11] Pan Y,He B,Song G,et al. CD147 silencing via RNA interference reduces tumor cell invasion,metastasis and increases chemosensitivity in pancreatic cancer cells[J]. Oncol Rep,2012,27(6):2003-2009.

[12] Saad AA,Awed NM,Abd Elkerim NN,et al. Prognostic significance of E-cadherin expression and peripheral blood micrometastasis in gastric carcinoma patients[J]. Ann Surg Oncol,2010,17(11):3059-3067.

[13] Nakamura K,Kodama J,Hongo A,et al. Role of emmprin in endometrial cancer[J]. BMC Cancer,2012,12(1):191.

[14] Ettl T,Baader K,Stiegler C,et al. Loss of PTEN is associated with elevated EGFR and HER2 expression and worse prognosis in salivary gland cancer[J]. Br J Cancer,2012,106(4):719-723.

[15] Kim M. Cooperative interactions of PTEN deficiency and RAS activation in melanoma metastasis[J]. Small Gtpases,2010,1(3):161-168.

[16] Lau MT,Klausen C,Leung PC,et al. E-cadherin inhibits tumor cell growth by suppressing PI3K/Akt signaling via β-catenin-Egr1-mediated PTEN expression[J]. Oncogene,2011,30(24):2753-2758.

(收稿日期:2012-06-26 修回日期:2012-11-07)
