# Supplementary material for: Expression of Concern: The prognostic and clinicopathologic characteristics of CD147 and esophagus cancer: A meta-analysis
Source: PLoS One. 2023 Feb 22;18(2):e0282229. doi: 10.1371/journal.pone.0282229 (PMC9946197; doi:10.1371/journal.pone.0282229)
Supplement: S1 File — (ZIP) [file pone.0282229.s001.zip › PDF of included paper/CD147íóMMP-2║═ VEGF╘┌╩│╣▄┴█╫┤╧╕░√░⌐╓╨╡─▒φ┤∩╝░╥Γ╥σ.pdf]

医学新知杂志 2009 年第 19 卷第 1 期

CD147、MMP-2 和 VEGF 在食管鳞状细胞癌中的表达及意义

马光　高志安　张玉华

【摘要】目的　探讨 CD147、MMP-2 和 VEGF 在食管鳞癌中表达的相关性及临床病理意义。**方法**　应用免疫组化 S-P 法检测 70 例食管鳞癌组织中 CD147、MMP-2 和 VEGF 的表达。**结果**　70 例食管鳞癌中，CD147、MMP-2 和 VEGF 的阳性率分别为 77.14%、61.43% 和 57.14%；高、中分化鳞癌组 MMP-2 和 VEGF 的表达高于低分化鳞癌组，差异有显著性（$P<0.05$）；浸润肌层组、浸润浆膜层组 CD147、MMP-2 和 VEGF 的表达显著高于浸润黏膜下层组，差异均有显著性（$P<0.05$）；有淋巴结转移组 3 种蛋白的表达显著高于无淋巴结转移组；CD147、MMP-2 和 VEGF 的异常表达在食管鳞状细胞癌的进展中具有协同作用。**结论**　CD147、MMP-2 和 VEGF 在食管鳞癌组织中呈高表达，其异常表达可能与食管鳞癌的恶性演进有关。

【关键词】食管肿瘤；CD147；MMP-2；VEGF；免疫组化

【中图分类号】R735.1　**【文献标识码】**A　**【文章编号】**1004-5511(2009)01-0012-04

Significance of Expressions of CD147, MMP-2 and VEGF in Esophageal Squamous Cell Carcinoma

MA Guang[1], GAO Zhian[2], ZHANG Yuhua[1]

1 Cangzhou Medical College, Cangzhou, Hebei 061001

2 Liaoning Medical College, Liaoning, Jinzhou 121001

【Abstract】Objective　To investigate the expressions of CD147, MMP-2 and VEGF in esophageal squamous cell carcinoma, and to research clinically pathological significance and correlation between them. **Methods**　The expressions of CD147, MMP-2 and VEGF were detected by immunohistochemical SP method in 70 cases of esophageal squamous cell carcinoma. **Results**　The positive rates of CD147, MMP-2 and VEGF were 77.14%, 61.43%, and 57.14% respectively; the positive rates of MMP-2 and VEGF in the group of well-differentiated and middle-differentiated carcinoma were higher than in the group of poor-differentiated carcinoma; higher positive rates of CD147, MMP-2 and VEGF were obtained in the group of muscular layer and chorion infiltration than in the group of submucosa infiltration; the positive rates of CD147, MMP-2 and VEGF in the group with lymph node metastasis were significantly higher than in the group without lymph node Metastasis; the abnormal expressions of CD147, MMP-2 and VEGF were in close correlation in esophageal squamous cell carcinoma. **Conclusion**　CD147, MMP-2 and VEGF were over-expressed in esophageal squamous cell carcinoma, which can be relevant to the malignant progression of esophageal squamous cell carcinoma.

【Keywords】esophageal carcinoma; CD147; MMP-2; VEGF; immunohistochemistry

食管癌是消化道最常见的恶性肿瘤之一，我国的食管癌发病率居世界首位，病死率占全部恶性肿瘤病死率的 23.5%，多数患者就诊时已属晚期，其浸润和转移是造成食管癌患者死亡的主要原因。在肿瘤发生、发展各个环节都伴随着多种肿瘤浸润和转移相关基因表达异常，这些肿瘤转移相关基因

作者单位：061001 河北沧州，沧州医学高等专科学校病理教研室（马光、张玉华）；121001 辽宁锦州，辽宁医学院（高志安）

通信作者：马光，E-mail：maguang791125@163.com

表达和/或基因功能异常可能是细胞恶变过程中获得浸润和转移能力的重要原因[1]。我们应用免疫组化的方法,检测 CD147、MMP-2、VEGF 在 70 例食管鳞状细胞癌中的表达,探讨其在食管鳞癌中的表达及意义,为临床防治食管癌和判断预后提供理论依据。

1 对象和方法

1.1 研究对象与分组 70 例食管鳞癌(食管鳞癌组)、10 例正常食管黏膜(对照组)标本取自 2001 年 1 月 ~2005 年 7 月辽宁医学院附属第一医院病理科的存档蜡块。标本经 10% 的福尔马林固定,石蜡包埋,4 μm 切片,常规 HE 染色。所有病例均按照 WHO 标准[2]确定病理诊断。食管鳞癌组中,男性 48 例,女性 22 例;年龄 40 ~77(平均 59.5)岁;肿物≥5 cm 31 例,肿物 <5 cm 39 例。所有病例术前均未做放疗、化疗和免疫治疗。食管鳞癌组按食管肿瘤分化程度分高、中、低分化鳞癌组,按浸润程度分浸润黏膜下层组、浸润肌层组、浸润浆膜层组,按有无转移分有淋巴结转移组、无淋巴结转移组。对照组中,男性 6 例,女性 4 例;年龄 42 ~76(平均 58)岁。

1.2 材料与方法 免疫组化染色采用 S-P 法。兔抗人多克隆抗体 CD147,鼠抗人单克隆抗体 MMP-2、VEGF 购自北京中杉生物技术有限公司;试剂盒为美国 ZYMED 公司产品。严格按说明书操作,DAB 显色。以已知 CD147,MMP-2 蛋白表达阳性的乳腺导管癌和 VEGF 蛋白表达阳性的结肠癌作为阳性对照,以 PBS 代替一抗为阴性对照。

1.3 结果判定标准 CD147 蛋白以细胞膜或细胞质呈棕黄色为阳性,MMP-2、VEGF 蛋白以细胞浆呈棕黄色为阳性。参照 Warnberg 等[3]判断标准,结合肿瘤细胞阳性染色所占百分比及染色强度确定本试验结果的判断标准。

(1)阳性细胞数(A 值)的判断:在 400 倍普通光镜下每张切片随机计数 10 个视野,每个视野计数 100 个肿瘤细胞,计算阳性细胞百分率。0 分,无阳性细胞或阳性细胞数≤25%;1 分,阳性细胞数 26% ~50%;2 分,阳性细胞数 51% ~75%;3 分,阳性细胞数 >75%。

(2)染色强度(B 值)的判断:0 分,无色;1 分,淡黄色;2 分,棕黄色;3 分,棕褐色。

(3)染色阳性细胞数与染色强度的和,即 A + B >3 分者为免疫组化反应阳性。

1.4 统计学处理 采用 SPSS 11.5 统计软件。计数资料应用 χ^2 检验、Fisher 精确概率法;等级相关检验用 Spearman 相关检验。

2 结果

2.1 CD147、MMP-2 和 VEGF 表达的检测结果 CD147、MMP-2 和 VEGF 在观察组中的阳性表达率分别为 77.14% (54/70)、61.43% (43/70)、57.14%(40/70),在对照组中的阳性表达率分别为 0% (0/10.00)、0% (0/10.00)、20% (2/10.00),经 Fisher 确切检验分析,3 种蛋白在两组间的表达有显著性差异($P<0.05$)。见表 1,图 1 ~3(见封 4)。

CD147 在高、中、低分化鳞癌组中的阳性表达率分别为 76.92%、78.57%、75.00%,两两比较均无显著性差异($P>0.05$)。MMP-2 和 VEGF 的表达在高、中分化鳞癌组高于低分化鳞癌组,差异有显著性($P<0.05$);但二者的表达在高分化鳞癌组和中分化鳞癌组,差异无显著性($P>0.05$)。

CD147、MMP-2 和 VEGF 的表达在浸润肌层组、浸润浆膜层组显著高于浸润黏膜下层组,差异均有显著性($P<0.05$);但三种蛋白在浸润肌层组与浸润浆膜层组的表达,差异无显著性($P>0.05$)。三种蛋白的表达在有淋巴结转移组显著高于无淋巴结转移组,差异有显著性($P<0.05$)。见表 2。

2.2 CD147、MMP-2 和 VEGF 在食管鳞癌中表达的相关性 Spearman 相关检验显示观察组中 CD147、MMP-2 和 VEGF 表达两两比较均呈显著性正相关。($r_1=0.268$, $r_2=0.265$, $r_3=0.330$, $P<0.05$)。见表 3 ~5。

3 讨论

肿瘤的浸润转移是一个多步骤的复杂过程,多种基因及其产物参与这一过程的调控。肿瘤细胞从原发灶脱落后,必需突破细胞外基质和基底膜组

成的屏障结构，才能向周围组织浸润，进而进入血液循环系统或淋巴系统，形成远隔器官转移或淋巴结转移。

表 1　CD147、MMP-2 和 VEGF 在正常食管黏膜和食管鳞癌中的表达情况/n

组别	例数	CD147			MMP-2			VEGF		
		+	−	χ^2	+	−	χ^2	+	−	χ^2
食管鳞癌组	70	54	16	23.736	43	27	13.282	40	30	4.841
对照组	10	0	10		0	10		2	8	

表 2　食管鳞癌组中不同临床病理变化 CD147、MMP-2 和 VEGF 表达/n

病理变化	例数	CD147			MMP-2			VEGF		
		+	−	χ^2	+	−	χ^2	+	−	χ^2
组织学分化程度										
高分化鳞癌	26	20	6	0.021	18	8	0.031	17	9	0.007
中分化鳞癌	28	22	6	0.020	20	8	4.072	18	10	4.627
低分化鳞癌	16	12	4	0.074	6	10	4.849	5	11	4.454
浸润深度										
黏膜下层	11	3	8	11.628	3	8	5.072	2	9	7.609
肌层	30	25	5	15.567	20	10	5.387	20	10	6.144
浆膜层	29	26	3	0.935	20	9	0.036	18	11	0.136
淋巴结转移										
无	36	24	12	4.613	18	18	4.086	26	10	6.882
有	34	30	4		25	9		14	20	

表 3　CD147 与 MMP-2 表达的相关性/n

CD147	MMP-2		合计
	+	−	
+	37	17	54
−	6	10	16
合计	43	27	70

$r_1 = 0.268, P < 0.05$

表 4　CD147 与 VEGF 表达的相关性/n

CD147	VEGF		合计
	+	−	
+	27	27	54
−	13	3	16
合计	40	30	70

$r_2 = 0.265, P < 0.05$

表 5　MMP-2 与 VEGF 表达的相关性/n

MMP-2	VEGF		合计
	+	−	
+	19	24	43
−	21	6	27
合计	40	30	70

$r_3 = 0.330, P < 0.05$

由于 MMPs 能降解细胞外基质中的多种蛋白成分，因而在肿瘤细胞突破细胞外基质和基底膜的屏障过程中起重要作用[4]。MMP-2 是 MMPs 家族的重要成员，是降解基底膜成分 – IV 型胶原纤

维的主要酶,在肿瘤的浸润、转移中亦起重要作用[5]。本研究结果显示,食管鳞癌组织中 MMP-2 的阳性表达率为 61.43%,明显高于正常食管黏膜。关于 MMP-2 的研究报道各家结果不一,阳性率从 25.6% 到 80.5%。MMP-2 的表达与食管鳞癌临床病理关系的研究显示:高、中分化鳞癌组 MMP-2 的表达高于低分化鳞癌组;浸润肌层组、浸润浆膜层组显著高于浸润黏膜下层组;有淋巴结转移组显著高于无淋巴结转移组。此结果表明,MMP-2 的高表达可能使肿瘤细胞获得较强的浸润和转移的能力。

CD147 是一种细胞表面黏附分子,介导细胞间的黏附,其高表达有助于癌细胞的浸润、转移[6]。本研究显示,食管鳞癌组织中 CD147 的表达明显高于正常食管黏膜组;浸润肌层组、浸润浆膜层组显著高于浸润黏膜下层组,有淋巴结转移组显著高于无淋巴结转移组。这提示 CD147 的高表达可能使肿瘤细胞获得侵袭特性,进而有利于转移的发生。另外,肿瘤细胞表达的 CD147 可刺激肿瘤相关的间质成纤维细胞和内皮细胞产生 MMPs,通过 MMP-2 的作用促进肿瘤的浸润、转移[7]。

VEGF 是一种肝素结合糖蛋白,是最主要的血管生成因子,在正常食管的腺细胞、单核巨噬细胞、血管内皮细胞的胞质内有少量 VEGF 的表达[8]。但在食管癌组织中,肿瘤细胞自身可表达 VEGF。VEGF 促进肿瘤血管形成,为肿瘤快速增殖和转移提供条件[9],在恶性肿瘤的生长及转移中起重要作用。本研究结果显示:VEGF 的表达在浸润肌层组、浸润浆膜层组显著高于浸润黏膜下层组,有淋巴结转移组显著高于无淋巴结转移组。

对 CD147、MMP-2 和 VEGF 的相关性分析提示:三者间有明显相关性。这可能因为 CD147 刺激肿瘤周围纤维母细胞产生 MMP-2,且其本身是 MMP-2 活化的主要因子;通过 MMP-2 的活化降解基底膜和细胞间质,导致肿瘤细胞浸润扩散。而 MMP-2 在降解基底膜和细胞间质过程中,通过释放 VEGF、bFGF、TGFβ 等促血管生成因子,促进新生血管形成,进而有利于肿瘤细胞生长。

上述研究结果表明,CD147、MMP-2 和 VEGF 的过表达在食管鳞癌的发生发展、浸润及转移过程中可能发挥着重要作用。检测这些肿瘤标志物有助于食管鳞癌的生物学行为的预测及预后的评价。

参考文献

1 韩玥,石景森,杨毅军,等. CD44V6 和基质金属蛋白酶 MMP-2 表达与胆囊癌侵袭和转移关系的研究[J]. 中华肝胆外科杂志,2002,8(4):253-254.

2 Watanabe H, Jass J R, Sobin L H. Histological typing of oesophageal and gastric tumours[M], 2nd ed. Berlin: Springer, 1990: 187 - 197.

3 Warnberg H Nordgren, L Bergkvist, Jeon H S, et al. Tumor markers in breast carcinoma correlate with grade rather than with invasivaeness[J]. British Journal of Cancer, 2001, 85 (6): 869-874.

4 Johnson N, Abonen M, Kahari V, et al. Matrix metalloproteinases in tumor invasion[J]. Cell Mol Life Sci, 2000, 57 (1): 5-15.

5 Eric E G, Thanh H X, A lain M, et al. EMMPRIN/CD147 and MMP modulator in cancer development and tissue repair [J]. Biochin Ie, 2005, 87(9): 361-368.

6 Kanekura T, Chen X, Kanezaki T. Basigin (CD147) is expressed on melanoma cells and induces tumor cell invasion by stimulating production of matrix metalloproteinase by fibroblast[J]. Int J Cancer, 2002, 99(4): 520-528.

7 Veikkola T, Karkkainen M, Claesson Welsh L, et al. Regulation of angiogenesis Via Vascular endothelial growth factor receptors[J]. Cancer Res, 2000, 60: 203-212.

8 Riedel F, Gotte K, Schwalb J, et al. Serum levels of matrix metalloproteinase-2 and - 9 in patients with head and neck squamous cell carcinoma [J]. Anticancer Res, 2000, 20: 3045-3049.

9 Kanekurn T, Chen X, Kanznki T. Basigin (CD147) is expressed tonmelanoms cells and induces tumor cell invasion by stimulating production of matrix metalloproteinases by fibroblasts[J]. Int J Cancer, 2002, 99(4): 520-528.

(收稿日期:2008-11-21)
(编辑:陈 捷)

CD147,MMP-2和VEGF在食管鳞状细胞癌中的表达及意义

(正文见第12页)

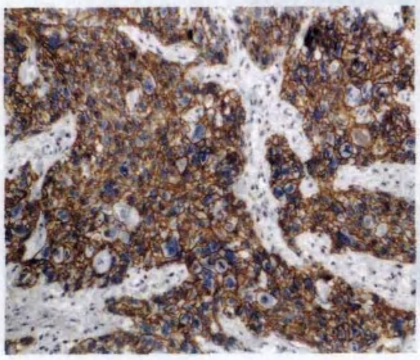

图1 Cd147蛋白在食管鳞癌中的表达定位于细胞膜，呈棕黄色颗粒状（SP法100×）

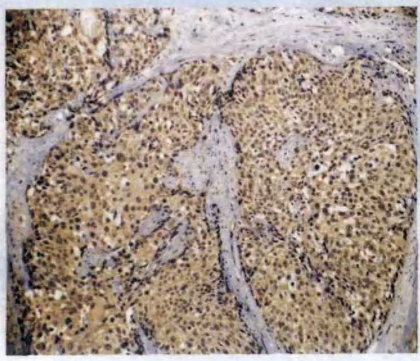

图2 VEGF蛋白在食管鳞癌中的表达定位于细胞浆，呈棕黄色颗粒状（SP法100×）

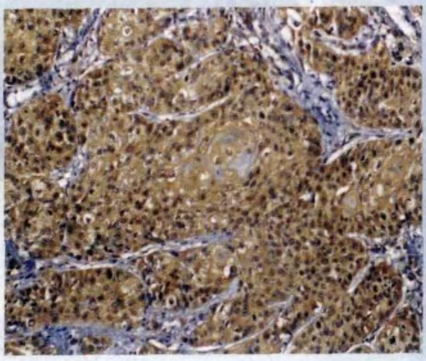

图3 MMP-2蛋白在食管鳞癌中的表达定位于细胞浆，呈棕黄色颗粒状（SP法100×）

98075X
09, 019, 001

B超在急诊软组织异物取出术中的运用

(正文见第26页)

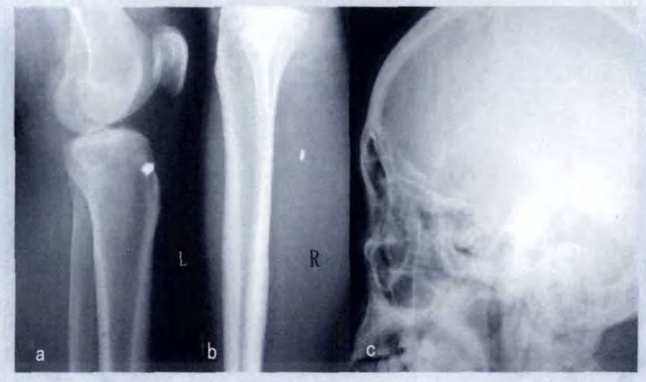

图1 软组织异物X线片：左膝下部（a）、右小腿三头肌内（b）、左眉弓上方（c）各有高密度影异物各1枚

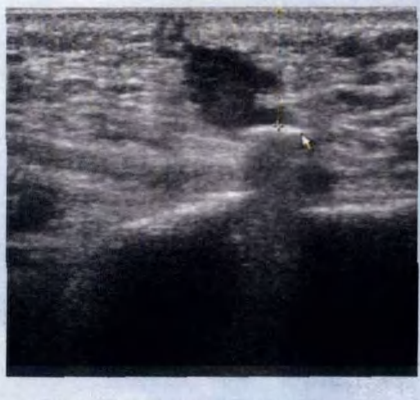

图2 额部异物B超图像：可见异物（箭头所示）伤道及异物下的骨面

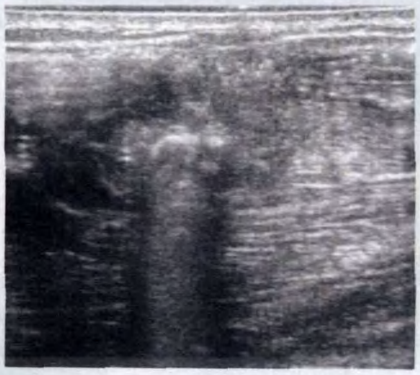

图3 左膝部异物B超图像 可见异物位于皮下

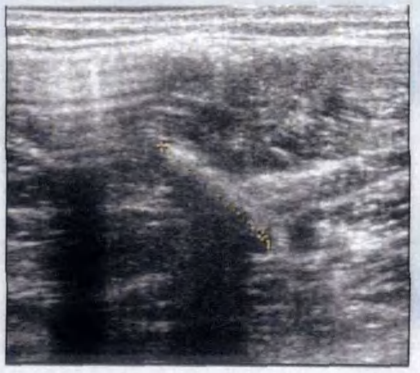

图4 右小腿异物B超图像 可见异物位于肌层内图示血管钳正在夹取异物，箭头所示为血管钳

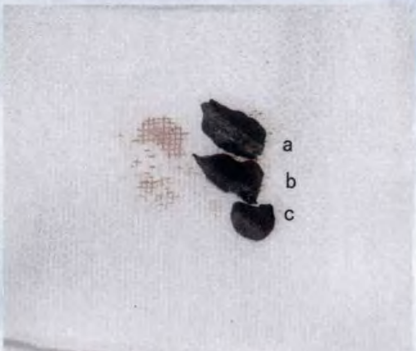

图5 异物外观 左膝下部（a）、右小腿三头肌内（b）、左眉弓上方（c）异物

刊号：ISSN 1004-5511 CN 42-1220/R 国内邮发代号：38-339 国内定价：每册8.00元
