# Supplementary material for: Expression of Concern: The prognostic and clinicopathologic characteristics of CD147 and esophagus cancer: A meta-analysis
Source: PLoS One. 2023 Feb 22;18(2):e0282229. doi: 10.1371/journal.pone.0282229 (PMC9946197; doi:10.1371/journal.pone.0282229)
Supplement: S1 File — (ZIP) [file pone.0282229.s001.zip › PDF of included paper/CD147íóMMP-2╡─▒φ┤∩╙δ╩│╣▄░⌐╔·╬∩╤o╠╪╨╘╣╪╧╡╡─┴┘┤▓╤╨╛┐.pdf]

目　录

中文摘要……1
英文摘要……3
符号说明……6
前　言……7
材料与方法……8
结　果……11
讨　论……16
结　论……30
附　图……31
参考文献……34
综　述……40
致　谢……54
攻读学位期间发表的学术论文……55
原创性声明……56

Y1973304

CD147、MMP-2的表达与食管癌生物学特性关系的临床研究

研究生：肖祥之

专　业：外科学

导　师：蒋仲敏　教授

中文摘要

目的

食管癌是人类常见的侵袭性恶性肿瘤之一，对人类健康的危害日益严重。近些年对肿瘤标志物的研究较多，能否通过简单的标志物来指导食管癌的治疗一直是人们期待的，如p53、CEA、特异性烯醇化酶等，但现在发现大多数经证实其特异性及灵敏度都不高，而细胞外基质金属蛋白酶诱导因子(extracelluir matrix metalloproteinase inducer，CD147/EMMPRIN)能明显调节基质金属蛋白酶-2(matrix metalloproteinase-2，MMP-2)的分泌并在近期多项研究中证实与肿瘤的进展有着密切的联系，但在食管癌方面的报道较少。本研究通过检测CD147和MMP-2在食管鳞癌及正常食管组织中的表达情况，分析二者与食管癌生物学行为的关系，探讨抑制恶性肿瘤侵袭转移的方法，为食管癌的治疗及判断预后提供一种新的指标。

方法

采用过氧化物酶标记的链霉卵白素染色法(即 S-P 法)免疫组织化学(immunohistochetnistry，IHC)检测60例食管鳞癌及正常食管组织中的CD147和MMP-2的表达情况并对食管癌中它们的表达与性别、年龄、大体类型、分化程度、淋巴结转移、肿瘤临床分期等的关系，及两者在食管癌进展中的相互关系进行初步研究。采用 SPSS13.0 统计软件分析，多组有序分类变量采用Wilcoxon和Kruskal-wallis检验，各组之间两两比较采用Wilcoxon检验；按临床不同病理指标分组，各组间率的比较采用Z或$\chi2$检验。CD147与MMP-2相关性分析采用Spearman秩相关。$P<0.05$为有统计学意义。

结果

1.CD147和MMP-2主要在食管肿瘤组织中表达，在正常组织中表达较少或不表达。

2. CD147 在正常食管组织、食管癌组织中的阳性表达率分别为 13.3%和 80.0%，正常食管组与食管癌组之间有显著性差异($\chi2$=7.849，P<0.001)。

3. MMP-2 在正常食管组织、食管癌组织中的阳性表达率分别为 10.0%和 90.0%，两组之间有显著性差异($\chi2$=12.415，P<0.01)。

4. 在食管癌中，CD147、MMP-2 表达阳性率在有淋巴结转移组均高于无淋巴结转移组(P<0.01)；它们在食管癌中的表达与年龄、性别、大体类型、分化程度关系不明显(P>0.05)。

5. 在食管癌中，CD147、MMP-2 表达阳性率在晚期组高于早期组(P<0.01)，它们在食管癌中表达与年龄、性别、大体类型、分化程度关系不明显(P>0.05)。

6. 食管癌组织中 CD147 与 MMP-2 之间呈正相关，相关系数 r_s=0.565。（P<0.001）

结论

CD147 和 MMP-2 在正常食管、食管癌组织中的表达有显著性差异；在食管癌中，CD147 、MMP-2 的表达在有淋巴结转移组的表达高于无淋巴结转移组；晚期组高于早期组；CD147 和 MMP-2 之间有正相关关系；提示 CD147 和 MMP-2 在食管癌的浸润和转移中高表达。联合检测 CD147 和 MMP-2 在食管癌中的表达有可能成为预测食管癌侵袭转移的肿瘤标志物和指导食管癌治疗的新靶标。

关键词：食管鳞癌 CD147 MMP-2 免疫组织化学

THE CLINICAL RESEARCH ON THE RELATIONSHIP BETWEEN THE EXPRESSION OF CD147 AND MMP-2 WITH BIOLOGICAL BEHEAVIOR IN ESOPHGEAL CANCER

Postgraduate : Xiao-XiangZhi

Major : Surgery

Tutor : Prof. Jiang-ZhongMin

ABSTRACT

Objective

Esophageal cancer is one of human common invasive malignant tumors, increasingly serious harm to human health.In recent years the research of tumor markers has been increasing, whether through simple marker tests to diagnose early esophageal cancer has been expected, Such as p53, CEA, specificity ene alcoholize enzyme etc, but its specificity and sensitivity is confirmed not high. extracelluir matrix metalloproteinase inducer, CD147 / EMMPRIN can obviously adjust matrixmetalloproteinase-2 (MMP-2) secretion, and in recent studies the MMP-2 secretion are closely confirm related to the tumor progression, this experiment through testing the expression of CD147, MMP-2 in patients with esophageal squamous cell carcinoma and the esophagus normal organization, but reports less in patients with esophageal cancer. analysis the relationship of them with biological behaviors in the esophageal cancer, discuss effect of the above indexes on esophageal occurrence, development, infiltration, transfer the role that the diagnosis, providing a new effective index for treatment and prognosis for esophageal cancer.

Methods

Using peroxidase marker chains mildew LuanBai staining method (S-P method) immunohistochetnistry, testing the expression of CD147 and MMP-2 of 60 patients with esophageal squamous cell carcinoma and the normal esophagus organization.analysising the relationship between expression of CD147 and MMP-2 with clinical pathology index with sex,age,general type,deffrentition degree,lmphnodes metastasis and clinical stages and histological grade and the

development in esophageal cancer, as the same time preliminarily investigates the relationship between the both and the development process of esophagus cancer. Using statistical software SPSS13.0 analysis,multiple orderly classification variables Kruskal-Wallis and Wilcoxon,using the binary comparison between groups inspection,using Wilcoxon inspection;according to the clinical different pathological index group, using Z or χ^2 test between each group.with CD147 and MMP-2 Spearman rank correlation analysis using relevant. *P*<0.05 with statietical significance.

Results

1. CD147 and MMP- 2 mainly express in the tumor tissue of esophagus but less or not in normal tissue.

2. Expression rate of CD147 in normal esophagus organization and esophageal tissue were respectively 13.3%,80.0%, which was significant difference (χ2=7.849, *P*<0.006).

3. Expression rate of MMP-2 in normal esophagus organization and esophageal tissue were respectively 10.0%,90.0%, which was significant difference (χ2=9.415, *P*<0.001).

4. In esophageal, the positive expression rate of CD147 and MMP-2 in lymph node metastasis was higher than no (*P*<0.01),and has nothing relationship with age、gender、gross type、degree of differentiation (*P*>0.05).

5. In esophageal, the positive expression of CD147 and MMP-2 protein in late was higher than early(*P*<0.01), and has nothing with and age、gender、gross type、degree of differentiation (*P*>0.05).

6. The CD147 and MMP - 2 Esophageal tissues was positively correlated, correlation coefficient r_s=0.565. (*P*<0.001)

Conclusion

There was significant difference expression of CD147 and MMP- 2 betwwen the normal esophageal and esophageal cancer tissue; and it in lymph node metastasis was higher than no; and higher in late than early, there was cooperation betwwen CD147 and MMP- 2;show that CD147 and MMP-2 in patients with esophageal plays an expression high in invasion and metastasis aspects, simultaneous determination of CD147 and MMP-2 might become new tumor mark in predicting invasion and metastasis of esophageal cancer and directing management.

Keywords: esophageal squamous cell cancer ; CD147 ; MMP-2 ; immunohistochemistry.

符号说明

英文缩写	英文名称	中文名称
CD147/EMMPRIN	extracellular matrix metalloproteinaseinducer	细胞外基质金属蛋白酶诱导因子
MMPs	matrix metalloproteinases	基质金属蛋白酶
S-P	streptavidin perosidase	链霉菌抗生物素蛋白-过氧化物酶连结法
MT- MMPs	membrane type matrix metalloproteinase	膜型基质金属蛋白酶
IHC	Immunohistochemistry	免疫组织化学
ECM	extracellular matrix	细胞外基质
MAPK	mitogen-activated protein kinase	促分裂原活化蛋白激酶
MDR	multidrugresistance	多药耐药
VEGF	vescular endothelial growth factor	血管内皮生长因子
ERK	extracellular signaler gulated kinase	细胞外信号调节激酶
TIMPs	Tissue inhibitors of metaloproteinase	组织金属蛋白酶抑制剂

前 言

食管癌是人类常见的以鳞状细胞癌(鳞癌)为主恶性肿瘤之一，而且多数患者就诊已属晚期。我国 2002 年因食管癌死亡人数为 30 万，2005 年则增加到 35 万，每年新增病例超过 25 万，约占世界新增病例的 50%[1]，对人类健康的危害日益严重。以往虽然采用细胞学检查如食管拉网及涂片等但诊断敏感度较低；或其他方法虽然其灵敏度升高但又缺乏特异性。所有这些都说明我们的诊断技术还有待于进一步提高。近年来广泛应用于临床酶学对于恶性肿瘤中随着病情进展不断变化的研究较多如 p53 蛋白、特异性烯醇化酶等，其特异性受患者并发症或合并症影响较大而相关性太差，难以从单个酶的表达升高来判定患病的真实性或可靠性，影响了疾病的判断从而影响治疗和预后。

在恶性肿瘤治疗中肿瘤的浸润及转移一直是恶性肿瘤治疗中一个难以解决的问题，它的作用机制我们了解甚少。我们相信，肿瘤浸润及转移是一个复杂的连续过程，它包含了细胞粘连、蛋白水解及迁移和血管生成等以及它们之间的相互作用。肿瘤侵袭和转移时，肿瘤细胞只有穿越基底膜才能侵入周围组织，因此降解细胞外基质(ECM)成为肿瘤侵袭转移的关键。

细胞外基质金属蛋白酶诱导因子（CD147）能明显调节基质金属蛋白酶(MMPs)的分泌，促进细胞外基质的降解并在多种恶性肿瘤中如乳腺癌、肺癌、宫颈癌等的表达升高已经研究证实与侵袭和转移相关，而在食管癌中的研究报道较少。本研究采用免疫组织化学的方法联合检测 CD147 与 MMP-2 在食管鳞癌及正常食管组织中的表达情况并分析二者与食管癌生物学行为的关系以及二者之间的相互关系，为食管癌的预后和治疗筛选特异性较高的新标志物及发现可能抑制肿瘤侵袭及转移的方法，在临床治疗和学术研究中具有重大意义。

材料和方法

1.1 一般资料与分组

收集山东省千佛山医院2002年2月至2005年10月之间食管癌经手术切除术且术后经病理证实为鳞癌的蜡块，同一病例包括所有肿瘤组织蜡块和远侧缘即切线蜡块，并经病理专家或在其指导下去除切线阳性和肿瘤经常规染色后结果不明确的如液化及坏死的病例，而且根据肿瘤切除原则，切线远离肿瘤组织大于5厘米即可视为正常组织，所以我们将肿瘤组织蜡块作为肿瘤组而将切线蜡块作为正常食管组，这样取得符合条件的标本共计60人份并记录其淋巴结转移个数以利于临床分期。每人份取食管肿瘤60例及食管远侧切线60例分别作为食管癌组和正常食管组来进行比较。60例食管癌按TNM分期(2009年):I期15例，II期25例，III期17例，Ⅳ期3例；手术病理证实有淋巴结转移者21例；病理分化程度:高分化10例，中分化15例，低分化35例。所有60例病人取食管组织标本前均未经放、化疗；切片均经病理专家两遍复核，所采取标本的患者年龄为29岁-84岁，平均年龄50岁，而且年龄无统计学差异。

1.2 实验试剂及其配制

兔抗人CD147多克隆抗体，编号: ZA-0455，购自北京中杉金桥生物技术有限公司，4℃冰箱保存，工作浓度1:50。

兔抗人MMP-2多克隆抗体，编号:ZA-0331，购自北京中杉金桥生物技术有限公司，4℃冰箱保存，工作浓度1:50。

过氧化物酶标记的链霉卵白素染色试剂盒:购自北京中杉金桥生物技术有限公司，

试剂包括:试剂(无色液体): 3%H_2O_2去离子水

试剂A(蓝色液体):封闭用山羊血清工作液

试剂B(黄色液体):生物素化二抗工作液

试剂C(橙色液体):高敏过氧化物酶复合物

磷酸盐缓冲液(PBS):0.01M,PH8.0,购自北京中杉金桥生物技术有限公司。

EDTA缓冲液:0.01M，PH8.0，购自北京中杉金桥生物技术有限公司。

DAB底物试剂盒:购自北京中杉金桥生物技术有限公司。

DAB溶液配制:DAB6mg；0.01M PH7.4的PBS溶液10ml ；3%$H_2O_2$15ul

APES:购自北京中杉金桥生物技术有限公司。

1.3 主要仪器

Leica 石蜡切片机

微量加样器

国产医用微波炉

国产低温电冰箱

国产恒温电烤箱

光学显微镜(日本 olympus)

1.4 实验方法

S-P 免疫组织化学法

准备载玻片：防脱磨砂载玻片用重铬酸钾浓硫酸液浸泡 24 小时，再放入自来水中漂洗干净，以无水酒精脱水，经防脱片处理，室温下晾干 24 小时后备用。

1.5 准备组织切片：

10%中性福尔马林固定，做 3um 厚度石蜡包埋组织连续切片，贴附于前述经处理的防脱磨砂玻片上，放入 67℃-70℃温箱中孵育 2 小时，分别做 HE 染色和免疫组织化学染色。

免疫组织化学(Immunohistochemistry，IHC)染色方法：

(1)67℃-70℃温箱内孵育 20 分钟；

(2)脱蜡:依次浸入二甲苯 I、II、III，各 10 分钟；

(3)水化:无水乙醇、90%乙醇 10 分钟各一次，80%蒸馏水冲洗 10 分钟；

(4)PBS 冲洗 3 次，每次各 3 分钟；

(5)抗原热修复:切片置于盛有 1mM EDTA pH8.0 缓冲液的容器中，中火(94-98℃)4 分钟一次，中低火 8 分钟一次，自然冷却至室温；

(6)PBS 冲洗 3 次，每次各 3 分钟；

(7)3%H2O2 室温孵育 15 分钟，以去除内源性过氧化物酶活性；

(8)PBS 冲洗 3 次，每次各 3 分钟；

(9)滴加正常山羊血清工作液封闭,室温下孵育 15 分钟，不洗；

(11)滴加一抗，4℃过夜；

(12)取出，恢复至室温，PBS 冲洗 3 次，每次各 3 分钟；

(13)滴加适当比例二抗，室温下孵育 15 分钟；

(14) PBS 冲洗 3 次，每次各 3 分钟；

(15)滴加辣根酶标记链霉卵白素工作液，置室温下孵育 15 分钟；

(16) PBS 冲洗 3 次，每次各 3 分钟；

(17)DAB 显色:DAB 显色液滴于切片上，显微镜下控制 5 分钟-15 分钟；

(18)充分水洗、苏木素复染、梯度酒精脱水、二甲苯透明、树胶封片；

1.6 免疫组化结果的评定方法

1. 以 PBS 代替一抗为阴性对照，以生物公司提供的乳腺癌阳性切片为阳性对照。

2. 免疫组化阳性细胞根据所占的比例和染色强度分别评分为 0 分-3 分。

3 染色强度评分:未着色或和背景相同为 0 分，浅着色为 1 分，中度着色为 2 分，深着色为 3 分。

4. 阳性细胞百分数：随机在高倍镜(×400)下取 10 个视野，每视野计数 10 个细胞。以平均值作为阳性细胞百分数。阳性细胞数<5%评 0 分，5%-25%评 1 分，26%-50%评 2 分，>50%评 3 分。

5. 将两项指标的积分数分成 4 级：阴性(-)为 0 分，弱阳性(+)为 1 分-2 分，阳性(++)为 3 分-4 分，强阳性(+++)为 5 分-6 分。

1.7 统计学分析

以 SPSS13.0 软件包对数据进行统计学处理，多组有序分类变量采用 Wilcoxon 和 Kruskal-wallis 检验，各组之间两两比较采用 Wilcoxon 检验；按临床不同病理指标分组，各组间率的比较采用 Z 或 $\chi 2$ 检验。CD147 与 MMP-2 相关性分析采用 Spearman 秩相关。检验水准为 α =0.05。

结 果

2.1 食管癌癌变过程中 CD147 和 MMP-2 的组织学表达

2.1.1 食管癌组织学 HE 染色结果

食管鳞癌组织分布呈巢状，由多少不等的纤维组织分隔，癌细胞呈多角形，细胞边界清晰，细胞核多呈圆形或椭圆形，位于细胞的中央，染色深。由于分化程度的不同鳞癌又可分为高、中和低分化。高分化的鳞癌中，细胞呈现明显角化，胞质丰富，细胞异形性不明显，核分裂象不多；而低分化鳞癌中，细胞未见角化现象，癌细胞多呈梭形或长椭圆形，细胞异形性明显，核分裂象较多；中分化鳞癌组织的形态介于高低分化之间。

2.1.2 食管癌患者 CD147 的免疫组织化学染色结果

CD147 主要于细胞膜上表达，细胞膜和(或)细胞质见棕黄色颗粒者为阳性。正常食管组(附图 1):CD147 不表达或弱表达；食管癌组(图 2，3):以癌细胞的细胞膜和(或)细胞质呈棕黄色为 CD147 阳性表达，与阳性对照细胞膜上出现较强的弥漫染色一致，部分病例细胞膜有不连续或较弱的表达，在癌旁正常组织或间质细胞中 CD147 则表达较弱或不表达。

2.1.3 正常食管组 CD147 阳性表达率为 13.33%;在 60 例食管癌组中 CD147 (+++)30 例，(++)15 例，(+) 3 例，(-)12 例，CD147 阳性表达率为 80%；两组间差异（见表 1）有显著性(Z=7.849，P<0.001)。

表 1 CD147 在正常食管及食管癌中的表达

病变类型	例数	CD147 表达				阳性率(%)
		−	+	++	+++	
正常食管组	60	52	6	2	0	13.33
食管癌组	60	12	3	15	30	80.00

注：Z=7.849，P<0.001

2.1.4 食管癌中 CD147 的表达和临床病理学指标的关系

本实验选取性别、年龄、大体类型、分化程度、淋巴结转移、临床分期等为研究对象，基本涵盖了恶性肿瘤的侵袭与转移生物学特性。发现发病年龄组中平均年龄 50 岁，说明该年龄为食管癌好发年龄，所以在设置对比时以 50 岁为分段标准；在入组的病例中，高分化组或中分化组病例数明显偏少，所以将其二组合并为一组与低分化组进行比较。CD147 在无淋巴结转移组和有淋巴结

转移的表达区别（图 2，图 3）。由表 2 我们可以看出，CD147 阳性表达率在无淋巴结转移组低于有淋巴结转移组(Z=2.765，P=0.006)；在Ⅱb 期-Ⅳ期组高于Ⅰ期-Ⅱa 期组(Z= 2.457，P=0.014)。食管癌中 CD147 的表达与年龄、性别、大体类型、分化程度均无明显相关性(P>0.5)。目前多数文献报道性别不能影响食管癌的预后与本研究结果一致，少数报道认为女性患者在围手术期比男性具有免疫优势，食管癌的女性患者好于男性患者。

表 2　食管癌患者 CD147 的组织学表达与临床病理指标的关系

临床病理指标	例数	CD147 表达				阳性率(%)	Z 或 χ^2	P
		−	+	++	+++			
年龄(岁)								
≤50	33	7	1	9	16	78.79	0.193	0.847
>50	27	5	2	6	14	81.48		
性别								
男	24	6	2	4	12	75.00	0.442	0.659
女	36	6	1	11	18	83.33		
大体类型								
蕈伞	14	2	0	4	8	85.71	1.206	0.752
髓质	19	3	1	6	9	84.21		
缩窄	18	5	2	3	8	72.22		
溃疡	9	2	0	2	5	77.77		
临床分期								
Ⅰ-Ⅱa	32	10	2	8	12	68.75	2.457	0.014
Ⅱb-Ⅳ	28	2	1	7	18	92.85		
分化程度								
高中分化	25	5	0	9	11	80.00	0.366	0.715
低分化	35	7	3	6	19	80.00		
淋巴结转移								
有	31	3	2	5	21	90.32	2.765	0.006
无	29	9	1	10	9	68.97		

2.2.1 食管癌患者 MMP-2 的免疫组织化学染色结果

MMP-2 主要于细胞浆中表达，细胞浆内着棕色或棕黄色颗粒的为阳性细胞。正常食管组(图 4):上皮细胞、间质细胞和基底膜细胞中几乎未见 MMP-2 阳性表达。食管癌组(图 5，图 6):阳性细胞呈巢样或散片状分布，表达较强的多在癌巢边缘，特别是在呈多角状低分化的癌组织中，间质组织或血管临界前沿表达更加突出；食管鳞癌间质细胞中阳性表达的细胞数显著高于癌细胞。

2.2.2 由表 3 可以看出正常食管组 MMP-2 阳性表达率为 10%；60 例食管癌中 CD147(+++)29 例，(++)16 例，(+)9 例，(-)6 例，MMP-2 阳性率表达更是高达 90.00%。两组间差异有显著性（Z=9.415，P<0.01）。

表 3 MMP-2 在正常食管及食管癌中的表达

病变类型	例数	MMP-2 表达				阳性率(%)
		−	+	++	+++	
正常食管组	60	54	5	1	0	10.00
食管癌组	60	6	9	16	29	90.00

注：Z=12.415，P<0.01

2.2.3 食管癌 MMP-2 的表达与临床病理学指标的关系

由表 4 可以看出，MMP-2 蛋白表达阳性率在临床分期 I 期-IIa 期低于IIb 期-IV 期(Z=3.292，P=0.001)；无淋巴结转移组低于有淋巴结转移组(Z=3.111，P=0.002)；食管癌中 MMP-2 的表达与年龄、性别、大体类型、分化程度均无明显相关性(P>0.05)，提示 MMP-2 在癌转移中起重要作用。在不同分化程度的食管鳞癌中，MMP-2 的表达无明显差异，此研究结果与少数文献报道相悖，可能由于高分化和中分化合为一组造成的偏差。而造成分化高经常发生转移的原因和关联病理因素相互作用的内在分子机制目前国内外仍没有明确阐述。

表 4　食管癌患者 MMP-2 的组织学表达与临床病理指标的关系

临床病理指标	例数	MMP-2 表达				阳性率(%)	Z 或 χ^2	P
		−	+	++	+++			
年龄(岁)								
≤50	33	3	5	9	16	90.91	0.246	0.806
>50	27	3	4	7	13	88.89		
性别								
男	24	2	3	8	11	91.67	0.032	0.974
女	36	4	6	8	18	88.89		
大体类型								
蕈伞	14	1	2	2	9	92.86	1.419	0.701
髓质	19	2	3	5	9	89.47		
缩窄	18	2	3	5	8	88.89		
溃疡	9	1	1	4	3	88.89		
临床分期								
Ⅰ～Ⅱa	32	6	7	9	10	81.25	3.292	0.001
Ⅱb～Ⅳ	28	0	2	7	19	100.00		
分化程度								
高中分化	25	2	4	5	14	92.00	0.807	0.420
低分化	35	4	5	11	15	88.57		
淋巴结转移								
有	31	0	3	8	20	100.00	3.111	0.002
无	29	6	6	8	9	79.31		

2.2.4 食管癌组织中 CD147 与 MMP-2 蛋白表达的相关性分析(见表 5)

对 60 例食管癌组织 CD147 与 MMP-2 的关系应用 Spearman 秩相关进行相关性分析和统计学处理，CD147 与 MMP-2 的相关系数 rs=0.565(P <0.001)，提示食管癌组织中 CD147 及 MMP-2 的表达成正相关。

表 5　CD147 和 MMP-2 在食管癌中的表达关系

CD147	MMP-2				合计
	−	+	++	+++	
−	4	3	3	2	12
+	1	1	1	0	3
++	1	3	6	5	15
+++	0	2	6	22	30
合计	6	9	16	29	60

注：r_s=0.565，P<0.001

讨 论

食管癌是常见的消化道癌肿，全世界每年约35万人死于食管癌，其中80%出现在发展中国家，而且以食管鳞状细胞癌为主，在我国发病率男性及女性多于100人/10万/年,低于胃癌,占各部位癌死亡的第二位，严重威胁广大人民群众的健康和生命。食管癌的医学特性是进展迅速、预后较差，它的发生和进展是一个复杂的、多因素参与的漫长的病理过程，癌基因及抑癌基因相应的活化和失活是细胞癌变的分子基础，其致癌机制和易感因素还有待于研究。现在的统计数字表明，即使曾接受放化疗的术后患者，其中位生存期1年-2年而5年生存率低于30%，只有约1%的I期病人接受诊治,而他们经过手术切除术后5年生存率却可以接近90%[2]，大多数患者就诊时已属晚期而失去了有效治疗的时机。复发和转移成为食管癌患者死亡的主要原因，因此降低食管癌的死亡率需要研究发现早期癌变或早期癌的新方法以便在早期就进行药物或者手术等干预。在食管癌的多种生物学特征中，侵袭与转移成为影响病人预后的关键因素，也是恶性肿瘤的重要标志。许多肿瘤标记物都与其侵袭及转移有关，但在肿瘤的诊断和治疗中，真正具有特异性的还有待研究。

食管癌侵袭和转移主要途径是直接蔓延及淋巴结转移，而血行转移则发生较晚。在肿瘤进展过程中肿瘤细胞和周围浸润的巨噬细胞分泌的降解ECM的酶是其侵袭性生长过程中的关键环节。此时需要生成肿瘤的血管，供给肿瘤的侵袭性生长需要的血液。如果能早期发现，并控制其侵袭和淋巴结转移，对于食管癌的治疗就将具有非常重大的意义。因此，筛选特异性肿瘤标记物，对肿瘤的诊断及治疗至关重要。早期诊断以及诊断后的综合治疗越来越引起人们的重视，也是食管癌治疗的趋势，人们已经开始尝试从基因水平进行诊断和治疗。研究CD147及MMP-2在食管癌侵袭及转移的表达，寻找特异性肿瘤标记物，探索阻止恶性肿瘤侵袭和转移的有效方法成为治愈恶性肿瘤的方向。

肿瘤细胞要降解ECM就需要水解酶类，而相关的蛋白水解酶可分为四种：MMPs、丝氨酸蛋白酶、弹力蛋白酶和半胱氨酸蛋白酶。MMPs即基质素又称基质金属蛋白酶，是现在发现的和肿瘤浸润及转移关系最密切的一种锌离子依赖性蛋白水解酶[3]，它可以在适宜的条件下降解大多数ECM成分以及其他底物，比如细胞表面受体、生长因子及其受体，以及趋化因子和粘附分子等，参与了多种正常的生物学过程以及疾病的病理过程，并依靠不同的水解机制发挥

作用。肿瘤组织中的MMPs主要存在于ECM或肿瘤细胞的表面，肿瘤细胞和基底膜表面存在的整合素受体以及各种非整合素受体结合并附着于基底膜，并诱导宿主细胞分泌或直接分泌MMPs，降解局部的基底膜和基质，进一步使肿瘤细胞沿着溶解的基质部位向邻近组织侵袭。在基底膜结构中，IV型胶原是最重要的构成部分，MMP-2和恶性肿瘤侵袭及转移之间的相互关系近年来研究较多，王瑞年[4]等发现，MMP-2在胃癌细胞中的表达异质性增强，且有浸润性生长及淋巴结转移者更为明显，但在食管癌表达的研究较少。

1. CD147的结构与功能

CD147分子即细胞外基质金属蛋白酶诱导因子曾有多种名称，是广泛表达于人体多种组织且相对分子量为50-60kD的一种单次跨膜糖蛋白，人类白细胞分化抗原协作组第六届会议将各实验室的不同名称统一命名为CD147，分属内皮细胞组[5]。过去一直把CD147视为肿瘤侵袭力的一种标志物，近来有学者提出把CD147作为肿瘤治疗中新的靶点。最早在1982年就由Biswas[6]发现，是广泛表达于人体多种类型组织细胞的一种高度糖基化的细胞表面跨膜糖蛋白，尤其是在肿瘤细胞中高表达，属于免疫球蛋白超家族成员，主要功能是参与细胞和细胞之间或细胞和基质之间的黏附，其基因定位于19p13.3，mRNA的长度约117kb，编码区能编码248个氨基酸残基，包含185个氨基酸残基构成细胞外的2个N端C2型免疫球蛋白区、24个氨基酸残基构成的跨膜功能区以及C端39个氨基酸残基构成的细胞内区，其中的细胞外免疫球蛋白区具有激活MMPs以及与小窝蛋白-1结合的活性，另外还包含三个Asn糖基化位点，两个位点位于免疫球蛋白区近细胞膜端，而另一个位于免疫球蛋白区的细胞膜远端。CD147含有的超过其一半分子量的N连接寡糖是其发挥作用所必需的。有研究证实CD147的N端高度糖基化，不同的组织其糖基化程度也不同，这是由组织特异性决定的。CD147糖基化程度又决定了其激活MMPs的能力，去糖基化的CD147经纯化即丧失了诱导MMPs的分泌的能力[7]。CD147结构上有一个亮氨酸拉链结构是其显著特点，使它具备和靶DNA相互作用来调控基因的表达能力。

肿瘤细胞产生的CD147有可溶型及膜型两种存在形式，它不但参与机体的炎症反应，还在多种肿瘤细胞中的表达也升高，通过介导肿瘤细胞和间质细胞间的作用来刺激肿瘤细胞及间质细胞的成纤维细胞分泌MMPs，使得细胞间质

及基底膜成分的降解，食管的屏障作用消失，肿瘤细胞得以向邻近的周围组织侵袭，在肿瘤浸润和转移中发挥作用。Ellis[8]等最早证实 CD147 在肺癌细胞系 LX21 的表面表达，它可以刺激人的成纤维细胞分泌 MMPs 而有利于癌细胞的浸润和扩散，CD147 从而成了在肿瘤进展方面的一个重要因子。Mayr D[9]等证实,CD147 在正常组织中仅极低表达或不表达，即便表达也主要在角化细胞表达且水平较低，但在肿瘤组织中却高表达,并可促进 MMPs 的分泌,亦和肿瘤的生物学行为及临床进展相关。而且在机体不同系统中存在的 CD147 可参与多种不同的生理过程，如胚胎的发育和伤口愈合等。Chen[10]等研究发现，人胚胎的皮肤中没有 CD147 的表达，随着胚胎的发育可在 20 周时基底细胞中找到少量表达的 CD147,在皮肤附属器干细胞及毛基质细胞却有大量 CD147 的表达；等到了幼儿和成人时 CD147 主要表达在表皮基底层、毛囊外根鞘细胞及毛基质细胞,而且随细胞的分化 CD147 表达逐渐减少。这充分证明在角质形成细胞的过程中，CD147 的表达和细胞的分化状态有密切关系，这就说明细胞分化异质性程度越高它的表达越高，与本研究结果相悖，但目前多数文献证实分化程度与其无关；它本身又是一种潜在的细胞粘附分子(CAM)，与细胞之间以及细胞与细胞外基质之间相互作用有关，主要参与细胞之间的识别并改变胶原的溶解平衡点促使 MMP 激活；还能通过调节 5-脂加氧酶及膦脂酶 A1 的活性来调控 MMP 的表达[11]，主要包括介导肿瘤细胞之间以及它和基质细胞之间的相互作用,形成肿瘤细胞-MMPs-基质成分复合物，局部构成了肿瘤侵袭及转移的微环境。通过羧基段溶解产生的微泡形式的 CD147 更使其刺激 MMPs 表达的能力从肿瘤附近的间质细胞扩展到远处的间质细胞,从而使得肿瘤的远处转移更容易。它还可以和 MMP 形成复合物,从而使其紧密黏附在肿瘤细胞的表面并促进肿瘤细胞间质的降解,使得肿瘤周围的微环境更适合其向周围浸润和远处转移。这也许就是 CD147 促进 MMPs 产生的作用机制。Zucker[12]等发现作为 MMP 的刺激物，CD147 具有刺激肿瘤细胞及其周围间质的成纤维细胞分泌基质金属蛋白酶的能力，促进肿瘤细胞的转移。在研究 CD147 和肺癌[13]、乳腺癌[14]、宫颈癌[15]、皮肤鳞状细胞癌[16]等多种肿瘤的关系时发现 CD147 在肿瘤细胞中均表达增高。Zucker[12]还证实患乳腺癌的裸鼠经转染 cDNACD147 后，肿瘤的生长速度加快并且转移增加，这和同期升高的 MMP-2 水平有关，这些都证实它能够刺激人成纤维细胞分泌和表达 MMPs，甚至增强了其表达能

力，从肿瘤附近的表达扩大到远处的间质细胞的表达，更利于癌细胞的浸润和扩散。

CD147 刺激 MMPs 产生的的信号途径研究不多，不同的肿瘤中可能有不同的作用机制。CD147 能通过抑制细胞内 Ca^{2+}的信号调控通路而诱导肝癌细胞保持高表达的稳定性和 MMPs 的活化。Sidhu[17]等也证实大部分 CD147 都以膜型出现，只有大约 2%-3%通过脱落变成可溶的微泡形式，而即便这种形式，同样可以诱导成纤维细胞分泌 MMPs。微泡可以作为载体发挥作用，这种途径需要在蛋白激酶 C、钙离子流动和促分裂原活化蛋白激酶(mitogen-activated protein kinase，MAPK)等相继作用下完成。CD147 是通过 Ca^{2+}依赖性信号途径来促进肿瘤浸润、转移的。Ca^{2+}是几条信号途径的同一调节点，它与肿瘤细胞的浸润及转移有关。Jiang[18]等用 CD147 干扰 NO/cGMP 介导的钙离子内流时，发现了钙离子及肝癌细胞转移潜能的关系，证实肝癌细胞的转移能力是由细胞内钙离子的稳定性决定的；它们在调节钙离子稳态中可能起着反作用，尤其在钙内流储存方面。研究证实 Ca^{2+}依赖性信号途径成为抑制恶性肿瘤生物学行为新的靶点。

有学者认为，侵袭和转移过程中包含细胞外基质及基底膜的多次降解，而 CD147 在促进肿瘤进展方面扮演了一个重要的角色。CD147 在肿瘤细胞浸润和转移促进作用表现为：存在于肿瘤细胞表面的 CD147 能够诱导肿瘤周围间质的成纤维细胞分泌 MMP-1、MMP-2、MMP-3 和 MMP-9 以及 MMP-11，与肿瘤细胞相关的 CD147 刺激 MMPs 表达的同时，肿瘤基质中增加的 MMPs 特别是 MMP-2 又促进细胞膜上的 CD147 降解，产生的水溶性 CD147 增加，而增加的水溶性 CD147 又作用于邻近及远处的肿瘤基质细胞，以这样级联反应的方式产生了更多 CD147 和 MMP-2，从而使肿瘤细胞得以进入循环发生转移。

近来的研究还发现 CD147 可以诱导血管内皮细胞生长因子（VEGF）及透明质酸的表达，而后两者与血管生成及多药耐药 (multidrugresistance，MDR) 关系密切。Jia[19]等证实在小鼠肝癌细胞系 HePal-6 中 CD147 能明显调节血管内皮细胞生长因子的表达、化学敏感性和肿瘤源性。CD147 的高表达，不但可激活基质金属蛋白酶-2 降解细胞外基质，还能上调血管内皮细胞生长因子的表达，从而促进肿瘤的侵袭和转移。CD147 通过刺激肿瘤细胞本身或其间质成纤维细胞产生的 MMP-2 在新血管生成的位置浓聚降解细胞外基质，破坏血管内

皮；而在血管内皮细胞生长因子参与下促进了血管管状结构建立，这也说明了CD147 可能与血管内皮生长因子间接促进了血管内皮的增生并形成肿瘤组织赖以生存的新的微血管，供应肿瘤组织养和营养。过去一直认为自然耐药及肿瘤细胞转移是相对独立的两个过程，最近有研究指出两者之间存在着某种联系，其中介物质可能就是 CD147。Yang[20]等在研究多药耐药肿瘤细胞系 MCF-7/Adr、KBV-1、A2780DX5 中 CD147 的表达时发现，其表达明显高于敏感的肿瘤细胞，并且和 MMP-2、CD147 的表达具有一致性；还显示在多药耐药细胞中的 MMP-2 数量及活性能被一种 MAPK/Erk 的抑制剂所抑制，可能是由于抑制了 CD147 对 MMP-2 的诱导，这表明 CD147 可能在肿瘤细胞耐药性诱导中起作用，多药耐药肿瘤细胞的基因转录和 CD147 刺激基质金属蛋白酶的分泌可能是通过同一途径来调节的。

此外，CD147 还能与整合素家族中的 α3β1、α6β1 形成蛋白复合体，也能和 α4β1、α4β7 相关[21,22]但作用机制不明。整合素家族的黏附分子主要作用是介导细胞和细胞外间质的黏附以及促进细胞的移动，其信号转导的最显著特征是具有双向性，它的主要配体是细胞外基质，和整合素相关的主要信号分子是 FAK，它在细胞骨架变化和细胞增殖及存活的调控中都起着重要的作用；CD147 和整合素 β1 及 CD98 可形成传感复合物，能调节微环境中的细胞外基质金属蛋白酶以及氨基酸和羧酸水平等[23]。

2 CD147 在食管癌中的研究

有关 CD147 在食管癌中表达的文献报道不多，马光[24]等在研究食管癌时发现，随着癌组织浸润深度的增加以及淋巴结的转移，CD147 的表达相应增高，提示 CD147 的表达可能与肿瘤的转移有关。熊莉娜[25]等在研究食管癌时发现，CD147 的表达在早期与中晚期食管癌的阳性表达率分别为 33.3%和 90.3%，在有转移组和无转移组的表达率分别为 87.9%和 28.6%，差异均有统计学意义，也说明 CD147 的表达与食管癌的浸润和转移有关，与本研究基本一致。Caudroy[26]等指出，CD147 可以刺激 MMP-2 的分泌，而 MMP-2 可以降解细胞外基质并促进肿瘤细胞的侵袭和转移，以 CD147 基因转染的 MDA-MB-436 肿瘤细胞具有更强的侵袭能力，而且发现 CD147 分子不调节 MMP-9 及 MT-MMP 的表达，对 MMP 的抑制物 TIMP 的表达也没有影响。

3 MMP-2 的结构与功能

基质金属蛋白酶是一类具有 Zn^{2+}依赖性的肽链内切酶，它是一种Ⅳ型胶原酶，其分子量为 72KD，结构包括 N 端前肽区、催化区、铰链区与 C 端类血红素结合蛋白区。膜型基质金属蛋白酶还包括 C 端跨膜区，在 N 端前肽区内有一保守序列与 MMP 酶原激活有密切关系。在距离酶活性催化功能区 Zn^{2+}位点外有另一个 Zn^{2+}位点，和至少一个 Ca^{2+}结合区，在两个 Zn^{2+}结合区中，一个为催化性 Zn^{2+}，另一个为结构性 Zn^{2+}，前者就与 MMP 活化有关。铰链区与类红素结合蛋白区与大多数 MMP 底物特异性有关，跨膜区将 MT-MMP 固定于细胞膜。按作用底物不同主要分为五大类:间质胶原酶、明胶酶又称Ⅳ型胶原酶、基质溶酶、膜型基质金属蛋白酶和其他类。MMPs 产生于正常组织细胞(包括结缔组织细胞、内皮细胞、胸腺细胞、巨噬细胞和淋巴细胞等)和肿瘤细胞，以酶原形式分泌，进入细胞外间隙激活后才能降解 ECM。MMPs 具有高度的同源性，它们的一级结构中均含有两个高度保守区，体内激活机制为纤溶酶原的放大机制，其活性受金属蛋白酶组织抑制剂(TIMPs)的调节。二者在 ECM 代谢中的相互作用是肿瘤细胞侵袭和转移的关键因素，研究 MMPs 在不同肿瘤中的表达和分泌水平对肿瘤的防治具有重要意义。MMPs 作为蛋白水解酶，至今发现有 24 种，MMP-2 只是其中的一种又称明胶酶，不仅在成纤维细胞表达，而且在肿瘤周围的血管内皮中也有表达，可降解包括基底膜主要成分 IV 型胶原酶在内的多种基质成分，是肿瘤细胞侵袭和转移的限速酶。MMPs 表达升高多见于许多正常的生理过程(如组织发生、伤口愈合)，还和动脉粥样硬化、结缔组织疾病[27]以及多种肿瘤[28-30]等的发生有关，而且在肿瘤的发病及侵袭转移中起到了重要作用。MMPs 依据不同的细胞类型而表现为多种不同的功能，其作用发挥有赖于不同的水解机制。正常情况下表达水平很低，一旦发生组织重建，如炎症、创伤愈合或发生癌症时，MMPs 便快速转录表达，被激活及分泌，在体内广泛表达。

肿瘤细胞转移的基本方式为：首先肿瘤细胞脱落，然后随着脱落的瘤细胞黏附并降解 ECM，从而进入血管或淋巴管并随之移动，到达继发部位，外渗进而增殖形成转移肿瘤。不管肿瘤细胞还是间质细胞，MMPs 在早期的表达，都有利于 ECM 的结构重建及 ECM 和/或膜结合生长因子的释放，这样就为原发肿瘤的形成提供一个良好的局部微环境。对于肿瘤来说，酶在其进展中扮演了一个重要的角色，包括血管再生、局部侵入、肿瘤细胞浸润和外侵以及发生

转移等。当然，它的转录和分泌同样受各种各样的因子的诱导，在多数情况下它以非活跃蛋白酶的形式隐匿，除非其他蛋白来激活或诱导，否则其不能自动活化。实验证实 MMP 的表达受基因、酶原及活化后调节三个水平的调控：

第一，基因水平的调节。许多因素都可能影响到 MMP 的表达，如激素、肿瘤基因及生长因子和细胞因子等。MMP-2 活化的信号传导机制仍有待进一步研究。研究证实 MAPK 传导途径和 MMP-2 的表达相关。Kurata[31]等研究表明 v-src 转化细胞能转化活性形式的 MAPK，从而在很大程度上增加了 MMP-2 的分泌及活化；而 PD98059(一种 MAPK 特异性抑制物)却能使 MMP-2 的分泌和活化受到明显抑制；wortmannin(一种 P13 激酶抑制物)也并未影响 MMP-2 的分泌和活化。P53 作为一个抑癌基因功能明确，主要是参与细胞凋亡信号调控的过程，近来的研究证实这一蛋白同时又是一个重要的转录因子，还参与多种基因的调控，这其中包括 MMP-2 基因[32]。超过半数人类癌症显示 P53 蛋白突变或缺乏，有些实验证实 P53 功能的缺失也和 MMPs 的过度表达关系密切。已有的研究表明，MMP-2 确是 P53 作用的靶点，它的启动区有 P53 的特异结合位点，P53 能激活 MMP-2 的启动子，促使 MMP-2 表达升高。Dvergugina[33]等证实整合素 α5β3 也能与 MTI-MMP 协同作用，促进其酶原快速转化为活性基质金属蛋白酶-2，作用于某些肿瘤恶变及浸润转移过程中。此外，转化生长因子(TGF)在恶性肿瘤中和高水平表达的 MMP-2 也有一定的内在关联[34]。随着肿瘤的快速增殖，可能由于瘤组织缺乏血氧供应，肿瘤的反馈机制启动，促使血管生成。在肿瘤新生血管的形成中，多种细胞因子以自分泌或旁分泌的形式参与此过程并相互作用，形成内皮细胞毛细血管，如转化生长因子等，均可间接激活 MMP，也可以直接诱导 MMP 基因的转录[35]。

第二，酶原的活化调节。有研究指出，MMP-2 以酶原的形式分泌，而且可以和 MT-MMP 及 TIMP 形成复合物从而活化。酶原的活化的过程中 MMP 发挥着重要的作用。目前，酶原活化的过程有逐步激活、细胞表面激活以及细胞内激活等 3 个不同的机制。其一，酶原的逐步激活：明胶酶和胶原酶以及间质溶素等要以酶原形式(proMMP)分泌进入 ECM，必须先水解去除前肽区才能被活化而发挥作用。研究发现，前肽区保守序列中 Cys 的 SH 基和活性中心催化性 Zn^{2+}离子的结合成为酶原维持稳定的重要环节，酶原逐步激活关键是通过多种方式分离 Zn^{2+}-Cys 的连接，才能使水分子和 Zn^{2+}离子得以相互反应[36]。

所以，Zn^{2+}-Cys 连接断裂才是 MMP 前体激活的关键；但陈超伍[37]等研究发现微量元素 Zn 在食管癌中的含量与 MMP-2 呈负相关,提示 Zn 的含量可能下调了 MMP-2 的蛋白表达但机制有待进一步研究。其二，细胞表面激活：膜型基质金属蛋白酶（MT-MMP）是通过其 c-末端的跨膜区定位在细胞膜上的。许多研究发现，MT1-MMP、MT2-MMP 以及 MT3-MMP 等都可以使 MMP-2 酶原转化成活性 MMP-2。Strongin[38]等在伴刀豆球蛋白处理过的 HT-1080 细胞膜上分离出一种化合物，它能与 proMMP-2 的 C-末端区互相作用，经研究为 MT1-MMP-TIMP2 复合物，它能进一步和细胞膜上的受体相结合形成三聚体，从而激活 proMMP-2；其三，细胞内激活：MT-MMP 和 proMMP-2 可以在细胞内活化，然后再以活性形式表达或分泌，这一过程也需要 Ca^{2+}等的参与。Munshi[39]等发现,肿瘤细胞经体外培养，加入 Ca^{2+}后可促进肿瘤细胞分泌 MMPs,继而促进肿瘤细胞的转移 Yue[40]等研究表明,Ca^{2+}拮抗剂 Mg^{2+}与肿瘤细胞在体外培养,可明显抑制 MMPs 尤其是 MMP-2 的表达及活化,提出 Mg^{2+}可能阻断了 Ca^{2+}信号传导通路，终止了酶的活化过程,起到抑制 MMPs 的表达与活化的作用,从而影响了肿瘤细胞侵袭和转移。

第三，活化后调节，即特异性抑制因子的抑制作用。MMPs 与金属蛋白酶组织抑制物(TIMPs)在细胞外基质的动态平衡中起重要作用,癌细胞与基底膜黏附后,释放或激活蛋白水解酶降解基底膜和细胞外基质,然后定向穿过缺损区,对恶性肿瘤侵袭转移及间质血管生成起重要作用[41]。尽管 MMPs 存在于人体多种细胞中，为避免组织过度损伤，通常其转录水平很低，这就需要 MMP 轴来高度调节。主要是通过天然的 MMP 抑制因子—组织金属蛋白酶抑制剂(TIMPs)来进行调节。作为 MMP-2 天然抑制因子，TIMP-2 具有下调 MMP-2 的活性的能力，为维持 ECM 的稳态起到了重要作用。所以，只有 MMP-2 与 TIMP-2 保持平衡，才能保证 ECM 内环境和完整性。在肿瘤进展时，必然存在某种因素，使 MMP 过度表达或者表达程度超过了 TIMP 的表达时，这种平衡就被打破，从而导致基质降解，利于肿瘤的浸润、转移；反之，TIMP 过度表达或者表达程度超过 MMP 时，就可防止基质降解，阻碍了肿瘤浸润转移[42]。而 α-巨球蛋白作为 MMPs 的血浆抑制剂，也可抑制 MMPs 活性。此外，细胞因子和炎症因子以旁分泌或者自分泌的方式也有一定的调节 MMPs 和 TIMPs 的作用：对明胶酶起上调节作用的有以下几种：TNF-α、IL-1β 和血小板衍化

生长因子-AB 以及 IL-6 和 IL-la；而下调的有 IL-4、IL-10 和 γ 干扰素。上调胶原酶是 TNF-α、IL-α、IL-1β、PDOF、内皮生长因子以及神经生长因子等；而下调的有 TGF-β、TNF-γ、IL-4 等。

MMP-2 在其他肿瘤中研究较多。Etoh T[43]等研究胃肿瘤组织临床病理和 MMP-2 表达的关系时发现 MMP-2 的表达和肿瘤的大小相关，可能是因为随着瘤体增大养和营养的供应相对较差,通过自身调高基质金属蛋白酶表达,降解细胞外基质，以利于肿瘤细胞向血供丰富的远处侵袭，而 MMP-2 正是降解细胞外基质的重要的功能基因,其表达自然会随着瘤体的增大而调高，本研究未涉及肿瘤大小，因为我们认为肿瘤大小并没有实际意义，有可能因为肿瘤细胞生长缓慢但病程较长所造成的，其根本还是肿瘤的临床分期起决定作用。国内外研究均发现，MMPs 在正常肺组织中则表现为低表达或不表达，而在肺癌组织表达明显升高。同时其表达和肺癌的侵袭转移和预后呈正相关[44]。这说明肺癌侵袭转移能力的增强，同其诱导并产生的能破坏细胞外基质完整性的水解酶的能力有关。王新允[45]等以 S-P 免疫组织化学技术检测 MMP-1、MMP-2、MMP-9 和 MMP-13、TIMP-1 及 TIMP-2 等在 104 例肺癌组织中的表达时，发现肺癌组织中阳性表达率均比正常组织明显升高，其中 MMP-2 表达与肺癌分级关系密切。有研究发现肺癌患者血清 MMP-2 水平和血清 TIMP-2 水平并未相应升高，产生了比例失衡，其原因可能是因为 TIPM-2 对 MMP-2 的反应：癌变时患者血清的 MMP-2 升高，同时机体可反馈调节调高 TIMP-2 的水平，但未能起到抑制 MMP-2 的作用，从而导致比例失调，或者某些癌细胞虽未丧失分泌 TIMP-2 的能力，却由于变异或基因突变等原因使 TIMP-2 的活性下降，尽管调高了表达，却并没有赋予其抑制 MMP-2 的能力导致肿瘤的发生和发展。

4 MMP-2 在食管癌中的研究

少数研究认为[46]单一的 MMP-2 或/和 MMP-9 或 MMP-7 可能与胃癌、结肠癌、原发性肝癌、膀胱癌等的侵袭和转移有关,但 MMPs 与食管癌侵袭和转移的关系较小。但多数研究认为 MMP-2 的过度表达与转移性肿瘤细胞的局部浸润和扩散之间关系密切[47],研究发现,食管癌中晚期的 MMP-2 表达量明显高于早期,有淋巴结转移或远处转移者明显高于无转移的食管癌患者,从而认为 MMPs 在食管癌侵袭和转移过程中发挥着重要的作用,还与食管癌的预后密切相关。食管的细胞外基质（ECM）作为防止食管癌侵袭和转移的重要天然屏障，

它的主要成分是胶原蛋白、糖蛋白及蛋白多糖和弹性蛋白等，其中的胶原蛋白约占食管成分的10%-20%并分布于食管结缔组织网状结构的多个部位。食管结构中主要有 6 种胶原[48]，它们的分子结构、基因编码、生理功能和分布位置各有不同，Ⅰ、Ⅱ、Ⅲ型及Ⅴ型和Ⅵ型胶原都是由人成纤维细胞分泌的，而Ⅳ型胶原分布在血管和食管间质组织主要是基底膜和间质中，由三条α肽链螺旋盘绕而成的条索状分子，多个该分子互相桥接形成的一种规则交叉的网状结构，形成基底膜的基本骨架，大多数蛋白水解酶无法降解这种特异性结构而维持了基底膜的完整性和稳定性，从而防止食管癌的侵袭和转移。在 MMPs 中人们普遍认同 MMP-2 是降解Ⅳ型胶原关键酶，而Ⅳ型胶原正是分布子食管间质中的基底膜骨架的主要成分。食管癌细胞及其周围成纤维细胞分泌的 MMP-2 通过已存在或新形成的结合位点或在粘附分子的作用下，与肿瘤细胞和细胞外基质结合，水解基底膜的网状结构骨架，破坏了基底膜的完整性并出现了破坏缺损区域,再经细胞外基质的缺损区向外转移。有研究认为它在中性 pH 环境中，可降解结缔组织并分解细胞间基质和基底膜的所有大分子蛋白，所以它的活性和肿瘤的侵袭和转移关系密切[49]，是肿瘤细胞向周围侵袭的生化基础。MMP-2 可作为预测肿瘤侵袭转移潜能的指标。

李淳[50]等研究食管癌时发现,有淋巴结转移的食管癌中，MMP-2 阳性表达率为 80.0%。MMP-2 阳性表达病例中,阳性表达细胞为癌细胞,间质细胞呈阴性,仅 5 例可见癌细胞及间质细胞中均有 MMP-2 表达。Ⅰ级癌组织阳性细胞多分布在癌巢边缘,Ⅱ-Ⅲ级癌组织阳性细胞散在分布,且无规律性。它的表达和癌组织的浸润深度有关，浸润越深，其阳性表达率越高。MMP-9、TIMP-1、TIMP-2 表达和癌组织浸润深度无关。该研究结果表明它在食管癌的浸润中起重要作用。

关于肿瘤细胞和 ECM 间的相互作用对肿瘤侵袭和转移的影响抑制都是肿瘤生物学研究的热点，研究涉及各个专业，基质金属蛋白酶在肿瘤中的作用越来越受到人们的关注，目前已有的研究其表达和活性的常用技术方法有免疫组化、酶联免疫吸附（ELISA）、原位杂交、酶谱分析法等。Koyama[51]等用原位杂交法检测 30 例食管鳞癌标本中明胶酶的活性,发现 MMP-9 的活性与癌细胞侵袭血管有关，MMP-2 的活性与癌细胞侵袭淋巴管、血管及淋巴结转移均有关系。Suzuki[52]等用明胶酶谱分析法、酶联免疫吸附试验及体外侵袭测定等方

法测定食管癌细胞系(TE 系)在细胞培养液中 MMP-2 和 MMP-9 的含量，发现前者与食管癌细胞体外侵袭程度有关，而后者则无关。陈超伍[37]等在研究中发现，食管癌 MMP-2 的表达率为 69.3%，远远高出在癌旁组织阳性表达率的 12.5%； MMP-2 在淋巴结转移组的阳性率也显著高于无转移组的阳性率，也提示 MMP-2 可能在食管癌的进展与肿瘤转移中发挥作用。

近来的研究还表明，MMP-2 还能与其他因子协同作用。食管癌的侵袭性生长方式需要血液来供应其营养和氧，这有赖于新生血管在肿瘤内部的形成，在形成血管的过程中基底膜的局部降解是血管形成的开始，活化的血管内皮细胞快速增殖并分泌 MMP-2 ，其通过以上多种方式调节自身的表达的作用降解血管基底膜，形成血管出芽部位，激活的血管内皮生长因子使血管芽植入基质中并形成新的血管。孙羿[53]等发现 VEGF、CD44v6 和 MMP-2 三者可能在促进血管生成以及与肿瘤的发生、发展、浸润、转移等恶性生物学行为相关性明显并发挥重要作用。

5 应用进展

CD147 主要刺激肿瘤周围的纤维母细胞产生 MMP,其本身是 MMP-2 活化的主要因子，经过活化后即成为活性 MMP-2，可以降解基底膜及细胞间质，而达到肿瘤细胞浸润扩散的目的；而 MMP 在降解基底膜和细胞间质时，使得内皮细胞移位，有利于 MMP 转移的同时，又释放出血管生成反馈调节中的一些分子如血管内皮细胞生长因子等，促进了新生血管的形成，直接促进了肿瘤细胞的生长[54]。如果能抑制 CD147，就能减少 MMP 的产生，同时阻碍基质的破坏，防止肿瘤细胞的侵袭。实验室中合成 CD147 抑制剂已有报道并被证实有效，它的细节功能怎样，以及如何减少它的副作用等，还有待于进一步研究。MMPs 在许多癌症中的表达升高，以及免疫组织化学的基础研究显示，MMP-2 在食管癌的表达增加，因此它有可能成为食管癌评估潜在辅助分子的重要标志物[55,56]。还有研究表明，血管内皮生长因子（VEGF）同样具有调节基质金属蛋白酶及其抑制剂 TIMPs 的作用[57]。MMP-2 降解毛细血管及淋巴管基底膜，使肿瘤进入循环中，并更多分泌 MMP-2，从而引起远处转移。所以，血液中 MMP-2 水平也应该升高。Hidefumi[58]等测定了肺癌患者血清中的 MMP-2 酶原水平，证实该水平的确比正常组明显升高，但与肿瘤的病理类型无关。Krzysztof[59]等在肾上腺肿瘤的研究中也证实了 MMP-2 血清水平升高，并且和

肿瘤类型无关。肺癌组织表达阳性率和病理类型无关，这就造成肺癌组织阳性表达率在血清与组织的不一致，可能与 MMP-2 分泌的来源细胞不同有关，在肿瘤组织中，主要为肿瘤细胞、间质细胞为主，而血液中则以内皮细胞、肿瘤细胞和巨嗜细胞等分泌为主。此外，也可能和病例数少有关，尚待进一步研究。另外 Gohji[60]等测定部分侵袭性膀胱癌患者血清的 MMP-2/TIMP-2 比值时发现，MMP-2 / TIMP-2 比值高者较低者更早出现复发，这说明 MMPs 及 TIMPs 可做为抗肿瘤治疗的良好标志物的同时，还可以区分复发高危因素的亚型。Davies[61]经过研究也指出，随着 MMPs 与 TIMPs 的比值升高，降解 ECM 的能力越来越强，肿瘤的侵袭能力也相应越来越强。Nuovo[62]等认为，肿瘤浸润和转移时，MMPs / TIMPs 比例失调比单纯的 MMPs 或 TIMPs 表达水平的变化意义更大。目前人们已经开发出安全有效的 MMPs 抑制物 MMPIs，以调节 MMPs / TIMPs 比例。目前已经研制出十余种，如 Taraboletti 等合成的第 1 代广谱抑制剂巴马司他(Batimastat,BB-94)除了能抑制肿瘤生长和侵袭外,还能抑制肿瘤的血管生成。Lee[63]等利用巴马司他(batimastat)对 MDA-MB-231 人乳腺癌细胞株接种形成长骨转移的裸鼠治疗，发现骨转移灶确实明显缩小。马马司他(marimastat)已进入 III 期临床试验。研究表明，对恶性肿瘤侵袭与转移的生物学特性的研究有助于选用更好和更具针对性的治疗方案进行治疗。

6 CD147 和 MMP-2 的研究趋势

CD147 与 MMP-2 多同时表达,可能是由于 CD147 可以促进 MMP-2 的产生。瘤细胞可通过其表面的 CD147 以粘附分子作用在邻近细胞之间以及细胞与细胞外基质之间，改变了胶原的平衡点，促使 MMP-2 活化并通过调节 5-脂加氧酶等进一步调控使其表达升高，形成细胞外基质-MMP-肿瘤细胞复合物，粘附于肿瘤细胞的表面，降解细胞外基质的同时降解 CD147 产生其水溶性形式，又刺激更多 MMP-2 表达，两者均以级联放大反应的方式升高；另一方面食管癌的侵袭性生长方式有赖于新生血管在肿瘤内部的形成，在形成血管的过程中基底膜的局部降解是血管形成的开始，血管基底膜作为 MMP-2 的作用底物其降解是必然的，活化的血管内皮细胞快速增殖并分泌 MMP-2 降解基底膜，形成血管出芽部位，激活的 VEGF 使血管芽植入基质中并形成新的血管有利于肿瘤细胞向邻近组织、血管和淋巴管侵袭，从而为肿瘤转移创造条件。Sun[64]等的研究证实,经过提纯的 CD147 确能诱导纤维母细胞及乳腺癌株 MDA-435 分

泌 MMP-2，抗 CD147 抗体则能抑制 MMP-2 的产生和依赖于 MMP-2 的癌细胞的侵袭能力，而且当这两种蛋白共同表达于食管癌时，明显提高了肿瘤细胞的侵袭和转移能力。这也说明 CD147 与 MMP-2 的表达呈明显正相关，与本研究的结果一致。

7 研究方向

研究表明，对恶性肿瘤侵袭与转移的生物学特性的研究有助于选用更好和更具针对性的治疗方案进行治疗。CD147 与 MMP-2 的表达及其相互关系证明了它们和肿瘤浸润、侵入脉管有关，也提示我们利用 MMP 的抑制剂可能会起到不错的效果，可以取得干扰肿瘤的浸润和转移的效果，但是有研究表明，利用 MMPs 抑制剂作为单一作用剂来治疗肿瘤疗效并不是十分明显[65]而且副作用很大，所以利用 MMPs 抑制剂来控制肿瘤的浸润及转移的方法并不十分理想。但是 CD147 作为一种跨膜糖蛋白在肿瘤细胞中高表达，并且有可溶性分子形式，具有黏附分子的作用；能促进 MMP-2 产生，形成一个介于肿瘤细胞与基底膜之间的微环境,同时 MMP-2 的浓度相对较高，促进了基底膜的降解和肿瘤细胞的移出；参与肿瘤血管的生成和重塑，为肿瘤转移提供新的通道；CD147 还可以通过共同表达调节机制，促进了 MMPs(尤其是 MMP-2)的分泌，影响了 MDRI 基因的转录，促进 p-gP 表达，增强了肿瘤细胞侵袭转移的能力，从而将化疗药物排出细胞,并诱导肿瘤的 MDR。另外，CD147 的糖基化程度和它对 MMPs 的激活能力密切相关，所以调节其糖基化程度也必将影响 MMPs 的分泌，这些都能为 MMPs 的相关疾病包括食管癌的治疗提供新的选择；CD147 抑制剂除了上述作用外，在抑制血管和透明质酸的生成途径方面也有广阔的前景，因此以 CDl47 为靶向分子筛选、设计及合成它的肽类拮抗剂，通过封闭肿瘤细胞膜表面的 CD147 表达,抑制 CD147 就能抑制 MMPs 的活性和分泌及新生血管形成，进而达到抑制肿瘤的浸润和转移的目的，而且有助于防止患者术后肿瘤的转移、复发和提高术后生存率最终延长食管癌患者的生存期。基于控制癌症的需要，研究 CD147 更细节方面的作用机制,包含每一个免疫球蛋白区的功能都是必要的。当然，CD147 的正常功能更加值得关注。虽然 MMP 抑制剂治疗作用不容易接受，但其与相应抑制剂比值的酶学检测却越来越受到研究人员的重视。Gohji[66]等通过研究发现，测定膀胱癌患者血清 MMP-2 与 TIMP-2 的比值，可用来预测尿路上皮肿瘤的复发。由于血清学方法

为定量法测量，能够直接地反映血清中的浓度，标本收集简便，可适用于任何患者；但免疫组化法为一定性试验，只能对病理组织学进行检查，适用于手术或行纤支镜并取活检等有创性检查的患者，有一定的局限性，所以，在条件有限或无法获得病理组织标本时，可以通过外周静脉血清的测定 MMP-2 与 TIMP-2 的比值，了解组织表达的情况，可以更简便地诊断肿瘤和判断病人预后。如果 CD147 或 MMP-2 可以通过血清学联合检测,我们就可以更加简便、高效、准确地判断患者的病情，通过生物芯片等新的实验技术,可能使联合检测变得更加快速、简便和可靠，这将是一次重大进步,还有待于我们去深入地研究和探索。但因其发生血行转移略晚，作为肿瘤标志物仍不十分理想，因此今后的研究任务是积极探究和发现特异性、敏感性俱佳的肿瘤标志物,并应用到临床，极大提高恶性肿瘤的早期诊断水平，。

小结

通过观察 CD147 和 MMP-2 在食管癌及正常食管组织中的表达情况，认为上述两个指标在肿瘤的侵袭与转移的过程中发挥了主要作用，而且它们的表达在食管癌和正常组织中差异明显，作用呈明显正相关关系，所以有望成为临床早期发现食管癌的标志物。食管癌治疗除了外科手术、放化疗等传统治疗以外一些新方法正从实验室走向临床，如免疫治疗和靶向治疗等，CD147 抑制剂在肿瘤领域还将有更为广阔的研究前景。随着对 MMP 抑制剂的认识以及 CD147 刺激 MMP 的分泌和活化机制研究的深入,食管癌的治疗前景将更加广阔。

本实验的局限性：

样本量过小，此结论仍缺少大样本量的数据支持；

选取时间较早的样本均为蜡块组织而非新鲜活体标本，酶的活性可能会下降影响观察和数据统计；

高、中分化组的数目过少只能作为一组与低分化组进行比较，可能掩盖了部分数据的真实性。

结　论

1. CD147 和 MMP-2 在正常食管组织与食管癌组织中的表达相比较，在食管癌组中明显升高，提示 CD147 和 MMP-2 与食管癌的进展密切相关。

2.CD147 和 MMP-2 在有淋巴结转移的食管癌病例组的表达均高于无淋巴结转移组；在 I 期-II 期组均明显低于 III 期-Ⅳ期组，说明它们的表达与淋巴结转移和临床分期有关，可作为判断预后的参数指标。

3.CD147 和 MMP-2 的表达呈正相关，在食管癌的形成和发展中起协同作用。

4.联合检测 CD147 和 MMP-2 有可能成为预测食管癌侵袭及转移的标志物和治疗的新靶点。

附 图

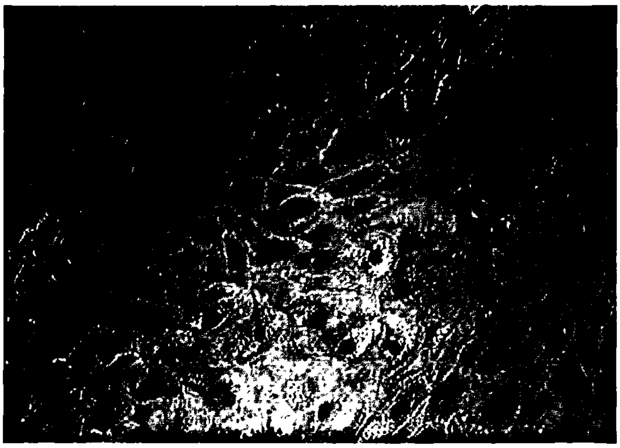

图 1 CD147 在正常组织或中间质细胞和基底膜未见表达或弱表达。免疫组化×100

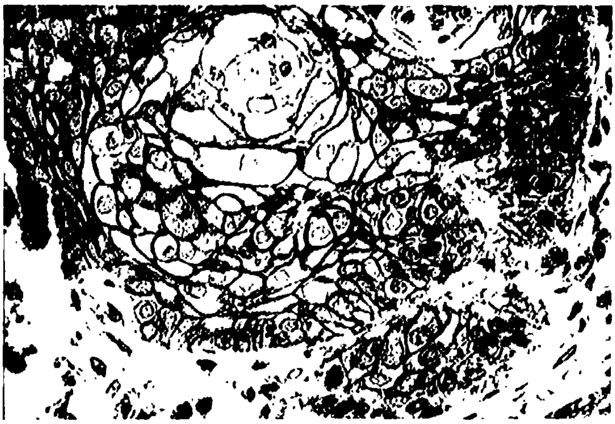

图 2 CD147 阳性表达有淋巴结转移组：细胞大小不等阳性细胞表达在细胞膜呈棕黄色，呈团巢状分布，多在癌巢边缘表达强烈。

免疫组化×400

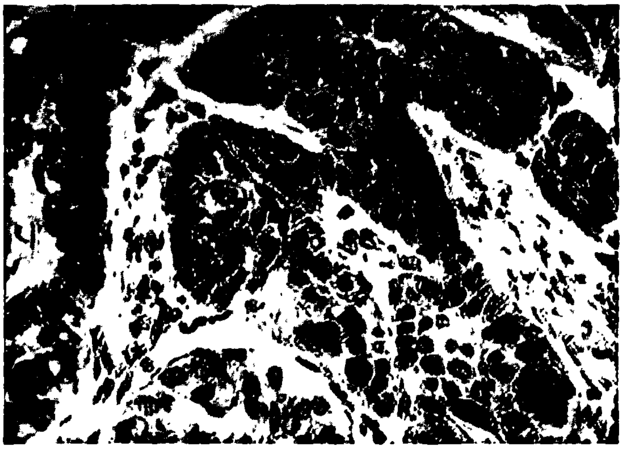

图 3　CD147(+)无淋巴结转移组：细胞大小不等成片状分布，癌巢边缘表达明显。

免疫组化×400

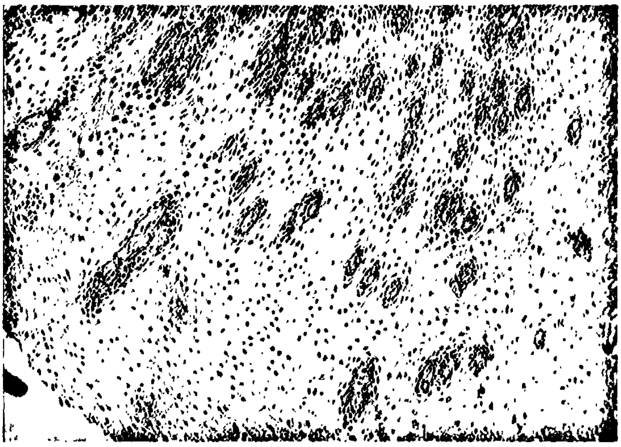

图 4 MMP-2(-)　细胞染色与背景基本一致，几乎未见表达。免疫组化×100

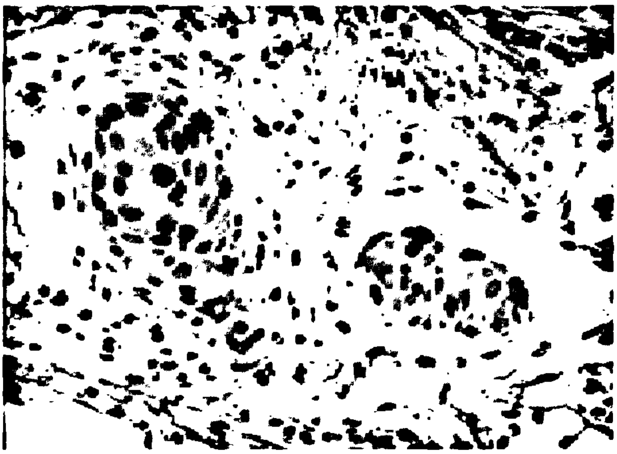

图 5 MMP-2(+)有转移组：细胞大小不等呈巢状分布，间质及细胞浆深棕色，癌巢染色均匀，间质阳性细胞数多。免疫组化×400

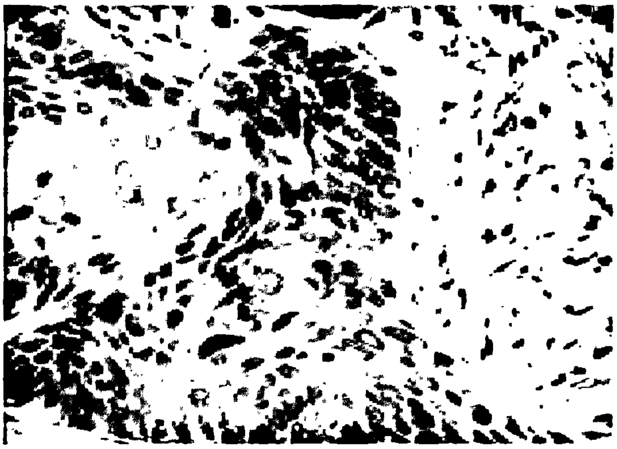

图 6 MMP-2(+)无转移组：阳性表达细胞大小不等呈多角形片状分布，染棕色颗粒，较强的多在癌巢边缘，间质阳性细胞少。免疫组化×400

参考文献

[1]Parkin M D,Bray F,Ferlay J,et al.Global cancer statistics,2002.[J].Cancer J Clin,2005,55:74-108.

[2]Guo H Q,Wei W Q,Lu N,et al.Liquid- based cytology for esophageal carcinoma screening[J].Ai Zheng 2009,28:1243-1247.

[3]Zhao H,Bemardo M M,Osenkowski P,et al.Differential inhibition of membrane type3(MT3)-matrix metalloProteinase(MMP) and MT1-MMP by tissue inhibitor of metalloProteinase(TIMP)-2 and TIMP-3 rgulates Pro-MMP-2 activation[J].J Biol Chem,2004,279(10):8592-8601.

[4]王瑞年,朱延波,薛建元等.胃癌浸润转移与整合蛋白、IV 型胶原酶及细胞外基质的关系[J].中华病理学杂志,1994,13(5):278-283.

[5]金波泉.细胞和分子免疫学[M].北京:科学出版社,2001:4.

[6]Biswas C.Tumor cell stimulation of collagenase production by fibroblasts [J].Biochem Biophys Res Commun,1982,109(3):1026-1034.

[7]Tang Y,Kesavanp, Nakada M T,et al. Tumor-stromainteraetion:positive feedback regulation of extracellular matrix metalloproteinase inducer(EMMPRIN) expression and matrix metalloproteinase-dependent generation of soluble EMMPRIN[J].Mol Cancer Res,2004,2(2):73-80.

[8]Ellis S M，Nabeshima K, Biswas C. Monoclonal antibody prepration and purification of a tumor cell collagenase-stimulatory factor[J].Cancer Res,1989,49 (12):3385-3391.

[9]Mayr D,Kanitz V,Anderegg B,et al.Analysis of gene amplification and prognostic markers in ovarian cancer using comparative genomic hybridization for microarrays and immunohistochemical analysis for tissue microarrays[J]. Am J Clin Pathol, 2006, 126(1):101-109.

[10]Chen X,Kanekura T,Tsuyama S,et al.Ultrastructural localization of basigin in normal human epidermis[J].Histochem Cell Bio J,2001, 115 (6):465-470.

[11]Xu J,Xu H Y,Zhang Q.HAb18G/CD147 Functions in invasion and metastasis of hepato cellu-lar carcinoma[J].Mole Cancer Res,2007,5(6):605-614.

[12]Zucker S,Hylnowitz M, Rollo E E,et al. Tumorigenic potential of extracellular matrix metalloproteinase inducer[J].Am J Pathol,2001,158(6):1921-1928.

[13]张惠忠,王梅,魏益平等.非小细胞肺癌中 EMMPRIN 和 HGF 的表达及其对淋

巴结转移和预后的影响[J].中国病理生理杂志,2007,23(9):1716-1719.
[14]王善伟,陈丽荣.CD147、MMP-9 和 p-ERK 在乳腺癌中的表达及临床意义[J].实用肿瘤杂志,2007,22(2):133-137.
[15]Ju X Z,Yang J M,Zhou X Y,et al.EMMPRIN expression as a prognostic factor in radiotherapy of cervical cancer[J].Clin Cancer Res,2008,14(2):494-501.
[16]陈星静,武海龙.CD147、MMP-2 和 TIMP-2 在皮肤鳞状细胞癌中的表达[J].基础医学与临床,2008,44(11):110-112.
[17]Sidhu S S,Mengistab A T,Tauscher A N,et al.The microvesicle as a vehicle for EMMPRIN in tumor-stromal interaetions[J].Onco gene,2004,23(4):956-963.
[18]Jiang J L,Zhou Q,Yu M K,et al.The involvementofHAb18G /CD147 in regulation of store-operated calcium entry and metastasis of human hepatoma cells[J].J Biol Chem, 2001,276(50):46870-46877.
[19] Jia L, Wang H, Qu S, et al.CD147 regulates vaseular endothelial growth factor-A expression,tumorigenicity,and chemosensitivity to curcumin in hepatocellular carcinoma[J].IUBMB Life,2008,60(l):57-63.
[20]Yang J M,Xu Z,Wu H,et al.Over expression of extracellular matrix metal oproteinase inducer in multidrug resistant cancer cells[J].Mol Cancer Res,2003, l(6): 420-427.
[21]BroodsP C,Stromblad S,Sanders L C et al.Localization of matrix metalloproteinase MMP-2 to the surface of inasive cellsby interaction with integrinαvβ3[J].Cell 1996,85(5):683-693.
[22]Liang L,Major T,Bocan T.Characterization of the promoter of human extracellular matrix metalloproteinase inducer (EMMPRIN) [J]. Gene, 2002, 282 (122):75-86.
[23]Kasinrerk W,Tokrasinwit N,Phunpae P.CD147 monoclonal antibodies induce homotypic cell aggregation of monocytic cell in U937 via LFA-1 /ICAM-1 pathway[J].Immunology,1999,96(2):184-192.
[24]马光,高志安,李春宏等.CD147 和 VEGF 蛋白在食管鳞状细胞癌中的表达及意义[J].锦州医学院学报,2006,27(3):21-23.
[25]熊莉娜,熊枝繁,涂艳. CDl47、NF-κB 在食管鳞状细胞癌组织中的表达及意义[J].中国医师杂志,2010,12(9):1171-1173.
[26]Caudroy S, PoletteN, Nawrocki-RabyB。EMMPRIN-mediated MMP regulation in tumor and endothelial cells [J].Clin Exp Metastasis,2002,19(8):697.

[27]Mengshol JA,Mix KS,Brinckerhoff CE.Matrix metalloproteinase as therapeutic targets in arthritic diseases:bull's eye or missing the mark[J]. Arthritis Rheum,2002,46(1):13-20.

[28]Komosinska-Vassev K,Olczyk P,Winsz-Szczotka k, et al. Age- and gender-dependent changes in connective tissue remodeling: physiologic differences in circulating MMP-3,MMP-10,TIMP-1 and TIMP-2 level[J]. Gerontology,2010,(3):1-9.

[29]Gao Z L, Zhang C,Du G Y,et al.Clinical significance of changes in tumor markers, extracellular matrix,MMP-9 and VEGF in patients with gastric carcinoma[J]. Hepatogastroenterology,2007,54:1591-1595.

[30]Fridman R.Metalloproteinases and cancer[J].Cancer Metastasis Rev, 2006,25: 7-8.

[31]Kurata H,Thant AA,Matsuo S,et a1.Constitutive activation of MAP kinase kinase(MEKl)is critical and sufficient for the activation of MMP-2[J].Exp Cell Res, 2000,254(1):180-188.

[32] 范金兰,王伟萍,吴小华.基质金属蛋白酶释放与活化的研究进展[J].中国癌症杂志,2002,12(3):269-272.

[33] Dergugina EL,Ratnikov B,Monosov E,et a1.MTl-MMP initiates acti- vation of pro-MMP-2 and integrin α5 β3 promotes maturation of MMP-2 in breast carcino- ma cells[J].Exp Cell Res.2001,263(2):209-223.

[34] Velasco P,Lange-Asschenfeldt B.Dermatological aspects of angio- genesis[J]. Br.J.Dermato1,2002,147:841-852.

[35] Krzysztof Kolomecki,Henryk Stepien,Magdalena Bartos,et a1.En-docrine Regulation[M].2001,35:9-46.

[36]Van Wart H E,Birkedal Hansen H.Thecysteine switch:a principle of regulation of metalloprOteinaSe activity with potential applicability to the entire matrix metalloproteinase gene family[J].J Biol Chem,1995,270(10):5331-5338.

[37]陈超伍,马洪升.基质金属蛋白酶在食管癌组织中的表达及与微量元素含量之间的关系[J].世界华人消化杂志,2010,18(19):1995-2000.

[38]Strongin AY,Collier I,Bannikov G,et a1.Mechanism of cell surface activation of 72-kDa type IV collagenase.Isolation of the activated form of the membrane metalloprotease[J].J Biol Chem,1995,270(10):5331-5338.

[39] Munshi HG,Wu YI,Ariztia EV,et al.Calcium regulation of matrix metallo prote- inase-mediated migration in oral squamous cell carcinoma cells[J].J

Biol Chem, 2002,277:41480.

[40] Yue H,Lee JD,Shimizu H,et al.Effect of magnesium on the production of extracellular matrix metalloproteinase incultured rat vascular smooth muscles cells[J]. Athero-sclerosis, 2003,166:271.

[41]Quaranta M,Daniele A,Coviello M,et al.MMP-2,MMP-9,VEGF and CA15.3 in breast cancer[J].Anticancer Res,2007,27(5B):3593-3600.

[42]Grignon D J,Sakr W,Toth M,et al.High levels of tissue inhibitor of metalloproteinase-2 (TIMP-2) expression are associtated with poor outcome in invasive bladder cancer[J].Cancer Res,1996,56(7):1654-1659.

[43]Etoh T,Inoue H,Yoshikawa Y,Barnard GF,Kitano S,Mori M.Increased expression of collagenase-3 (MMP-13)and MT1-MMP in oesophageal cancer is related to cancer aggressiveness[J].Gut 2000,47:50-56.

[44]吕志强,李海刚,谢德荣等.肺痈组织中 MMP-2 表达及与肿瘤血管形成关系[J].中国肿瘤,2004,13(6):378-380.

[45]王新允,杨菊红,马莹,等.肺癌 MMPs 和 TIMPs 的表达及浸润转移的研究[J].中国肺癌杂志,2003,6(4):278-282.

[46]Samantaray S,Sharma R,Chattopadhyaya TK, et al.Increased expression of MMP-2 and MMP-9 in esophageal squamous cell carcinoma[J].J Cancer Res Clin Oncol, 2004,130:37-44.

[47]Tanioka Y,Yoshida T,Yagawa T,et al.Matrix metalloproteinase-7 and matrix metalloproteinase-9 are associated with unfavourable prognosis in superficial oesophageal cancer[J].Br J Cancer,2003,89:2116-2121.

[48]田东波,肖伟民,曾峰等.非小细胞肺癌组织中 MMP-2 及 TIMP-2 的表达与转移、预后相关性研究[J].实用临床医学,2005,6(1):4-11.

[49]Ding Y,Shimada Y,Gorrin-Rivas M J,et al.Clinicopathological significance of human macrophage metalloelastase expression in esophageal squamous cell carcinoma [J].Oncology,2002,63:378-384.

[50]李淳,吴名耀,吴贤英.食管癌 MMP-2、MMP-9 及其组织抑制剂 TIMP-1、TIMP-2 的表达及临床意义[J].实用癌症杂志,2004,19(2):139-141.

[51]Koyama H,Iwata H,Kuwabara Y,et al.Gelatinolytic activity of matrix metaloproteinase-2 and-9 in oesophageal carcinoma,a study using in situ zymography [J].Eur J Cancer,2000,36(16):2164-217.

[52] Suzuki T,Kuwabara Y,IwataH,et al.Role of matrixmetalloproteinase-9 in inv-

itro invasion of esophageal carcinoma cells[J].J Surg Oncol,2002,81(2):80-86.
[53]孙羿,何辉,马强等.膀胱肿瘤抗原利透明质酸等七项肿瘤标志物在膀胱肿瘤诊断中的应用价值[J].中华医学杂志,2005,85(35):2507-2512.
[54]Kanekura T,Chen X,Kanznki T.Basigin (CD147) is expressed tonmelanoma cells and induces tumor cell invasion bystimulating production of matrix metalloproteinases by fibroblasts[J].Int J Cancer,2002,99(4):520-528.
[55]Li Y,Ma J,Guo Q,et al.Overexpression of MMP-2 and MMP-9 in esophageal squamous cell carcinoma[J].Dis Esophagus 2009,22(8):664-7.
[56]Gu Z D,Li J Y,Li M,et al.Matrix metalloproteinases expression correlates with survival in patients with esophageal squamous cell carcinoma[J].Am J Gastroenterol 2005,100:1835-1843.
[57]Ylisirni G S,H6yhty6 M,Turpeermiemi HujanenT.Serum matrix metalloproteinases-2,-9 and tissue inhibitors ofmetalloproteinases-1,-2 in lung cancer TIMP -l as a prognostic marker[J].Anticancer Res,2000,20:1311-1316.
[58] Hidefumi Sasaki, Masanobu Kiriyama, Ichiro Fukai, et a1. Elevated serum pro-MMP2 levels in patients with advanced lung cancer are not suitable as a prognostic marker. Surgery Today[J].2002,32:93-95.
[59] Krzysztof Kolomecki,Henryk Stepien,Magdalena Bartos,et a1.En-docrine Regulation[M].2001,35:9-46.
[60]Gohji K,Fujimoto N,Ohkawa J,et a1.Imbalance between serum matrix metalloproteinase-2 and its inhibitor as a predictor of recurrence ofurothelial cancer[J].Br J Cancer,1998,77:650.
[61]Davies B,Miles DW,Happerfield LC,et a1.Activity of type IV collagenases in benign and malignantbreast disease[J].Br J Cancer,1993,67:1126.
[62]Nuovo G J,Macconnell P B,Simbir A,et a1.Correlation of the in situ detection of polymerase chainreaction-amplified MMPcomplementary DNAs and their inhibitors with prognosis in cervical carcinoma[J].Cancer Res,1995,55:267-275.
[63]Lee J,Weber M, Mejia S,et a1.A matrix metalloproteinase inhibitor batimastat, retards the development of osteolytic bone metastases by MDA-MB-231 human breast cancer cells in Balb C nunu mice[J].Eur J Cancer, 2001,37(1):106-113.
[64]Sun J,Hemler M E.Regulation of MMP-1 and MMP-2 production through CD147/extracellular matrix metalloproteinase inducer interactions[J].Cancer

Res,2001,61(5):2276-2281.
[65]Moore MJ,Hamm J,Dancey J,et al.National Cancer Institute of Canada Clinical Trials Group.Comparison of gemcitabine versus the matrix metalloproteinase inhibitor BAY 12-9566 in patients with advanced or metastatic adenocarcinoma of the pancreas:a phase III trial of the National Cancer Institute of Canada Clinical Trials Group[J].Clin Oncol.2003, 21: 3296 –3302.
[66]Gohji K,Fujimoto N,Komiyama T,et alElevation of serum levels of matrix metalloproteinase-2 and-3 as new predictors of recurrence in patients with urothelial carcinoma [J].Cancer,1996,78(11):2379-2387.

综 述

MMP-2 与食管癌、肺癌侵袭转移关系的研究进展

肖祥之 综述 蒋仲敏 审校

【摘 要】基质金属蛋白酶(MMPs)是在肿瘤发生和增殖过程中,由肿瘤细胞通过其表面的基质金属蛋白酶诱导因子（CD147）作用于邻近正常细胞分泌的一种锌依赖性蛋白水解酶,能够降解细胞外基质(extracellular matrix，ECM)，从而有利于肿瘤细胞向邻近组织、血管和淋巴管侵袭，为肿瘤转移创造条件；还能与 CD147 分子在肿瘤细胞表面形成复合物,这种复合物的形成将 MMP-2 浓缩在肿瘤细胞周围,更有利于肿瘤细胞周围基质的降解,使肿瘤细胞易于扩散和转移。近年来，基质金属蛋白酶-2(MMP-2) 作为降解细胞外基质的关键酶，对食管癌、肺癌的诊断、病程分期和病理分型、肿瘤的疗效检测及预后等方面越来越受到人们的重视。本文就基质金属蛋白酶(MMP-2)在食管癌、肺癌的生物学特性的关系的研究予以综述。

【关键词】基质金属蛋白酶，基质金属蛋白酶抑制剂，侵袭，转移

食管癌和肺癌是胸部外科中常见的恶性肿瘤。我国是世界上食管癌高发地区之一，每年因食管癌死亡人数占恶性肿瘤死亡人数的第 6 位，新增病例约占世界新增病例的 50%；肺癌发病率近几年有明显上升的趋势。由于食管癌、肺癌的淋巴结转移率及复发率高,其临床进展较快,预后较差,对人类健康的危害日益严重。随着内镜技术的不断进步，提高了对早期病变的认识水平，使得局限于黏膜内及黏膜下的食管癌的检出率有所提高,但仍未能够达到一个理想的水平。因此,食管癌、肺癌的早期诊断在食管癌的治疗和预后方面有着积极的意义。以往通过细胞学检查如活检及涂片等,耐受性和特异性差，可以辨识如结节,溃疡狭窄等，但对于一些早期癌症,尤其是在进展期发育阶段,经常看不出表面上的变化而判定为正常，导致误诊；而且许多新技术还存在不少的需要专家用组织学知识解释的现象，以及依靠操作经验带有很大的主观性；临床通过检测 p53、CEA、特异性烯醇化酶等来监测肿瘤的进展等，但经证实其特异性及灵敏度都不高。目前缺乏合适的筛查和早期诊断方法,导致许多患者直至出现临床症状或至疾病晚期才到医院就诊,成为上述肿瘤高死亡率原因之一。而肿瘤标志物以其高效、高灵敏、标本易获取及经济适用等优势为临床医生所采用。而近年来基质金属蛋白酶的研究较多,为恶性肿瘤的诊断带来了新的解

决方法。

MMPs又称为基质素或者基质金属蛋白酶，它是一类锌离子依赖性蛋白酶[1]。肿瘤细胞穿透基底膜的可能机制为：通过基质金属蛋白酶诱导因子等介导肿瘤细胞与间质细胞的相互作用，刺激肿瘤细胞本身和间质细胞，就可以直接分泌或诱导宿主细胞分泌MMPs，完成局部降解基底膜和基质，使肿瘤细胞沿着溶解的基质部位进一步向周围组织侵袭。MMPs作为蛋白水解酶,至今发现有24种，MMP-2只是其中之一，又称为明胶酶，在成纤维细胞及肿瘤周围的血管内中均有表达，而Ⅳ型胶原是其作用底物同时又是基底膜的主要成分，MMP-2可以很容易水解Ⅳ型胶原，破坏基底膜的完整性，达到侵袭转移的目的。有研究指出，在胃癌细胞中MMP-2呈异质性增强表达，符合浸润性生长方式和伴有淋巴结转移的患者的胃癌细胞中表现尤为突出[2]。

1 基质金属蛋白酶的结构

基质金属蛋白酶是一类具有Zn^{2+}依赖性的肽链内切酶，其结构由N端前肽区、催化区、铰链区与C端类血红素结合蛋白区等组成。MT-MMP还包括C端跨膜区。在N端前肽区内保守序列(PRCGVPDV)与MMP酶原激活关系密切。在距离酶活性催化功能区Zn^{2+}位点外有另一个Zn^{2+}位点，和至少一个Ca^{2+}结合区，在两个Zn^{2+}结合区中，一个为催化性Zn^{2+}，另一个为结构性Zn^{2+}，前者与MMP活化有关。铰链区与类红素结合蛋白区与大多数MMP底物特异性有关，跨膜区将MT-MMP固定于细胞膜。按作用底物不同主要分为五大类:明胶酶又称Ⅳ型胶原酶、间质胶原酶、膜型基质金属蛋白酶、基质溶酶和其他类。其它成员有类血红素结合蛋白的羧基端，底物的特异性是由4个重复序列约200个氨基酸决定的，并参与MMP与组织TIMP之间的相互作用的调节。

2 基质金属蛋白酶的功能

MMPs产生于正常组织细胞(包括结缔组织细胞、内皮细胞、胸腺细胞、巨噬细胞和淋巴细胞等)和肿瘤细胞,以酶原的形式分泌,进入细胞外间隙激活后才能降解ECM。MMPs具有高度的同源性,它们的一级结构中均含有两个高度保守区,体内激活机制为纤溶酶原的放大机制,其活性受金属蛋白酶组织抑制剂(TIMPs)的调节。二者在ECM代谢中的相互作用成为肿瘤细胞侵袭和转移的关键因素,研究MMPs在不同类型肿瘤中的表达和分泌水平对肿瘤的防治具有重要意义[3]。MMP-2是分子量为72kDA的明胶酶A,主要由巨噬细胞结缔组织

细胞和某些肿瘤细胞合成分泌,可降解多种细胞外基质(ECM)。MMPs表达升高多见于许多正常的生理过程(如组织发生、伤口愈合)，还和动脉粥样硬化、结缔组织疾病[4]以及多种肿瘤[5-7] 等的发生有关，在肿瘤的发病及侵袭转移中也起到了重要作用。MMPs依据不同的细胞类型而表现为多种不同的功能，其作用发挥有赖于不同的水解机制。正常情况下表达水平很低，一旦发生组织重建，如炎症、创伤愈合或发生癌症时， MMPs 则快速转录表达，被激活及分泌，在体内广泛表达[8]。

3 基质金属蛋白酶的作用机制

侵袭和转移是恶性肿瘤的重要特征,是宿主细胞、肿瘤细胞及ECM之间一系列复杂、多步骤互相作用的结果，在这一过程中包含着多次细胞外基质及基底膜的降解，其中，肿瘤细胞及ECM之间的互相作用是该过程的关键。对于肿瘤来说，基底膜是肿瘤细胞浸润扩散过程中的一道天屏障。Ⅳ型胶原纤维是ECM和基底膜的重要组成之一,而肿瘤细胞分泌的MMPs中,MMP-2是降解Ⅳ型胶原最主要的酶，在其进展中扮演了一个重要的角色，包括血管再生、侵袭（局部侵入、肿瘤细胞浸润和外侵）以及发生转移。它的侵袭方式为：细胞外基质包括基底膜和间质组织，肿瘤细胞以MMP-2酶原（proMMP-2）的形式分泌,进入细胞外间隙激活后，通过信号传导激活，产生活化的MMP-2，黏附于ECM 的基底膜，并通过已存在的或新形成的结合位点与细胞外基质基底膜的受体相结合，释放或激活蛋白水解酶，降解基底膜的主要成分明胶酶，从而破坏基底膜和细胞外基质的完整性,然后定向运动穿过缺损区，使肿瘤细胞沿着溶解的基质部位进一步向周围组织侵袭；肿瘤细胞转移的基本方式为：首先肿瘤细胞脱落，然后随着脱落的瘤细胞黏附并降解 ECM，从而进入微血管或微淋巴管并随之移动,到达继发部位的靶器官,外渗进而增殖形成单个转移肿瘤。大量分泌的MMP-2,为形成广泛转移奠定了基础。不管肿瘤细胞还是间质细胞，MMP-2在早期的表达，都有利于ECM的结构重建及ECM和/或膜结合生长因子的释放，这样就为原发肿瘤的形成提供一个良好的局部微环境。当然，它的转录和分泌同样受各种各样的因子的诱导,在多数情况下它以非活跃蛋白酶的形式隐匿,除非其他蛋白来激活或诱导，否则其不能自动活化。实验证实MMP的表达受基因、酶原活化及活化后调节三个水平的调控：

第一，基因水平的调节。许多因素都可能影响到MMP的表达，如激素、

肿瘤基因及生长因子和细胞因子等。MMP-2 活化的信号传导机制仍有待进一步研究。研究证实 MAPK 传导途径和 MMP-2 的表达相关。Kurata[9]等研究表明 v-src 转化细胞能转化活性形式的 MEKl，从而在很大程度上增加了 MMP-2 的分泌及活化；而 PD98059(一种 MEKl 特异性抑制物)却能使 MMP-2 的分泌和活化受到明显抑制；wortmannin(一种 P13 激酶抑制物)也并未影响 MMP-2 的分泌和活化。

第二，酶原的活化调节。酶原的活化的过程中 MMP 发挥着重要的作用。目前，酶原活化的过程有逐步激活、细胞表面激活以及细胞内激活等 3 个不同的机制。其一，酶原的逐步激活：明胶酶和胶原酶以及间质溶素等要以酶原形式(proMMP)分泌进入 ECM，必须先水解去除前肽区才能被活化而发挥作用。研究发现，前肽区保守序列中 Cys 的 SH 基和活性中心催化性 Zn^{2+}离子的结合成为酶原维持稳定的重要环节，酶原逐步激活关键是通过多种方式分离 Zn^{2+}-Cys 的连接，才能使水分子和 Zn^{2+}离子得以相互反应[10]。所以，Zn^{2+}-Cys 连接断裂才是 MMP 前体激活的关键；其二，细胞表面激活：膜型基质金属蛋白酶（MT-MMP）是通过其 c-末端的跨膜区定位在细胞膜上的。许多研究发现，MTl-MMP、MT2-MMP 以及 MT3-MMP 等都可以使 proMMP-2 转化成活性 MMP-2。Strongin 等[11]在伴刀豆球蛋白处理过的 HT-l080 细胞膜上分离出一种化合物，它能与 proMMP-2 的 C-末端区互相作用，经研究为 MTI-MMP-TIMP2 复合物，它能进一步和细胞膜上的受体相结合形成三聚体，从而激活 proMMP-2；其三，细胞内激活：MT-MMP 和 proMMP-2 可以在细胞内活化，然后再以活性形式表达或分泌，这一过程需要 Ca^{2+}等的参与。Munshi[12]等发现,肿瘤细胞经体外培养，加入 Ca^{2+}后可促进肿瘤细胞分泌 MMPs,继而促进肿瘤细胞的转移。Jiang[13]等报道细胞内 Ca^{2+}水平的调高可增强肝癌的转移潜力。Yue[14]等研究表明,Ca^{2+}拮抗剂 Mg^{2+}与肿瘤细胞在体外培养,可明显抑制 MMPs 尤其是 MMP-2 的表达及活化,提出 Mg^{2+}可能阻断了 Ca^{2+}信号传导通路，终止了酶的活化过程,起到抑制 MMPs 的表达与活化的作用,从而影响了肿瘤细胞侵袭和转移。

第三，活化后调节，即特异性抑制因子的抑制作用。尽管 MMPs 存在于人体多种细胞中，为避免组织过度损伤，通常其转录水平很低，这就需要 MMP 轴来高度调节。主要是通过天然的 MMP 抑制因子—组织金属蛋白酶抑制剂

(TIMPs)来进行调节。作为 MMP-2 天然抑制因子，TIMP-2 具有下调 MMP-2 的活性的能力，为维持 ECM 的稳态起到了重要作用。所以，只有 MMP-2 与 TIMP-2 保持平衡，才能保证 ECM 内环境的稳定和完整性。在肿瘤进展时，必然存在某种因素，使 MMP 过度表达或者表达程度超过了 TIMP 的表达时，这种平衡就被打破，从而导致基质降解，利于肿瘤的浸润、转移；反之，TIMP 过度表达或者表达程度超过 MMP 时，就可防止基质降解，阻碍了肿瘤浸润转移[15]。而 α-巨球蛋白作为 MMPs 的血浆抑制剂，也可抑制 MMPs 活性。此外，细胞因子和炎症因子以旁分泌或者自分泌的方式也有一定的调节 MMPs 和 TIMPs 的作用：对明胶酶起上调节作用的有以下几种：TNF-α、IL-1β 和血小板衍化生长因子-AB 以及 IL-6 和 IL-la；而下调的有 IL-4、IL-10 和 γ 干扰素。上调胶原酶是 TNF-α、IL-α、IL-1β、PDOF、内皮生长因子以及神经生长因子等；而下调的有 TGF-β、TNF-γ、IL-4 等。

4 基质金属蛋白酶与食管癌

许多研究表明基质金属蛋白酶在食管癌中表达升高，并且与 CD147 等因子的关系非常密切，多同时表达，而且呈现出正相关。陈超伍[16]等在研究中发现，食管癌 MMP-2 的表达率为 69.3%，远远高出在癌旁组织表达阳性率的 12.5%； MMP-2 在淋巴结转移组的阳性率也显著高于无转移组的阳性率，这提示 MMP-2 可能在食管癌的进展与肿瘤转移中发挥作用。李淳[17]等研究食管癌时发现,MMP-2 阳性表达率为 80.0%(36/45)，在 MMP-2 阳性表达病例中,阳性表达细胞为癌细胞,间质细胞呈阴性,仅 5 例均有 MMP-2 表达。Ⅰ级癌组织中，阳性细胞多分布于癌巢边缘；Ⅱ-Ⅲ级癌组织中，阳性细胞则散在分布，无规律性。MMP-2 表达与癌组织的浸润深度有关，癌浸润越深 MMP-2 阳性表达率越高。阳性表达与淋巴结转移和临床分期有关，与大体类型、分化程度等无关。该研究结果还提示, MMP-2 在食管癌的浸润过程中起重要作用。

有研究认为[18],单一 MMP-2 或/和 MMP-9 或 MMP-7 可能与胃癌、结肠癌、原发性肝癌、膀胱癌等的侵袭和转移有关,但 MMPs 与食管癌侵袭转移性的关系较小。但也有人认为 MMP-2 的过度表达与转移性肿瘤细胞的局部浸润和扩散之间具有很密切的关系[19],研究发现,食管癌中晚期的表达量明显高于早期,有淋巴结转移或远处转移者明显高于无转移的食管癌患者,提示 MMPs 在食管癌侵袭及转移过程中发挥着重要的作用,还与食管癌的预后密切相关，MMP-2

可作为临床预测肿瘤侵袭转移潜能的指标之一。

近年来的研究表明，MMP-2 还与其他因子协同作用。孙羿[20-22]等发现血管内皮生长因子（VEGF）、CD44v6 和 MMP-2 三者可能在促进血管生成在肿瘤的生长、浸润和转移等恶性生物学行为具有明显相关性并起重要作用。P53 作为一个抑癌基因功能明确，主要是参与细胞凋亡信号调控的过程，近来的研究证实这一蛋白同时又是一个重要的转录因子，还参与多种基因的调控，这其中包括 MMP-2 基因[23]。超过半数人类癌症显示 P53 蛋白突变或缺乏，有些实验证实 P53 功能的缺失也和 MMPs 的过度表达关系密切。已有的研究表明，MMP-2 确是 P53 作用的靶点，它的启动区有 P53 的特异结合位点， P53 能激活 MMP-2 的启动子，促使 MMP-2 表达升高[24]。Dvergugina[25]等证实整合素α5β3 能与 MTI-MMP 协同作用，促进 MMP-2 酶原快速转化为活性 MMP-2，作用于某些肿瘤恶变及浸润转移过程中。此外，转化生长因子(TGF)在恶性肿瘤中和高水平表达的 MMP-2 也有一定的内在关联[26]。随着肿瘤的快速增殖，可能由于瘤组织缺乏血氧供应，肿瘤的反馈机制启动，促使血管生成。在肿瘤新生血管的形成中，多种细胞因子以自分泌或旁分泌的形式参与此过程并相互作用，形成内皮细胞毛细血管，如血管内皮生长因子、转化生长因子等，均可间接激活 MMP，也可以直接诱导 MMP 基因的转录[27,28]。

MMP-2 及 TIMP-2 在食管癌中的阳性表达显著增强，与非肿瘤组织阳性表达有显著差异。在正常或非肿瘤情况下，食管组织的细胞外基质较稠厚而且硬度高，有利于维持细胞的形态和功能。而一旦转化为食管癌，随着恶性程度不同，癌细胞会不同程度增强分泌 MMPs(主要是 MMP-2)，促进 ECM 的降解而变得稀薄和疏松，利于瘤细胞的侵袭和转移，为食管癌向周围侵袭生长创造了适宜的微环境。与此同时，作为一种代偿性反应，食管会增加 TIMPs 的分泌，试图维持二者的平衡，但是其补偿反应并不充分，当然 MMP-2 与 TIMP-2 表达是失比例的，从而造成了肿瘤的生长、浸润和转移等恶性生物学行为。

5 基质金属蛋白酶与肺癌

国内外研究表明，MMPs 在肺癌组织表达升高，而在正常肺组织中低表达或不表达，而且 MMPs 的表达与肺癌的侵袭转移及其预后呈正相关[29-31]。

王新允[29]等以 S-P 免疫组织化学技术检测 MMP-1、MMP-2、MMP-9 和 MMP-13、TIMP-1 及 TIMP-2 等在 104 例肺癌组织中的表达时，发现肺癌组织

中阳性表达率均比正常组织明显升高，其中MMP-2表达与肺癌分级关系密切。吕志强[30]等以同样的方法检测了MMP-2在42例肺癌组织中的表达，证实肺癌组织中MMP-2的表达和淋巴结转移呈明显正相关，MMP-2表达高的预后较差，同时MMP-2与肿瘤血管形成关系密切。肺癌组织MMP-2的表达水平较癌旁肺组织明显升高，癌细胞分化程度越低，而TNM分期越晚或合并淋巴结转移，其癌组织中MMP-2的mRNA转录表达水平也越高。提示MMP-2的过度表达为肺癌细胞的浸润、转移创造了条件，MMP-2可能是通过降解细胞外基质中的基质成分促进肿瘤的侵袭和转移，对肺癌的发生、发展起重要作用。

关于肿瘤细胞和ECM间的相互作用对肿瘤侵袭和转移的影响抑制都是肿瘤生物学研究的热点，研究涉及各个专业，基质金属蛋白酶在肿瘤中的作用越来越受到人们的关注，目前已有的研究其表达和活性的常用技术方法有免疫组化、酶联免疫吸附（ELISA）、原位杂交、酶谱分析法、Northern 印迹法、Westrn 印迹法等。Delebecq[32]等采用Norlhem印迹技术等对119例非小细胞肺癌患者的肺癌标本中的uPA，MMP-2和MMP-11以及两种组织蛋白的表达进行研究时发现，MMP-2、MMP-11与肿瘤关系密切，而且在胰腺癌、膀胱癌等肿瘤中的表达也得到了证实[33,34]。MMP-2表达水平升高，与细胞外基质成分、多种细胞因子和原癌基因产物以及致癌物等有关，它们都能诱导多种细胞表达MMP。

6 基质金属蛋白酶的应用进展

MMP-2降解毛细血管及淋巴管基底膜，使肿瘤进入循环中，并更多分泌MMP-2，从而引起远处转移。所以，血液中MMP-2水平也应该升高。Hidefumi[35]等测定了肺癌患者血清中的MMP-2酶原水平，证实该水平的确比正常组明显升高，但与肿瘤的病理类型无关。Krzysztof[36]等在肾上腺肿瘤的研究中也证实了MMP-2血清水平升高，并且和肿瘤类型无关。肺癌组织表达阳性率和病理类型无关，这就造成肺癌组织阳性表达率在血清与组织的不一致，可能与MMP-2分泌的来源细胞不同有关，在肿瘤组织中，主要为肿瘤细胞、间质细胞为主，而血液中则以内皮细胞、肿瘤细胞和巨嗜细胞等分泌为主。此外，也可能和病例数少有关，尚待进一步研究。

Hidefumi[35]等还测定了肺癌患者的血清MMP-2酶原，结果表明IIIb期肺癌血清中的MMP-2浓度较I期明显升高。因为MMP-2酶原被激活后，即变成

活性金属蛋白酶，所以酶原的升高必然会引起 MMP-2 的升高，这也间接证明 MMP-2 和肺癌侵袭水平关系密切。在结肠癌、前列腺癌和胃肠道癌以及乳腺癌等患者的血或组织中，也证实 MMP-2 水平升高，且与病理分期有关。经过术后存活时间追踪，发现 MMP 及 TIMP 水平升高可以作为判断肿瘤分期及估计预后的指标[37-41]。Talvensaari Mattila[42]等对 177 个经过手术治疗的乳腺癌病人进行随访时发现，MMP-2 表达阳性率与病人的总生存期相关。通过 5 年随访，肿瘤 MMP-2 表达阴性的病人约 88%存活，而表达弱阳性及中度阳性的病人约 73%存活，强阳性的病人却只有 56%的存活，差别有统计学意义。所以，MMP-2 在判断肿瘤的侵袭、转移、恶性程度以及估计预后方面具有重要的临床意义。

基质金属蛋白酶抑制剂可以明显下调 MMP-2 的活性对维持细胞外基质的稳定意义重大。在正常情况下 MMP-2 及其抑制剂能保持相对平衡，保持了 ECM 的完整性和稳定性，一旦平衡被打破就会出现两个截然不同结果：MMP-2 过度表达，有利于基质降解和肿瘤的侵袭转移；或者是其抑制剂过度表达阻止了基质降解从而避免了肿瘤的侵袭转移，细胞外基质维持完整稳定[43]。有研究表明，MMPs 及 TIMPs 的表达还受血管内皮生长因子作用的调节[44]。Gohji[45,46]等测定部分侵袭性膀胱癌患者血清的 MMP-2/TIMP-2 比值时发现，MMP-2/TIMP-2 比值高者较低者更早出现复发，这说明 MMPs 及 TIMPs 可作为抗肿瘤治疗的良好标志物的同时，还可以区分复发高危因素的亚型；Davies[47]也经过研究指出，随着 MMPs 与 TIMPs 的比值升高，降解 ECM 的能力越来越强，肿瘤的侵袭能力也相应越来越强。Nuovo[48]等认为，肿瘤浸润和转移时，MMPs/TIMPs 比例失调比单纯的 MMPs 或 TIMPs 表达水平的变化意义更大。Gohji[49,50]等通过研究发现，测定膀胱癌患者血清 MMP-2 与 TIMP-2 的比值，可用来预测尿路上皮肿瘤是否复发。由于血清学方法为定量法测量，能够直接反映其血清中的浓度，且标本收集简便，可适用于任何患者；但免疫组化法的局限性在于：它是一定性试验，只能行病理组织学检查，因此只适用于手术或行纤支镜并取活检等有创性检查的患者。所以，在无法获得病理组织标本或条件所限时，可以通过外周静脉血清的测定，了解组织表达的情况，可以更简便地诊断肿瘤和判断病人预后。鉴于 TIMPs 对肿瘤有抑制作用，人们努力争取开发安全有效的 MMPs 抑制物，用来调节 MMPs/TIMPs 比例。目前，MMPIs 已

经研制出成品十余种。Taraboletti 等合成的第 1 代 MMP 广谱抑制剂巴马司他(Batimastat,BB-94)不仅具有抑制肿瘤生长及侵袭,还可抑制肿瘤的血管生成。Lee[51]等用巴马司他(batimastat)在治疗 MDA—MB-231 人乳腺癌细胞株接种所形成的长骨转移的裸鼠时发现，骨转移灶确实明显缩小。

7 研究方向

研究表明，利用基质金属蛋白酶抑制剂治疗肿瘤，马马司他已进入 III 期临床试验[52]，但均证实疗效并不是十分明显或疗效明显但副作用很大，所以利用其抑制剂来控制肿瘤的浸润及转移的方法还不是十分理想，我们期待会是一个疗效好但副作用小的抑制剂。我们不能仅仅局限于研究其抑制剂，有人提出以基质金属蛋白酶的诱导剂 CD147 为靶向分子，也可以有效地治疗肿瘤，且副作用较小。通过抑制 CD147 就能抑制基质金属蛋白酶的活性和分泌，而达到抑制肿瘤的浸润和转移的目的。CD147 的抑制剂除了上述作用外还在抑制血管和透明质酸的生成途径方面也有广阔的前景。基于控制癌症的需要，研究 CD147 更细节方面的作用机制包含每一个免疫球蛋白区的功能都是必要的。

综上所述，肿瘤细胞是以 MMP-2 酶原的形式分泌并进入细胞外间隙的，通过信号传导机制激活，产生活化的 MMP-2，黏附与 ECM 的基底膜，并通过已存在的或新形成的结合位点与细胞外基质基底膜的受体相结合，同时需要 Ca^{2+}、Zn^{2+}等多种因子的参与，释放或激活蛋白水解酶降解基底膜的主要成分明胶酶，从而破坏基底膜和细胞外基质完整性然后定向穿过缺损区，使肿瘤细胞沿着溶解的基质部位进一步向邻近组织侵袭；随着脱落的瘤细胞黏附并降解细胞外基质，从而进入微淋巴管或微血管并随之移动，到达继发部位的靶器官，外渗进而增殖形成转移瘤。MMP-2 一旦进入循环就可以检测其血液中的浓度，检测方便而且简单。在食管癌或肺癌患者的组织及血清中，MMP-2 及其抑制剂 TIMP-2 随着肿瘤的进展，MMP-2 增加而 TIMP-2 减少，所以 MMP-2 成为判断肿瘤的侵袭、转移和判断肿瘤分期及估计预后的重要指标，但因其血行转移发生较晚，作为一种肿瘤标志物其敏感性和可靠性仍不十分理想。从肿瘤标志物被正式开始应用至今,肿瘤标志物的应用日趋成熟,从单一肿瘤标志物的应用到联合检测；从单一辅助诊断到疗效评价和判断预后,肿瘤标志物的应用前景广阔。但目前还未发现针对食管癌、肺癌特异性和敏感性都较高的肿瘤标志物。因此今后的研究任务是积极探究和发现特异性、敏感性俱佳的肿瘤标志物,

并应用到临床。通过生物芯片等新的实验技术,使联合检测变得更加快速、简便和可靠。

参考文献

[1] Zhao H,Bemardo M M,Osenkowski P,et al.Differential inhibition of membrane type3(MT3)-matrix metalloProteinase(MMP) and MT1-MMP by tissue inhibitor of metalloProteinase(TIMP)-2 and TIMP-3 regulates Pro-MMP-2 activation [J].J Biol Chem,2004,279(10):8592-8601.

[2] 王瑞年,朱延波,薛建元等.胃癌浸润转移与整合蛋白、IV 型胶原酶及细胞外基质的关系[J].中华病理学杂志,1994,13(5):278-283.

[3] 侯振江,张宗英.基质金属蛋白酶系统与恶性肿瘤[J].国外医学临床生物化学与检验学分册,2004,25(1):83-85.

[4] Mengshol J A,Mix K S,Brinckerhoff C E.Matrix metalloproteinase as therapeutic targets in arthritic diseases:bull's eye or missing the mark[J].Arthritis Rheum,2002,46(1):13-20.

[5] Komosinska-Vassev K,Olczyk P,Winsz-Szczotka k, et al. Age and gender dependent changes in connective tissue remodeling: physiologic differences in circulating MMP-3,MMP-10,TIMP-1 and TIMP-2 level [J]. Gerontology, 2010, (3):1-9.

[6] Gao Z L, Zhang C,Du G Y,et al.Clinical significance of changes in tumor markers, extracellular matrix,MMP-9 and VEGF in patients with gastric carcinoma [J]. Hepatogastroenterology,2007,54:1591-1595.

[7] Fridman R.Metalloproteinases and cancer[J].Cancer Metastasis Rev,2006,25:7-8.

[8] Wu C Y,Hsieh H I,Jou M J,et al.Involvement of p42 / p44 MAPK,p33 MAPK, JNK and nuclear factor-kappa B in inter leukin- 1 beta-induced matrix metallo- proteinase-9 expression in rat brain astrocytes[J].J Neurochem, 2004, 90(6):1477.

[9] Kurata H,Thant AA,Matsuo S,et a1.Constitutive activation of MAP kinase kinase(MEKl)is critical and sufficient for the activation of MMP-2[J].Exp Cell Res,2000,254(1):180-188.

[10] Van Wart H E,Birkedal Hansen H.Thecysteine switch:a principle of regulation of metalloprOteinaSe activity with potential applicability to the entire matrix metalloproteinase gene family[J].J Biol Chem,1995,270(10):5331-5338.

[11] Strongin AY,Collier I,Bannikov G,et a1.Mechanism of cell surface activation of 72-kDa type IV collagenase.Isolation of the activated form of the membrane metalloprotease[J].J Biol Chem,1995, 270(10):5331-5338.

[12] Munshi HG,Wu YI,Ariztia EV,et al.Calcium regulation of matrix metallo proteinase-mediated migration in oral squamous cell carcinoma cells[J].J Biol Chem, 2002,277:41480

[13] Jiang JL,Zhou Q,Yu MK,et al.The involvement of Hab18G/CD147 in regulation of store-operated calcium entry and metastasia of human hepatoma cells[J].J Biol Chem,2001,276:46870.

[14] Yue H,Lee JD,Shimizu H,et al.Effect of magnesium on the production of extracellular matrix metalloproteinase incultured rat vascular smooth muscles cells[J]. Athero-sclerosis, 2003,166:271.

[15] Grignon D J,Sakr W,Toth M,et a1.High levels of tissue inhibitor of metallo-proteinase-2 (TIMP-2) expression are associtated with poor outcome in invasive bladder cancer[J].Cancer Res,1996,56 (7):1654-1659.

[16] 陈超伍,马洪升.基质金属蛋白酶在食管癌组织中的表达及与微量元素含量之间关系[J].世界华人消化杂志,2010,18(19):1995-2000.

[17] 李淳,吴名耀,吴贤英.食管癌 MMP-2、MMP-9 及其组织抑制剂 TIMP-1、TIMP-2 的表达及临床意义实用癌症杂志,2004,19(2):139-141.

[18] Samantaray S,Sharma R,Chattopadhyaya TK,et al.Increased expressi- on of MMP-2 and MMP-9 in esophageal squamous cell carcinoma[J].Cancer Res Clin Oncol, 2004,130: 37-44.

[19] Tanioka Y,Yoshida T,Yagawa T,et al.Matrix metalloproteinase-7 and matrix metalloproteinase-9 are associated with unfavourable prognosis in superficial oes- ophageal cancer[J].Br J Cancer,2003,89:2116-2121.

[20] LinksChe G,Chen J,Liu L,et al.Transfection of nm23-H1 increased expression of beta-Catenin,E-Cadherin and TIMP-1 and decreased the expression of MMP-2, CD44v6 and VEGF and inhibited the metastatic potential of human non-small cell lung cancer cell line L9981[J].Neoplasma,2006,53(6):530-537.

[21] Zheng H,Takahashi H,Murai Y,et al.Expressions of MMP-2,MMP-9 and VEGF are closely linked to growth, invasion,metastasis and angiogenesis of gastr- ic carcinoma[J]. Anticancer Res,2006,26(5A):3579-3583.

[22]孙羿,何辉,马强,等.膀胱肿瘤抗原利透明质酸等七项肿瘤标志物在膀胱肿

瘤诊断中的应用价值[J].中华医学杂志,2005,85(35):2507-2512.

[23] 范金兰,王伟萍,吴小华.基质金属蛋白酶释放与活化的研究进展[J].中国癌症杂志,2002,12(3):269-272.

[24] Yarnada S D, Baldwin HL, Karlan BY Ovarian carcinoma cell cultures are resistant to TGF-betal-mediated growth inhibitiondespite expression of functional receptors[J]. Gynecol Onco1,1999,750):72-77.

[25] Dergugina El,Ratnikov B,Monosov E,et a1.MTl-MMP initiates acti- vation of pro-MMP-2 and integrin α5 β3 promotes maturation of MMP-2 in breast carcino- ma cells[J].Exp Cell Res.2001,263(2):209-223.

[26] Velasco P,Lange-Asschenfeldt B.Dermatological aspects of angio- genesis[J]. Br.J.Dermato1,2002,147:841-852.

[27] Beckal Jr D,Amore,P A.Vascular development:cellular and molecular regula-tion[J].FASEB J,1997,11:365-373.

[28] Obes A B,Sporn M B.Regulation of endothelial cell growth, arch itecture, and matrix synthesis by TGF-B[J].Am J Res Dis,1989,140:1126-1128.

[29]王新允,杨菊红,马莹,等.肺癌 MMPs 和 TIMPs 的表达及浸润转移的研究[J].中国肺癌杂志,2003,6(4):278-282.

[30]吕志强,李海刚,谢德荣等.肺痈组织中 MMP-2 表达及与肿瘤血管形成关系[J].中国肿瘤,2004,13(6):378-380.

[31] Susskind H,Hymowitz M H,Lau Y H,et a1.Increased plasma levels of matrix metalloproteinases-9 and tissue inhibitor of meralloproteinase-1 in lung and breast cancer are altered during chest radiotherapy[J].Int J Radiat Oncol Biol Phys, 2003, 56(4):1161- 1169.

[32] Delebecq TJ, Porte H,Zerimech F,et a1.Overexpression level of stom- elysin S is related to lymph node involvement in non-small cell lung cancer[J].Clin Cancer Res,2000,6 (3):l086-1092.

[33] Bei B,Bucana CD,Fidler IJ.Density—dependent induction of 92-Kd type IV collagenase activityin cultures of A43 1 humanepidermoid carcinoma cells[J].Am J Pathology,1994,114:1058-1067.

[34] Bian J,Sun Y.Transcriptional activation by p53 if the human type IV collage-nase (gelatinase A or matrix metalloproteinase-2) promoter [J].Mol Cell Bio1, 1997, 17:6330-6338.

[35] Hidefumi Sasaki, Masanobu Kiriyama, Ichiro Fukai, et a1. Elevated serum

pro-MMP2 levels in patients with advanced lung cancer are not suitable as a prognostic marker. Surgery Today[J].2002,32:93-95.

[36] Krzysztof Kolomecki,Henryk Stepien,Magdalena Bartos,et al.En-docrine Regulation[M].2001,35:9-46.

[37] Zucker S,Lysik R Vl,Zarrabi MH,et al.92000 type IV collagenase is increased in plasma of patients with colon cancer and breast cancer[J].Cancer Res,1993,53: 140-146.

[38] Endo K,Maehare Y,Baba H,et al.Elevated levels ofserum and plasma metalo-proteinases in patients with gastric cancer[J].Anticancer Res,1997,17: 2253-2258.

[39] Gohji K,Fujimoto N,Ham I,et al.Serum matrix metalloproteinase-2 and its density in men with prostate cancer as a new predictor ofdisease extension[J].Int J Cancer,1998,79:96-101.

[40] Oberg A,Hbyhtya M,Tavelin B,et al.Limited value of preoperative serum analyses of matrix metalloproteinases(MMP-2,MMP-9)and tissue inhibitors of matrix metalloproteinases(TIMP-1,TIMP-2)in colorectal cancer[J].Anticancer Res, 2000,20:1085-1092.

[41] Erynck R,Akhurst R J,Balmain A.TGF-13 signaling in tumor suppressi on and cancer progression[J].Nat Genet,2001,29:117-129.

[42] Talvensari Mattila A,Paakko P,Hoyhtya M,et al.Matrix metallopro- teinase-2 immunoreactive protein: a marker of aggressiveness in breast carcinoma[J]. Cancer, 1998,83(6):1135-1162.

[43] Grignon DJ,Sakr W,Toth M,et al.High levels of tissue inhibitor of metallopr-oteinase-2(TIMP-2) expression are associtated with poor outcome in invasive bladder cancer[J]. Cancer Res,1996,56(7):1654-1659 .

[44]Ylisirni G S,Hyhty M,Turpeermiemi HujanenT.Serum matrix metalloprotein-ases-2,-9 and tissue inhibitors ofmetalloproteinases-1,-2 in lung cancer-TIMP-l as a prognostic marker[J].Anticancer Res,2000,20:1311-1316.

[45] Gohji K,Fujimoto N,Fujii A,et al.Prognostic significance of circu- lating matrix metalloproteinase-2 to tissue inhibitor of metalloproteinases-2 ratio in rec- urrence of urothelial cancer after complete resection[J].Cancer Res,I996, 56: 3196.

[46]Gohji K,Fujimoto N,Ohkawa J,et al.Imbalance between serum matrix metallo-proteinase-2 and its inhibitor as a predictor of recurrence ofurothelial

cancer[J].Br J Cancer,1998,77:650.

[47] Davies B, Miles DW, Happerfield LC, et al.Activity of type IV collagenases in benign and malignantbreast disease[J].Br J Cancer,1993,67:1126.

[48] Nuovo G J,Macconnell P B,Simbir A,et al.Correlation of the in situ detection of polymerase chainreaction-amplified MMPcomplementary DNAs and their inhibitors with prognosis in cervical carcinoma[J].Cancer Res,1995,55:267-275.

[49] Gohji K,Fujimoto N,Komiyama T,et al.Elevation of serum levels of matrix metalloproteinase-2 and-3 as new predictors of recurrence in patients with urothe- lial carcinoma[J].Cancer,1996,78(11):2379-2387.

[50] Gohji K, Fujimoto N, Komiyama T,et al.Prognostic significance of circulate-ng matrix metalloproteinase-2 to tissue inhibitor of metalloproteinases-2 ratio in recurrence of urothelial cancer after complete resection[J].Cancer Res,1996,56 (14):3196-3198.

[51] Lee J, Weber M, Mejia S, et al. A matrix metalloproteinase inhibitor batima-stat, retards thedevelopment of osteolytic bone metastases by MDA.MB-231 hum- an breast cancer cells in Balb C nunu mice[J].Eur J Cancer, 2001, 37(1): 106-113.

[52] Deeg H J,Blazar B R,Bolwell B J,et al.Treatment of steroidrefractory acute craft-versus-hostdisease with anti-CD147 monoelonal antibody ABX-CBL[J]. Blood, 2001,98 (7):2052-2058.

致 谢

首先衷心感谢我的导师蒋仲敏教授对我的悉心培养和亲切关怀!蒋教授正直、热情，工作上一丝不苟，言传身教，还一直关注课题的进展与论文写作，给予我很多指导意见，使我在科研能力和临床技能方面有了长足的进步，是我学习的楷模；生活上平易近人，和蔼可亲，给予了我无微不至的关怀与帮助。

衷心感谢在研究生学习期间胸外科臧琦、宋晓明、王伟以及病理科孙青、周萍等老师给予的指导和无私帮助。

感谢就读研究生期间在学习、工作和生活上给予我关心和帮助的所有老师和朋友们。

最后还要感谢我的父母、爱人和女儿，他们的关爱和支持，使我在学习和工作中不断进步。

攻读硕士学位期间发表的学术论文

1．MMP-2 与食管癌及肺癌生物学特性的关系的研究进展。已投稿，待发表。

2. 斜切口吻合技术在预防食管癌术后吻合口狭窄的应用。已投稿，待发表。

原 创 性 声 明

本人郑重声明：所呈交的学位论文，是本人在导师的指导下，独立进行研究所取得的成果。除文中已经注明引用的内容外，本论文不包含任何其他个人或集体已经发表或撰写过的科研成果。对本文的研究作出重要贡献的个人和集体，均已在文中以明确方式标明。本人完全意识到本声明的法律责任由本人承担。

论文作者签名：________ 日 期：2011.6.7

关于学位论文使用授权的声明

本人完全了解泰山医学院有关保留、使用学位论文的规定，同意学校保留或向国家有关部门或机构送交论文的复印件和电子版，允许论文被查阅和借阅；本人授权泰山医学院可以将本学位论文的全部或部分内容编入有关数据库进行检索，可以采用影印、缩印或其它复制手段保存论文和汇编本学位论文。

(保密论文在解密后应遵守此规定)

论文作者签名：________ 导师签名：________ 日 期：2011.6.7
