# Supplementary material for: Expression of Concern: The prognostic and clinicopathologic characteristics of CD147 and esophagus cancer: A meta-analysis
Source: PLoS One. 2023 Feb 22;18(2):e0282229. doi: 10.1371/journal.pone.0282229 (PMC9946197; doi:10.1371/journal.pone.0282229)
Supplement: S1 File — (ZIP) [file pone.0282229.s001.zip › PDF of included paper/CD147íóMMP9íóS100A4╡░░╫╡─▒φ┤∩╙δ╩│╣▄┴█╫┤╧╕░√░⌐╫¬╥╞╡─╣╪╧╡.pdf]

DOI:10.3971/j.issn.1000-8578.2009.07.016

# CD147、MMP9、S100A4 蛋白的表达与食管鳞状细胞癌转移的关系

陈金绪[1],蔡科军[1],陈月新[2]

## Relationship between Metastasis of Esophageal Squamous Cell Carcinoma and Expression of CD147, MMP9 and S100A4

CHEN Jin-xu[1], CAI Ke-jun[1], CHEN Yue-xin[2]

1. *Department of Basic Medicine, Liuzhou Medical College, Liuzhou* 545006, *China*; 2. *Clinical Laboratory of The People's Hospital of Yulin City*

**Abstract: Objective** To investigate the expression of CD147, MMP9 and S100A4 in esophageal squamous cell carcinoma and further to explore the correlation between their expression and metastasis of esophageal squamous cell carcinoma's. **Methods** Immunohistochemistry(Maxvison) was used to detect the expression of CD147, MMP9 and S100A4 in 50 cases of esophageal squamous cell carcinoma tissues and 10 cases of normal esophageal tissues, respectively. **Results** Significant difference was observed in the expression of CD147, MMP9 and S100A4 between esophageal squamous cell carcinoma tissues and normal esophageal tissues; The expression of CD147 and S100A4 was positively related with the expression of MMP9 respectively. **Conclusion** The expression of CD147, MMP9 and S100A4 involves the occurrence of esophageal squemous cell carcinoma; they are all positively correlated with the metastasis of esophageal squamous cell carcinoma, and may work cooperatives.

**Key words**: Esophageal squemous cell arcinoma; CD147; MMP9; S100A4; Immunohistochemistry

**摘 要:目的** 研究 CD147、MMP9、S100A4 蛋白在食管鳞状细胞癌中的表达,探讨其与食管鳞状细胞癌侵袭和转移的关系。**方法** 采用免疫组织化学(Maxvison 法)检测 10 例食管癌旁正常组织和 50 例食管癌切除组织中 CD147、MMP9、S100A4 的表达。**结果** CD147、MMP9、S100A4 蛋白在食管鳞状细胞癌组织的表达与其在癌旁组织的表达差异均有统计学意义; MMP9 的表达分别与 CD471、S100A4 的表达呈正相关。**结论** S100A4、CD147 和 MMP9 蛋白表达均参与了食管磷状细胞癌的发生发展; 三者均与食管鳞状细胞癌的转移密切相关,且三者之间具有协同性。

**关键词**:食管磷状细胞癌;CD147;MMP9;S100A4;免疫组织化学

**中图分类号**:R361⁺.2;R735.1 **文献标识码**:A **文章编号**:1000-8578(2009)07-0596-04

## 0 引言

浸润性食管鳞状细胞癌是指原发于食管的鳞癌呈浸润性生长,可侵及食管壁的深层、食管旁组织甚至远处转移,是我国常见的恶性肿瘤之一。目前食管鳞状细胞癌的转移仍然是困扰临床治疗的难题。肿瘤的转移必须突破它原有的细胞外组织学屏障,基质金属蛋白酶家族由于其对细胞外基质的酶解作用而在肿瘤侵袭转移中发挥重要作用,而基质金属蛋白酶 9(MMP9)属于该家族的主要成员,能降解细胞外基质和基底膜中的主要成份,尤其是Ⅳ型胶原,使肿瘤细胞突破原发部位而发生侵袭和转移[1]。CD147 又称为基金属蛋白酶诱导因子,已被证实在体外能诱导 MMPs 的表达,促进 MMPs 的酶解功能[2]。而 S100A4 蛋白属于钙离子结合蛋白,最近研究发现,在多种肿瘤中,S100A4 蛋白与细胞增殖和肿瘤进展有关,且有报道提示 S100A4 可能参与 MMPs 的表达调节过程[3]。故本研究以 CD147、MMP9 和 S100A4 这三个与肿瘤转移密切相关的指标为检测对象,探讨它们在食管鳞状细胞癌组织中的表达情况及相互关系,从而揭示此三者的表达在食管鳞状细胞癌转移中的作用以及它们用于辅助临床判断患者预后的价值。

**收稿日期**:2008-06-05;**修回日期**:2008-07-31

**作者单位**:1. 545006 广西省柳州医专组胚教研室;2. 广西玉林市第一人民医院检验科

**作者简介**:陈金绪(1974-),男,硕士,讲师,主要从事病理组织学研究

## 1 资料与方法

### 1.1 资料来源

收集玉林市第一人民医院 2003～2006 年期间手术切除的浸润性食管鳞状细胞癌确诊标本 50 例作为研究对象，所有标本术前皆未行放、化疗或其他特殊治疗，其中男 28 例，女 22 例，年龄范围 27 岁～65 岁，平均年龄 42 岁。50 例病例中伴有淋巴结转移者 26 例(指手术清扫的锁骨上、食管旁或肺门淋巴结组织经病理镜检诊断为阳性的病例)，无淋巴结转移者 24 例。另取癌旁正常食管黏膜组织标本 10 例(指癌组织切缘旁组织，经病理诊断为正常黏膜组织，无肿瘤组织残留的标本)作为正常对照组。

1.2 主要试剂

兔抗人 CD147 多克隆抗体，鼠抗人 MMP9 的单克隆抗体、兔抗人 S100A4 多克隆抗体和 Maxvison™二抗试剂盒均购自福州迈新生物技术公司。

1.3 免疫组织化学染色 Maxvison™法检测 CD147、MMP9 和 S100A4 的表达

石蜡切片(3～5 μm 厚)常规脱蜡、水化后用 PBS(pH＝7.4)冲洗 3 次，每次 3 min；采用各种抗原相应的修复方法(用 EDTA(pH＝9.0)沸液热修复 CD147，微波炉高温柠檬酸修复 MMP9 和 S100A4)修复组织抗原；待切片自然冷却后，每张切片滴加 50 μl 3%的过氧化氢，室温下孵育 10 min，以阻断内源性过氧化物酶；每张切片滴加 50 μl 的第一抗体，室温下孵育 2 h；每张切片滴加 50 μl 即用型 Maxvison™二抗试剂，室温下孵育 15 min；各步骤之间皆用 PBS(pH＝7.4)冲洗 3 min×3 次。漂洗完二抗之后，每张切片加 100 μl 新鲜配制的 DAB 溶液，显微镜下观察显色 3～5 min；自来水冲洗切片，苏木精复染，自来水冲洗返蓝；切片经梯度酒精脱水干燥，二甲苯透明，中性树胶封固。

1.4 结果判断

1.4.1 研究对象的形态学特点

收集的 50 例食管鳞状细胞癌标本的病理切片镜下显示，肿瘤组织呈巢团状生长，瘤细胞核大深染，部分区域可见角化珠和细胞间桥，符合浸润性鳞癌的形态学表现，见图 1。

1.4.2 免疫组织化学结果判断

CD147 的阳性部位主要出现在细胞膜，少数在细胞质，以肿瘤细胞出现棕黄色颗粒为阳性。MMP9 阳性表达部位主要在肿瘤细胞胞浆，以出现棕黄色颗粒为阳性。S100A4 阳性染色为胞质呈棕黄色，胞核可少量着色。三者阳性判断方法：高倍镜下随机选取 5 个不同的视野，各计 200 个细胞，根据阳性肿瘤细胞占所有肿瘤细胞的百分比进行半定量分析：切片中完全无表达或阳性染色细胞率＜10%者为阴性(－)；阳性染色细胞率在 10%～25%或染色浅，为弱阳性(＋)；阳性染色细胞率在 26%～50%染色清晰，为阳性(＋＋)；阳性染色细胞率＞50%阳性或染色深，为强阳性(＋＋＋)。

在所有的免疫组织化学染色过程中，以 PBS 代替一抗作为阴性对照，公司提供的阳性对照片作为阳性对照。

1.5 统计学方法

两样本率比较采用 $\chi^2$ 检验；CD147 和 S100A4 表达与 MMP9 表达之间的相关性分析采用 Spearman 等级相关分析。$P<0.05$认为有统计学意义。

## 2 结果

2.1 CD147、MMP9、S100A4 蛋白的表达特征

CD147 主要表达于细胞膜，部分表达于细胞膜和(或)细胞质，呈棕黄色颗粒，见图 2。MMP9 主要表达于肿瘤细胞胞质，呈棕黄色颗粒，见图 3。S100A4 蛋白阳性表达部位主要定位于细胞质中，少数定位于胞核，呈棕黄色细颗粒状，见图 4。

2.2 CD147、MMP9、S100A4 蛋白分别在食管癌组和对照组中的表达情况

见表 1。CD147 在对 10 例正常食管黏膜组织

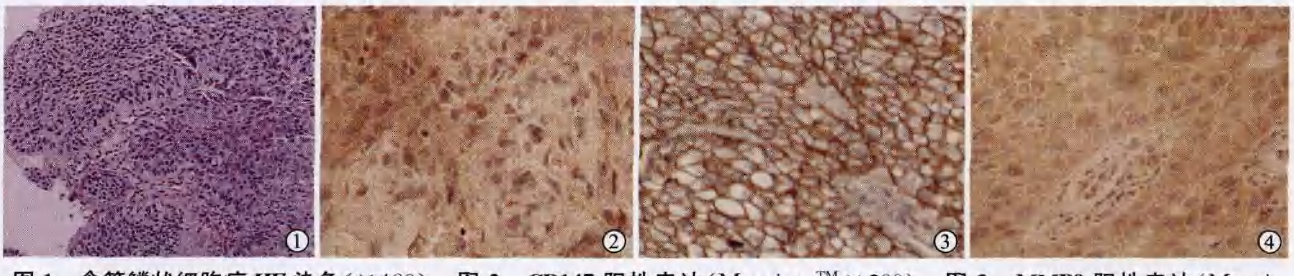

**图 1 食管鳞状细胞癌 HE 染色**(×100) **图 2 CD147 阳性表达**(Maxvison™×200) **图 3 MMP9 阳性表达**(Maxvison™×200) **图 4 S100A4 阳性表达**(Maxvison™×200)

**Figure 1 The HE staining of esophageal squamous cell carcinoma**(×100) **Figure 2 The positie expression of CD147**(Maxvison™×200) ***Figure 3 The positive expression of MMP9***(Maxvison™×200) **Figure 4 The postitive expression of S100A4**(Maxvison™×200)

表 1　CD147、MMP9 和 S100A4 蛋白在食管鳞状细胞癌组织和对照组中的表达

Table 1　The expression of CD147, MMP9 and S100A4 in the esophageal squamous cell carcinoma and control tissues

| Groups | $n$ | CD147 | | | | % | MMP9 | | | | % | S100A4 | | | | % |
|---|---|---|---|---|---|---|---|---|---|---|---|---|---|---|---|---|
| | | − | + | ++ | +++ | | − | + | ++ | +++ | | − | + | ++ | +++ | |
| Control | 10 | 9 | 1 | − | − | 10 | 8 | 2 | − | − | 20 | 7 | 2 | 1 | − | 30 |
| ESC | 50 | 5 | 25 | 12 | 8 | 90* | 6 | 16 | 20 | 8 | 88* | 8 | 30 | 7 | 5 | 84▲ |
| NM | 24 | 5 | 12 | 5 | 2 | 79.2 | 6 | 10 | 5 | 3 | 75 | 6 | 15 | 2 | 1 | 75 |
| M | 26 | 0 | 13 | 7 | 6 | 100# | 0 | 16 | 5 | 5 | 100# | 2 | 15 | 5 | 4 | 92.3# |

ESC: Esophageal squamous cell carcinoma group; NM: Not metastatic group; M: Metastatic group; − : A blank; * : The comparison with control group, $P<0.005$; ▲ : The comparison with control group, $P<0.01$; # : The comparison with not metastatic group, $P<0.05$

和 50 食管鳞状细胞癌组织中的阳性表达率分别为 10%(1/9)和 90%(45/50)，且差异有统计学意义($P<0.005$)，即说明 CD147 在食管鳞状细胞癌组织的表达高于正常对照组。MMP9 在食管鳞状细胞癌组织中的阳性率为 88%(44/50)，显著高于其在对照组中的阳性率 20%(2/10)($P<0.005$)。此外，S100A4 在食管鳞状细胞癌组织中的阳性率为 84%(42/50)，也显著高于其在对照组中的阳性率 30%(3/10)($P<0.01$)。CD147、MMP9 和 S100A4 在食管鳞状细胞癌组织中的高表达表明它们可能共同参与了食管鳞状细胞癌的发生。

2.3　CD147、MMP9、S100A4 蛋白分别在食管癌淋巴结转移组和无淋巴结转移组中的表达情况

见表 1。本研究结果显示，CD147 在 26 例伴有淋巴结转移组和 24 例无淋巴结转移组中的阳性表达率分别为 100%(26/26)和79.2%(19/24)，且差异有统计学意义($P<0.05$)，即说明 CD147 在伴有淋巴结转移的食管鳞状细胞癌组织中的表达高于其在无淋巴结转移的食管鳞状细胞癌组织中的表达。MMP9 在伴有淋巴结转移的食管鳞状细胞癌组中的阳性率为 100%(26/26)，显著高于其在无淋巴结转移的食管鳞癌组中的阳性率 75%(18/24)($P<0.05$)。此外，S100A4 在伴有淋巴结转移的食管鳞状细胞癌组中的阳性率为92.3%(24/26)，也显著高于其在无淋巴结转移的食管鳞状细胞癌组中的阳性率 75%(18/24)($P<0.05$)。结果表明 CD147、MMP9 和 S100A4 的高表达都与食管鳞状细胞癌的淋巴结转移密切相关。

2.4　CD147 和 S100A4 与 MMP9 蛋白表达之间的关系

经 Spearman 等级相关分析发现，CD147 的表达与 MMP9 的表达呈正相关($r=0.366$, $P<0.01$)；而 S100A4 的表达与 MMP9 的表达也呈明显的正相关性($r=0.339$, $P<0.01$)。

## 3　讨论

肿瘤的转移是多步骤的，是肿瘤细胞和周围正常组织相互作用的结果。这过程中最主要的是肿瘤细胞周围组织学屏障的破坏[4]，而基底膜和细胞外基质构成肿瘤细胞在侵袭转移过程中第一道组织学屏障。MMP9 是属于 MMPs 家族中分子量最大的明胶酶，也是降解基底膜和细胞外基质的主要蛋白酶之一。本研究发现，MMP9 蛋白在食管鳞状细胞癌细胞中的表达显著高于其在正常食管黏膜的表达，且其在伴有淋巴结转移的食管癌细胞中的表达也显著高于其在无淋巴结转移的癌细胞的表达。这一结果表明，MMP9 的高表达不但与食管鳞状细胞癌的发生有关，且与食管鳞状细胞癌的转移也密切相关。这与 Tanioka 等[5] 的报道结果相一致。此外，研究表明 MMP9 促进肿瘤的转移还与其通过促进血管内皮生长因子的表达有关[6]。

CD147 是存在于肿瘤分子表面的一种黏附分子，CD147 属于免疫超家族成员，最初命名细胞外基质金属蛋白酶诱导子[7]。CD147 分子结构上有一个典型的亮氨酸拉链结构，使得 CD147 具有与靶 DNA 相互作用从而调控基因表达的功能。本研究结果显示，CD147 高表达于食管鳞状细胞癌细胞，且伴有淋巴结转移癌细胞的表达水平高于无转移，说明 CD147 同样也参与了食管鳞状细胞癌的发生，且与食管鳞状细胞癌的淋巴结转移密切相关，这与 Eben 等[8]研究结果相符。近年来一个重要的体外实验证明，CD147 能促进肿瘤细胞 MMPs 的表达，这也是 CD147 促进肿瘤转移的主要机制之一。本研究也发现，在食管鳞状细胞癌组织中 CD147 的表达与 MMP9 的表达之间呈正相关，与上述观点吻合。当然，CD147 促进肿瘤侵袭转移还与其自身其他一些生物学功能有关，如上调肿瘤组织的血管内皮生长因子的表达[9]。本研究中 CD147 和 MMP9 的表达之间的正相关说明两者在食管癌的转移中具有很强的协同效应，共同参与食管癌的淋巴结转移。

S100A4 基因定位于 1q21，其编码的 S100A4 蛋白属于钙结合蛋白[10]，被认为是一种肿瘤转移相

关蛋白，近年受到关注。S100A4 与促进肿瘤转移方面的功能有关，包括抑制 p53 依赖性细胞凋亡[11]；S100A4 基因表达伴随上皮型钙粘蛋白表达缺失[12]及细胞表面的 CD44 表达增强，使肿瘤细胞间黏附力降低而与基质黏附性增强，促进肿瘤侵袭转移；通过上调 MMPs 的表达[13]或者影响 MMPs 与 TIMPs 间的平衡，加速基底膜的降解，促进肿瘤转移；增加肿瘤细胞的运动性[14]、增强血管内皮细胞的“爬行”，促进新生血管形成等[3]。本研究结果显示，S100A4 蛋白高表达于食管鳞状细胞癌组织，且其在伴有淋巴结转移的食管鳞状细胞癌组织的表达也显著高于无淋巴结转移的癌组织，说明 S100A4 参与食管癌的发生发展，且与食管鳞状细胞癌的转移也是密切相关的，与 Kimura 等[15]的报道一致。此外，在食管鳞状细胞癌组织中 S100A4 的表达与 MMP9 的表达存在正相关关系，说明 S100A4 通过上调 MMPs 的表达重塑细胞外基质而促进肿瘤的转移，与 Bjornland 等[16]的研究结果相符。S100A4 上调 MMP9 具体机制尚不十分清楚，有报道提示可能与其激活 NF-κB 信号通路而诱导 MMP 的合成有关[17]。

本研究探讨了 CD147、MMP9 和 S100A4 这三个与肿瘤转移密切相关的指标在食管鳞状细胞癌组织中的表达情况，并揭示了它们表达之间的相互关系。研究结果提示，CD147、MMP9 和 S100A4 都与食管鳞状细胞癌的转移密切相关，且三者之间有协同效应。所以，联合检测 CD147、MMP9 和 S100A4 的表达在判断食管鳞状细胞癌患者的早期转移、评价患者预后方面具有一定的价值。诚然，CD147、MMP9 和 S100A4 在促进肿瘤转移的机制方面尚有待于更深入的研究，尤其是 CD147 和 S100A4 参与调节 MMP9 的表达的确切机制目前还不清楚，而对此方面有关信号转导的研究也将必定成为肿瘤分子生物学研究的热点。

**参考文献：**

[1] Jin-ming Y, zude X, Hao W, et al. Overexpression of extracelluar matrix metalloproteinase inducer in multidrug resistant cancer cells[J]. Mol Cancer Res,2003,19(6):420-427.

[2] 申志华，揭伟，姜汉国，等. CD147、MMP-2 与 MMP-9 在 NHL 中的表达及临床病理意义[J]. 肿瘤防治研究，2005,32(7): 420-422.

[3] Schmidt B, Ornas D, Grigorian M, et al. Extracellular S100A4 (mts1) stimulates invasive growth of mouse endothelial cells and modulates MMP-13 matrix metalloproteinase activity[J]. Oncogene,2004,23 (32):5487-5495.

[4] Liotta LA. Tumor invasion and metastases: role of the basement membran[J]. Am J Pathol,1984, 117(3):339-348.

[5] Tanioka Y, VoshidaT, Yagawa T, et al. Matrix metalloproteinase-7 and matrix metalloproteinase-9 are associated with unfavourable prognosis in superficial oesophageal cancer [J]. British J Cancer,2003,89(11):2116-2121.

[6] 于建渤，殷平，李志强，等. 结肠癌 MMP-9、VEGF 的表达和微血管密度的相关研究[J]. 肿瘤防治研究，2005,32(1):30-32.

[7] Guo H, Zucker S, Gordon MK, et al. Stimulation of matrix metalloproteinase production by recombinant extracellular matrix metalloproteinase inducer from transfected Chinese hamster ovary cells[J]. J Biol Chem,1997, 272(1): 24-27.

[8] Eben L, Shreenivas S, Glenn E, et al. Expression of Extracellular Matrix Metalloprotease Inducer in Laryngeal Squamous Cell Carcinoma[J]. Laryngoscope, 2003, 113(8): 1406-1410.

[9] Tang Y, Nakada MT, Kesavan P, et al. Extracellular Matrix Metalloproteinase Inducer Stimulates Tumor Angiogenesis by Elevating Vascular Endothelial Cell Growth Factor and Matrix Metalloproteinase[J]. Cancer Res,2005,65(8):3193-3199.

[10] Mazzucchelli L. Protein S100A4: too long overlooked by pathologists[J]. Am J Pathol,2002,160 (1):7-13.

[11] Grigorian M, Andresen S, Tulchinsky E, et al. Tumor suppressor p53 protein is a new target for the metastasis-associated Mts1/S100A4 protein: functional consequences of their interaction[J]. J Biol Chem, 2001, 276(25): 22699-22708.

[12] Ikoma N, Yamazaki H, Abe Y, et al. S100A4 expression with reduced E-cadherin expression predicts distant metastasis of human malignant melanoma cell lines in the NODPSCIDPgammaCnull (NOG) mouse model[J]. Oncol Rep,2005,14(3): 633-637.

[13] Gao XN, Tang SQ, Zhang XF, et al. S100A4 antisense oligodeoxynucleotide suppresses invasive potential of neuroblastoma cells[J]. J Pediatr Surg, 2005,40(4):648-652.

[14] Ambartsumian N, Klingelhofer J, Grigorian M, et al. The metastasis associated Mts1(S100A4) protein could act as an angiogenic factor[J]. Oncogene,2001,20(34):4685-4695.

[15] Kimura K, Endo Y, Yonemura Y, et al. Clinical significance of S100A4 and E-cadherin-related adhesion molecules in non-small cell lung cancer[J]. Int J Oncol, 2000, 16 (6): 1125-1131.

[16] Bjornland K, Bratland A, Rugnes E, et al. Expression of matrix metalloproteinases and the metastasis-associated gene S100A4 in humanneuroblastoma and primitive neuroectodermal umor cells[J]. J Pediatr Surg, 2001, 36(7):1040-1044.

[17] Han YP, Tuan TL, Wu H, et al. TNF-alpha stimulates activation of pro-MMP2 in human skin through NF-(kappa)B mediated induction of MT1-MMP[J]. J Cell Sci, 2001, 114(1): 131-139.

[编辑校对：周永红]
