# Supplementary material for: Expression of Concern: The prognostic and clinicopathologic characteristics of CD147 and esophagus cancer: A meta-analysis
Source: PLoS One. 2023 Feb 22;18(2):e0282229. doi: 10.1371/journal.pone.0282229 (PMC9946197; doi:10.1371/journal.pone.0282229)
Supplement: S1 File — (ZIP) [file pone.0282229.s001.zip › PDF of included paper/CD147íó║╦╫¬┬╝╥≥╫╙a╩B...╫┤╧╕░√░⌐╫Θ╓»╓╨╡─▒φ┤∩╝░╥Γ╥σ_╨▄└≥─╚.pdf]

·论著·

CD147、核转录因子κB、表皮生长因子受体在食管鳞状细胞癌组织中的表达及意义

熊莉娜，熊枝繁
(华中科技大学附属梨园医院 消化科，湖北 武汉 430077)

摘　要：目的　研究基质金属蛋白酶刺激因子(CD147)、核转录因子κB(NF-κB(p65))、表皮生长因子受体(EGFR)在食管鳞状细胞癌组织中的表达，探讨其在食管鳞癌侵袭发展中的作用及可能机制。**方法**　应用免疫组织化学方法检测40例食管鳞癌患者癌组织及其癌旁正常对照组织中CD147、NF-κB、EGFR的表达，并以图像分析软件进行相关测定。**结果**　食管鳞癌组织和癌旁正常对照组织中CD147、NF-κB 、EGFR表达阳性率为77.5%(31/40) vs 25.0%(10/40)，87.5%(35/40) vs 42.5%(17/40)，90.0%(36/40) vs 25.0%(10/40)(均 $P<0.01$)。早期和中晚期食管癌表达阳性率在CD147为33.3%(3/9) vs 90.3%(28/31)($P<0.01$)；在NF-κB为55.6%(5/9) vs 96.8%(30/31)($P<0.01$)；在EGFR为77.8%(7/9) vs 93.5%(29/31)($P>0.05$)；CD147、NF-κB、EGFR在食管鳞癌无转移和转移组表达阳性率为28.6%(2/7) vs 87.9%(29/33)，42.8%(3/7) vs 97.0%(32/33)，57.1%(4/7) vs 97.0%(32/33)($P<0.05$或<0.01)。**结论**　CD147、NF-κB、EGFR在食管鳞癌组织中均有高表达，可能与肿瘤的发生发展有关，而且EGFR可能是通过激活CD147和NF-κB途径来实现的。

关键词：食管肿瘤；抗原，CD147；NF-κB；受体，表皮生长因子
中图分类号：R735.1　**文献标识码**：A　**文章编号**：1004-583X(2011)01-0020-04

Significance and expression of CD147 and nuclear factor-κB and epithelial growth factor receptor in esophageal squamous cell carcinoma

XIONG Li-na, XIONG Zhi-fan
Department of Digestive Disease, Liyuan Hospital, Huazhong University of Science & Technology, Wuhan 430077, China
Corresponding author: XIONG Zhi-fan, Email: xiongzhifan@126.com

ABSTRACT: Objective　To investigate the expression of extracellular matrix metalloproteinase inducer(CD147), nuclear factor kappa B(NF-κB), epithelial growth factor receptor(EGFR) in esophageal squamous cell carcinoma tissue; probe their relation to invasion and metabasis of esophageal carcinoma. **Methods**　Immunochemical staining was performed to detect the expression of CD147, NF-κB, EGFR protein in 40 cases of esophageal carcinoma tissues and 40 cases of normal control. **Results**　The positive rate of CD147, NF-κB, EGFR in esophageal carcinoma tissues and adjacent noncancerous tissues were 77.5%(31/40), 87.5%(35/40), 90.0%(36/40) vs 25.0%(10/40), 42.5%(17/40), 25.0%(10/40)(all $P<0.01$). The positive rates of their earlyphase and metaphase-advanced stage esophageal carcinoma tissues were 33.3%(3/9) vs 90.3%(28/31)($P<0.05$) for CD147, 55.6%(5/9) vs 96.8%(30/31) ($P<0.01$) for NF-κB, 77.8%(7/9) vs 93.5%(29/31) for EGFR($P>0.05$). The positive rates of nonmetastatic and metastatic esophageal carcinoma tissues were 28.6%(2/7), 42.8%(3/7), 57.1%(4/7) vs 87.9%(29/33), 97.0%(32/33), 97.0%(32/33)($P<0.05$ or <0.01). **Conclusion**　The results suggest that increased expression of EGFR contributes to tumor angiogenesis in esophageal squamous cell carcinoma, probably through activation of CD147 and NF-κB pathways.

KEY WORDS: esophageal neoplasms; antigens, CD147; NF-kappa B; receptor, epidermal growth factor

食管鳞状细胞癌的恶性程度较高，有临床随机研究证实食管鳞癌术后5年生存率不超过20%[1]，因此早期诊断和及时治疗是提高其生存率的关键。本研究通过检测基质金属蛋白酶刺激因子(CD147)、核转录因子κB(NF-κB(p65))、表皮生长因子受体(EGFR)在食管鳞癌患者癌组织和癌旁正常组织中的表达变化，探讨其在食管鳞癌侵袭和转移过程中的作用，进一步明确食管鳞癌侵袭转移的发生机制，为其早期诊断和开发更多靶向治疗药物提供可能的前期依据和方向。

1　资料与方法

1.1　病例选择　2000～2008年武汉梨园医院外科

通信作者：熊枝繁，Email：xiongzhifan@126.com

(C)1994-2022 China Academic Journal Electronic Publishing House. All rights reserved.　http://www.cnki.net

食管鳞状细胞癌手术患者组织标本40例，以癌中心组织为癌症组，以癌旁正常组织为正常对照组。40例食管癌患者中男26例，年龄41～79岁，平均(56.8±10.2)岁；女14例，年龄35～77岁，平均(59.8±13.3)岁。其中早期食管癌9例，中晚期食管癌31例；无淋巴结转移组7例，有淋巴结转移组33例。所有病例均无糖尿病、糖耐量异常病史，术前均未作放疗、化疗及免疫治疗。所有癌症组织均经病理组织学检测确诊，所有作为正常对照的断端组织均未发现癌细胞。

1.2 方法 免疫组织化学染色方法检测CD147、NF-κB及EGFR的表达，采用链霉亲和素-生物素-过氧化物酶连结法(SABC法)对CD147、NF-κB及EGFR的蛋白表达进行检测。以磷酸盐缓冲液(PBS)代替一抗作为阴性对照，以随试剂所附已知阳性对照片作为阳性对照。

1.3 染色结果观察 使用显微镜及显微摄影仪对染色组织切片进行观察、照相，使用全自动彩色图像分析系统进行图像分析。肿瘤细胞胞膜及胞浆出现棕黄色颗粒为CD147、EGFR阳性表达，肿瘤细胞胞核出现棕黄色颗粒为NF-κB阳性表达，随机观察5个高倍镜视野，计算均值。按肿瘤细胞中阳性比例计算百分率，＜5%为(－)，5%～25%为(＋)，＞25%～50%为(＋＋)，＞50%为(＋＋＋)区分染色强度。最后随机摄取5个高倍镜视野照相。

1.4 统计学方法 应用SPSS 15.0统计软件进行数据处理，所有结果用 χ^2 检验或秩和检验，$P<0.05$ 表示差异有统计学意义。

2 结果

2.1 CD147、NF-κB、EGFR的表达和分布 CD147在绝大部分癌症组标本中可见着色，呈棕黄色颗粒定位于细胞膜和细胞浆中，大部分位于细胞膜，部分癌旁正常对照组标本中也可见着色，散在分布于间质。NF-κB在绝大部分癌症组标本中呈棕黄色颗粒定位于胞核，部分癌旁正常对照组标本中也可见着色。EGFR在绝大部分癌症组标本呈棕黄色颗粒定位于胞膜和胞浆，部分癌旁正常对照组标本中也可见着色。食管鳞癌组织中CD147、NF-κB及EGFR的染色强度明显高于正常对照组，癌组织阳性表达率也显著高于正常组织($P<0.01$)。见表1，图1～3。

表1 CD147、NF-κB、EGFR在食管鳞癌组织、癌旁正常组织中的表达[例(%)]

组别	例数	CD147					NF-κB					EGFR				
		－	＋	＋＋	＋＋＋	阳性合计	－	＋	＋＋	＋＋＋	阳性合计	－	＋	＋＋	＋＋＋	阳性合计
癌症组	40	9(22.5)	7(17.5)	9(22.5)	15(37.5)	31(77.5)	5(12.5)	8(20.0)	10(25.0)	17(42.5)	35(87.5)	4(10.0)	9(22.5)	10(25.0)	17(42.5)	36(90.0)
对照组	40	30(75.0)	6(15.0)	3(7.5)	1(2.5)	10(25.0)	23(57.5)	12(30.0)	3(7.5)	2(5.0)	17(42.5)	30(75.0)	7(17.5)	2(5.0)	1(2.5)	10(25.0)
u_c 值		5.116					5.176					6.210				
P 值		＜0.01					＜0.01					＜0.01				

2.2 CD147、NF-κB、EGFR的表达和临床病理的关系 CD147、NF-κB在早期食管癌表达阳性率均低于中晚期食管癌，差异有统计学意义($P<0.01$)。EGFR在早期食管癌表达阳性率低于中晚期食管癌，但差异无统计学意义($P>0.05$)。依据手术切除后局部淋巴结转移情况，CD147、NF-κB和EGFR在无转移组表达阳性率均低于有转移组，差异有统计学意义($P<0.05$或<0.01)。见表2。

表2 CD147、NF-κB、EGFR在食管鳞癌组织中的表达和浸润深度、淋巴结转移的关系[例(%)]

浸润深度	例数	CD147	NF-κB	EGFR
早期食管癌	9	3(33.3)	5(55.6)	7(77.8)
中晚期食管癌	31	28(90.3)	30(96.8)	29(93.5)
χ^2 值		9.928	7.394	1.928
P 值		＜0.01	＜0.01	＞0.05
**淋巴结转移**	**例数**	**CD147**	**NF-κB**	**EGFR**
无	7	2(28.6)	3(42.8)	4(57.1)
有	33	29(87.9)	32(97.0)	32(97.0)
χ^2 值		8.496	10.909	6.234
P 值		＜0.01	＜0.01	＜0.05

3 讨论

CD147是相对分子质量为50 000～60 000广泛存在于人体各个组织器官的免疫球蛋白超家族的跨膜糖蛋白[2]，通过细胞-细胞及细胞-间质黏附作用参与机体多种生理过程[3]，并降解细胞外基质中的各种蛋白来调节肿瘤细胞的各种生物学行为如侵袭和转移等。目前国内外不少研究者发现CD147在结直肠癌、口腔鳞状细胞癌、皮肤鳞状细胞癌、泌尿系肿瘤、肺癌、乳腺癌、子宫内膜癌、黑色素瘤、骨髓癌、神经胶质瘤等肿瘤中均增高，相对于正常组织，在癌组织中其mRNA表达增高，并与肿瘤的浸润转移相关。本研究观察到CD147的表达阳性率在食管鳞癌组中高于正常对照组，中晚期组中高于早期组，有转移组中高于无转移组，且差异有统计学意义。因此，将CD147作为靶向分子，设计、筛选并合成它的肽类拮抗剂，可能对防止患者术后肿瘤的转移和复发，提高术后生存率有一定帮助。

(C)1994-2022 China Academic Journal Electronic Publishing House. All rights reserved. http://www.cnki.net

NF-κB广泛存在于各种细胞中，是重要的转录调控因子，与免疫应激、细胞增殖、生长分化、细胞周期及凋亡有密切联系[4]，其与肿瘤的发生、发展，尤其是与肿瘤的浸润转移、组织学分级以及肿瘤耐药问题也密切相关[5]。本研究观察到NF-κB的表达阳性率在癌症组中高于正常对照组，中晚期组中高于早期组，有转移组中高于无转移组，其差异均有统计学意义，CD147能通过促进血管内皮细胞生长因子(VEGF)的表达促进肿瘤血管生成或增加透明质酸的表达共同促进肿瘤的发展，而NF-κB的抑制信号能有效的抑制高转移性前列腺癌PC23细胞的VEGF、白细胞介素8(IL-8)、基质金属蛋白酶9(MMP-9)3种主要前血管生成分子的表达而减少肿瘤血管生成，抑制肿瘤的侵袭转移，因此考虑CD147和NF-κB可能有共同的作用途径来促进肿瘤的发展。

EGFR属于酪氨酸激酶受体家族，当与其配体表皮生长因子(EGF)结合后，可启动细胞生长周期，使受体的酪氨酸蛋白激酶被激活，将生长信号传导至核内，导致DNA合成增加，刺激细胞生长和增殖[6]。EGFR所介导的信号传导通路与肿瘤的发生、发展关系密切[7]。本研究显示，EGFR的表达阳性率在癌症组中高于正常对照组，有转移组中高于无转移组，其差异有统计学意义；中晚期组中高于早期组，但差异无统计学意义，可能与肿瘤浸润深浅无关。EGFR可通过酪氨酸蛋白激酶的激活诱导CD147表达，表明EGFR信号通路在此调节中的重要作用，EGFR和VEGF通过共同的信号转导通路表达NF-κB。EGFR可能是通过激活CD147及NF-κB途径来实现肿瘤表达的。

综上所述，CD147、NF-κB、EGFR对食管鳞癌的诊断有一定帮助，且可为食管鳞癌的靶向治疗提供新的靶点。(本文图见封三)

参考文献：

[1] Mariette C, Finzi L, Fabre S, et al. Factors predictive of complete resection of operable esophageal cancer: a prospective study[J]. Ann Thorac Surg, 2003, 75(6): 1720-1726.

[2] Wang B, Xu YF, He BS, et al. RNAi-mediated silencing of CD147 inhibits tumor cell proliferation, invasion and increases chemosensitivity to cisplatin in SGC7901 cells in vitro[J]. J Exp Clin Cancer Res, 2010, 29: 61.

[3] Yurchenko V, Constant S, Eisenmesser E, et al. Cyclophilin-CD147 interactions: a new target for anti-inflammatory therapeutics[J]. Clin Exp Immunol, 2010, 160(3): 305-317.

[4] Luqman S, Pezzuto JM. NF kappaB: a promising target for natural products in cancer chemoprevention[J]. Phytother Res, 2010, 24(7): 949-963.

[5] Gupta SC, Prasad S, Reuter S, et al. Modification of cysteine 179 of IkappaBalpha kinase by nimbolide leads to down-regulation of NF-kappaB-regulated cell survival and proliferative proteins and sensitization of tumor cells to chemotherapeutic agents[J]. J Biol Chem, 2010, 285(46): 35406-35417.

[6] Ray RM, Bhattacharya S, Johnson LR. EGFR plays a pivotal role in the regulation of polyamine-dependent apoptosis in intestinal epithelial cells[J]. Cell Signal, 2007 19(12): 2519-2527.

[7] Pu J, McCaig CD, Cao L, et al. EGF receptor signalling is essential for electric-field-directed migration of breast cancer cells[J]. J Cell Sci, 2007, 120(Pt 19): 3395-3403.

收稿日期：2010-08-30　修回日期：2010-11-15　编辑：张卫国

(上接第19页)

本研究显示几乎所有部位的斑块无论其大小、性质和数量多少AP均有不同程度增高，提示有症状的颈动脉斑块都可导致轻度不一的脑缺血发生，但不一定有脑损害的定位体征和影像等的改变，而斑块的数量与LPA、AP水平变化无关，提示斑块无论数目多少只要有斑块存在脑缺血就已发生，卒中的危险性也相对增加。在此其及早进行临床干预，为防治缺血性脑卒中的发生有一定临床意义。

参考文献：

[1] Crouse JR 3rd, Grobbee DE, O'Leary DH, et al. Carotid intima-media thickness in low-risk individuals with asymptomatic atherosclerosis: baseline data from the METEOR study[J]. Curr Med Res Opin, 2007, 23(3): 641-648.

[2] 袁力.颈动脉粥样硬化与脑梗死[J].实用心脑肺血管病杂志, 2006, 14(6): 502-503.

[3] Chung SM, Bae ON, Lim KM, et al. Lysophosphatidic acid induecs thrombogenic ctivity through phosphatidylserine exposure and procoagulant microvesicle generation in human erythrocytes[J]. Arterioscler Thromb Vasc Biol, 2007, 27(2): 414-421.

[4] 李振光, 伍期专, 唐朝枢, 等.动脉粥样硬化患者颈动脉内膜中层厚度与血浆溶血磷脂酸含量的变化[J].中华医学杂志, 2004, 84(13): 1071-1072.

[5] 李霞, 周丽, 宋春雨.急性缺血性脑血管病患者血浆溶血磷脂酸和总磷脂水平的变化[J].脑与神经疾病杂志, 2009, 17(5): 394-395.

[6] Adibhatla RM, Hatcher JF. Citicoline decreases phospholijase Az stimulation and hydroxylradical generation in transient cerebral ischemia[J]. J Neurosci Res, 2003, 73(3): 308-315.

[7] Siess W, Zhangl KJ, Essler M, et al. Lysophosphatidic acid mediates the rapid activation of platelets and endothelial cells by mildly oxidized low density lipoprotein and accumulates in human atherosclerotic lesions[J]. Proc Natl Acad Sci USA, 1999, 96(7): 6931-6936.

[8] 伍丽红, 王继阳, 程立山, 等.应用颈动脉超声分析缺血性脑血管病危险因素与颈动脉斑块的关系[J].中国脑血管病杂志, 2006, 3(1): 22-23.

[9] 宁彬, 何文, 项东英, 等.颈动脉狭窄超声检查的应用及进展[J].中国卒中杂志, 2007, 2(1): 74-75.

收稿日期：2010-09-21　修回日期：2010-11-17　编辑：张卫国

(C)1994-2022 China Academic Journal Electronic Publishing House. All rights reserved. http://www.cnki.net

CD147、NF-κB、EGFR 在食管鳞状细胞癌组织中的表达及意义

(正文见第20页)

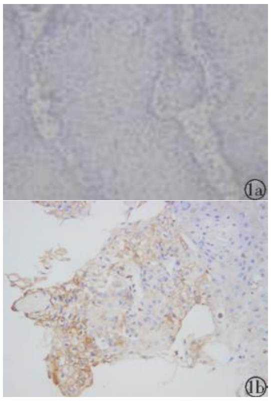

a:癌旁正常对照组(SABC法×200);
b:癌症组(SABC法×400)
图1 CD147的表达

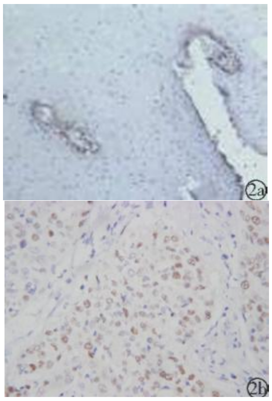

a:癌旁正常对照组(SABC法×200);
b:癌症组(SABC法×400)
图2 NF-κB的表达

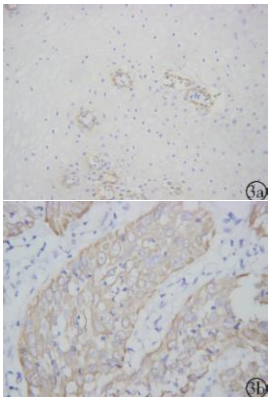

a:癌旁正常对照组(SABC法×200);
b:癌症组(SABC法×400)
图3 EGFR的表达

结节性甲状腺肿伴腺瘤样增生的超声诊断与病理分析

(正文见第34页)

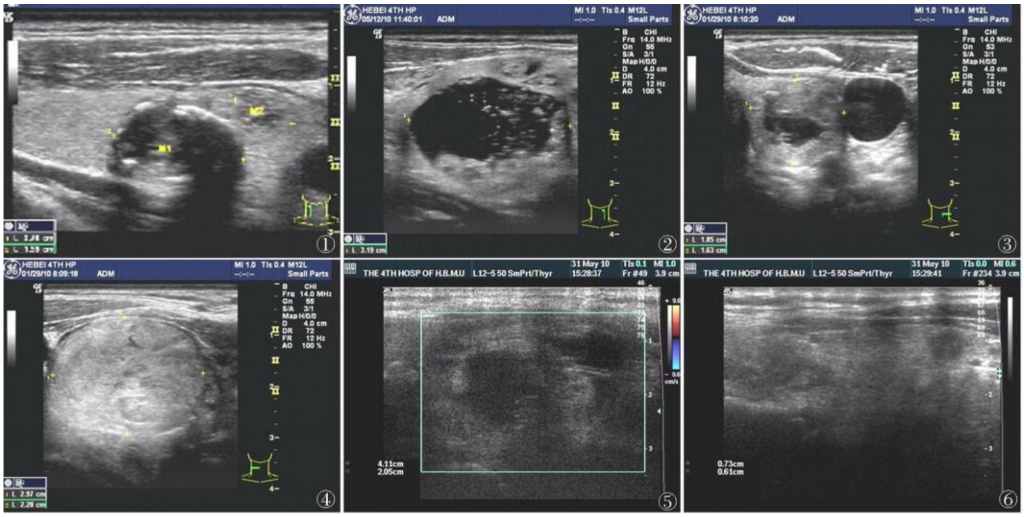

图1 右叶结节性甲状腺肿伴腺瘤样增生粗大钙化斑;图2 左叶结节性甲状腺肿伴腺瘤样增生囊性变;图3 左叶结节性甲状腺肿伴腺瘤样增囊性变;图4 右叶甲状腺腺瘤;图5 右叶结节性甲状腺肿伴腺瘤样增生;图6 左叶甲状腺乳头状腺癌

育龄妇女颅内静脉窦血栓的临床分析

(正文见第49页)

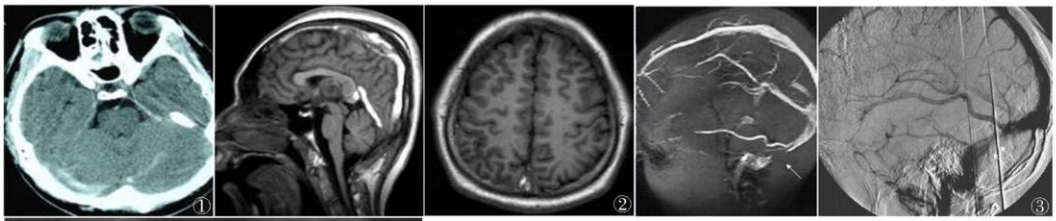

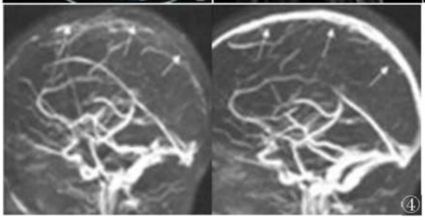

右侧横窦束带征

图1 头CT检查

T_1WI见上矢状窦高信号,为亚急性期,血管内正铁血红蛋白形成

图2 头MRI检查

图3 头MRV与DSA对照,左横窦充盈缺损(箭头处),引流静脉迂曲扩张

上矢状窦未显影,血流信号消失,髓静脉扩张。治疗后上矢状窦显影,髓静脉走形正常

图4 MRV检查

(C)1994-2022 China Academic Journal Electronic Publishing House. All rights reserved. http://www.cnki.net
