# Supplementary material for: Expression of Concern: The prognostic and clinicopathologic characteristics of CD147 and esophagus cancer: A meta-analysis
Source: PLoS One. 2023 Feb 22;18(2):e0282229. doi: 10.1371/journal.pone.0282229 (PMC9946197; doi:10.1371/journal.pone.0282229)
Supplement: S1 File — (ZIP) [file pone.0282229.s001.zip › PDF of included paper/CD147║═ MMP-2╘┌╘τ╞┌╩│╣▄░⌐╓╨╡─▒φ┤∩.pdf]

CD147和MMP-2在早期食管癌中的表达

刘海明 窦俊峰

(河南省卫生学校附属医院 河南安阳 455000)

【摘要】目的 探讨CD147和MMP-2在食管癌发生过程中的作用及其相关性。方法 应用免疫组织化学S-P法检测CD147和MMP-2在19例食管早期癌和20例正常食管黏膜标本中的表达。结果 CD147和MMP-2在早期癌中的表达均高于正常食管黏膜($P<0.05$)。结论 CD147和MMP-2可能与食管癌的发生有关,他们的变化可能是食管癌发生中的早期事件。

【关键词】p21 p16 食管早期癌 免疫组织化学

【中图分类号】R735 【文献标识码】A 【文章编号】1674-0742(2010)10(b)-0003-02

The Expression of CD147 and MMP-2 in Human Early Esophageal Carcinoma

LIU Haiming DOU Junfeng

The Affiliated Hospital of Henan Medical School,Henan 455000,China

【Abstract】Objective To study the effect of CD147 and MMP-2 in esophageal carcinogenesis and their relationship.Methods Immunohistochemical S-P method was used to detect the expressions of CD147 and MMP-2 in 19 cases of esophageal early carcinoma and 20 cases of normal esophageal mucous.Results The expression of CD147 and MMP-2 were both higher in early esophageal carcinoma than in normal esophageal mucous($P<0.05$).Conclusion CD147 and MMP-2 are probably related to esophageal carcinogenesis,and their changes are early events in esophageal carcinogenesis.

【Key Words】P21;p16;Esophageal Early Carcinoma;Immunohistochemistry

我国是食管癌的高发国,由于患者到医院就诊时大多已经是中晚期,治疗效果较差,而早期癌治疗后,5年存活率>90%。所以,要想大幅提高食管癌患者的预后,关键在于早期发现。本实验拟通过对早期食管癌中CD147和MMP-2表达的检测,探讨两者在食管癌发生中的作用及其相互关系。

1 材料与方法

1.1 材料

19例食管早期癌和20例正常食管黏膜标本均为河南省医学科学研究所2001年至2004年对林州市食管癌高危人群普查内镜活检标本。其中男性20例,女性19例。组织学类型:早期癌均为鳞癌。所有标本经10%甲醛固定,石蜡包埋切片;抗体及免疫组化试剂盒均购自北京中杉公司。

1.2 方法

免疫组织化学S-P法:切片常规脱蜡水化,高压抗原热修复,其余步骤按试剂说明书上进行。CD147和MMP-2的抗体稀释度均为1:100,用已知阳性切片作为阳性对照,用PBS代替一抗做阴性对照。

1.3 结果判定

细胞核或细胞浆内见均匀一致的棕黄色颗粒为阳性,根据阳性细胞的数量进行结果判定:阳性细胞数<10%为阴性(-),阳性细胞数>10%为阳性(+)。

1.4 统计学处理

采用Fisher's精确概率检验,检验水准$\alpha=0.05$。

2 结果

CD147和MMP-2阳性反应主要位于细胞浆和细胞膜。CD147在早期癌和正常食管黏膜中的阳性率分别为63.2%(12/19)和15%(3/20),CD147在早期癌中与正常食管黏膜相比表达增高($P<0.05$);MMP-2在早期癌和正常食管黏膜中的阳性率分别为57.9%(11/19)和10%(2/20),MMP-2在早期癌中与正常食管黏膜相比表达增高($P<0.05$)。对CD147和MMP-2在早期癌中表达的相关性分析,$P<0.05$,表明两者的表达之间存在相关性。

3 讨论

最近研究发现[1],CD147可以诱导肿瘤细胞周围的纤维母细胞产生多种MMP,从而影响肿瘤的生物学行为和临床进展过程。CD147可能参与了肿瘤的发生和转移过程[2]。肿瘤间质中的纤维母细胞能分泌多种基质金属蛋白酶(matrix metalloproteinases,MMP)在肿瘤细胞的生长、存活、侵袭、血管新生和转移中起重要作用。有关肿瘤组织合成MMPs的机制尚不清楚。MMPs是一个含锌离子的肽链内切酶家族[3],MMP-2和MMP-9是主要成员,它们能分解基底膜中的纤维连接蛋白和层黏连蛋白,降解细胞外基质。近年来的研究表明MMP-2或MMP-9的高表达与多种肿瘤的生物学行为改变和临床进展有关。

本实验结果显示,CD147和MMP-2在早期癌和正常食管粘膜相比表达增高,提示这两个基因可能参与了食管癌的发生过程,可能和其他相关基因一起,共同导致了食管粘膜的癌变。另外,本实验结果显示CD147和MMP-2在食管早期癌中的表达存在相关性,这和之前的研究相一致,印证了两者的表达之间存在者相互作用,而两者之间具体的作用机制,还有待于进一步深入研究。

(下转5页)

应，不仅术前会产生焦虑抑郁等心理，术后仍有较高水平的负性情绪。由于应对心理应激能力的降低．患者角色的习惯化等，50%的患者容易对家属和医护人员产生依赖心理；部分患者由于经济状况和文化水平低而产生无奈心理。患者术后患肢需平伸制动，生活不能自理，使患者感到自己成为亲人负担，社会和家庭地位的改变，使其自尊心受到伤害，从而感到自卑[3]。

人作为生物–心理–社会的整体，心理因素已成为影响身心健康不容忽视的因素之一，对心脏介入诊疗术患者采用一定的心理干预，在一定程度上改善或消除了患者的焦虑、抑郁情绪[4]。本研究结果亦表明，对患者进行心理支持干预后患者的焦虑、抑郁情况有一定程度的改善。坦度螺酮属新上市的非苯二氮䓬类抗焦虑药物，不论是对功能性焦虑还是躯体疾病伴发的焦虑，坦度螺酮都有较好的疗效。本研究结果亦显示：治疗前后患者的焦虑，抑郁症状有明显改善，但由于该药出现明显疗效较慢，约1～2周，长者达4周[5]，因此本研究显示：对心脏介入围手术期焦虑，抑郁症状的改善无稳心颗粒组显著。

焦虑症属中医“郁证”范畴，与肝脾的功能失调有关。“思伤脾”、“怒伤肝”，情志失调，致肝气郁结，脾胃失健，气血生化乏源，心失所养，心神不宁，则见失眠、心烦、易怒、纳差等症。

纵观其病因病机，阴阳气血失调是其本，七情损伤是其标，其中肝郁血虚则是病的根本所在[6]。步长稳心颗粒冲剂由党参、黄精、三七、琥珀、甘松组成，其中党参、黄精性甘平，益气养阴、健脾化源；三七、甘松性温理气化瘀、开郁醒脾；琥珀性甘平，宁心护脉、活血利水；研究表明稳心颗粒具有益气养阴、宁心复脉、活血化瘀、定悸安神功效。本着“异病同治”、“治病求本”的辩证思想，本研究采用稳心颗粒治疗心脏起搏器安置术患者的焦虑症，显示出较好的临床疗效。

参考文献

[1] Holt-.Lunstad J,Birmingham W,Jones BQ.Is there something unique about marriage?The relative impact of marital status,relationship quality,and net-work social support on ambulatory blood pressure and mental health[J].Ann Be hay Med,2006,35:45～51.

[2] Chaput JP,Arguin H,Gagnon C.Increase in depression symptonm with weight loss:association with gluc0se homeostasis and thyroid function[J].Appl Physiol Nutr Metab,2007,33:86～92.

[3] 刘熔雪.心脏介入诊疗术中患者心理应激及干预[J].中国医药导报，2008,5(33):124.

[4] 齐丽雯，孙玉香.心理干预对减轻心脏介入患者术前焦虑的效果观察[J].中国误诊学杂志，2006,6(6):1066～1067.

[5] 房茂胜，赵靖平.抗焦虑药物的合理应用[J].中国处方药，2007,62(5):49～51.

[6] 闫炳远.中医药结合心理疗法治疗焦虑症[J].浙江中西医结合杂志，2009,19(1):24～25.

【收稿日期】2010-07-28

(上接3页)

参考文献

[1] Yu W,Liu J,Xiong X,et al.Expression of MMpq and CD147 in invasive squamous cell carcinoma of the uterine cervix and their implication[J].Pathol Res Pract,2009,205(10):709～715.

[2] Han ZD,He HC,Bi XC,et al.Expression and clinical significance of CD147 in genitourinary carcinomas.[J].J Surg Res,2010May 15,160(2):260～267.

[3] Zucker S,Hymowitz M,Rollo EE,et al.Tumorigenic potential of extracellular matrix metalloproteinase inducer[J].Am J Pathol,2001,158(6):1921～1928.

【收稿日期】2010-06-07
