# Supplementary material for: Expression of Concern: The prognostic and clinicopathologic characteristics of CD147 and esophagus cancer: A meta-analysis
Source: PLoS One. 2023 Feb 22;18(2):e0282229. doi: 10.1371/journal.pone.0282229 (PMC9946197; doi:10.1371/journal.pone.0282229)
Supplement: S1 File — (ZIP) [file pone.0282229.s001.zip › PDF of included paper/Cancer - 2004 - Ishibashi - CD147 and matrix metalloproteinase⌐/2 protein expression as significant prognostic factors in.pdf]

CD147 and Matrix Metalloproteinase-2 Protein Expression as Significant Prognostic Factors in Esophageal Squamous Cell Carcinoma

Yoshio Ishibashi, M.D.[1]
Tomoe Matsumoto[2]
Mikio Niwa[2]
Yutaka Suzuki, M.D.[1]
Nobuo Omura, M.D.[1]
Nobuyoshi Hanyu, M.D.[1]
Koji Nakada, M.D.[1]
Katsuhiko Yanaga, M.D.[1]
Kyosuke Yamada, M.D.[3,4]
Kiyoshi Ohkawa, M.D.[4]
Makio Kawakami, M.D.[5]
Mitsuyoshi Urashima, M.D., M.P.H.[6]

[1] Department of Surgery, Jikei University School of Medicine, Tokyo, Japan.

[2] Toagosei, Co., Ltd., Ibaraki, Japan.

[3] Department of Obstetrics and Gynecology, Jikei University School of Medicine, Tokyo, Japan.

[4] Department of Biochemistry, Jikei University School of Medicine, Tokyo, Japan.

[5] Department of Pathology, Clinical Service, Jikei University School of Medicine, Tokyo, Japan.

[6] Division of Clinical Research and Development, Jikei University School of Medicine, Tokyo, Japan.

Supported by a Grant in Aid for Scientific Research (Japan).

The authors thank Ms. Michiko Kasai, Ms. Naoko Ikeda, and Ms. Ayumi Saito for their technical help with immunohistochemical staining.

Address for reprints: Mitsuyoshi Urashima, M.D., Ph.D., M.P.H., Division of Clinical Research and Development, Jikei University School of Medicine, Nishi-shimbashi 3-25-8, Minato-ku, Tokyo 105-8461, Japan; Fax: (011) 81-3-5400-1250; E-mail: urashima@jikei.ac.jp

Received April 13, 2004; revision received July 8, 2004; accepted July 15, 2004.

BACKGROUND. The authors investigated whether the presence of matrix metalloproteinase-2 (MMP-2) and its inducer, CD147, in cancerous esophageal lesions and surrounding tissue might help to predict patient prognosis.

METHODS. Tissue samples from 101 patients with esophageal squamous cell carcinoma were stained with anti-CD147 and anti–MMP-2 antibodies for immunohistochemical analysis.

RESULTS. CD147 was expressed in cancerous and dysplastic lesions, but not in normal tissue. In contrast, MMP-2 was detected mainly in normal interstitial tissue adjacent to cancerous lesions, but it was detected also in cancerous lesions in some patients. Pathologic findings demonstrated that the intensity of MMP-2 staining in normal tissue was associated positively with the depth of tumor infiltration and the stage of disease, whereas MMP-2 staining in cancerous tissue was associated positively with vascular and lymphatic vessel invasion as well as with immature differentiation of cancer cells. Using a proportional hazard model, including information on CD147 staining patterns within cancerous lesions along with clinical cancer staging, improved the accuracy of predicting patient prognosis.

CONCLUSIONS. These results suggested that measurement of CD147 and MMP-2 expression with simple immunohistochemical staining may enhance further the understanding of the pathophysiology of invading tumor cells and, when used in combination with cancer staging, may increase the ability of investigators to predict prognosis in patients with esophageal squamous cell carcinoma. ***Cancer* 2004;101:1994–2000.** *© 2004 American Cancer Society.*

KEYWORDS: CD147, metalloproteinase, esophagus, cancer, prognosis.

Interactions between cancer cells and the surrounding microenvironment may allow tumor invasion into adjacent organs and trigger metastasis via vascular/lymphatic vessels.[1] Results of investigations into tumor biology can contribute to the development of novel promising therapies, such as protease inhibitors for myeloma treatment.[2] CD147, also known as extracellular matrix (ECM) metalloproteinase inducer (EMMPRIN) or basigin, is highly expressed on the outer surface of carcinoma cells, but not on normal mucosal cells. CD147 stimulates adjacent interstitial normal cells to produce matrix metalloproteinases (MMPs).[3] MMPs are proteases known to degrade the ECM.[4] Thus, carcinoma cells can interact with adjacent normal cells to produce MMPs via CD147 on their surface, and, in turn, invade lymphatic tissue and blood vessels and penetrate through the ECM to adjacent organs with the help of MMPs.[5]

The roles of CD147 and MMPs in tumor invasiveness have been confirmed immunohistochemically in several types of cancer cells

© 2004 American Cancer Society
DOI 10.1002/cncr.20593
Published online 15 September 2004 in Wiley InterScience (www.interscience.wiley.com).

and surrounding tissue, including astrocytomas[6] and melanomas.[7] Moreover, the expression of MMPs is reported to correlate with the clinical prognosis of patients with breast carcinoma[8] and other types of cancers.[9–11]

Esophageal squamous cell carcinoma is an aggressive cancer with a poor prognosis. The median survival ranges from 1 to 2 years even with chemoradiotherapy before surgery,[12,13] although survival has continued to improve.[14] Currently, clinical TNM staging before treatment is the best way to predict the prognosis of patients with esophageal carcinoma.[15] However, the combination of immunohistochemical examinations with ordinal TNM staging has been reported to improve diagnostic accuracy in terms of predicting prognosis.[16,17] In addition, recent progress in molecular diagnostics using gene expression profiles may help to predict the prognosis of patients with esophageal carcinoma[18] and other types of cancer.[19,20]

In the current study, CD147 and MMP protein expression patterns within esophageal cancer cells and surrounding tissue were examined immunohistochemically to determine their relation to clinicopathologic findings and disease recurrence-free survival of patients.

MATERIALS AND METHODS

Patients

Between February 1994 and December 2001, 117 patients with esophageal tumors were treated at Jikei University Hospital (Tokyo, Japan). Of the 117 patients, 16 were excluded (4 patients did not have paraffin-embedded specimens available, 6 patients displayed adenocarcinoma, and 6 patients had carcinoma in situ [CIS]). Therefore, 101 patients for whom specimens and clinical information could be obtained were included in the current study.

Clinical information was abstracted from surgical and clinical charts. Some patients received radiotherapy to improve quality of life. Tumor stages were classified according to the 5th edition of the TNM classification system.[21] Patients were periodically (every 1–3 months) examined on an outpatient basis to make sure they did not have disease recurrence. Examinations consisted of standard tests, including endoscopy and computed tomography scans of the chest and abdomen.

Pathologic Specimens

Specimens were obtained by endoscopic mucosal resection or surgery. Formalin-fixed, paraffin-embedded specimens of esophageal tumors were processed for conventional histologic assessment by hematoxylin and eosin (H & E) staining. Only patients with squamous cell carcinoma of the esophagus confirmed by two or more board-certified pathologists were included. Patients with all other forms of carcinoma, including partly adenomatous lesions, were excluded. All specimens were free of cancer invasion of the tumor margin and patients with CIS were excluded. Histologic features of the extent of the lesions, invasion into lymphatic or blood vessels, intramural metastasis, and lymph node metastasis were evaluated. Pathologic diagnosis and classification of specimens were accomplished using the guidelines of the Japanese Society for Esophageal Diseases.[22] Lymphatic vessel invasion was determined to be definite when cancer cells were detected in thin-walled, endothelium-lined spaces containing no red blood cells (RBC) and occasional lymph fluid. Similarly, blood vessel invasion was defined by the presence of cancer cells and RBC within round or ovoid endothelium-lined spaces surrounded by a layer of smooth muscle.[23–25] Intramural metastasis was defined as evidence of tumor within the esophageal wall, not directly related to the primary tumor.[26,27] Histologic grades of differentiation were assigned according to whether a tumor was well, moderately, or poorly differentiated.[28]

Establishment of Murine Anti-CD147 Antibody

A murine monoclonal antibody (MoAb 12C3) that is specific to human ovarian carcinoma was generated by immunizing mice with a human ovarian germinoma cell line (JOHYC-2).[29] To recognize the antigen epitope defined by MoAb 12C3, a cDNA library was constructed from the SKOV3 human ovarian carcinoma cell line using a T7Select 10-3 vector (Novagen, Madison, WI). The phages were bound to microplate wells (Immulon-2, Dynatec Laboratory, Chantilly, VA), which were immobilized with MoAb 12C3, and were selected from either the SKOV3 cDNA or the colon carcinoma cDNA library (Novagen) by the panning method. Furthermore, a single-phage clone was purified by immunostaining with MoAb 12C3 from the SKOV3 and colon carcinoma cDNA libraries. These single-phase clones were termed SKOV3-1 and Colon-1, respectively. Then, the DNA sequences of the phage selected were determined by dideoxynucleotide chain termination. A homology search using amino acid sequence with the BLAST program was performed and revealed that SKOV3-1 and Colon-1 matched with the 17th to 174th and the 17th to 149th amino acid residues, respectively, which were on the extracellular region of CD147 (Fig. 1).

Immunohistochemical Staining for CD147 and MMP-2

Using paraffin-embedded specimens from patients with esophageal squamous cell carcinoma, CD147

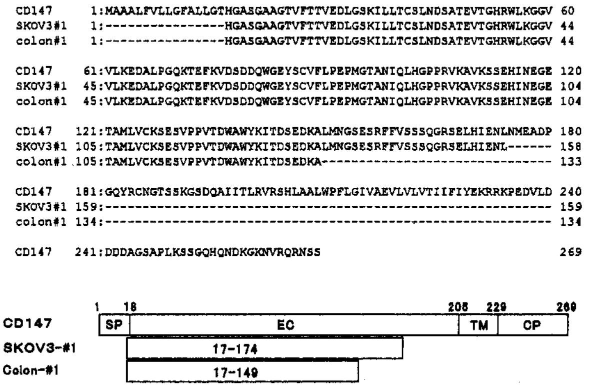

FIGURE 1. Single-phage clone purified by immunostaining with monoclonal antibody 12C3 from the SKOV3 and colon carcinoma cDNA libraries, termed SKOV3-1 and Colon-1, respectively. A homology search using an amino acid sequence with the BLAST program was performed and revealed that SKOV3-1 and Colon-1 matched the 17th to the 174th and the 17th to the 149th amino acid residues, respectively, which were on the extracellular region of CD147. SP: signal peptide region; EC: extracellular region; TM: transmembrane region; CP: cytoplasmic region. The numbers within the rectangles indicate amino acid residues encoded by isolated clones.

protein was detected using the anti-CD147 murine MoAb using a dextran polymer conjugate two-step visualization system.[30] Using different slices from the same paraffin-embedded specimens, MMP-2 protein was stained with anti-human MMP-2 murine MoAb and with purified immunoglbulin G (F68; Daiichi Fine Chemical Co., Ltd., Toyama, Japan).[31] Results of staining for CD147 in both cancerous and dysplastic lesions were classified into the following three patterns: no staining, partial staining, and diffuse and strong staining. Results of staining for MMP-2 both in normal tissue adjacent to cancerous lesions and in cancerous lesions were classified into just negative or positive. The examiners were blinded to patients' clinicohistologic (H & E staining) information when assigning staining patterns as positive/negative. Two investigators (Y.I. and M.U.) evaluated the staining levels independently, after which discordant evaluations were adjusted by connected microscopes.

Statistical Analysis

Kappa statistical analyses were applied to measure agreements of CD147 immunohistochemical staining between dysplastic and cancerous lesions. Chi-square tests and simple linear regression analysis were used to evaluate the relation between immunohistochemical staining patterns and several clinicopathologic parameters. Survival curves of the patients were compared using the Kaplan–Meier method and analyzed by the log-rank test. Cox proportional hazards models were fitted for multivariate analysis.

RESULTS

Patients' age ranged from 43 to 83 years (mean ± standard deviation: 61 ± 9 years) and there were more men (n = 69) than women (n = 32). All patients were followed from Day 16 after surgery to a maximum of 3175 days (median, 981 days). Forty-seven patients died of esophageal carcinoma and six of other causes. Thus, 48 patients were alive and 53 had died. Three patients were lost to follow-up and included in the current study as censored on the final day in the outpatient clinic.

Immunohistochemistry for CD147 and MMP-2 Protein Expression Patterns in Patients with Esophageal Lesions

Typical histologic pictures of CD147 staining of cancerous and dysplastic tissue are shown in Figure 2A–C. CD147 was enriched on the surface of cancer cells. Forty-six cancerous lesions stained with the CD147 antibody showed a diffuse and strong pattern, whereas 24 showed a partial pattern and 31 showed no staining. Conversely, in dysplastic tissue, staining with the CD147 antibody was negative in 45 lesions, partial in 38 lesions, and diffuse and strong in 18 lesions. In 45 specimens, CD147 expression patterns were matched between cancerous and dysplastic lesions (chi-square test: $P < 0.001$). The other 56 specimens did not exhibit any matching between cancerous and dysplastic lesions (kappa = 0.20, 95% confidence interval, 0.08–0.32). Neither normal esophageal mucosal tissue nor submucosal interstitial cells were stained with the anti-CD147 antibody.

Submucosal interstitial cells around cancerous lesions expressed MMP-2 protein (Fig. 2D). The frequency for each staining was as follows: negative, 24 specimens; positive, 77 specimens. In contrast, submucosal interstitial cells distant from cancerous tissue were not stained with the anti–MMP-2 antibody. Moreover, cancerous tissue was stained with the anti–MMP-2 antibody in 25 patients (Fig. 2E), which showed also positive MMP-2 staining in interstitial cells. However, the expression of CD147 in cancerous or dysplastic lesions had no association with the expression of MMP-2 on submucosal or cancerous lesions.

CD147 and MMP-2 Expression Patterns and Clinicopathologic Variables

CD147 protein expression patterns within cancerous and dysplastic lesions were not associated with any of the following variables: TNM classification, stage, extent of the lesions, invasion into lymphatic or blood vessels, intramural metastasis, or histologic grades of differentiation.

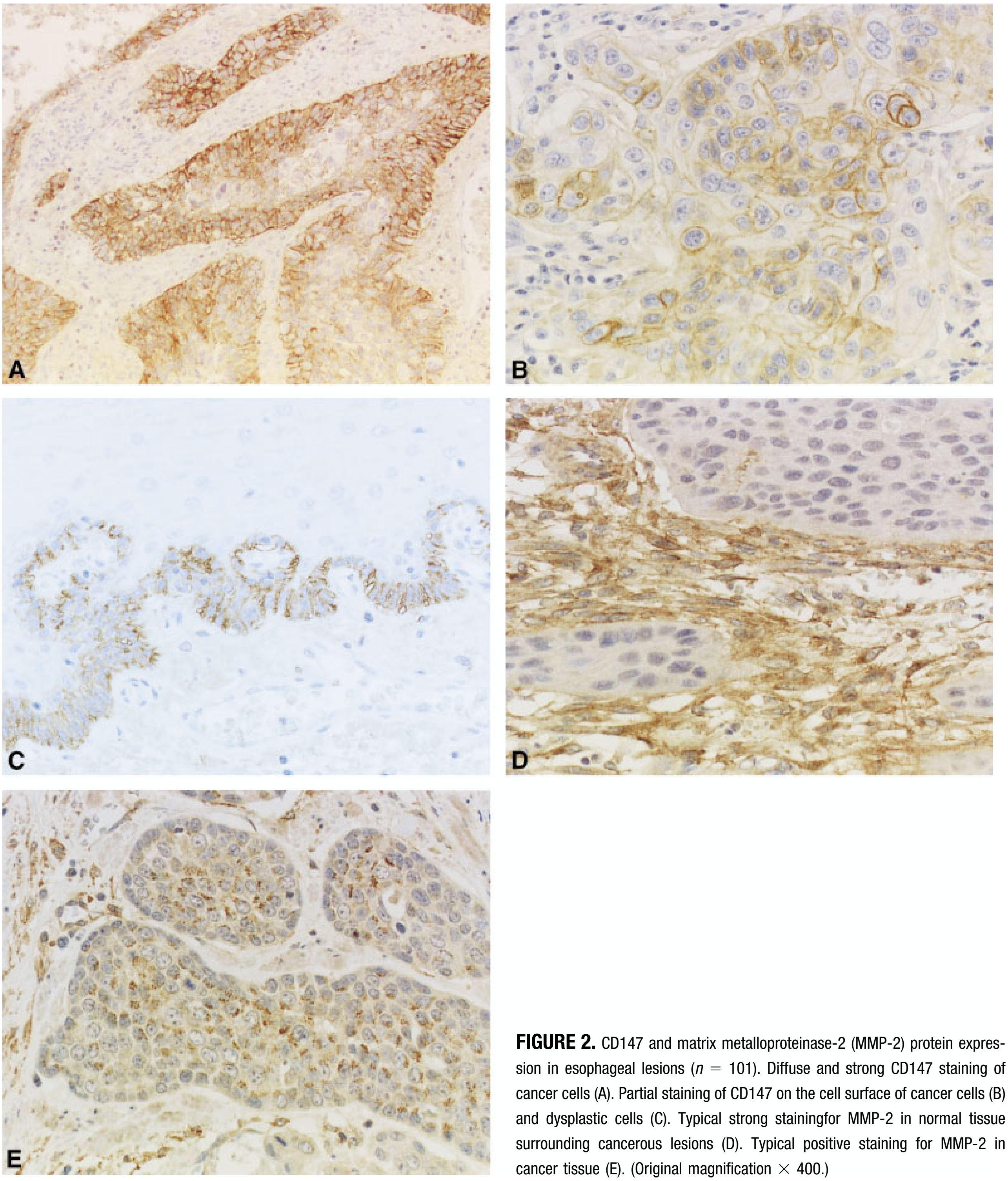

FIGURE 2. CD147 and matrix metalloproteinase-2 (MMP-2) protein expression in esophageal lesions ($n = 101$). Diffuse and strong CD147 staining of cancer cells (A). Partial staining of CD147 on the cell surface of cancer cells (B) and dysplastic cells (C). Typical strong stainingfor MMP-2 in normal tissue surrounding cancerous lesions (D). Typical positive staining for MMP-2 in cancer tissue (E). (Original magnification × 400.)

The presence of MMP-2 protein within the normal tissue around cancerous lesions was associated positively with tumor infiltration in the TNM classification system (chi-square test: $P = 0.018$) as well as with stage of cancer ($P = 0.04$), but not with lymph node metastasis, distant metastasis, extent of the lesions, invasion into lymphatic or blood vessels, intramural metastasis, or histologic grades of differentiation. In contrast, staining of MMP-2 protein within cancerous lesions was associated positively with invasion into

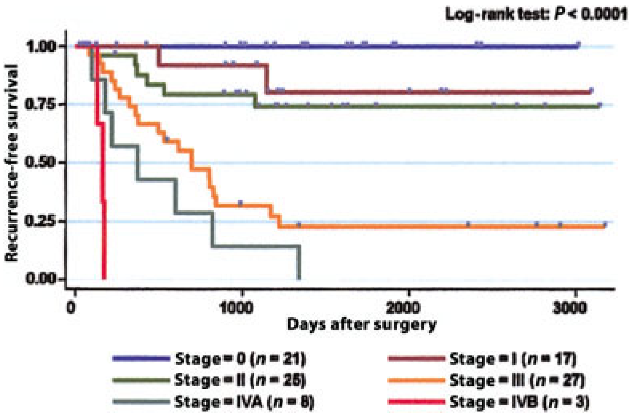

FIGURE 3. Stage (0, I, II, III, IVA, IVB) and disease recurrence-free survival using the Kaplan–Meier method (n = 101).

lymphatic tissue (P = 0.005), as well as with blood vessels (P = 0.023) and immature differentiation (P = 0.003) of histopathology.

CD147 and MMP-2 Expression Patterns and Disease Recurrence-Free Survival

Kaplan–Meier survival curves were first created based on tumor stages (0, n = 21; I, n = 17; II, n = 25; III, n = 27; IVA, n = 8; and IVB, n = 3) for the 101 patients (Fig. 3). As expected, patients with more advanced stages of cancer had shorter median disease recurrence-free survival periods: Stage III, 697 days; Stage IVA, 373 days; Stage IVB, 162 days (log-rank test: P < 0.0001).

According to the pattern of CD147 expression noted among cancerous and dysplastic cells, Kaplan–Meier survival curves were compared using the log-rank test. Neither cancerous nor dysplastic tissue expression of CD147 was associated with disease recurrence-free survival without adjusting for stages.

MMP-2 protein expression within normal tissue reduced disease recurrence-free survival significantly (P = 0.022) in patients with Stage IVA and IVB disease (Fig. 4A), but not in patients with Stage III and lower stages of disease (Fig. 4B). The presence of MMP-2 protein within cancerous tissue had no significant effect on disease recurrence-free survival, regardless of cancer stage.

The Cox Hazard Model

Cox regression analysis was performed to determine if the studied prognostic significance of CD147 and/or MMP-2 expression improved the accuracy of staging in predicting disease recurrence-free survival. Including data on CD147 expression in cancerous lesions significantly improved the model of ordinal staging alone (log-likelihood test: P = 0.019; Table 1). However, including data on MMP-2 expression in normal and/or cancerous tissue with disease stage, or including cancer stage plus CD147 expression data, did not alter the accuracy of the models.

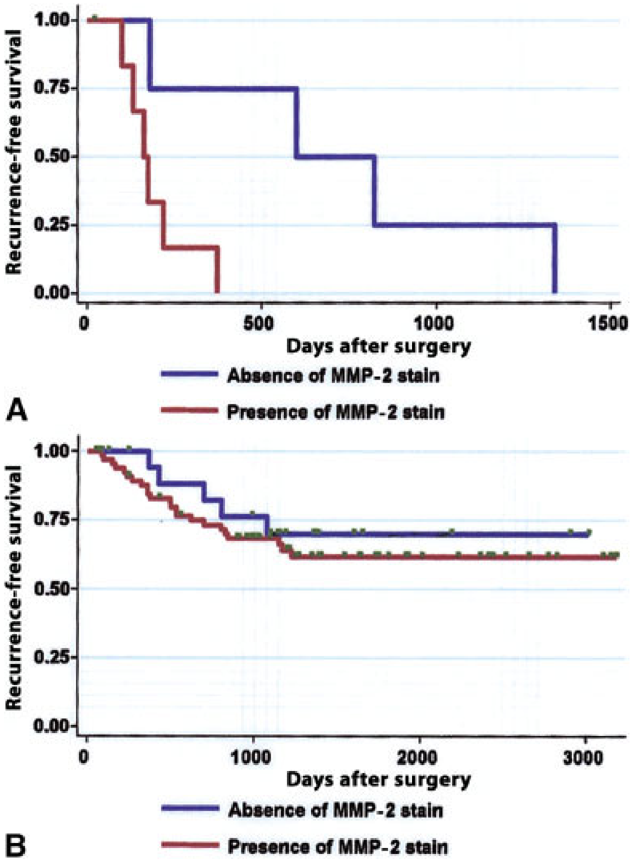

FIGURE 4. Matrix metalloproteinase-2 expression (presence/absence) in normal tissue adjacent to cancerous lesions and disease recurrence-free survival using the Kaplan–Meier method (n = 101). (A) Patients were restricted to Stage IVA and IVB disease. (B) Patients were restricted to Stage 0, I, II, and III disease.

TABLE 1
Cox Regression Analysis of CD147 Expression Levels in Cancerous Tissue Specimens Adjusted for Various Histologic Findings

Variables	Hazard ratio	95% Confidence interval	P value
Stage 0 and Stage I	1		
Stage II	5.2	1.0–25.9	0.041
Stage III	30.0	6.5–138	< 0.001
Stage IVA and Stage IVB	109.5	20.3–590	< 0.001
CD147 no staining	1		
CD147 partial staining	4.6	1.55–13.4	0.006
CD147 diffuse and strong staining	2.2	0.96–5.09	0.063

DISCUSSION

In the current study, we determined protein expression of CD147 and MMP-2 in cancerous and surrounding lesions with immunohistochemical staining, and explored the association of these protein expressions with clinicopathologic findings and disease recurrence-free survival in 101 patients with esophageal squamous cell carcinoma. CD147 protein was detected mainly in cancerous lesions and in some dysplastic lesions, but not in normal tissue. In contrast, MMP-2 protein was found in normal interstitial cells adjacent to cancer lesions but not in normal tissue far from cancer lesions. These findings are consistent with previous reports that CD147 expression on cancer cells stimulates surrounding normal tissue to produce MMP-2.[5–7] However, the intensity of the expression of CD147 in cancer lesions was not associated with that of MMP-2 in surrounding normal tissue, suggesting that reactivity of normal tissue to CD147 on cancer cells may be different among individuals.

Importantly, advanced T stage in the TNM classification system, which represents the depth of tumor infiltration, was associated positively with expression levels of MMP-2 in normal tissue. Moreover, greater expression of MMP-2 in normal tissue in patients with advanced-stage cancer was significantly associated with poor prognosis. These results suggest that MMP-2 expressed in normal cells may facilitate infiltration of cancer cells, resulting in advancement of T stage and reduced disease recurrence-free survival in patients with Stage IV disease.

In the current study, MMP-2 was expressed not only in normal tissue but also in cancer cells in some tissue specimens. Moreover, MMP-2 expression in cancer cells was associated with lymphatic and vascular invasion and cellular immaturity. In advanced gastric carcinoma, MMP-2 immunostaining has been observed exclusively and correlated with vascular invasion by tumor cells.[32,33] Furthermore, CD147 was shown to interact not only with adjacent stromal cells but also with MMP-1 on tumor cells.[34] These findings suggest that cancer cells may invade lymphatic and blood vessels by secreting MMP-2 themselves.

CD147 and MMP-2 expression have been demonstrated to be associated with tumor infiltration and invasion into vessels, suggesting that blockade of these molecules may prolong disease recurrence-free survival by interfering with tumor infiltration and invasion. In clinical trials, MMP inhibitors may enhance the effect of antitumor agents,[35,36] although the usage of an MMP inhibitor as a single agent has not been shown to be effective.[37,38]

In conclusion, measurement of CD147 and MMP-2 expression with simple immunohistochemical staining may further enhance understanding of the pathophysiology of invading tumor cells and, when used in combination with cancer staging, may increase our ability to predict prognosis in patients with esophageal squamous cell carcinoma.

REFERENCES

1. Anderson KC. Moving disease biology from the lab to the clinic. *Cancer.* 2003;97:796–801.
2. Richardson PG., Barlogie B, Berenson J, et al. A phase 2 study of bortezomib in relapsed, refractory myeloma. *N Engl J Med.* 2003;348:2609–2617.
3. Ellis SM, Nabeshima K, Biswas C. Monoclonal antibody preparation and purification of a tumor cell collagenase-stimulatory factor. *Cancer Res.* 1989;49:3385–3391.
4. Basset P, Bellocq JP, Wolf C, et al. A novel metalloproteinase gene specifically expressed in stromal cells of breast carcinomas. *Nature.* 1990;348:699–704.
5. Guo H, Li R, Zucker S, Toole BP. EMMPRIN (CD147), an inducer of matrix metalloproteinase synthesis, also binds interstitial collagenase to the tumor cell surface. *Cancer Res.* 2000;60:888–891.
6. Sameshima T, Nabeshima K, Toole BP, et al. Expression of emmprin (CD147), a cell surface inducer of matrix metalloproteinases, in normal human brain and gliomas. *Int J Cancer.* 2000;88:21–27.
7. Kanekura T, Chen X, Kanzaki T. Basigin (CD147) is expressed on melanoma cells and induces tumor cell invasion by stimulating production of matrix metalloproteinases by fibroblasts. *Int J Cancer.* 2002;99:520–528.
8. Ranuncolo SM, Armanasco E, Cresta C, et al. Plasma MMP-9 (92 kDa-MMP) activity is useful in the follow-up and in the assessment of prognosis in breast cancer patients. *Int J Cancer.* 2003;106:745–751.
9. Sienel W, Hellers J, Morresi-Hauf A, et al. Prognostic impact of matrix metalloproteinase-9 in operable non-small cell lung cancer. *Int J Cancer.* 2003;103:647–651.
10. Vihinen P, Kahari VM. Matrix metalloproteinases in cancer: prognostic markers and therapeutic targets. *Int J Cancer.* 2002;99:157–166.
11. Cai M, Onoda K, Takao M, et al. Degradation of tenascin-C and activity of matrix metalloproteinase-2 are associated with tumor recurrence in early stage non-small cell lung cancer. *Clin Cancer Res.* 2002;8:1152–1156.
12. Medical Research Council Oesophageal Cancer Working Group. Surgical resection with or without preoperative chemotherapy in oesophageal cancer: a randomised controlled trial. *Lancet.* 2002;359:1727–1733.
13. Kelsen DP, Ginsberg R, Pajak TF, et al. Chemotherapy followed by surgery compared with surgery alone for localized esophageal cancer. *N Engl J Med.* 1998;339:1979–1984.
14. Law S, Kwong DL, Kwok KF, et al. Improvement in treatment results and long-term survival of patients with esophageal cancer: impact of chemoradiation and change in treatment strategy. *Ann Surg.* 2003;238:339–347.
15. Ikeda M, Natsugoe S, Ueno S, Baba M, Aikou T. Significant host- and tumor-related factors for predicting prognosis in patients with esophageal carcinoma. *Ann Surg.* 2003;238:197–202.

16. Izbicki JR, Hosch SB, Pichlmeier U, et al. Prognostic value of immunohistochemically identifiable tumor cells in lymph nodes of patients with completely resected esophageal cancer. *N Engl J Med.* 1997;337:1188–1194.
17. Ishibashi Y, Hanyu N, Nakada K, et al. Endothelin protein expression as a significant prognostic factor in oesophageal squamous cell carcinoma. *Eur J Cancer.* 2003;39:1409–1415.
18. Ishibashi Y, Hanyu N, Nakada K, et al. Profiling gene expression ratios of paired cancerous and normal tissue predicts relapse of esophageal squamous cell carcinoma. *Cancer Res.* 2003;63:5159–5164.
19. van de Vijver MJ, He YD, van't Veer LJ, et al. A gene-expression signature as a predictor of survival in breast cancer. *N Engl J Med.* 2002;347:1999–2009.
20. Ye QH, Qin LX, Forgues M, et al. Predicting hepatitis B virus-positive metastatic hepatocellular carcinomas using gene expression profiling and supervised machine learning. *Nat Med.* 2003;9:416–423.
21. Sobin LH, Wittekind C. TNM classification of malignant tumors, 5th ed. New York: John Wiley & Sons, 1997.
22. The Japanese Society for Esophageal Diseases. Comprehensive registry of esophageal cancer in Japan (1998, 1999) and long-term results of esophagectomy in Japan (1988-1997), 3rd ed. Tokyo: The Japanese Society for Esophageal Diseases. Available from URL: http://plaza.umin.ac.jp/~jsed/file/crec/3rd_edition/CREC_JPN_3rd.pdf [accessed January 16, 2004].
23. Sarbia M, Porschen R, Borchard F, Horstmann O, Willers R, Gabbert HE. Incidence and prognostic significance of vascular and neural invasion in squamous cell carcinoma of the esophagus. *Int J Cancer.* 1995;61:333–336.
24. Theunissen PH, Borchaud F, Poortvliet DC. Histopathological evaluation of oesophageal carcinoma, the significance of venous invasion. *Br J Surg.* 1991;78:930–932.
25. Inoue T, Mori M, Shimono R, Kuwano H, Sugimachi K. Vascular invasion of colorectal carcinoma readily visible with certain stains. *Dis Colon Rectum.* 1992;35:34–39.
26. Takubo K, Sasashima K, Yamashita K, Tanaka Y, Fujita K. Prognostic significance of intramural metastasis in patients with esophageal carcinoma. *Cancer.* 1990;65:1816–1819.
27. Kuwano H, Watanabe M, Sadanaga N, et al. Univariate and multivariate analysis of the prognostic significance of discontinuous intramural metastasis in patients with esophageal cancer. *J Surg Oncol.* 1994;57:17–21.
28. Edwards JM, Hillier VF, Lawson RA, Moussalli H, Hasleton PS. Squamous carcinoma of the oesophagus, histological criteria and their prognostic significance. *Br J Cancer.* 1989;59:429–433.
29. Yamada K, Ohkawa K, Joh K. Monoclonal antibody, Mab 12C3, is a sensitive immunohistochemical marker of early malignant change in epitheial ovarian tumours. *Am J Clin Pathol.* 1995;103:288–294.
30. Vyberg M, Nielsen S. Dextran polymer conjugate two-step visualization system for immunohistochemistry. *Appl Immunohistochem.* 1998;6:3–10.
31. Kawahara E, Okada Y, Nakanishi I, et al. The expression of invasive behavior of differentiated squamous carcinoma cell line evaluated by an in vitro invasion model. *Jpn J Cancer Res.* 1993;84:409–418.
32. Nomura H, Sato H, Seiki M, Mai M, Okada Y. Expression of membrane-type matrix metalloproteinase in human gastric carcinomas. *Cancer Res.* 1995;55:3263–3266.
33. Nomura H, Fujimoto N, Seiki M, Mai M, Okada Y. Enhanced production of matrix metalloproteinases and activation of matrix metalloproteinase 2 (gelatinase A) in human gastric carcinomas. *Int J Cancer.* 1996;69:9–16.
34. Guo H, Li R, Zucker S, Toole BP. EMMPRIN (CD147), an inducer of matrix metalloproteinase synthesis, also binds interstitial collagenase to the tumor cell surface. *Cancer Res.* 2000;60:888–891.
35. Groves MD, Puduvalli VK, Hess KR, et al. Phase II trial of temozolomide plus the matrix metalloproteinase inhibitor, marimastat, in recurrent and progressive glioblastoma multiforme. *J Clin Oncol.* 2002;20:1383–1388.
36. Mitsiades N, Poulaki V, Mitsiades CS, Anderson KC. Induction of tumour cell apoptosis by matrix metalloproteinase inhibitors: new tricks from a (not so) old drug. *Expert Opin Invest Drugs.* 2001;10:1075–1084.
37. Moore MJ, Hamm J, Dancey J, et al. National Cancer Institute of Canada Clinical Trials Group. Comparison of gemcitabine versus the matrix metalloproteinase inhibitor BAY 12-9566 in patients with advanced or metastatic adenocarcinoma of the pancreas: a phase III trial of the National Cancer Institute of Canada Clinical Trials Group. *J Clin Oncol.* 2003;21:3296–3302.
38. Shepherd FA, Giaccone G, Seymour L, et al. Prospective, randomized, double-blind, placebo-controlled trial of marimastat after response to first-line chemotherapy in patients with small-cell lung cancer: a trial of the National Cancer Institute of Canada-Clinical Trials Group and the European Organization for Research and Treatment of Cancer. *J Clin Oncol.* 2002;20:4434–4439.
