# Supplementary material for: Expression of Concern: The prognostic and clinicopathologic characteristics of CD147 and esophagus cancer: A meta-analysis
Source: PLoS One. 2023 Feb 22;18(2):e0282229. doi: 10.1371/journal.pone.0282229 (PMC9946197; doi:10.1371/journal.pone.0282229)
Supplement: S1 File — (ZIP) [file pone.0282229.s001.zip › PDF of included paper/Clinical Impact of HAb18G CD147 Expression in Esophageal Squamous Cell Carcinoma.pdf]

Dig Dis Sci (2011) 56:3569–3576
DOI 10.1007/s10620-011-1812-x

ORIGINAL ARTICLE

Clinical Impact of HAb18G/CD147 Expression in Esophageal Squamous Cell Carcinoma

Shaojun Zhu · Yanhong Li · Li Mi · Yang Zhang · Li Zhang · Li Gong · Xiujuan Han · Li Yao · Miao Lan · Zhinan Chen · Wei Zhang

Received: 18 August 2010 / Accepted: 25 June 2011 / Published online: 26 July 2011
© Springer Science+Business Media, LLC 2011

Abstract
Background HAb18G/CD147 expression has been associated with many tumor invasion molecules, which play important roles in recurrence and poor differentiation of esophageal squamous cell carcinoma (ESCC). However, the clinical implications of HAb18G/CD147 in ESCC are still unclear.
Aims In this study, we clarified the clinical significance of HAb18G/CD147 and characterized the association between HAb18G/CD147 and tumor invasion in ESCC cases.
Methods Tumor tissues were obtained from 86 ESCC patients who underwent surgical resection between 2002 and 2005. All patients that had received previous therapy were excluded. ESCC tissues were analyzed by IHC using anti HAb18G/CD147 antibody. The expression of HAb18G/CD147 mRNA in esophageal cancer cell lines was analyzed by RT–PCR.
Results HAb18G/CD147 was uniformly expressed in EC109 and EC871214 cell lines, but negatively expressed in EPC2, esophageal normal squamous cell line. HAb18G/CD147 mainly localized to the membrane of tumor cells in 84.9% of ESCC patients (64 out of 86 cases). Furthermore, we also found that higher HAb18G/CD147 expression levels significantly correlated with lymph node metastasis, depth of tumor invasion and differentiation ($P < 0.05$). But the expression levels of HAb18G/CD147 in lymph node metastatic tissues were almost equal to that in the primary tumor tissues. Furthermore, lymph node metastasis and expression of HAB18G/CD147 were independent prognostic indicators in ESCC.
Conclusions The expression of HAb18G/CD147 might be involved in the progression and survival of ESCC. Therefore, HAb18G/CD147 could be a clinical marker for the poor prognosis in ESCC patients and may also be a potentially therapeutic target to improve the progression of ESCC.

Keywords HAb18G/CD147 · ESCC · Immunohistochemistry · Survival

Shaojun Zhu and Yanhong Li contributed equally to this article.

S. Zhu · Y. Li · L. Zhang · L. Gong · X. Han · L. Yao · M. Lan · W. Zhang (✉)
The Helmholtz Sino-German Research Laboratory for Cancer, Department of Pathology, Tangdu Hospital, Fourth Military Medical University, Xi'an 710038, Shaanxi, People's Republic of China
e-mail: zhwlyh@fmmu.edu.cn

Y. Li
Department of Gynecology and Obstetrics, Tangdu Hospital, Fourth Military Medical University, Xi'an, People's Republic of China

L. Mi · Y. Zhang · Z. Chen
State Key Laboratory of Cancer Biology, Cell Engineering Research Centre and Department of Cell Biology, National Key Discipline of Cell Biology, Fourth Military Medical University, Xi'an, People's Republic of China

Introduction

The origin of esophageal carcinoma (EC) has been related to many factors [1]. The major histological type is esophageal squamous cell carcinoma (ESCC) that comprises 90% of EC worldwide [2, 3]. A 5-year survival rate of 20–40% indicates its poor prognosis [4]. Although numerous advances occur in the treatment of ESCC, it is still identified as a locally advanced disease and tends to metastasize to adjacent lymph nodes and organs leading to a poor prognosis [5]. Therefore, exploration of novel

molecular markers in prognosis is required in clinical diagnosis and therapy.

HAb18G/CD147, a hepatoma associated antigen cloned by anti-hepatoma monoclonal antibody HAb18 screening of human hepatocellular carcinoma cDNA library, has an identical nucleotide and amino acid sequence to CD147. It is also called extracellular matrix metalloproteinase inducer (EMMPRIN) and is a member of the immunoglobulin superfamily, with a structure related to the putative primordial form of the family [6–8]. Previous studies found that HAb18G/CD147 is a multi-functional protein [9–12] and might promote invasion and metastasis by induction and regulation of basement membrane degrading proteases, e.g. MMP1, MMP2, MMP3 and MMP9, which are associated with tumor invasion and poor progression [13–17]. Therefore, HAb18G/CD147 seems to be associated with a poor prognosis in many types of tumors. Although HAb18G/CD147 expression has been identified in many solid tumors, including ESCC, renal carcinoma, hepatoma, melanoma, breast cancer, nasopharyngeal carcinoma and pancreatic carcinoma [18–25]. The association between HAb18G/CD147 expression and pathological characteristics or prognosis in ESCC remains unclear.

In this study, we evaluated the expression of HAb18G/CD147 in different esophageal cancer cell lines and tissues by RT-PCR, Western-blot and immunohistochemistry to clarify the clinical significance of HAb18G/CD147 and characterize the association between HAb18G/CD147 and tumor invasive features in ESCC progression.

Materials and Methods

Patients

Surgical specimens of ESCC were collected from 86 patients at the Department of Pathology, Tangdu Hospital, China, from 2002 to 2005. The surgically resected samples were taken before radiation therapy, chemotherapy, and combined therapy. The patients ranged in age from 40 to 78 years including 57 males and 29 females. Among them, tumor tissues from nine cases were diagnosed as low infiltrating; 26 cases were moderate infiltrating; 51 cases were deeply infiltrating. Lymph node metastasis occurred in 30 out of the 86 cases according to their histopathology. Hematoxylin and eosin (H&E) stained slides of all cases were reviewed. The diagnosis was evaluated by three pathologists respectively.

After surgery, the patients were followed-up for 4–6 years (up to April, 2009). All patients who died of other diseases except ESCC or unexpected events were excluded from the study. Informed consent was obtained from all patients. Every specimen was handled anonymously according to the ethical and legal standards. The study protocol was approved by the ethical guidelines of the 1975 Declaration of Helsinki as reflected in an a priori approval by Tangdu Hospital of the Fourth Military Medical University.

Cell Culture

Complete medium (RPMI-1640) consisted of RPMI-1640 supplemented with 2 mmol/L Glutamax, 100 units/mL penicillin, 100 mg/mL streptomycin, 10 mmol/L HEPES (Invitrogen, USA.) and 10% FCS (Thermo Trace, Australia). The cell lines (EPC2, Eca-109, EC871214 and SMMC-7721) were purchased from Zhongshan University (China) and kept by the Department of Pathology of Tangdu Hospital. All tumor cell lines were cultured in complete RPMI-1640.

Semi-Quantitative and Reverse-Transcription PCR

Total RNA was isolated from cell lines using Trizol reagent (Invitrogen, USA). Amount of 500 ng RNA was reverse transcribed using Kit (Takara, Japan). Subsequent RT-PCR for HAb18G/CD147 fragment amplification was carried out. The following primers were used (5′–3′): sense: CGGAGTC CACTCCCAGTGC; antisense: CCATGACTCAGAC CCA GAGG. β-actin was used as an internal control for normalization (primer sequences available on request). Semi-quantitative RT-PCR of HAb18G/CD147 transcripts were done by comparing the signal intensities of PCR products of the HAb18G/CD147 gene to that of the β-actin gene from the same RNA sample by agarose gel electrophoresis. The intensities of the product bands were quantified by densitometric scanning in the gel documentation system (Pharmacia Biotech) using 'Total image' 1D GEL ANALYSIS software. A DNA marker (Takara, Japan) was run with each gel to confirm the size of the PCR product.

Western-Blot Analysis

To assess HAb18G/CD147 expression, whole cell lysates were subjected to SDS–PAGE electrophoresis, followed by blotting onto a nitrocellulose (NC) membrane. Membranes were probed with mouse anti human HAb18G/CD147 monoclonal antibody overnight at 4°C followed by a secondary horseradish peroxidase-conjugated antibody (HAb18G/CD147 was obtained by Cell Engineering Research Center and Department of Cell Biology, Fourth Military Medical University, No.CGMCC0426). Mouse anti-human β-actin monoclonal antibody was used as an internal control (R&D, USA). The membrane was incubated with enhanced chemiluminescence detection kit (Anmei, China) and then exposed to X-ray film and developed.

Immunohistochemistry

Paraffin-embedded resected ESCC specimens were retrieved from Tangdu hospital tissue bank. The paraffin-embedded tissues were cut at 4 μm, deparaffinized with xylene and rehydrated for further peroxidase (DAB) immunohistochemistry staining using a MaxVisionTMHRP-Polymer anti-mouse/Rabbit IHC kit (Fuzhou Maxim, China). Deparaffinized sections were treated with methanol containing 3% hydrogen peroxide for 12 min. After washing with PBS, blocking serum was applied for 30 min. The sections were incubated with the anti-HAb18G/CD147 monoclonal antibody overnight at 4°C. A biotin-marked secondary antibody was applied for 20 min at 37°C, followed by a peroxidase-marked streptavidin for an additional 20 min. After washing, substrate-chromogen was used to visualize the staining of the targeted proteins, and the nuclei were counterstained with hematoxylin. Positive and negative immunohistochemistry controls were routinely used. The slides were examined systematically using an image analyzer system (Olympus BH-2 microscope; Japan).

Histopathological Assessment of Immunohistochemistry

Histological type of differentiation, tumor origin, lymph node metastasis and invasion were prospectively registered according to a standardized protocol for histopathological examination. Slides were reviewed by three experienced pathologists independently. Semiquantitative analysis of HAb18G/CD147 staining was assessed as 0, 1+, 2+, and 3+ [26, 27]. Grade 0 was defined as the complete absence or weak HAb18G/CD147 immunostaining in <5% of the tumor cells; grade 1+ was focal positivity in 5–25% of the tumor cells; grade 2+ was frequency staining in 25–50% of the tumor cells; and grade 3+ was frequency staining in >50% of the tumor cells. A global assessment of the entire tumor was made without selection for the invasive front or areas of active tumor growth. The frequency staining and semiquantitative analysis of positive tumors for all regions was calculated for statistical comparisons.

Statistical Analysis

SPSS13.0 software for Windows (SPSS Inc., Chicago, Ill) and SAS 9.1 (SAS Institute, Cary, NC) was used for statistical analysis. Continuous variables were expressed as means ± standard error. Differences in proportions were compared with Mann–Whitney U test. Statistical correlations between differentiation or depth of tumor invasion and the staining level of anti HAb18G/CD147 antibody were analyzed by the Cochran-Mantel–Haenszel test. Cox regression analysis was used to identify the multivariate factor relating to prognosis. Differences with a $P < 0.05$ were considered statistically significant.

Results

Expression of HAb18G/CD147 in Esophageal Carcinoma Cell Lines

The expression of HAb18G/CD147 in the esophageal carcinoma cell lines was analyzed by semiquantitative RT-PCR and Western-blot. The results of RT-PCR are shown in Fig. 1a. HAb18G/CD147 was uniformly transcripted in EC109 and EC871214 cell lines. But it was not found in EPC2 (normal esophageal cell line). Furthermore, the HAb18G/CD147 protein was also detected in esophageal carcinoma cell lines, not EPC2, by Western blotting (Fig. 1b), although the intensity of expression was variable.

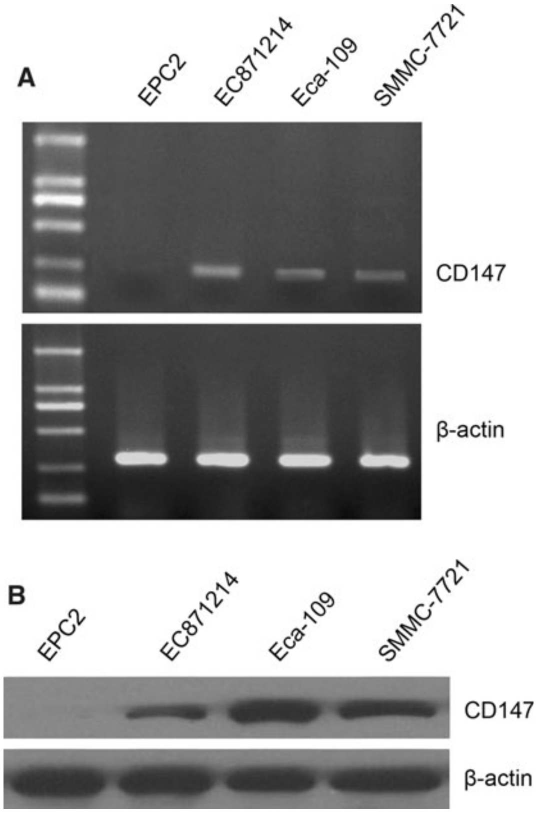

Fig. 1 Expression of HAb18G/CD147 in esophageal carcinoma cell lines. **a** Expression of HAb18G/CD147 mRNA in different esophageal cell lines and hepatoma cell line (as positive control) by RT-PCR. *β*-actin was used to verify the integrity of the template cDNA preparations. **b** Expression of HAb18G/CD147 in different esophageal cell lines. The hepatoma cell line was analyzed by Western blotting. The EPC2, normal cell line, served as negative control

HAb18G/CD147 Expression and Localization in ESCC Tissues

IHC of anti HAb18G/CD147 antibody in ESCC specimens was done on a set of 86 tissue sections of ESCC (Table 1). Pathologists evaluated the expression of HAb18G/CD147 in different ESCC samples according to the percentage of anti-human HAb18G/CD147 antibody staining (Figs. 2, 3).

It was mostly localized to the membrane of esophageal squamous cell carcinoma cells (73 out of 86 cases, 84.9%) and seldom expressed in the cytoplasm (13 out of 86 cases, 15.1%). In all, 22 out of 86 cases (25.6%) did not express HAb18G/CD147; 37 cases (43.0%) showed low expression, 18 cases (20.9%) showed intermediate expression, and nine cases (10.5%) showed high expression.

Clinic-Pathological Features of HAb18G/CD147 in ESCC

The association between HAb18G/CD147 expression and the clinic-pathological characteristics of ESCC patients is shown in Table 1. Statistical results of the IHC showed that the expression of HAb18G/CD147 was higher in lymph node metastasis cases ($P = 0.003$) and poor prognosis cases ($P < 0.001$), and the expression is correlated with differentiation of ESCC ($P = 0.002$). A clear linear trend of ESCC differentiation was observed by the HAb18G/CD147 staining scores (nonzero correlation = 9.199, $P = 0.002$; general association = 15.568, $P = 0.016$; departure from linear regression = 6.369, $0.5 < P < 0.25$; Table 1). A significant linear trend between depth of tumor invasion and HAb18G/CD147 expression was found in ESCC tissues as well (non-zero correlation = 5.857, $P = 0.016$; general association = 9.079, $P = 0.169$; departure from linear regression = 3.223, $0.75 < P < 0.5$). Gender and age were also found to be unrelated with the expression of HAb18G/CD147 ($P = 0.569$).

HAb18G/CD147 Expression in Lymph Node Metastasis Deposits of ESCC

IHC staining was performed on 27 tissue sections of lymph node metastasis deposits in ESCC (Table 2). The pathologists evaluated and compared the expression levels of HAb18G/CD147 in lymph node metastasis tissues to the corresponding primary cancer tissues. As shown in Table 2, the expression of HAb18G/CD147 in lymph node

Table 1 Association of HAb18G/CD147 with clinicopathologic grades and staining frequency in 86 ESCC in tissue sections

HAb18G/CD147 expression	No. of tumor specimens ($N = 86$)	Anti HAB18G/CD147 Ab immunohistochemistry frequency score, *n* (%)				*P*
		0	1	2	3	
Gender						
Male	57	18 (31.6)	19 (33.3)	13 (22.8)	7 (12.3)	0.779
Female	29	4 (13.8)	18 (62.1)	5 (17.2)	2 (6.9)	
Age						
> 60	45	12 (26.7)	18 (40.0)	9 (20.0)	6 (13.3)	0.805
≤ 60	41	10 (24.4)	19 (46.3)	9 (22.0)	3 (7.3)	
Grade						
Well differentiated	16	6 (37.5)	8 (50.0)	2 (12.5)	0 (0)	0.002*[a]
Moderately differentiated	46	11 (23.9)	23 (50.0)	10 (21.7)	2 (4.3)	
Poorly differentiated	24	5 (20.8)	6 (25)	6 (25)	7 (29.2)	
Depth of tumor invasion						
Mucous layer	9	4 (44.4)	4 (44.4)	0 (0)	1 (11.1)	0.016*[a]
Muscular layer	26	8 (30.8)	13 (50)	5 (19.2)	0 (0)	
Serous layer	51	10 (19.6)	20 (39.2)	13 (25.5)	8 (15.7)	
Lymph node metastasis						
Absent	56	16 (28.6)	30 (53.6)	8 (14.3)	2 (3.6)	0.003*
Present	30	6 (20.0)	7 (23.3)	10 (33.3)	7 (23.3)	
Outcome						
Alive	37	14 (37.8)	19 (51.4)	4 (10.8)	0 (0)	0.000*
Death	49	8 (16.3)	18 (36.7)	14 (28.6)	9 (18.4)	

ESCC esophageal squamous cell carcinoma

[a] Statistic by CMH (Cochran-Mantel–Haenszel test)

* $P < 0.05$

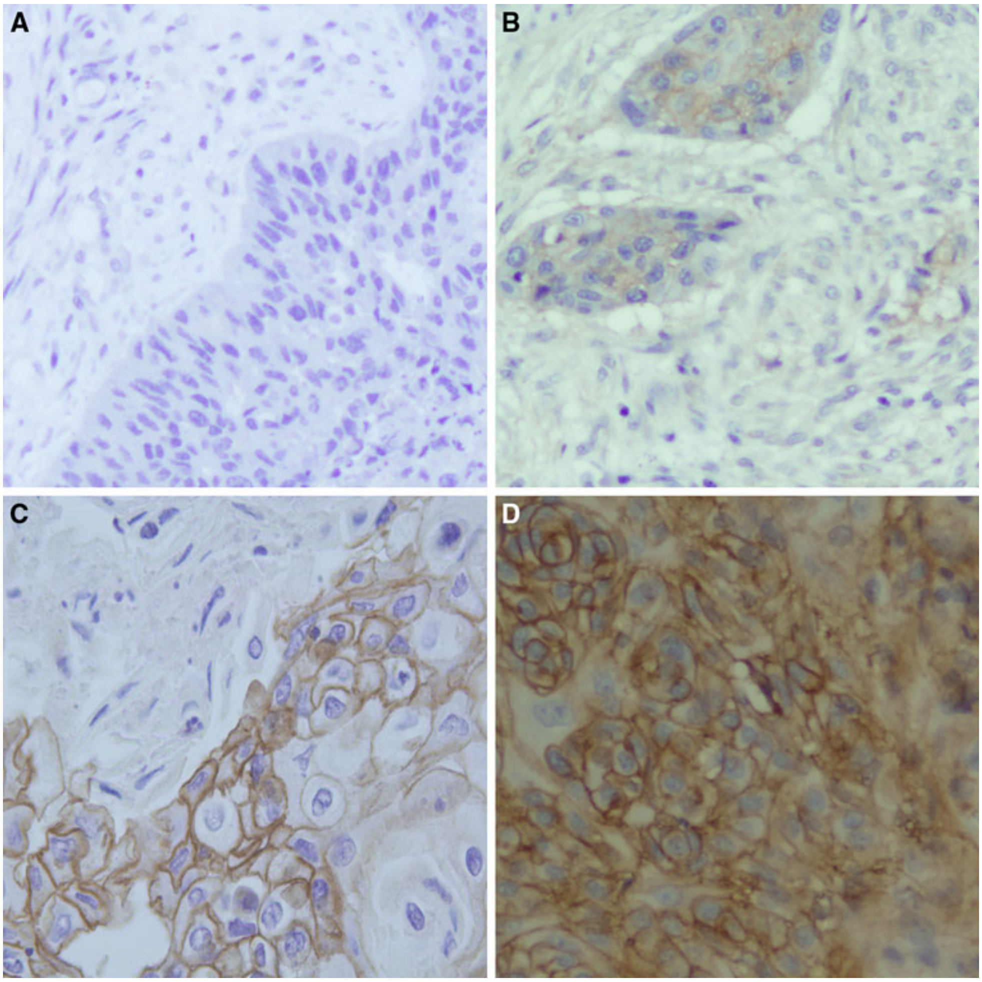

Fig. 2 Standard staining frequency scores of anti HAb18G/CD147 antibody in esophageal squamous cell carcinoma (ESCC) tissues (200×). **a** Score 0 (staining localized). **b** Score 1 (staining frequency < 25%). **c** Score 2 (staining frequency 25–50%). **d** Score 3 (staining frequency > 50%)

metastatic tissues were almost equal to that in the primary tumor tissues ($P = 0.213$).

Prognostic Implications of HAb18G/CD147 in ESCC

After analyzing the survival information, we found that 3-year survival rates of ESCC patients were 46.5% (40/86). In this study, the median survival time was 53 months (95% CI, 48–56). Using Cox regression analysis of the 86 patients, lymph node metastasis and expression of HAB18G/CD147 were independent prognostic indicators ($P = 0.002$ and $P = 0.021$, respectively, Table 3) in ESCC.

Discussion

Previous studies reported that HAb18G/CD147 was mainly expressed at the periphery of invasive tumor clusters corresponding to the leading edge of tumor invasion [28], although there is limited evidence to elucidate the mechanisms of HAb18G/CD147 functions in tumor progression and invasion. The assumption that HAb18G/CD147 was involved in tumor progression has been strengthened by the fact that a high level of HAb18G/CD147 occurs in numerous malignant tumors, including bladder, skin, lung carcinoma, breast carcinoma, hepatoma, cervical squamous cell carcinoma and lymphoma [29–35].

In this research, we identified the expression of HAb18G/CD147 in different esophageal cell lines and the relationships between HAb18G/CD147 expression and clinical characteristics including gender, lymph node metastasis, depth of tumor invasion, tumor differentiation and survival in ESCC tissues. HAb18G/CD147 mRNA and protein were positively expressed in esophageal carcinoma cell lines, but not in normal cell lines. IHC analysis of ESCC tissues hinted that the expression of HAb18G/CD147 could promote tumor cells not only to transfer to lymph nodes, but also to deeply infiltrate into the wall of esophagus. HAb18G/CD147 is also highly expressed in

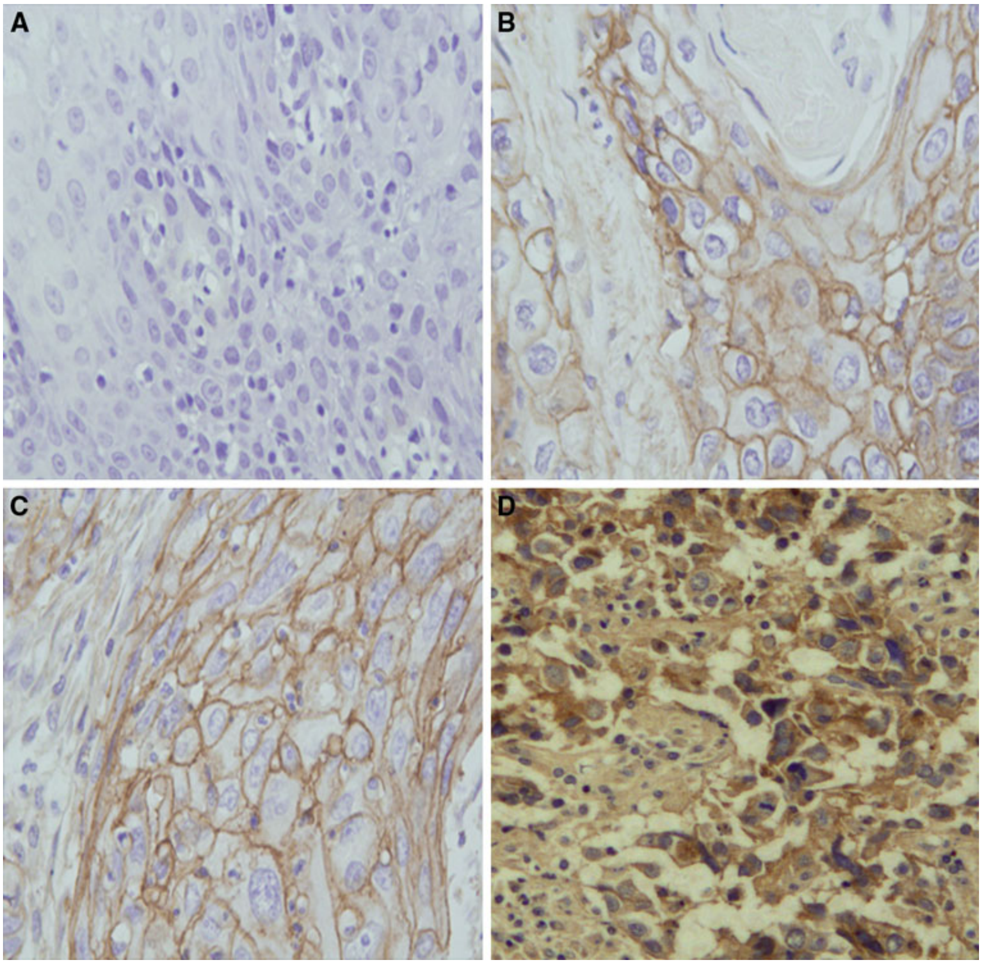

Fig. 3 IHC staining of anti HAb18G/CD147 antibody in vary differentiated esophageal squamous cell carcinoma (ESCC) tissues (200×). **a** Normal tissue. **b** Well-differentiated ESCC. **c** Moderately-differentiated ESCC. **d** Poorly-differentiated ESCC

Table 2 HAb18G/CD147 expression in lymph node metastasis focus and corresponding primary site in 27 ESCC in tissue sections

HAb18G/CD147 expression	No. of tumor specimens ($N = 27$)	HAb18G/CD147 immunohistochemistry frequency score, *n* (%)				*P*
		0	1	2	3	
Primary cancerous tissue	27	6 (22.2)	6 (22.2)	9 (33.3)	6 (22.2)	0.213
Lymph node metastasis focus	27	8 (29.6)	10 (37.0)	5 (18.5)	4 (14.8)	

ESCC esophageal squamous cell carcinoma

Table 3 Prognostic value of HAb18G/CD147 positive expression in multivariate analysis by Cox Proportional hazard model (stepwise)

Variable	*df*	Parameter estimate	Standard error	Chi-square	Pr > chi-square	Hazard ratio
HAb18G/CD147 expression	1	0.394	0.171	5.309	0.021*	1.482
Lymph node metastasis	1	0.966	0.319	9.198	0.002*	2.629

* $P < 0.05$

many cases of patients with poor survival. Significant differences in prognosis were found among different expression levels of HAb18G/CD147 in ESCC. Patients with lower HAb18G/CD147 expression had better outcome, whereas higher HAb18G/CD147 expression was associated with worse outcome. This is consistent with previous results that the expression of HAb18G/CD147 was higher in poorly differentiated ESCC tissues than that in more

highly differentiated ones. Expression of HAb18G/CD147 in lymph node metastasis deposit was equivalent to that of the corresponding primary tumor tissue. It was of note that, in few cases, HAb18G/CD147 was negatively expressed in lymph node metastasis tissues and positively expressed in the primary tumor tissues, which was correlated to a better outcome. However, because of the limited number of cases, the statistical significance of this relationship needs more work to be confirmed.

HAb18G/CD147 is a multifunctional protein and plays important roles in many checkpoints of tumor invasion and metastasis [9–12]. Many studies reported that HAb18G/CD147 facilitated the production of MMPs from adjacent stroma cells to more distal ones [8]. That may suggest that the expression of HAb18G/CD147 promoted tumor cells to transfer to distant sites [36]. Some reports elucidated that HAb18G/CD147 could bind to MMP-1 at the tumor cell surface, potentially concentrating and localizing the collagen degrading enzyme at this site, thereby promoting cell invasion [37]. Furthermore, HAb18G/CD147 has a paracrine effect on MMPs production by endothelial cells, suggesting involvement of tumor-produced HAb18G/CD147 in angiogenesis through the regulation of MMPs by endothelial cells, permitting greater infiltration into adjacent normal tissue [38, 39]. These features and exhibits are consistent with our results about the relationship between the expression of HAb18G/CD147 and poor prognosis in ESCC. Our finding indicated that HAb18G/CD147 could be an independent indicator to identify patients with more aggressive tumors in clinical diagnosis and suggested that the survival of ESCC patients might be influenced by the expression and functions of HAb18G/CD147.

However, HAb18G/CD147 associated functions and network molecules in ESCC are still unclear. According to our results, we reached the conclusion that the expression of HAb18G/CD147 can be an important indicator in the prognosis and progression of ESCC. In this research, the anti HAb18G/CD147 monoclonal antibody, which was developed by the Cell Engineering Research Center and Department of Cell Biology, described previously, was screened to bind the transmembrane domain of HAb18G/CD147 specifically. This may confine the research work in the function of HAb18G/CD147 in membrane pattern. Although we found some cytoplasm staining of HAb18G/CD147 in IHC, the cases were limited [25, 40]. More work is necessary to study the function of HAb18G/CD147 in the cytoplasm.

In conclusion, HAb18G/CD147 might be involved in the progression of ESCC. Its clinical characteristics in ESCC warrants further study to understand its roles in the development and progression of ESCC. Therefore, HAb18G/CD147 could be applied as a clinical marker for the poor prognosis in ESCC patients and may also be a potentially therapeutic target to improve the progression of ESCC.

Acknowledgments This work was supported by The National Natural Science Foundation of China (No. 30672013 and No. 81001088) and The National Basic Research Program (973 Program) of China (No. 2009CB521705).

Conflicts of interest None to declare.

References

1. Stoner GD, Gupta A. Etiology and chemoprevention of esophageal squamous cell carcinoma. *Carcinogenesis*. 2001;22:1737–1746.
2. Stoner GD, Rustgi AK. Biology of the esophageal squamous cell carcinoma. *Gastrointest Cancers Biol Diagn*. 1995;8:141–146.
3. WHO. The World Health Report 1997—conquering suffering, enriching humanity. *World Health Forum*. 1997;18:248–260.
4. Reed CE. Surgical management of esophageal carcinoma. *Oncologist*. 1999;4:95–105.
5. De LL, Curia MC, Aceto GM, et al. Analysis of extended genomic rearrangements in oncological research. *Ann Oncol*. 2007;18:173–178.
6. Biswas C, Zhang Y, DeCastro R, et al. The human tumor cell-derived collagenase stimulatory factor (renamed EMMPRIN) is a member of the immunoglobulin superfamily. *Cancer Res*. 1995;55:434–439.
7. Tang W, Chang SB, Hemler ME. Links between CD147 function, glycosylation, and caveolin-1. *Mol Biol Cell*. 2004;15:4043–4050.
8. Yan L, Zucker S, Toole BP, et al. Roles of the multifunctional glycoprotein, emmprin (basigin; CD147), in tumour progression. *Thromb Haemost*. 2005;93:199–204.
9. Kirk P, Wilson MC, Heddle C, et al. CD147 is tightly associated with lactate transporters MCT1 and MCT4 and facilitates their cell surface expression. *EMBO J*. 2000;19:3896–3904.
10. Xu D, Hemler ME. Metabolic activation-related CD147-CD98 complex. *Mol Cell Proteomics*. 2005;4:1061–1071.
11. Berditchevski F, Chang S, Bodorova J, et al. Generation of monoclonal antibodies to integrin-associated proteins. Evidence that alpha3beta1 complexes with EMMPRIN/basigin/OX47/M6. *J Biol Chem*. 1997;272:29174–29180.
12. Curtin KD, Meinertzhagen IA, Wyman RJ. Basigin (EMMPRIN/CD147) interacts with integrin to affect cellular architecture. *J Cell Sci*. 2005;118:2649–2660.
13. Gordon JM, Bauer EA, Eisen AZ. Collagenase in human cornea: immunologic localization. *Arch Ophthalmol*. 1980;98:341–345.
14. Wagoner MD, Kenyon KR. Distribution of collagenase and cell types in sterile ulceration of human corneal grafts. *Acta Ophthalmol*. 1989;192:65–71.
15. Major TC, Liang L, Lu X, Rosebury W, Bocan TM. Extracellular matrix metalloproteinase inducer (EMMPRIN) is induced upon monocyte differentiation and is expressed in human atheroma. *Arterioscler Thromb Vasc Biol*. 2002;22:1200–1207.
16. Li Z, Ren Y, Wu QC, et al. Macrophage migration inhibitory factor enhances neoplastic cell invasion by inducing the expression of matrixmetalloproteinase 9 and interleukin-8 in nasopharyngeal carcinoma cell lines. *J Chin Med*. 2004;117:107–114.
17. Zucker S, Hymowitz M, Rollo EE, et al. Tumorigenic potential of extracellular matrixmetalloproteinase inducer. *Am J Pathol*. 2001;158:1921–1928.
18. Ramos-Simone N, Hahn-Dantona E, Sipley J, et al. Activation of matrix metalloprotease-9 (MMP-9) via a converting plasmin/stromelysin-1 cascade enhances tumor cell invasion. *J Biol Chem*. 1999;274:13066–13076.
19. Davidson B, Goldberg I, Berner A, et al. EMMPRIN (extracellular matrix metalloproteinase inducer) is a novel marker of poor

outcome in serous ovarian carcinoma. *Clin Exp Metastasis*. 2003;20:161–169.
20. Guo H, Li R, Zucker S, et al. EMMPRIN (CD147), an inducer of matrix metalloproteinase synthesis, also binds interstitial collagenase to the tumor cell surface. *Cancer Res*. 2000;60:888–891.
21. Bourguignon L, Gunja-Smith Z, Iida N, et al. CD44v (3, 8–10) is involved in cytoskeleton-mediated tumor cell migration and matrix metalloproteinase (MMP-9) association in metastatic breast cancer cells. *J Cell Physiol*. 1998;176:206–215.
22. Li Z, Ren Y, Wu QC, et al. Macrophage migration inhibitory factor enhances neoplastic cell invasion by inducing the expression of matrix metalloproteinase 9 and interleukin-8 in nasopharyngeal carcinoma cell lines. *Chin Med J*. 2004;117:107–114.
23. Tang J, Zhou HW, Jiang JL, et al. ßig-h3 is involved in the CD147/CD147-mediated metastasis process in human hepatoma cells. *Exp Biol Med*. 2007;232:344–352.
24. Cheng MF, Tzao C, Tsai WC, et al. Expression of EMMPRIN and matriptase in esophageal squamous cell carcinoma: correlation with clinicopathological parameters. *Dis Esophagus*. 2006;19:482–486.
25. Tang J, Wu YM, Zhao P, Yang XM, Jiang JL, Chen ZN. Overexpression of HAb18G/CD147 promotes invasion and metastasis via alpha3beta1 integrin mediated FAK-paxillin and FAK-PI3K-Ca2+ pathways. *Cell Mol Life Sci*. 2008;65:2933–2942.
26. Iwasa S, Okada K, Chen WT, et al. Increased expression of seprase, a membrane-type serine protease, is associated with lymph node metastasis in human colorectal cancer. *Cancer Lett*. 2003;199:91–98.
27. Ariga N, Sato E, Ohuchi N, Nagura H, Ohtani H. Stromal expression of fibroblast activation protein/seprase, a cell membrane serine proteinase and gelatinase, is associated with longer survival in patients with invasive ductal carcinoma of breast. *Int J Cancer*. 2001;95:67–72.
28. Caudroy S, Polette M, Tournier JM, et al. Expression of the extracellular matrix metalloproteinase inducer (EMMPRIN) and the matrix metalloproteinase-2 in bronchopulmonary and breast lesions. *J Histochem Cytochem*. 1999;47:1575–1580.
29. Polette M, Gilles C, Marchand V, et al. Tumor collagenase stimulatory factor (TCSF) expression and localization in human lung and breast cancers. *J Histochem Cytochem*. 1997;45:703–709.
30. Bordador LC, Li X, Toole B, et al. Expression of EMMPRIN by oral squamous cell carcinoma. *Int J Cancer*. 2000;85:347–352.
31. Thorns C, Feller AC, Merz H. EMMPRIN (CD 174) is expressed in Hodgkin's lymphoma and anaplastic large cell lymphoma. An immunohistochemical study of 60 cases. *Anticancer Res*. 2002;22:1983–1986.
32. Gabison EE, Huet E, Baudouin C, Menashi S. Direct epithelial-stromal interaction in corneal wound healing: Role of EMMPRIN/CD147 in MMPs induction and beyond. *Prog Retin Eye Res*. 2009;28:19–33.
33. Yu W, Liu J, Xiong X, Ai Y, Wang H. Expression of MMP9 and CD147 in invasive squamous cell carcinoma of the uterine cervix and their implication. *Pathol Res Pract*. 2009;205:709–715.
34. Riethdorf S, Reimers N, Assmann V, et al. High incidence of EMMPRIN expression in human tumors. *Int J Cancer*. 2006;119:1800–1810.
35. Nabeshima K, Iwasaki H, Koga K, Hojo H, Suzumiya J, Kikuchi M. Emmprin (basigin/CD147): matrix metalloproteinase modulator and multifunctional cell recognition molecule that plays a critical role in cancer progression. *Pathol Int*. 2006;56:359–367.
36. Tang Y, Kesavan P, Nakada MT, Yan L. Tumor-stroma interaction: positive feedback regulation of extracellular matrix metalloproteinase inducer (EMMPRIN) expression and matrix metalloproteinase-dependent generation of soluble EMMPRIN. *Mol Cancer Res*. 2004;2:73–80.
37. Moll UM, Lane B, Zucker S, Suzuki K, Nagase H. Localization of collagenase at the basal plasma membrane of a human pancreatic carcinoma cell line. *Cancer Res*. 1990;50:6995–7002.
38. Caudroy S, Polette M, Nawrocki-Raby B, et al. EMMPRIN-mediated MMP regulation in tumor and endothelial cells. *Clin Exp Metastasis*. 2002;19:697–702.
39. Menashi S, Serova M, Ma L, Vignot S, Mourah S, Calvo F. Regulation of extracellular matrix metalloproteinase inducer and matrix metalloproteinase expression by amphiregulin in transformed human breast epithelial cells. *Cancer Res*. 2003;63:7575–7580.
40. Qian AR, Zhang W, Cao JP, et al. Downregulation of CD147 expression alters cytoskeleton architecture and inhibits gelatinase production and SAPK pathway in human hepatocellular carcinoma cells. *J Exp Clin Cancer Res*. 2008;27:50.
