# Supplementary material for: Expression of Concern: The prognostic and clinicopathologic characteristics of CD147 and esophagus cancer: A meta-analysis
Source: PLoS One. 2023 Feb 22;18(2):e0282229. doi: 10.1371/journal.pone.0282229 (PMC9946197; doi:10.1371/journal.pone.0282229)
Supplement: S1 File — (ZIP) [file pone.0282229.s001.zip › PDF of included paper/Expression of EMMPRIN and matriptase in esophageal squamous cell carcinomaú║Correlation with clinicopathological parameters.pdf]

Diseases of the Esophagus (2006) **19**, 482–486
DOI: 10.1111/j.1442-2050.2006.00613.x

DISEASES OF THE ESOPHAGUS

Original article

Expression of EMMPRIN and matriptase in esophageal squamous cell carcinoma: Correlation with clinicopathological parameters

M.-F. Cheng,[1] C. Tzao,[2] W.-C. Tsai,[1] W.-H. Lee,[1] A. Chen,[1] H. Chiang,[3] L.-F. Sheu,[1] J.-S. Jin[1]

[1]*Department of Pathology,* [2]*Division of Thoracic Surgery, Tri-Service General Hospital, National Defense Medical Center, and* [3]*Taipei Institute of Pathology, Taiwan, Republic of China*

SUMMARY. **Extracellular matrix metalloproteinase inducer (EMMPRIN) and the type II transmembrane serine protease, matriptase, are expressed in several human cancers and play an important role in tumor progression. The aim of the present study was to investigate the immuno-staining patterns of EMMPRIN and matriptase in patients with esophageal squamous cell carcinomas (SCC) and correlate the percentage tumor staining with tumor differentiation and clinical parameters. EMMPRIN and matriptase immunoreactivity was seen on the cell membrane and in the cytoplasm of tumor cells in all 41 cases of esophageal SCC evaluated. The percentage tumor staining of EMMPRIN was 48 ± 3% for well differentiated, 73 ± 3% for moderately differentiated, and 92 ± 3% for poorly differentiated esophageal SCC. Higher percentage tumor staining with EMMPRIN correlated significantly with poorly differentiated esophageal SCC ($P < 0.05$). The percentage tumor staining with matriptase correlated significantly with tumor differentiation (52 ± 3% for well differentiated, 85 ± 2% for moderately differentiated, and 88 ± 3% for poorly differentiated esophageal SCC). Additionally, higher percentage tumor staining with matriptase was significantly correlated with the advanced N and M stages ($P < 0.05$). Our results demonstrate that EMMPRIN and matriptase are over-expressed in esophageal SCC and are correlated with advanced clinicopathological stages. Pharmacological agents targeting EMMPRIN and matriptase expressions may be beneficial in the treatment of esophageal SCC.**

KEY WORDS: **CD147, EMMPRIN, esophagus, matriptase, squamous cell carcinoma.**

INTRODUCTION

Esophageal squamous cell carcinoma (SCC) is one of the most common cancers in the world and has a poor prognosis.[1–3] Despite improved survival rates due to surgical treatment and chemotherapy, median survival is still poor, ranging from 1–2 years, and overall 5-year survival rates range from 5–35%.[4–8] Several studies have demonstrated that gene expression profiles, such as Ki67, p53, cyclin D1, pRB and p63, may influence the prognosis and carcinogenesis of esophageal SCC.[9–12] However, there is still no convincing biomarker to predict the prognosis of patients with esophageal SCC.

Extracellular matrix metalloproteinase inducer (EMMPRIN), known as CD147 or basigin, is a 55-kDa molecule found on the surface of tumor cells. It can stimulate the expression of matrix metalloproteinases that facilitate the invasiveness of cancer cells.[3,13] The role of EMMPRIN in tumor behavior has been demonstrated in several malignancies, such as serous ovarian and breast cancers.[13–15] Although one study showed the immunohistochemistry expression of EMMPRIN in individual cases was not associated with the clinical stages of esophageal cancers,[3] the important role of EMMPRIN as yet needs to be clarified.

Matriptase, also known as membrane-type serine protease-1 (MT-SP1) or tumor-associated differentially expressed gene-15, is able to cleave various synthetic substrates, such as hepatocyte growth factor (HGF) and urokinase-type plasminogen activator (uPA).[16,17] Several studies have shown that the expression of matriptase correlates with the progression of several tumors, including breast, lung, cervix, ovary, colon, and stomach cancers.[18–20] The expression profiles of matriptase in esophageal SCC

Address correspondence to: Jong-Shiaw Jin, MD, PhD, Department of Pathology, Tri-Service General Hospital, National Defense Medical Center, No. 325, Sec. 2, Cheng-Kung Road, Neihu 114, Taipei, Taiwan. Email: jsjin@ndmctsgh.edu.tw

© 2006 The Authors
Journal compilation © 2006 The International Society for Diseases of the Esophagus

Downloaded from https://academic.oup.com/dote/article/19/6/482/2419771 by Central South University user on 21 August 2022

remains unclear. The aim of the present study was to investigate the immunostaining patterns of EMMPRIN and matriptase in patients with esophageal SCC and correlate them with tumor differentiation and clinicopathological parameters.

MATERIALS AND METHODS

Tissue specimens

Paraffin-embedded tumor tissues were obtained from 41 patients with esophageal SCC at the Department of Pathology, Tri-Service General Hospital, Taipei, Taiwan. The study was reviewed and approved by the institution's Surveillance Committee, which allowed us to get access to patients' medical records and to obtain tissue samples. For construction of tissue microarray slides, one core was taken from selected areas of each paraffin-embedded tumor tissue block. Each representative core in tissue microarray slides was 2 mm in diameter and the pathological diagnosis in each of these cases was reviewed by at least two experienced pathologists. Sections with less than 50% of the original tissue remaining on the microarray slides after processing were excluded and not analyzed. Clinical stages were classified according to the TNM and staging system of the American Joint Committee on Cancer (AJCC). Normal esophageal tissues that included stratified squamous epithelia were obtained from 16 cases; tissues were taken at least 4 cm from the neoplasm. None of the cases had received radiation or chemotherapy before surgery.

Immunohistochemistry

Tissue microarray sections were dewaxed in xylene, rehydrated in alcohol, and immersed in 3% hydrogen peroxide for 5 min to suppress endogenous peroxidase activity. Antigen retrieval was performed by heating (100°C) each section for 30 min in 0.01 mol/L sodium citrate buffer (pH 6.0). After three rinses (each for 5 min) in phosphate-buffered saline (PBS), sections were incubated for 1 h at room temperature with a mouse antihuman EMMPRIN antibody (1 : 100, DAKO, Glostrup, Denmark) or a rabbit antihuman matriptase/ST14 antibody (1 : 100, BETHYL Laboratories, Montgomery, TX, USA) diluted in PBS. After three washes (each for 5 min) in PBS, sections were incubated with goat antimouse immunoglobulin (1 : 100, DAKO, Glostrup, Denmark) or mouse antirabbit immunoglobulin (DAKO, Glostrup, Denmark) for 30 min at room temperature. After three additional washes, peroxidase activity was developed with AEC+ substrate chromogen system (DAKO, Glostrup, Denmark) at room temperature.

Expression of EMMPRIN and matriptase

To assess EMMPRIN and matriptase expressions, the non-tumor portion of esophageal parenchyma was used as a negative internal control. Membranous and cytoplasmic staining was calculated in all tumor cells. For evaluation of immunoreactivity and histological appearance, all tissue microarray experiments were repeated three times and the slides were examined and scored by two authors concurrently. The intensity of cytoplasmic or membranous immuno-staining was analyzed on a scale of 0 (no staining), 1+ (weak intensity), 2+ (moderate intensity), and 3+ (strongest intensity), and the percentage of tumor cells staining was estimated (from 0–100%).

Statistical analysis

Extracellular matrix metalloproteinase inducer and matriptase expressions for each histopathological differentiation were compared to the immunostaining patterns of fibro-vascular stroma using the Student's *t*-test. *P*-values of < 0.05 were considered statistically significant. SigmaState software (Jandel Scientific, CA, USA) was used to perform linear regression testing to analyze the relationships between the percentage tumor staining and clinicopathological parameters of esophageal SCCs.

RESULTS

EMMPRIN expression in esophageal SCC

The study sample included 28 men and 13 women. The median age was 62.5 years and ranged from 37–80 years. EMMPRIN was expressed as parabasal staining in normal esophageal squamous epithelia in all 16 control cases (Fig. 1a,b). EMMPRIN immunoreactivity was seen in all cases with varying levels of percentage tumor staining and intensities (Table 1). In well-differentiated esophageal SCC, tumor staining was 48%, and the average EMMPRIN intensity was 2+ (Fig. 1d,e). Tumor staining was 73%, and the average intensity was 3+ in moderately-differentiated esophageal SCC (Fig. 1g,h). In poorly differentiated esophageal SCC, tumor staining was 92% and the average intensity was 3+ (Fig. 1j,k). Higher percentage tumor staining with EMMPRIN significantly correlated with poorly differentiated esophageal SCC (Fig. 2).

Matriptase expression in esophageal SCC

All 16 cases of normal esophageal squamous epithelia showed significant expression of matriptase (Fig. 1c). Among all esophageal SCC cases, matriptase immunoreactivities were present on the cell membrane

© 2006 The Authors
Journal compilation © 2006 The International Society for Diseases of the Esophagus

Downloaded from https://academic.oup.com/dote/article/19/6/482/2419771 by Central South University user on 21 August 2022

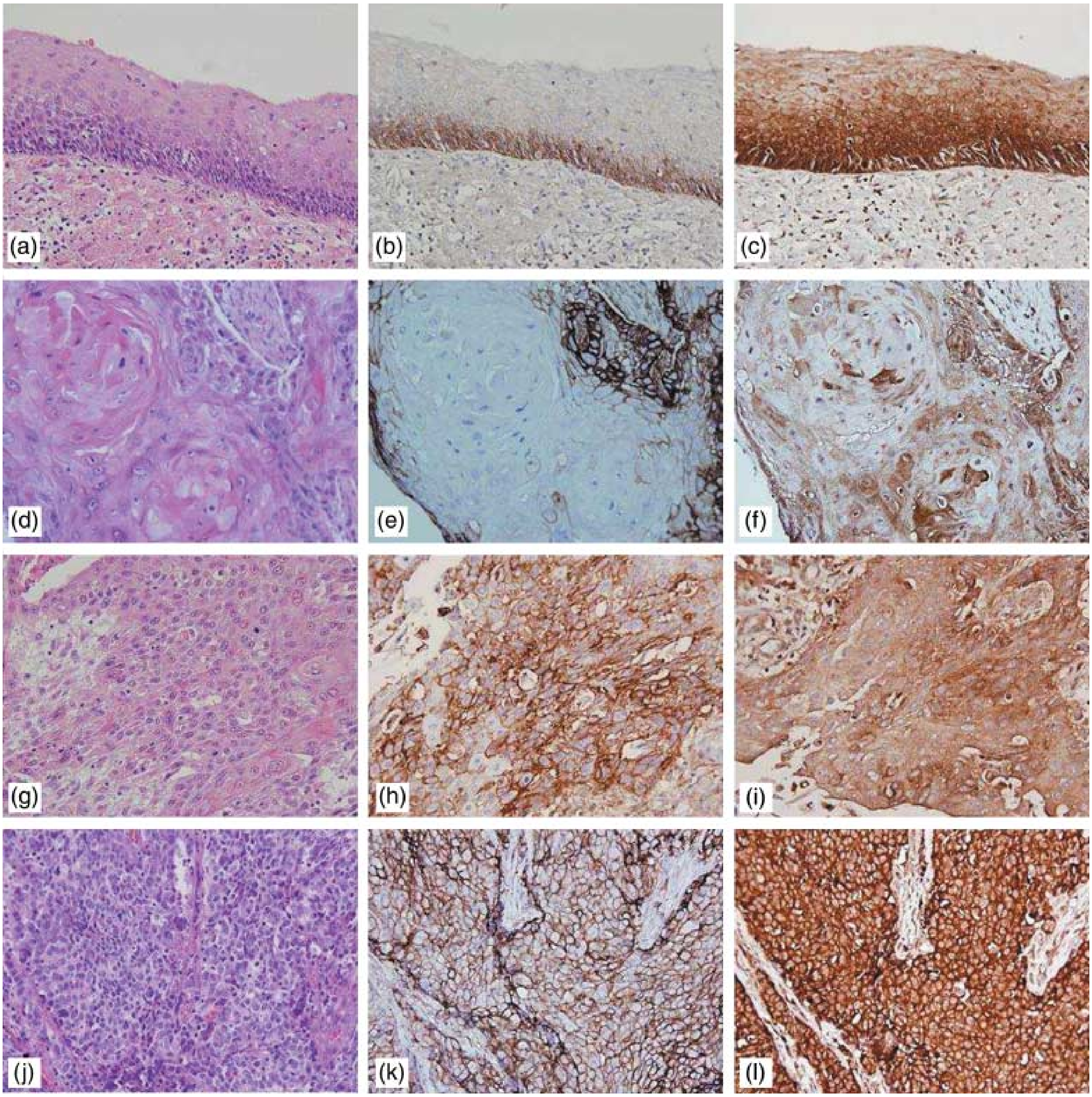

Fig. 1 Hematoxylin and eosin staining of normal esophageal epithelia (a), well differentiated (d), moderately differentiated (g), and poorly differentiated esophageal squamous cell carcinoma (j); and immunohistochemical stain of EMMPRIN in normal esophageal epithelia (b), well differentiated (e), moderately differentiated (h), and poorly differentiated esophageal squamous cell carcinoma (k); and immunohistochemical stain of matriptase in normal esophageal epithelia (c), well differentiated (f), moderately differentiated (i), and poorly differentiated esophageal squamous cell carcinoma (l). Original magnification ×400.

and in the cytoplasm at varying levels of intensity and staining percentages. In well-differentiated esophageal SCC, the percentage of tumor staining was 52%, and the average intensity of matriptase was 2+ (Fig. 1f). In moderately differentiated esophageal SCC, the percentage of tumor staining was 85%, and the average intensity was 3+ (Fig. 1i). In poorly differentiated esophageal SCC, the percentage of tumor staining was 88%, and the average intensity was 3+ (Fig. 1l). Higher percentage tumor staining with matriptase was significantly correlated with poorly differentiated esophageal SCC (Fig. 3). In addition, higher percentage tumor staining with matriptase was significantly correlated with more advanced N and M stages (Fig. 3).

DISCUSSION

We demonstrated, in tissue microarray preparation, that the expression of EMMPRIN and matriptase in esophageal SCC correlates with clinicopathological parameters. EMMPRIN is a member of cell surface glycoproteins and belongs to the immunoglobulin

© 2006 The Authors
Journal compilation © 2006 The International Society for Diseases of the Esophagus

Downloaded from https://academic.oup.com/dote/article/19/6/482/2419771 by Central South University user on 21 August 2022

Table 1 Immunostaining patterns of EMMPRIN and matriptase in esophageal squamous cell carcinomas

Esophageal SCC	% staining	Average intensity
**EMMPRIN**		
Well differentiation (*n* = 10)	48 ± 3*	2+
Moderate differentiation (*n* = 19)	73 ± 3*	3+
Poor differentiation (*n* = 12)	92 ± 3*	3+
Non-tumor part (*n* = 41)	13 ± 3	1+
**Matriptase**		
Well differentiation (*n* = 10)	52 ± 3*	2+
Moderate differentiation (*n* = 19)	85 ± 2*	3+
Poor differentiation (*n* = 12)	88 ± 3*	3+
Non-tumor part (*n* = 41)	12 ± 3	1+

Data are the average immunohistochemical intensity and percentage tumor staining of Extracellular matrix metalloproteinase inducer (EMMPRIN) and matriptase in esophageal squamous cell carcinoma (SCC) and non-tumor esophageal fibrovascular tissue. *Indicates statistical significance compared with non-tumor part fibro-vascular tissue ($P < 0.05$).

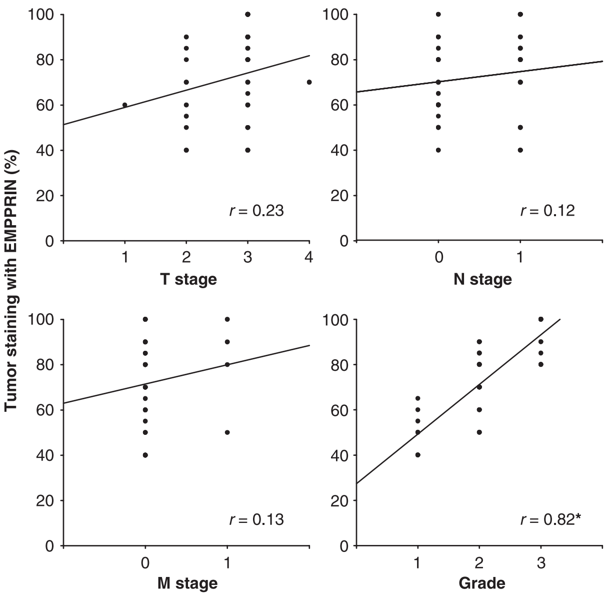

Fig. 2 Clinicopathological correlations with the percentage tumor staining with EMMPRIN in esophageal squamous cell carcinoma. *Indicates statistical significance of linear regression testing ($P < 0.05$).

superfamily.[13,21] The glycoprotein has been known to express on the surface of several malignant cells and play a role in stimulation of several matrix metalloproteinases.[13,22,23] Several studies have shown that the expression of EMMPRIN in tumor cells is associated with malignancy and/or poor prognosis in several tumors including esophageal, serous ovarian, breast, laryngeal carcinomas and glioma.[3,13,14,23–25] In the present study, we demonstrated that higher percentage tumor staining with EMMPRIN is significantly associated with poorly differentiated esophageal SCC.

We found no correlation between the percentage tumor staining with EMMPRIN and the TNM stages. Our finding is consistent with Ishibashi *et al.*

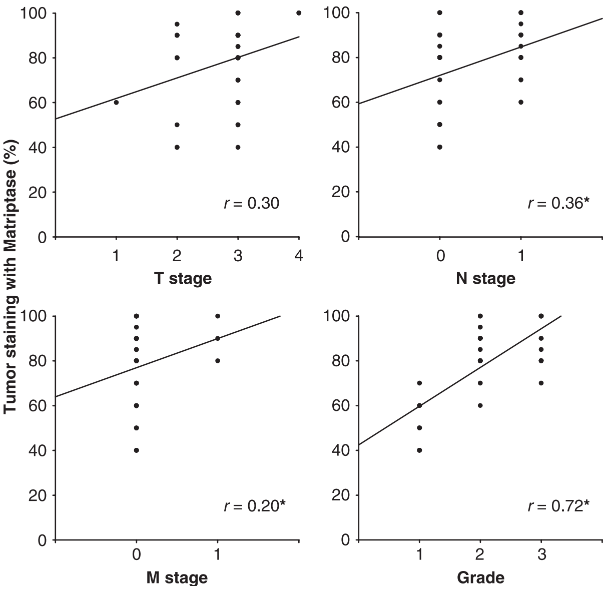

Fig. 3 Clinicopathological correlations with the percentage tumor staining with matriptase in esophageal squamous cell carcinoma. *Indicates statistical significance of linear regression testing ($P < 0.05$).

who evaluated EMMPRIN staining patterns using a three-level scale (none, partial and diffuse staining) on individual tissues.[3] This scale may have limited the quantitative analysis of immunohistochemical intensity due to instability of the signal generated by a chemical reaction under different environmental conditions.[3] In our study, all the tumor tissues were placed in tissue microarray slides which has recently been recognized as a powerful histological and immunohistochemical tool for simultaneous evaluation of tumors.[26] The reliability of immunohistochemistry studies conducted in tissue microarray slides has been established.[27]

We also demonstrated that the percentage tumor staining with matriptase in esophageal SCC was significantly correlated with histopathologic differentiation and with N and M stages. Matriptase was initially isolated as a trypsin-like serine proteinase from breast carcinoma cells.[28] This protease has been shown to act as an upstream activator of uPA and HGF, which is thought to play a role in tumor progression.[16,17] Matriptase has been known to express in breast, lung, cervix, ovary, colon, and stomach cancers[18–20] and be correlated with tumor progression in ovarian, cervical and breast carcinomas.[19,29,30] Our current results support the concept that matriptase is a satisfactory biomarker to predict the invasiveness of esophageal SCC.

In conclusion, our study demonstrates that the expression of EMMPRIN and matriptase are both effective in predicting histopathologic differentiation of esophageal SCC. In addition, higher expression of matriptase was significantly associated with advanced clinicopathological stages of esophageal

© 2006 The Authors
Journal compilation © 2006 The International Society for Diseases of the Esophagus

Downloaded from https://academic.oup.com/dote/article/19/6/482/2419771 by Central South University user on 21 August 2022

SCC. Pharmacological agents targeting EMMPRIN and matriptase expressions may be beneficial in the treatment of esophageal SCC.

Acknowledgments

This study was supported by grants from National Science Counsel, SC94-2314-B-016–025, and Tri-Service General Hospital, TSGH-C95-S04, Taiwan, R.O.C.

References

1 Pisani P, Parkin D M, Ferlay J. Estimates of the worldwide mortality from eighteen major cancers in 1985. Implications for prevention and projections of future burden. Int J Cancer 1993; 55: 891–903.
2 Landis S H, Murray T, Bolden S, Wingo P A. Cancer statistics. CA Cancer J Clin 1999; 49: 8–31.
3 Ishibashi Y, Matsumoto T, Niwa M *et al.* CD147 and matrix metalloproteinase-2 protein expression as significant prognostic factors in esophageal squamous cell carcinoma. Cancer 2004; 101: 1994–2000.
4 Samantaray S, Sharma R, Chattopadhyaya T K, Gupta S D, Ralhan R. Increased expression of MMP-2 and MMP-9 in esophageal squamous cell carcinoma. J Cancer Res Clin 2004; 130: 37–44.
5 Yang G Z, Li L, Ding H Y, Zhou J S. Cyclooxygenase-2 is over-expressed in Chinese esophageal squamous cell carcinoma, and correlated with NF-kappaB: an immunohistochemical study. Exp Mol Pathol 2005; 79: 214–18.
6 Kelsen D P, Ginsberg R, Pajak T F *et al.* Chemotherapy followed by surgery compared with surgery alone for localized esophageal cancer. N Engl J Med 1998; 339: 1979–84.
7 Law S, Kwong D L, Kwok K F *et al.* Improvement in treatment results and long-term survival of patients with esophageal cancer: impact of chemoradiation and change in treatment strategy. Ann Surg 2003; 238: 339–47.
8 Sarbia M, Verreet P, Bittinger F *et al.* Basaloid squamous cell carcinoma of the esophagus: diagnosis and prognosis. Cancer 1997; 79: 1871–8.
9 Kawakubo H, Ozawa S, Ando N *et al.* Alterations of p53, cyclin D1 and pRB expression in the carcinogenesis of esophageal squamous cell carcinoma. Oncol Rep 2005; 14: 1453–9.
10 Kuwano H, Kato H, Miyazaki T *et al.* Genetic alterations in esophageal cancer. Surg Today 2005; 35: 7–18.
11 Mandard A M, Hainaut P, Hollstein M. Genetic steps in the development of squamous cell carcinoma of the esophagus. Mutat Res 2000; 462: 335–42.
12 Hara T, Kijima H, Yamamoto S *et al.* Ubiquitous p63 expression in human esophageal squamous cell carcinoma. Int J Mol Med 2004; 14: 169–73.
13 Li Y, Stanley Z, Toole B P. Roles of the multifunctional glycoprotein, emmprin (basigin; CD147), in tumour progression. Thromb Haemost 2005; 93: 199–204.
14 Davidson B, Goldberg I, Berner A, Kristensen G B, Reich R. EMMPRIN (extracellular matrix metalloproteinase inducer) is a novel marker of poor outcome in serous ovarian carcinoma. Clin Exp Metastasis 2003; 20: 161–9.
15 Reimers N, Zafrakas K, Assmann V *et al.* Expression of extracellular matrix metalloproteases inducer on micrometastatic and primary mammary carcinoma cells. Clin Cancer Res 2004; 10: 3422–8.
16 Lee S L, Dickson R B, Lin C Y. Activation of hepatocyte growth factor and urokinase/plasminogen activator by matriptase, an epithelial membrane serine protease. J Biol Chem 2000; 275: 36720–5.
17 Takeuchi T, Harris J L, Huang W, Yan K W, Coughlin S R, Craik C S. Cellular localization of membrane-type serine protease 1 and identification of protease-activated receptor-2 and single-chain urokinase-type plasminogen activator as substrates. J Biol Chem 2000; 275: 26333–42.
18 Oberst M, Anders J, Xie B *et al.* Matriptase and HAI-1 are expressed by normal and malignant epithelial cells *in vitro* and *in vivo*. Am J Pathol 2001; 158: 1301–11.
19 Oberst M D, Johnson M D, Dickson R B *et al.* Expression of the serine protease matriptase and its inhibitor HAI-1 in epithelial ovarian cancer: correlation with clinical outcome and tumor clinicopathological parameters. Clin Cancer Res 2002; 8: 1101–7.
20 Jin X, Hirosaki T, Lin C Y *et al.* Production of soluble matriptase by human cancer cell lines and cell surface activation of its zymogen by trypsin. J Cell Biochem 2005; 95: 632–47.
21 Miyauchi T, Kanekura T, Yamaoka A, Ozawa M, Miyazawa S, Muramatsu T. Basigin, a new, broadly distributed member of the immunoglobulin superfamily, has strong homology with both the immunoglobulin V domain and the beta-chain of major histocompatibility complex class II antigen. J Biochem 1990; 107: 316–23.
22 Guo H, Zucker S, Gordon M K, Toole B P, Biswas C. Stimulation of matrix metalloproteinase production by recombinant extracellular matrix metalloproteinase inducer from transfected Chinese hamster ovary cells. J Biol Chem 1997; 272: 24–7.
23 Dalberg K, Eriksson E, Enberg U, Kjellman M, Backdahl M. Gelatinase A, membrane type 1 matrix metalloproteinase, and extracellular matrix metalloproteinase inducer mRNA expression: correlation with invasive growth of breast cancer. World J Surg 2000; 24: 334–40.
24 Rosenthal E L, Shreenivas S, Peters G E, Grizzle W E, Desmond R, Gladson C L. Expression of extracellular matrix metalloproteinase inducer in laryngeal squamous cell carcinoma. Laryngoscope 2003; 113: 1406–10.
25 Sameshima T, Nabeshima K, Toole B P *et al.* Expression of emmprin (CD147), a cell surface inducer of matrix metalloproteinases, in normal human brain and gliomas. Int J Cancer 2000; 88: 21–7.
26 Lam S, Belldegrum A S, Figlin R A. Tissue array-based predictions of pathobiology, prognosis and response to treatment for renal cell carcinoma therapy. Clin Cancer Res 2004; 10: 6304s–9s.
27 Gerdes J, Lemke H, Baisk H *et al.* Cell cycle analysis of the cell proliferation associated human nuclear antigen defined by the monoclonal antiboby Ki67. J Immunol 1984; 133: 1710–5.
28 Lin C Y, Wang J K, Torri J, Dou L, Sang Q A, Dickson R B. Characterization of a novel, membrane-bound, 80-kDa matrix-degrading protease from human breast cancer cells. J Biol Chem 1997; 272: 9147–52.
29 Lee J W, Song S Y, Choi J J *et al.* Increased expression of matriptase is associated with histopathologic grades of cervical neoplasia. Hum Pathol 2005; 36: 626–33.
30 Kang J Y, Dolled-Filhart M, Ocal I T *et al.* Tissue microarray analysis of hepatocyte growth factor/Met pathway components reveals a role for Met, matriptase, and hepatocyte growth factor activator inhibitor 1 in the progression of node-negative breast cancer. Cancer Res 2003; 63: 1101–5.

Downloaded from https://academic.oup.com/dote/article/19/6/482/2419771 by Central South University user on 21 August 2022

© 2006 The Authors
Journal compilation © 2006 The International Society for Diseases of the Esophagus
