# Supplementary material for: Expression of Concern: The prognostic and clinicopathologic characteristics of CD147 and esophagus cancer: A meta-analysis
Source: PLoS One. 2023 Feb 22;18(2):e0282229. doi: 10.1371/journal.pone.0282229 (PMC9946197; doi:10.1371/journal.pone.0282229)
Supplement: S1 File — (ZIP) [file pone.0282229.s001.zip › PDF of included paper/╗∙╓╩╜≡╩⌠╡░░╫├╕-9íó╜≡╩⌠╡░...╩│╣▄┴█╫┤╧╕░√░⌐╫Θ╓»╓╨╡─▒φ┤∩_╞δ▓⌐.pdf]

基质金属蛋白酶－9、金属蛋白酶抑制因子－1及CD147在食管鳞状细胞癌组织中的表达

齐博，师启众，刘尚国，赵宝生
（新乡医学院第一附属医院胸外科，河南 卫辉 453100）

摘要：目的　探讨基质金属蛋白酶－9(MMP－9)、金属蛋白酶抑制因子－1(TIMP－1)及CD147在食管鳞状细胞癌(鳞癌)的表达及意义。**方法**　选择2005年1月～2007年1月在新乡医学院第一附属医院胸心外科行食管癌根治术切除的标本52例，术后均经病理证实为食管鳞癌。采用免疫组织化学方法检测食管癌组织及癌旁正常食管组织中MMP－9、TIMP－1及CD147的表达。**结果**　食管鳞癌组织中MMP－9、TIMP－1及CD147的阳性表达率分别为73.0%、44.2%和80.8%，与癌旁正常食管组织(23.1%、30.8%、19.2%)比较有显著性差异($P<0.05$)。鳞癌组织中MMP－9阳性表达率与TIMP－1无明显相关性($P>0.05$)，与CD147成正相关($r=0.457,P<0.05$)。MMP－9阳性表达率与肿瘤浸润及淋巴结转移有关($P<0.05$)。**结论**　食管鳞癌组织中MMP－9/TIMP－1失衡参与了其侵袭转移过程，CD147可能作为诱导剂促进了MMP－9的表达。

关键词： 基质金属蛋白酶－9；金属蛋白酶抑制因子－1；CD147；食管鳞状细胞癌
中图分类号：R735.1　　**文献标志码：**A　　**文章编号：**1006－1959(2008)10－0886－03

Expression of matrix metalloproteinase－9, tissue inhibitor of metalloproteinase－1 and CD147 in esophageal squamous cell cancer

QI Bo, SHI Qi－zhong, LIU Shang－guo, et al
(Department of Thoracic Surgery, the First Affiliated Hospital of Xinxiang Medical University, Weihui 453100, Henan Province, China)

Abstract: Objective　To investigate expression of matrix metalloproteinase－9 (MMP－9), tissue inhibitor of metalloproteinase－1 (TIMP－1) and CD147 in esophageal squamous cell cancer (ESCC) and their significance. **Methods**　Fifty－two patients with esophageal carcinoma and had total correction in the First Affiliated Hospital of Xinxiang Medical University from Jan. 2005 to Jan. 2007. All the patients were confirmed to be ESCC. Immunohistochemistry was used to detect the expression of MMP－9, TIMP－1 and CD147 protein of dysplasia tissues and normal esophageal mucosa tissues in the patients. **Results**　The expression rates of MMP－9, TIMP－1 and CD147 protein were 73.0%, 44.2% and 80.8% respectirely, which has significant difference compared with normal tissue (23.1%, 30.8% and 19.2%, $P<0.05$). There was positive correlation between the expression rates of MMP－9 and CD147 ($r=0.457$, $P<0.05$) and no correlation between MMP－9 and TIMP－1 ($P>0.05$). **Conclusions**　The imbalance of MMP/TIMP is closely correlated to the lymphnode invasion and metastasis of ESCC, CD147 may be the revulsivum of MMP－9 and promote the expression of MMP－9.

Key words: matrix metalloproteinase－9; tissue inhibitor of metalloproteinase－1; CD147; esophageal squamous cell cancer

食管鳞状细胞癌(鳞癌)是我国常见消化道恶性肿瘤之一，其侵袭转移是一个复杂的过程。基质金属蛋白酶(MMP)是肿瘤侵袭转移过程中较为重要的一类蛋白水解酶，可降解细胞外基质(ECM)，从而解除肿瘤侵袭转移的天然屏障。金属蛋白酶抑制因子－1(TIMP－1)是MMP－9的特异性抑制物[1]。研究发现，MMP－9/TIMP－1失衡可能与肿瘤侵袭转移有关。CD147是肿瘤细胞表明的MMP诱导因子，在体内外恶性肿瘤中均可高表达[2]。本研究检测了人食管鳞癌组织中MMP－9、TIMP－1及CD147的表达，探讨其在食管鳞癌侵袭转移中的作用及相互关系，报告如下。

1　资料与方法

1.1　一般资料

收稿日期：2008－7－10

(C)1994-2022 China Academic Journal Electronic Publishing House. All rights reserved.　http://www.cnki.net

选择 2005 年 1 月 ~2007 年 1 月新乡医学院第一附属医院胸心外科确诊为食管癌并行食管癌根治术的患者 52 例,男 36 例,女 16 例,年龄 42 ~69 岁,平均(58.2 ±9.3)岁。所有患者术前均无放疗、化疗及免疫治疗史,术后均经病理证实为食管鳞癌。术中取癌组织及癌旁正常组织。

1.2 主要试剂

鼠抗人 MMP -9、TIMP -1 及 CD147 单克隆抗体及免疫组织化学试剂盒、DAB 显色试剂盒购自武汉博士德生物公司。

1.3 方法

所有癌组织及癌旁正常组织标本经 10% 甲醛固定、石蜡包埋、4 μm 切片。MMP -9、TIMP -1 及 CD147 的检测严格按说明书操作。以已知食管鳞癌阳性标本作阳性对照,用 PBS 代替一抗作阴性对照。以胞质内出现棕黄色颗粒为阳性,根据阳性细胞所占百分比分为:阴性(-):无阳性细胞染色;弱阳性(+):阳性癌细胞小于癌细胞总数 50% 或显色浅;强阳性(++):阳性细胞大于 50% 或显色深。

1.5 统计学处理

应用 SPSS 10.0 软件行卡方检验和直线相关性分析。$P<0.05$ 为差异有统计学意义。

2 结果

2.1 食管鳞癌组织及正常食管黏膜组织中 MMP -9、TIMP -1 及 CD147 的表达 见表 1。

表 1 人食管鳞癌组织及正常食管黏膜组织中 MMP -9、TIMP -1 及 CD147 阳性表达率[n(%)]

部位	n	MMP -9	TIMP -1	CD147
癌组织	52	38(73.0)	23(44.2)	42(80.8)
正常组织	52	12(23.1)	16(30.8)	10(19.2)
P		<0.05	<0.05	<0.01

2.2 MMP -9、TIMP -1 及 CD147 表达阳性与食管鳞癌肿瘤浸润及淋巴结转移的关系 见表 2。

表 2 MMP -9、TIMP -1 及 CD147 表达阳性与食管鳞癌肿瘤浸润及淋巴结转移的关系[n(%)]

临床病理特征	n	MMP -9	TIMP -1	CD147
浸润至黏膜及黏膜下	12	2(16.7)	8(66.7)	3(25.0)
浸润至肌和外膜层	40	34(85.0)	6(15.0)	29(72.5)
P		<0.05	<0.05	<0.05
有淋巴结转移	15	13(86.7)	4(26.7)	11(73.3)
无淋巴结转移	37	3(20.0)	28(75.7)	4(10.8)
P		<0.05	<0.05	<0.01

2.3 MMP -9 与 TIMP -1 及 CD147 相关性分析

人食管鳞癌组织中 MMP -9 阳性表达率与 TIMP -1 无明显相关性($P>0.05$),与 CD147 成正相关($r=0.457$, $P<0.05$)。

3 讨论

肿瘤侵袭转移是癌症患者死亡的主要原因之一,ECM 是肿瘤侵袭转移时的天然屏障,肿瘤细胞及其基质细胞分泌蛋白酶降解 ECM,使基底膜产生局部的缺损,肿瘤细胞由此方式穿过血管的基底膜缺损处进入血管,形成转移。MMPs 是一类 Zn^{2+} 依赖特异性蛋白水解酶,通常由肿瘤细胞和(或)基质成纤维细胞合成并分泌到组织中,能在中性 pH 条件下降解 ECM。MMP -9 属于明胶酶类,又称为明胶酶 B,可有效降解基底膜的主要成分Ⅳ、Ⅴ、Ⅵ、Ⅹ型胶原,从而破坏基底膜的完整性[1,3]。TIMP -1 可与活化的 MMP -9 以 1:1 非共价键结合,从而阻断 MMP -9 与底物结合,抑制 MMP -9 对 ECM 的降解,进而抑制肿瘤的浸润和转移[2,4]。本研究结果显示,食管鳞癌 MMP -9 阳性表达率为 73.0%,较癌旁组织(23.1%)显著增高,MMP -9 在肿瘤侵犯黏膜及黏膜下层时的阳性率较侵犯至肌层、外膜层时显著降低($P<0.05$)。有淋巴结转移组的 MMP -9 阳性率显著高于无淋巴结转移组($P<0.05$),提示 MMP -9 不仅与食管癌的发生、发展关系密切,而且在食管癌的浸润转移过程中起重要作用。本研究发现 TIMP -1 在食管癌组织中的表达与浸润深度及有无淋巴结转移密切相关($P<0.05$),可能预示在肿瘤不断深入浸润及转移过程中,并不一定为 MMP -9 的分泌量持续升高及 TIMP -1 的分泌合成不断减少所致,而是由于 MMP -9 升高的比例大大超过了 TIMP -1 升高的比例,从而导致 MMP -9/TIMP -1 比例失衡的结果。相关性分析显示 MMP -9 阳性表达率与 TIMP -1 无相关性也支持这一推测。CD147 属于免疫球蛋白超家族的成员之一,是一种新的细胞表面黏附分子,介导细胞与细胞、细胞与间质的黏附作用[5]。CD147 在体内分布非常广泛,参与人体的多种生理活动,如胚胎着床、子宫复旧及伤口愈合等,但其在肿瘤侵袭和转移中的作用更值得关注。CD147 作为细胞外 MMP 刺激物,具有刺激肿瘤细胞及周围间质成纤维细胞分泌Ⅳ型胶原酶的能力,促进肿瘤细胞的转移。本研究结果显示,食管癌组织中 CD147 阳性表达率较癌旁组织显著升高($P<0.05$),随着癌组织浸润

(C)1994-2022 China Academic Journal Electronic Publishing House. All rights reserved. http://www.cnki.net

深度的增加及淋巴结的转移其表达亦增强,提示CD147可能参与了食管鳞癌的发生、侵袭及转移。

Guo等[6]研究发现,CD147具有刺激肿瘤细胞周围间质成纤维细胞分泌Ⅳ型胶原酶(MMP-9)的能力,促进肿瘤的转移。本研究结果表明,MMP-9阳性表达率与CD147成正相关($r=0.457$, $P<0.05$),推测在食管鳞癌的浸润和转移中,二者可能存在相互作用,提示CD147可能作为诱导因子促进MMP-9的合成和分泌,二者的协同作用共同促进ECM恶性细胞的增殖和侵袭转移。

本研究结果表明,食管鳞癌组织中MMP-9/TIMP-1失衡参与了其侵袭转移过程,CD147可能作为诱导剂促进了MMP-9的表达。MMP-9/TIMP-1及CD147可能作为衡量肿瘤恶性程度的潜在指标,为肿瘤诊断、评价预后及化学治疗提供了一个新的思路。

[参考文献]

[1] Suzuki T, Kuwabara Y, Iwata H, et al. Role of matrix metalloproteinase-9 in in vitro invasion of esophageal carcinoma cells[J]. J Surg Oncol, 2002, 81:80-86.

[2] Kähäri VM, Saarialho-Kere U. Matrix metalloproteinases and their inhibitors in tumour growth and invasion [J]. Ann Med, 1999, 31:34-45.

[3] Mroczko B, Kozowski M, Groblewska M, et al. The diagnostic value of the measurement of matrix metalloproteinase 9 (MMP-9), squamous cell cancer antigen (SCC) and carcinoembryonic antigen (CEA) in the sera of esophageal cancer patients[J]. Clin Chim Acta, 2008, 389:61-66.

[4] Herszenyi L, Hritz I, Pregun I, et al. Alterations of glutathione S-transferase and matrix metalloproteinase-9 expressions are early events in esophageal carcinogenesis[J]. World J Gastroenterol, 2007, 13:676-682.

[5] Cheng MF, Tzao C, Tsai WC, et al. Expression of EMMPRIN and matriptase in esophageal squamous cell carcinoma: correlation with clinicopathological parameters[J]. Dis Esophagus, 2006, 19:482-486.

[6] Guo H, Li R, Zucker S, et al. EMMPRIN (CD147), an inducer of matrix metalloproteinase synthesis, also binds interstitial collagenase to the tumor cell surface[J]. Cancer Res, 2000, 60:888-891.

(本文编辑:耿永涛)

利多卡因气管粘膜表面麻醉对全麻病人血流动力学的影响

晁葳

(登封市中医院麻醉科,河南 登封 452470)

摘要:目的 评价利多卡因气管粘膜表面麻醉对全麻患者气管插管时心血管反应的影响。**方法** 选择全麻手术患者30例,年龄26~70岁,体重45~77 kg,ASA Ⅰ~Ⅱ级,随机分为2组($n=15$):利多卡因气管粘膜表面麻醉组(L组)和对照组(C组)。两组均全麻诱导依次静脉注射咪唑安定0.1 mg·kg^{-1}芬太尼3 μg·kg^{-1},依托咪酯0.3 mg·kg^{-1},琥珀胆碱2 mg·kg^{-1},肌松完善后喉镜显露声门,L组插入气管喷雾器注入2%利多卡因5 ml,然后气管内插管;C组直接气管内插管,机械通气。分别于麻醉诱导前(T_0),麻醉诱导后(T_1),气管插管后即刻(T_2),气管插管后5 min(T_3),10 min(T_4)记录收缩压(SBP)、舒张压(DBP)、平均动脉压(MAP)、心率(HR)。**结果** L组在T_2时SBP与C组比较差异有统计学意义,SBP明显低于C组$P<0.05$。组内比较L组T_0与T_2、T_3比较SBP、DBP、MAP相比差异有统计学意义$P<0.05$,T_0与T_4比较SBP降低,相比差异有统计学意义$P<0.05$;T_1与T_3比较SBP在T_3时明显降低,两者相比差异有统计学意义$P<0.05$。C组T_0与T_2比较,T_2时SBP、DBP、MAP明显降低,两者相比差异有统计学意义$P<0.05$,T_0与T_4比较T_4时SBP明显下降,两者相比差异有统计学意义$P<0.05$;T_1与T_3比较,T_3时DBP、MAP明显下降,两者相比差异有统计学意义$P<0.05$。**结论** 利多卡因气管粘膜喷射表面麻醉可适当降低全麻患者气管插管时的心血管反应,但起效时间稍慢。如要真正降低气管插管时的心血管反应,还应该依赖各种静脉诱导药的复合作用。

关键词: 利多卡因;气管粘膜表面麻醉;气管插管;心血管反应

中图分类号:R971$^+$.2　**文献标志码:**A　**文章编号:**1006-1959(2008)10-0888-03

收稿日期:2008-7-28

(C)1994-2022 China Academic Journal Electronic Publishing House. All rights reserved. http://www.cnki.net
