# Supplementary material for: Expression of Concern: The prognostic and clinicopathologic characteristics of CD147 and esophagus cancer: A meta-analysis
Source: PLoS One. 2023 Feb 22;18(2):e0282229. doi: 10.1371/journal.pone.0282229 (PMC9946197; doi:10.1371/journal.pone.0282229)
Supplement: S1 File — (ZIP) [file pone.0282229.s001.zip › PDF of included paper/╗∙╓╩╜≡╩⌠╡░░╫├╕2╝░CD147╘┌╩│╣▄┴█╫┤╧╕░√░⌐╓╨╡─▒φ┤∩╝░╥Γ╥σ.pdf]

# 基质金属蛋白酶 2 及 CD147 在食管鳞状细胞癌中的表达及意义

赵建华, 樊克武

(南京医科大学附属南京第一医院病理科,江苏 南京 210006)

[摘 要] 目的:探讨食管鳞癌基质金属蛋白酶 2(MMP-2)和 CD147 表达及其与癌浸润转移的关系。方法:应用免疫组化 S-P 法观察 70 例食管鳞癌组织中 MMP-2 和 CD147 的表达,并探讨其与临床病理资料的关系。结果:在低分化、中和高分化食管鳞癌中 MMP-2 和 CD147 的阳性率分别为 90.5%、87.8%及 81.0%、73.5%。在食管鳞癌中,MMP-2 及 CD147 的表达均与食管鳞癌的浸润深度有关,与分化程度无明显关联;有淋巴结转移的病例阳性表达率明显高于无淋巴结转移组。结论:MMP-2 和 CD147 均与食管鳞癌的浸润深度及淋巴结转移有关。

[关键词] 食管肿瘤; 基质金属蛋白酶-2; CD147; 免疫组化

[中图分类号] 735.1 [文献标识码] A [文章编号] 1007-4368(2004)06-0621-03

## Expression and Significance of Matrix Metalloproteinase-2 and CD147 in Esophageal Squamous Cell Carcinoma

ZHAO Jian-hua, FAN Ke-wu

*(Department of Pathology, the Affiliated Nanjing First Hospital of NJMU, Nanjing 210006, China)*

[**Abstract**] **Objective**: To investigate the expression of matrix metalloproteinase-2 (MMP-2)and CD147 in esophageal squamous cell carcinoma. **Methods**:The expression of MMP-2 and CD147 was detected in esophageal squamous cell carcinoma by immunohistochemical SP method. **Results**:In esophageal squamous cell carcinoma, the expression of MMP-2 and CD147 had a significant relevance to infiltrating depth and lymph node metastasis, but no association was observed with pathological grades. **Conclusion**:Both MMP-2 and CD147 have relevance to infiltrating depth and lymph node metastasis of esophageal squamous cell carcinoma.

[**Key words**] esophageal carcinoma; matrix metalloproteinase-2; CD147; immunohistochemistry

[Acta Univ Med Nanjing, 2004, 24(6): 621-623]

食管鳞癌是消化道常见恶性肿瘤,多数患者就诊时已属晚期,常有深度浸润及转移,死亡率很高。癌细胞从原位增殖到浸润转移的过程中,必需具备降解细胞外基质的能力, 因此产生能降解细胞外基质及基膜的酶与食管鳞癌的转移密切相关。基质金属蛋白酶(matrix metalloproteinase-2,MMP-2)由于能降解细胞外基质的主要成分Ⅳ型胶原,被认为与肿瘤的浸润转移和预后密切相关[1]。而 CD147 由于能刺激 MMP-2 的产生也有利于肿瘤细胞的转移。本文收集本院外科 2002~2003 年手术切除的食管鳞癌标本 70 例, 用免疫组化法检测食管鳞癌 MMP-2 和 CD147 的表达, 并探讨 MMP-2 及 CD147 与食管鳞癌浸润转移的相关性。

## 1 资料和方法

### 1.1 一般资料

本组 70 例食管鳞癌中,男性 52 例,女性 18 例;年龄 38~80 岁,平均 54.6 岁。主要症状为进行性吞咽困难。所有患者术前均未做化疗和放疗。标本经 10%中性福尔马林固定,石蜡包埋,连续切片 4 μm 厚,苏木精-伊红(HE)染色。

### 1.2 方法

免疫组化染色采用 S-P 法,单克隆抗 MMP-2 抗体及抗 CD147 抗体购自福州迈新生物技术公司,试剂盒为美国 Maxim 公司产品, 严格按说明书操作,DAB 显色。用缓冲液代替一抗作阴性对照,以已知

(C)1994-2022 China Academic Journal Electronic Publishing House. All rights reserved. http://www.cnki.net

食管鳞癌阳性标本作阳性对照。结果判断标准：MMP-2以胞质呈清晰棕黄色为阳性，CD147以胞膜及胞质呈棕黄色为阳性。根据显色强度及范围分为：阴性(－)：无阳性细胞染色；弱阳性(+)：阳性癌细胞小于癌细胞总数的50%或显色浅；强阳性(++)：阳性细胞大于50%或显色深。

1.3 统计学方法

研究中所得数据均采用 $\chi^2$ 检验分析。

## 2 结 果

2.1 组织学观察结果

食管鳞癌组织呈巢状分布，为多少不等的纤维组织分隔，癌细胞呈多角形，细胞边界较清楚，细胞核呈圆形或卵圆形，位于细胞中央，深染。根据分化程度，鳞癌可分为高分化、中分化和低分化。高分化鳞癌细胞有明显角化现象，胞质丰富，核分裂象不多，细胞多形性不明显；低分化鳞癌细胞不见角化，癌细胞呈梭形或长椭圆形，核分裂象多，细胞多形性明显；中分化鳞癌的组织形态界于高分化和低分化鳞癌之间。

2.2 免疫组化结果

MMP-2及CD147在食管鳞癌中的表达结果见表1及图1~4(彩页2)。MMP-2表达在食管鳞癌癌细胞的胞质，呈棕黄色，MMP-2在低分化鳞癌及中高分化鳞癌中的阳性表达率分别为90.5%和87.8%，低分化与中高分化组阳性表达率相比差异无显著性($P$ >0.05)。CD147的表达为胞质、胞膜型，在低分化鳞癌及中高分化鳞癌中的阳性表达率为81.0%和73.5%，低分化与中高分化组阳性表达率相比差异也无显著性($P$ > 0.05)。

2.3 MMP-2及CD147与食管鳞癌浸润深度的关系

70例食管鳞癌中，MMP-2及CD147的表达与浸润深度的关系见表1。其中MMP-2在黏膜及黏膜下层与肌层、黏膜及黏膜下层与外膜的阳性表达率差异有显著性($P$ < 0.05)，但在肌层与外膜的阳性表达率差异无显著性。同样，CD147在黏膜及黏膜下层与肌层、黏膜及黏膜下层与外膜的阳性表达率差异也呈显著性($P$ < 0.01)，但在肌层与外膜的阳性表达率差异无显著性。

2.4 MMP-2及CD147与淋巴结转移的关系

70例食管鳞癌中，MMP-2及CD147的表达与淋巴结转移的关系见表1。有淋巴结转移的癌组织MMP-2及CD147的阳性表达率均明显高于无淋巴结转移组($P$ < 0.01)。

**表1 MMP-2和CD117在食管鳞癌中的表达** (例)

| | MMP-2 | | | | | CD147 | | | |
|---|---|---|---|---|---|---|---|---|---|
| | $n$ | － | + | ++ | 阳性率(%) | － | + | ++ | 阳性率(%) |
| 分化程度 | | | | | | | | | |
| 低分化癌 | 21 | 2 | 7 | 12 | 90.5* | 4 | 3 | 14 | 81.0* |
| 中高分化癌 | 49 | 6 | 23 | 20 | 87.8 | 13 | 8 | 28 | 73.5 |
| 浸润深度 | | | | | | | | | |
| 黏膜及黏膜下层 | 14 | 5 | 5 | 4 | 64.3** | 11 | 1 | 2 | 21.4△△ |
| 肌层 | 26 | 2 | 16 | 8 | 92.3△ | 3 | 5 | 18 | 88.5△ |
| 外膜 | 30 | 1 | 9 | 20 | 96.7 | 3 | 5 | 22 | 90.0 |
| 淋巴结转移 | | | | | | | | | |
| 阴性组 | 37 | 8 | 23 | 6 | 78.4 | 16 | 8 | 13 | 56.8 |
| 阳性组 | 33 | 0 | 7 | 26 | 100.0*** | 1 | 3 | 29 | 97.0*** |

与中高分化癌比较，$^*P$ > 0.05；与肌层及外膜比较，$^{**}P$ < 0.05；与阴性组比较，$^{***}P$ < 0.01；与外膜比较，$^{\triangle}P$ > 0.05；与肌层及外膜比较，$^{\triangle\triangle}P$ < 0.01。

2.5 MMP-2与CD147阳性表达的关系

MMP-2及CD147的联合表达率为64.5%，但MMP-2的阳性表达率为88.6%，明显高于CD147的阳性表达率75.7%($P$ < 0.05)。

## 3 讨 论

基质金属蛋白酶(MMPs)是一个锌离子依赖性的蛋白质酶家族，从1962年发现MMPs家族的第一个成员起[2]，至今已发现20种以上的MMPs[3]；MMP-2是其中的一种，它以无活性的酶原形式分泌，激活后能降解一种或几种细胞外基质成分，包括基膜的主要成分——Ⅳ型胶原[4]。因此在肿瘤的转移中具有重要作用。当MMP-2表达升高时，肿瘤的转移能力增强[5]。本组结果表明，MMP-2表达与食管鳞癌浸润深度和淋巴结转移呈正相关，提示MMP-2在癌转移中起重要作用，这与国外许多研究结果一

(C)1994-2022 China Academic Journal Electronic Publishing House. All rights reserved. http://www.cnki.net

致[6,7]。但在不同分化程度的食管鳞癌中 MMP-2 的表达无明显差异，这一结果可以解释分化较好的食管鳞癌组织却常见发生胸腔扩散及淋巴结转移。

CD147 是相对分子质量为 50~60 ku 的跨膜糖蛋白，为免疫球蛋白超家族成员，主要功能为参与细胞-细胞或细胞-基质的黏附。CD147 作为细胞外基质金属蛋白酶刺激物，具有刺激肿瘤细胞及周围间质成纤维细胞分泌Ⅵ型胶原酶的能力，促进肿瘤细胞的转移[8]。本组结果显示，随着癌组织浸润深度的增加及淋巴结的转移，CD147 的表达亦增强，提示 CD147 的表达可能与肿瘤的转移相关。

CD147 与 MMP-2 常同时表达，可能表明 CD147 能促进 MMP-2 的产生。Sun 等[9]的研究也证实，提纯的 CD147 能诱导纤维母细胞和乳腺癌细胞株 MDA-435 分泌 MMP-2，而抗 CD147 抗体能抑制 MMP-2 的产生以及依赖于 MMP-2 的恶性细胞侵袭力。但本文 MMP-2 表达阳性率高于 CD147 可能反映 MMP-2 的产生不仅与 CD147 有关，尚与其他因素有关。有学者认为，MMP-2 的表达和活性与表皮生长因子（EGF）、转化生长因子（TGF-β）等多种活性递质有关[10]，这需要进一步研究。

［参考文献］

［1］ Westerlund A, Hujanen E, Puistola U, *et al*. Fibroblasts stimulate human ovarian cancer cell invasion and expression of 72-kDa gelatinase A (MMP-2)[J].Gynecol Oncol,1997,67:76–82.

［2］ Woessner JF. Matrix metalloproteinases and their inhibitors in connective tissue remodeling [J]. FASEB J, 1991,5(8):2145–2154.

［3］ Ota K, Stetler-Stevenson WG, Yang Q, *et al*. Cloning of murine membrane-type-1-matrix metalloproteinase (MT-1-MMP)and its inhibitor[J]. Kidney International, 1998,54: 131–142.

［4］ Goetzl EJ, Banda MJ, Leppert D. Matrix metalloproteinases in immunity[J]. J Immunol, 1996,156(1):1–4.

［5］ 李红梅，方伟岗，郑 杰，等. 不同转移潜能的人肿瘤细胞系金属蛋白酶活性分析 [J]. 中华病理学杂志, 1998,27(5):341–343.

［6］ Charoenrat P, Modjtahedi H, Rhys P, *et al*. Epidermal growth factor-like ligands differentially up-regulate matrix metalloproteinase 9 in head and neck squamous carcinoma cells[J]. Cancer Res, 2000, 60(4):1121–1128.

［7］ Kusukawa J, Sasaguri Y, Shima I, *et al*. Expression or matrix metalloproteinase-2 related to lymph node metastasis of oral squamous carcinoma cells. A clinicopathologic study[J].Am J Clin pathol,1993,99(1):18–23.

［8］ Guo H, Zucker S, Gordon MK, *et al*. Stimulation of matrix metalloproteinase production by recombinan extracelluar matrix metalloproteinase inducer from transfected Chinese hamster ovary cells[J]. J Biol Chem, 1997,272(1):24–27.

［9］ Sun J, Hemler ME. Regulation of MMP-1 and MMP-2 production through CD147/extracellular matrix metalloproteinase inducer interactions [J]. Cancer Res,2001,61 (5):2276–2281.

［10］ Curran S, Murray GI. Matrix metalloproteinase in tumour invasion and metastasis[J]. J Pathol,1999,189(3):300–308.

［收稿日期］ 2004–03–09

# 来稿图表制作要求

1.每幅图单占 1 页，集中附于文后；表则随文不单列。分别按其在正文出现的先后次序连续编码。每幅图表应冠有图(表)题。说明性的资料应置于图(表)下方的注释中。

2.本刊采用三横线表(顶线、表头线、底线)，如遇有合计或统计学处理行(如 $t$ 值、$P$ 值等)，则在这行上面加一条分界横线；表内数据要求同一指标有效位数一致，一般按标准差的 1/3 确定有效位数。

3.线条图应墨绘在白纸上，高度比例约为 5:7 左右。以计算机制图者应提供激光打印图样。照片图要求有良好的清晰度和对比度。图中须标注的符号(包括箭头)请用另张纸标上，每幅图的背面应贴上标签，注明图号及图的上下方向。大体标本照片在图内应有尺度标记。病理照片要求注明染色方法和放大倍数。

(C)1994-2022 China Academic Journal Electronic Publishing House. All rights reserved. http://www.cnki.net

## 尼莫地平对沙土鼠脑缺血后细胞凋亡及 Bcl-2 的影响

(正文见第 602 页)

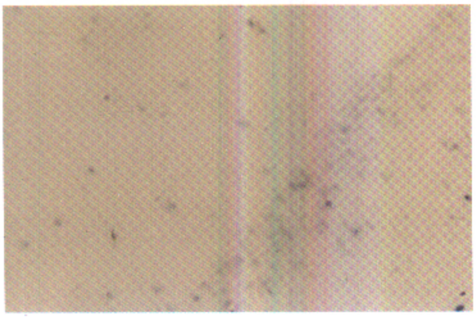

图 1　假手术组(72 h)海马 CA1 区神经元 TUNEL 染色特征(SP 法，×330)

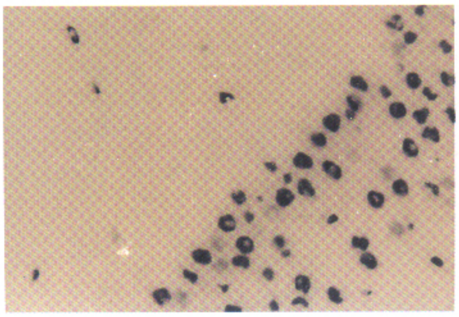

图 2　对照组(72 h)海马 CA1 区神经元 TUNEL 染色特征(SP 法，×660)

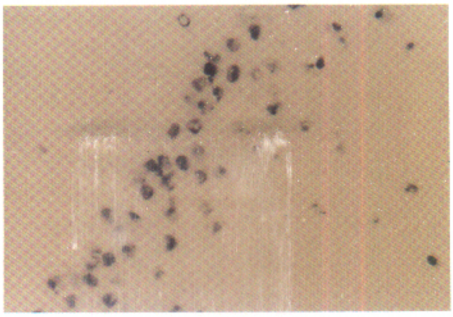

图 3　尼莫地平组(72 h)海马 CA1 区神经元 TUNEL 染色特征(SP 法，×660)

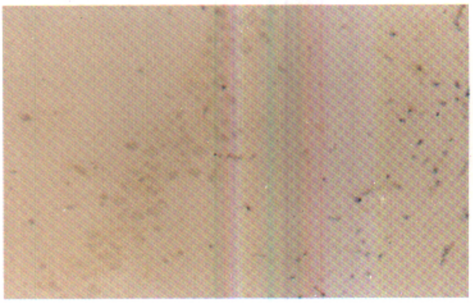

图 4　假手术组(72 h)海马 CA1 区神经元 Bcl-2 的表达(SP 法，×330)

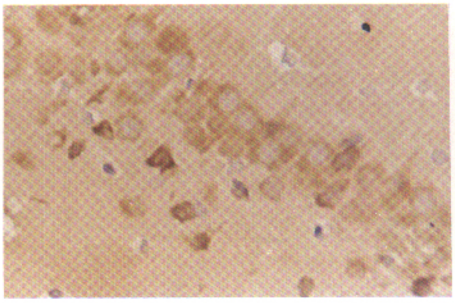

图 5　对照组(72 h)海马 CA1 区神经元 Bcl-2 的表达(SP 法，×660)

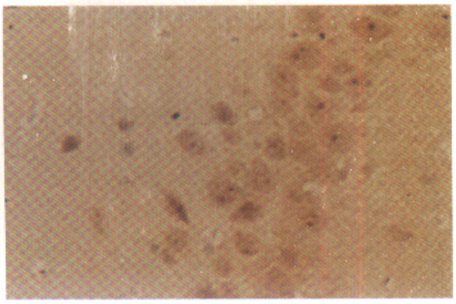

图 6　尼莫地平组(72 h)海马 CA1 区神经元 Bcl-2 的表达(SP 法，×660)

## 基质金属蛋白酶 2 及 CD147 在食管鳞状细胞癌中的表达及意义

(正文见第 621 页)

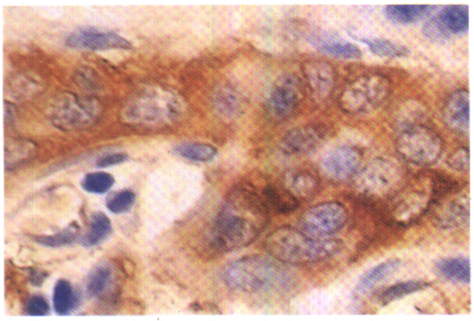

细胞质内见棕色颗粒。

图 1　食管鳞癌细胞 MMP-2 阳性表达(免疫组化 SP 法，DAB 显色，×400)

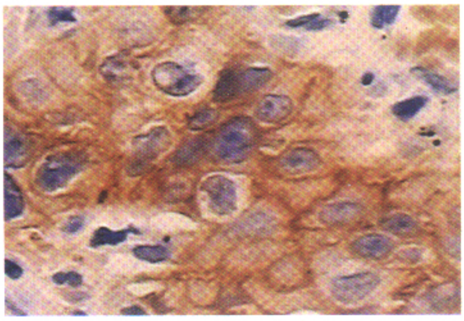

细胞膜着色为主，少量胞质着色。

图 2　食管鳞癌细胞 CD147 阳性表达(免疫组化 SP 法，DAB 显色，×400)

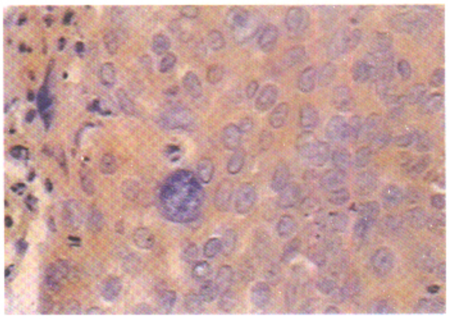

细胞质着色，与图 4 为同一部位。

图 3　食管鳞癌细胞 MMP-2 阳性表达(免疫组化 SP 法，DAB 显色，×200)

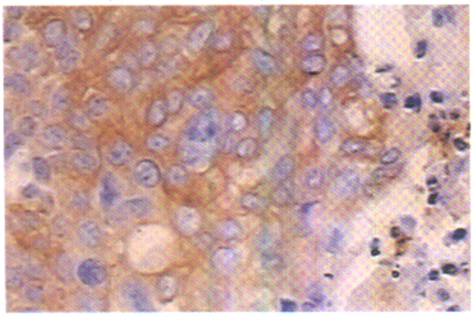

细胞膜和细胞质均着色。

图 4　食管鳞癌细胞 CD147 阳性表达(免疫组化 SP 法，DAB 显色，×200)

## 人脐血 CD56$^{+}$细胞毒性淋巴细胞的扩增研究

(正文见第 612 页)

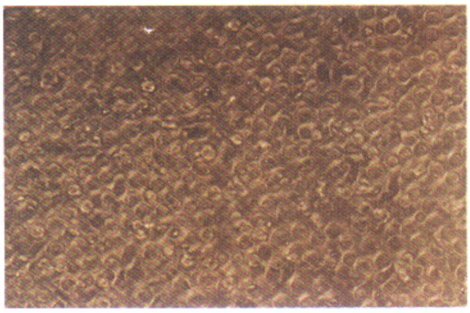

图　培养 12 天 CD56$^{+}$CIK 细胞形态(倒置显微镜×400)

(C)1994-2022 China Academic Journal Electronic Publishing House. All rights reserved. http://www.cnki.net
