# Supplementary material for: Expression of Concern: The prognostic and clinicopathologic characteristics of CD147 and esophagus cancer: A meta-analysis
Source: PLoS One. 2023 Feb 22;18(2):e0282229. doi: 10.1371/journal.pone.0282229 (PMC9946197; doi:10.1371/journal.pone.0282229)
Supplement: S1 File — (ZIP) [file pone.0282229.s001.zip › PDF of included paper/╓∞╔┘╛2-2011.pdf]

DOI：10.13423/j.cnki.cjcmi.006082

·论著·

文章编号：1007－8738（2011）07－0784－03

HAb18G/CD147在食管鳞状细胞癌中的表达及其临床意义

朱少君[1]，米　力[2]，李艳红[3]，张　阳[2]，巩　丽[1]，韩秀娟[1]，姚　丽[1]，兰　淼[1]，张　伟[1*]
（第四军医大学：[1]唐都医院病理科，[2]细胞工程研究中心，[3]唐都医院妇产科，陕西 西安 710038）

[摘 要] 目的：探讨HAb18G基因产物表达与食管鳞状细胞癌（ESCC）临床病理特征及预后的关系。方法：应用免疫组化SP染色法分析了108例食管鳞状细胞癌标本中HAb18G基因产物表达情况和定位，并对其进行了术后3年随访。结果：HAb18G基因产物在有淋巴结癌转移和预后差的食管鳞状细胞癌病例中高表达；HAb18G的高表达与食管鳞状细胞癌的浸润转移、分化程度均相关（$P<0.05$）；淋巴结转移和HAb18G表达可作为食管鳞状细胞癌患者预后独立指标。结论：HAb18G在食管鳞状细胞癌中的表达与食管癌的发生发展、浸润转移和预后密切相关，可作为临床预测浸润转移和估计预后的一个重要参考指标。

[关键词] HAb18G/CD147；食管鳞状细胞癌；免疫组化；预后
[中图分类号] R735.1　　[文献标识码] A

HAb18G/CD147分子，又名EMMPRIN（细胞外基质金属蛋白酶诱导因子），最初源于人肺癌细胞系LX21[1]。大量研究表明，HAb18G/CD147与其效应细胞（癌周成纤维细胞等）结合，可诱导产生基质金属蛋白酶（matrix metalloproteinase，MMP），使肿瘤组织中MMP的含量及活性显著提高，间质成分和血管基底膜的胶原蛋白过度降解，癌细胞便可穿过基底膜及结缔组织屏障不断侵袭扩散，与肿瘤的发生、转移和预后关系密切[2]。有研究报道HAb18G/CD147在食管鳞状细胞癌癌细胞胞膜和细胞浆中高表达并与肿瘤分化差密切相关，但HAb18G/CD147表达与临床病理特征和患者预后关系仍不清楚[3]。

食管鳞状细胞癌是我国常见的消化道恶性肿瘤之一，在世界范围内，其发病率一直呈上升趋势[4]。浸润和转移使得其发病率和死亡率比较高，严重影响了我国国民的生命健康。食管鳞状细胞癌发生和浸润、转移是非常复杂的过程，受多种相关基因的调控。我们应用免疫组化方法对108例食管鳞状细胞癌进行研究，探讨HAb18G的表达与食管鳞状细胞癌临床分期、浸润转移和预后的关系。

1 材料和方法

1.1 材料 鼠抗人HAb18G单克隆抗体由第四军医大学细胞工程研究中心制备，SP免疫组化试剂盒购自福州迈新公司。标本108例，福尔马林固定，石蜡包埋食管鳞状细胞癌标本来自2003－07/2005－09第四军医大学唐都医院病理科。所有患者手术前均未进行放疗、化疗或其他相关治疗。包括73例男性和35例女性，其中21例浸润至浅肌层、24例浸润至深肌层、63例浸润至全层。每例均经过两位病理科大夫分别观察，按多数认可的形态学标准[5]诊断证实。患者的年龄、性别、吸烟状态、家族史、病理类型及淋巴结转移等情况均来自住院病历；患者年龄为36～78岁，上述标本均排除存在其他恶性肿瘤病史。手术后，所有患者均进行了3～5年随访，所有死于意外事故或其他疾病的患者均剔除本研究。

1.2 方法 采用免疫组化SP法检测癌组织及癌转移淋巴结组织中HAb18G蛋白表达情况。标本用40 g/L甲醛液固定，石蜡包埋，切成3 μm厚，切片经63℃烤箱烘烤过夜后分别经二甲苯脱蜡，梯度酒精水化，以后免疫组化染色操作步骤严格按SP试剂盒说明书进行。

1.3 结果判断 HAb18G阳性标准：阳性显色为棕黄色，以胞膜着色为主，出现少量胞质着色。结果参照文献[6]评分方法，选5个高倍视野，计算阳性细胞数，以阳性细胞数<5%记为0分；35%≥阳性数>5%为1分；70%≥阳性数>35%为2分；阳性数>70%为3分。

1.4 统计学分析 采用SPSS 13.0软件，根据数据类型分别使用方差分析、χ^2检验，$P<0.05$为有统计学意义。HAb18G表达与预后分析用秩和检验，肿瘤分化、浸润深度、HAb18G表达等多变量与预后关系用COX回归分析。

2 结果

2.1 HAb18G在食管癌组织中的表达和定位 应用免疫组化方法共对108例食管鳞状细胞癌标本进行了染色（表1），并由2位高年资病理学专家根据免疫组化结果判定标准进行诊断（图1）。大部分病例在细胞膜着色（87.0%，94例/108例），少数病例只在细胞质着色（13.0%，14例/108例）。在所有病例中，29例HAb18G表达阴性（26.9%），44例低表达（40.7%），33例中度表达（30.5%），2例高表达（1.9%）。

2.2 HAb18G在淋巴结转移灶中的表达与原发灶一致 对27例有癌转移的食管旁淋巴结转移灶进行了染色，并与原发灶染色结果进行了对比，结果显示HAb18G在食管鳞状细胞癌淋巴结转移灶表达与原发灶表达结果一致（$P>0.05$，表2）。

收稿日期：2010－10－28；接受日期：2010－11－17
基金项目：国家高新技术研究发展计划（973）重点项目（2009CB521704）；国家自然科学基金资助项目（81001088，30672013，30800417）
作者简介：朱少君（1977－），男，山东泰安人，主治医师，硕士
Tel：029-84777744，E-mail：zhusjk@fmmu.edu.cn
* Corresponding author

?1994-2016 China Academic Journal Electronic Publishing House. All rights reserved. http://www.cnki.net

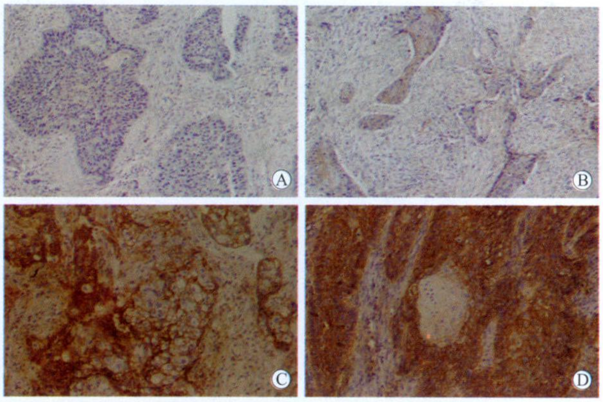

图 1 食管鳞状细胞癌免疫组化染色评分

A：0分（阳性细胞数＜5%）（×100）；B：1分（35%≥阳性细胞数＞5%）（×100）；B：2分（70%≥阳性细胞数＞35%）（×100）；D：3分（阳性细胞数＞70%）（×100）.

表 1 108例食管鳞状细胞癌 HAb18G表达强度与临床病理特征之间的关系 [n(%)]

	n	免疫组化强度评分			
		0	1	2	3
性别					
男	73	24(32.9)	25(34.2)	22(30.1)	2(2.8)
女	35	5(14.3)	19(54.3)	11(31.4)	0(0)
年龄					
＞60	49	11(22.5)	20(40.8)	17(34.7)	1(2.0)
≤60	59	18(30.5)	24(40.7)	16(27.1)	1(1.7)
分级					
高分化	24	8(33.3)	10(41.7)	6(25)	0(0)
中分化	54	14(25.9)	25(46.3)	15(27.8)	0(0)
低分化	30	7(23.3)	9(30.0)	12(40.0)	2(6.7)
浸润深度					
黏膜层	21	10(47.7)	7(33.3)	4(19.0)	0(0)
肌层	24	6(25.0)	13(54.2)	5(20.8)	0(0)
浆膜层	63	13(20.6)	24(38.1)	24(38.1)	2(3.2)
l转移情况					
无转移	64	21(32.8)	31(48.4)	12(18.8)	0(0)
有转移	44	8(18.2)	13(29.5)	21(47.7)	2(4.6)
随访结果					
生存	43	16(37.2)	19(44.2)	8(18.6)	0(0)
死亡	65	13(20.0)	25(38.5)	25(38.5)	2(3.0)

表 2 HAb18G在 27例淋巴结转移灶表达与肿瘤原发灶表达的关系 [n=27，n(%)]

HAb18G表达	免疫组化强度评分			
	0	1	2	3
原发灶组织	6(22.2)	6(22.2)	9(33.3)	6(22.2)
转移灶组织	8(29.6)	10(37.0)	5(18.5)	4(14.8)

2.3 食管鳞状细胞癌患者临床病理特征与 HAb18G表达的关系 表 1显示了 HAb18G表达与食管鳞状细胞癌患者临床病理特征的关系。免疫组化结果统计学分析显示在淋巴结转移病例（$P<0.01$）和预后差（$P<0.01$）的病例中 HAb18G高表达；HAb18G表达与食管鳞状细胞癌的分化也相关（$P<0.01$）。从表 1中我们还发现食管鳞状细胞癌分化程度与 HAb18G的表达线性相关（$P<0.01$），分化差的病例 HAb18G表达强；同时，肿瘤的浸润深度与 HAb18G的表达也呈线性相关（$P<0.05$），浸润深的病例 HAb18G表达强。食管鳞状细胞癌患者的性别和年龄与 HAb18G表达无关（$P>0.05$）。

2.4 食管鳞状细胞癌患者预后与 HAb18G表达的关系 食管鳞状细胞癌 3年生存率为 39.8%（43/108），平均生存时间为 53个月。对 108例食管癌患者进行 Cox回归分析显示，淋巴结转移和 HAb18G表达是食管鳞状细胞癌独立的预后指标（$P<0.05$，表 3）。

表 3 HAb18G表达和淋巴结转移是食管鳞状细胞癌独立的预后指标

变量	自由度	参数估计	标准差	χ^2	危险比
HAb18G表达	1	0.394	0.171	5.309	1.482
淋巴结转移	1	0.966	0.319	9.198	2.629

3 讨论

食管鳞状细胞癌是致死率相当高的恶性肿瘤，在由各类癌症引起的死亡中位居第 6位，在世界范围内其发病率一直呈上升趋势[4]。食管鳞状细胞癌的发生与环境及遗传等因素有关，暴露于环境致癌因素、不良饮食习惯、腌制食品以及微量元素等营养缺乏可能是人群患 ESCC的危险因素。食管鳞状细胞癌初诊时患者多为中晚期，手术切除病灶后患者的 3年生存率较低。侵袭转移是造成食管鳞状细胞癌患者死亡的主要原因，而转移又受多基因、多步骤的调控。

免疫组化检测 HAb18G/CD147主要在外周浸润的肿瘤细胞中表达，这与文献报道的 HAb18G/CD147在肿瘤与间质作用中起重要作用一致[7]。HAb18G/CD147在膀胱癌、皮肤癌、肺癌、乳腺癌以及淋巴瘤等许多恶性肿瘤中高表达，表明 HAb18G/CD147表达可能与肿瘤发生和发展有关，而且有文献报道 HAb18G/CD147阳性患者生存期短于阴性患者[9]。尽管肿瘤发生的原因和机制十分复杂，尚未完全清楚，但是 HAb18G/CD147在肝癌、宫颈癌等许多恶性肿瘤中起到很重要的作用。

本研究中，我们分析了食管鳞状细胞癌中 HAb18G的表达和定位情况。同时分析了 HAb18G的表达与食管鳞状细胞癌患者的性别、年龄、淋巴结转移、肿瘤浸润深度以及患者生存期的关系。结果显示 HAb18G在食管鳞状细胞癌中高表达，统计学分析显示在食管鳞状细胞癌中，HAb18G不仅与鳞状细胞癌淋巴结转移有关，还与肿瘤细胞浸润深度有关，有淋巴结转移和浸润较深的病例，HAb18G阳性率高。在低分化的食管鳞状细胞癌中，HAb18G表达阳性率较高，这与文献报道一致。同时发现，不同 HAb18G表达水平食管癌患者预后明显不同，

?1994-2016 China Academic Journal Electronic Publishing House. All rights reserved. http://www.cnki.net

HAb18G低表达的患者预后好，HAb18G高表达的患者预后明显差。HAb18G在淋巴结转移灶的表达与原发灶一致。同时我们也发现在一些原发灶阳性表达病例其转移灶是阴性表达的，这些病例的预后似乎很好（因为病例有限，我们无法对其进行统计学分析）。我们还发现淋巴结转移和HAb18G表达可独立作为食管鳞状细胞癌预后指标。以前的研究[9]认为HAb18G/CD147可以促进MMP产物从邻近基质细胞到远处细胞从而促进肿瘤细胞的转移。还有报道认为HAb18G/CD147可以在肿瘤细胞表面与MMP-1结合，在肿瘤细胞表面可以浓集胶原降解酶，从而促进肿瘤浸润。本研究结果显示HAb18G/CD147可能通过诱导或调节不同的功能蛋白的表达最后影响食管鳞状细胞癌的预后，其中的机制需要进一步深入的研究，但是淋巴结转移和HAb18G/CD147表达将是食管鳞状细胞癌发展和预后的独立预测指标。

总之，HAb18G高表达可能会提示肿瘤易于浸润和淋巴结转移，从而使患者预后较差。HAb18G在食管鳞状细胞癌中的表达为进一步研究食管鳞状细胞癌发生机制提供新的思路，并将促使我们进一步研究其在食管鳞状细胞癌发展中的关系和作用。同时，HAb18G作为MMP的诱导剂，将为食管鳞状细胞癌的靶向药物治疗提供一个新策略。

参考文献：

[1] Nabeshima K, Lane W S, Biswas C. Partial sequencing and characterization of the tumor cell-derived collagenase stimulatory factor[J]. Arch Biochem Biophys, 1991, 285(1): 90－96

[2] Li Z, Ren Y, Wu QC, et al. Macrophage migration inhibitory factor enhances neoplastic cell invasion by inducing the expression of matrix metalloproteinase 9 and interleukin-8 in nasopharyngeal carcinoma cell lines[J]. J Chin Med, 2004, 117: 107－114

[3] Cheng MF, Tzao C, Tsai WC, et al. Expression of EMMPRIN and matriptase in esophageal squamous cell carcinoma: correlation with clinicopathological parameters[J]. Dis Esophagus, 2006, 19: 482－486

[4] 朱少君，张 力，张 伟，等. 上皮性钙黏蛋白G-347 GA SNP单核苷酸多态性与食管癌的关系[J]. 细胞与分子免疫学杂志，2009，25(1): 1040－1041.

[5] 吴秀金. 食管疾病[A]. 见：邓仲端，熊敏，主编. 外科病理学[M]. 2版. 武汉：湖北科学技术出版社，1999: 84－94

[6] Iwasa S, Okada K, Chen WT, et al. Increased expression of seprase, a membrane-type serine protease, is associated with lymph node metastasis in human colorectal cancer[J]. Cancer Lett, 2003, 199(1): 91－98

[7] Caudroy S, Polette M, Tournier JM, et al. Expression of the extracellular matrix metalloproteinase inducer (EMMPRIN) and the matrix metalloproteinase-2 in bronchopulmonary and breast lesions[J]. J Histochem Cytochem, 1999, 47: 1575－1580.

[8] Gabison EE, Huet E, Baudouin C, et al. Direct epithelial-stromal interaction in corneal wound healing: Role of EMMPRIN/HAb18G/CD147 in MMPs induction and beyond[J]. Prog Retin Eye Res, 2009, 28(1): 19－33.

[9] Tang Y, Kesavan P, Nakada MT, et al. Tumor-stroma interaction: positive feedback regulation of extracellular matrix metalloproteinase inducer (EMMPRIN) expression and matrix metalloproteinase-dependent generation of soluble EMMPRIN[J]. Mol Cancer Res, 2004, 16(2): 73－80.

?1994-2016 China Academic Journal Electronic Publishing House. All rights reserved. http://www.cnki.net
