# Supplementary material for: Expression of Concern: The prognostic and clinicopathologic characteristics of CD147 and esophagus cancer: A meta-analysis
Source: PLoS One. 2023 Feb 22;18(2):e0282229. doi: 10.1371/journal.pone.0282229 (PMC9946197; doi:10.1371/journal.pone.0282229)
Supplement: S1 File — (ZIP) [file pone.0282229.s001.zip › PDF of included paper/╦└═÷╧α╣╪╡░░╫╝ñ├╕1║═CD147╘┌╩│╣▄┴█░⌐╓╨╡─▒φ┤∩╝░╞Σ┴┘┤▓╥Γ╥σ.pdf]

·临床研究·

死亡相关蛋白激酶 1 和 CD147 在食管鳞癌中的表达及其临床意义

万勇　吴心愿

【摘要】 **目的**　探讨死亡相关蛋白激酶 1(DAPK1)、CD147 在食管鳞癌中的表达状态及其与食管鳞癌预后的关系。**方法**　应用免疫组织化学方法和逆转录聚合酶链反应检测 DAPK1、CD147 在食管鳞癌和正常食管上皮组织中的表达水平,结合临床病理特征进行统计学分析。**结果**　DAPK1 蛋白在食管鳞癌组织及癌旁正常组织中的阳性表达率分别为 31.3% 和 58.5 %,CD147 蛋白的表达分别为 57.5 % 和 25.0%,差异均有统计学意义(均 $P<0.01$)。不同浸润深度、淋巴结转移状态、TNM 分期、分化程度的食管鳞癌患者 DAPK1 蛋白表达情况不同,差异有统计学意义(均 $P<0.01$);不同浸润深度、淋巴结转移、TNM 分期、分化程度的食管鳞癌患者 CD147 蛋白表达情况不同,差异有统计学意义(均 $P<0.01$)。DAPK1 mRNA 在 52 例食管鳞癌组织及癌旁正常组织中的表达水平分别为 0.236 ± 0.049 和 0.395 ± 0.058,CD147 mRNA 的表达水平分别为 0.942 ± 0.204 和 0.821 ± 0.171,差异均有统计学意义(均 $P<0.01$)。在无淋巴结转移、分化程度高、病理分期低的食管鳞癌组织中 DAPK1 mRNA 表达水平高;在淋巴结转移、分化程度低、病理分期高的食管鳞癌组织中 CD147 mRNA 表达水平高。**结论**　DAPK1、CD147 蛋白的表达与食管鳞癌的临床病理学特征有关,DAPK1、CD147 可能参与了食管鳞癌的转移过程,DAPK1、CD147 作为预测食管鳞癌预后的重要指标。

【主题词】 食管肿瘤;　肿瘤,鳞状细胞;　死亡相关蛋白激酶 1;　CD147;　预后

Expression and clinical significance of DAPK1 and CD47 in esophageal squamous cell carcinoma

WAN Yong, WU Xin-yuan. Department of Gastrosurgery, the Affiliated Union Hospital, Fujian Medical University, Fuzhou 350001, China

Corresponding author: WU Xin-yuan, Email: wuxinyuan1950@163.com

【**Abstract**】 **Objective**　To explore the amplification and expression status of DAPK1 and CD147 in esophageal squamous cell carcinoma (ESCC) and their relationship with the prognosis of ESCC. **Methods** Immunohistochemical staining and RT-PCR were used to detect the expression and amplification of DAPK1 and CD147 in esophageal squamous carcinoma tissue and normal esophageal mucosa. Statistical analysis of the clinocopathological data was performed with SPSS 11.5 software package. **Results**　The positive rates of expression of DAPK1 protein and CD147 protein in the specimens of esophageal carcinoma were 31.3% and 58.5%, and in normal esophageal mucosa 57.5% and 25.0%, respectively, with a statistically significant difference ($P<0.001$). The expressions of DAPK1 and CD147 were significant correlated with invasion depth, lymph node metastasis, TNM stage and the degree of cancer differentiation. ($P<0.05$). The negative expression of DAPK1 and positive expression of CD147 indicated a poor prognosis. In 52 ESCC cases, the expression of DAPK1 in cancer tissues was 0.236 ± 0.049, and 0.395 ± 0.058 in normal esophageal mucosa, while that of CD147 mRNA expression was 0.942 ± 0.204 and 0.821 ± 171, respectively, statistically both with a very significant difference ($P<0.01$). There was a higher expression level of DAPK1 mRNA in the cancer tissue in patients with no lymph node metastasis, well differentiation, and earlier pathological stage, and a higher expression level of CD147 mRNA in the cancer tissues in patients with lymph node metastasis, poor differentiation, and later pathological stage. **Conclusions**　The expression of DAPK1 and CD147 proteins is closely correlated with the clinicopathological characteristics of ESCC. The genes DAPK1 and CD147 may participate in the metastasis and apoptosis of ESCC. The expression of DAPK1 and CD147 may be used as important prognostic predictors in ESCC.

【**Subject words**】 Esophageal neoplasms;　Neoplasms, squamous cell;　Death associated protein kinase 1;　CD147;　Prognosis

---

DOI:10.3760/cma.j.issn.0253-3766.2012.01.010

作者单位:350001 福州,福建医科大学附属协和医院胃外科[万勇(现工作单位:350004 福州,福建省人民医院肿瘤外科)、吴心愿]

通信作者:吴心愿, Email: wuxinyuan1950@163.com

食管癌因其预后较差、易发生浸润转移的特点，一直是肿瘤治疗学中亟待解决的重点和难点问题之一。明确了食管鳞癌的增殖、抗凋亡及侵袭转移机制，可为临床提高食管癌的治疗效果、改善患者预后打开一扇希望之门。死亡相关蛋白激酶1（death associated protein kinase 1，DAPK1）是一种与肿瘤凋亡、转移等密切相关的丝氨酸/苏氨酸激酶，可激活经典的p53-半胱氨酸蛋白酶（caspase）途径以及不依赖于caspase凋亡途径的多条肿瘤细胞凋亡通路，同时DAPK1可被Fas、γ-干扰素（INF-γ）、肿瘤坏死因子β（TNF-β）、c-myc等因子激活，很可能是多种信号诱导肿瘤细胞凋亡的汇合点[1]。CD147是一种在多种组织的细胞膜表面表达的跨膜蛋白，通过诱导基质金属蛋白酶（matrix metalloproteinases，MMPs）产生，强化胶原蛋白酶水解作用，同时与整合素α3β1和α6β1形成复合体，促进基底膜的降解和肿瘤细胞的移出，在肿瘤的转移过程中具有重要作用。我们探讨DAPK1、CD147表达情况与食管鳞癌转移的关系，为食管鳞癌的临床病理诊断及其预后提供参考。

资料与方法

1. 标本：收集2003年12月至2006年6月我院病理科，行手术切除且具有完整随访资料的食管鳞癌存档石蜡标本80例。80例患者中，男57例，女23例，年龄37～78岁，中位年龄58.8岁。高分化鳞癌27例，中分化鳞癌24例，低分化鳞癌29例。临床分期依据2009年国际抗癌联盟（UICC）TNM分期标准，Ⅰ期10例，Ⅱ期32例，Ⅲ期30例，Ⅳ期8例。淋巴结转移41例，无淋巴结转移39例。所有标本均经10%中性甲醛固定，石蜡包埋，连续4 μm切片，分别进行HE染色和免疫组化染色。取食管鳞癌标本的相应癌旁正常上皮组织（距离癌灶>5 cm）80例，均经病理确诊，作为对照组。另外选取2009年10月至2010年3月，我院新发食管鳞癌患者食管鳞癌及癌旁食管正常上皮组织新鲜手术标本52例。所有标本均在手术切除后立即装入冻存管内，转移到－80℃液氮罐内过夜，以备提取总RNA。所有入选病例术前均未经过化疗或放疗等抗肿瘤治疗。

2. 主要试剂：兔抗人DAPK1多克隆抗体（工作浓度1:150）、鼠抗人CD147单克隆抗体（工作浓度1:50）均购自美国Santa Cruz公司，免疫组化试剂盒购自北京中山金桥生物公司，总RNA提取试剂Trizol、逆转录试剂盒均购自美国Invitrogen公司，DEPC、Taq PCR MasterMIX、核酸染料Goldview、600 bp DNA marker均购自北京百泰克生物公司，氯仿、异丙醇均为国产分析纯。DAPK1、CD147、内参照β-actin引物序列，根据美国生物技术信息中心GenBank中人的cDNA序列，由上海生物工程有限公司合成（表1）。

表1 DAPK1、CD147和β-actin的引物序列

基因	引物序列	片段大小(bp)
DAPK1	上游:5′-TGACAGTTTATCATGACCCGTGTTCAG-3′	245
	下游:5′-GTGCTGGATCTCCTTCAGGAT-3′	
CD147	上游:5′-GAGAGCAGGTTCTTCGTGAGTTC-3′	318
	下游:5′-GCCTTTGTCATTCTGGTGCTG-3′	
β-actin	上游:5′-CTGGGACGACATGGAGAA AA-3′	564
	下游:5′-AAGGAAGGCTGGAAGAGTGC-3′	

注：DAPK1：死亡相关蛋白激酶1

3. 免疫组化检测及结果判定：免疫组化检测使用的标本是80例食管鳞癌存档石蜡标本。严格按照免疫组化试剂盒说明书进行操作，用磷酸盐缓冲液（0.01 mol/L，pH 7.4）代替一抗作为阴性对照。免疫组化结果判定：(1)DAPK1蛋白定位于细胞浆，以细胞浆出现棕色染色为阳性细胞。每张切片随机观察8个高倍视野（×400），计数癌细胞总数和阳性表达的细胞个数，计算阳性细胞表达率。阳性细胞表达率≥30%为阳性（+），<30%为阴性（－）。(2)CD147蛋白定位于细胞膜，见淡黄色颗粒，明显高于背景为1分，较多棕黄色颗粒为2分，大量深棕色颗粒为3分。每张切片随机观察5个高倍视野（×400），计数癌细胞总数和阳性表达的细胞个数。阳性细胞表达率≤5%为0分，6%～25%为1分，26%～50%为2分，51%～75%为3分，≥76%为4分。结果以阳性细胞着色程度评分和阳性细胞表达率评分两项乘积表示，0～4分为低表达，>5分为高表达[2]。

4. 半定量RT-PCR检测：半定量RT-PCR检测使用的标本是52例食管鳞癌及癌旁食管正常上皮组织新鲜手术标本。分别提取食管鳞癌和癌旁正常组织的总RNA，按试剂盒说明书逆转录合成cDNA，再进行PCR扩增。PCR反应条件：95℃预变性2 min，95℃变性30 s，退火（DAPK1 58℃、CD 147 55℃、β-actin 53℃）30 s，72℃延伸5 min，共30个循环。循环结束后，72℃延伸10 min。用TBE缓冲液配置琼脂糖凝胶10 g/L，取RT-PCR产物6 μl，与

10×凝胶上样缓冲液混合均匀后上样。在 TBE 缓冲液中做潜水电泳，稳压 85 V 电泳 45 min。电泳完毕后，使用 Gel-Pro 凝胶图像分析系统，分别测定电泳产物条带的绝对积分光密度值（integral optical density, IOD），结果以目的 mRNA 的 RT-PCR 产物的电泳条带 IOD 比值表示，进行半定量分析。IOD 比值 = 目的基因 IOD 值/内参基因 IOD 值×100%。

5. 随访：所有患者均进行术后随访，随访时间 1～72个月。其中失访 7 例，随访率为 91.3%。

6. 统计学方法：采用 SPSS 11.5 统计学软件包进行统计学处理。计量资料数据以均数 ± 标准差（$\bar{x} \pm s$）表示。对数据进行正态性检验和组间方差齐性检验，数据呈正态分布和方差齐，采用单因素方差分析（One-Way ANOVA），否则采用秩和检验。计数资料采用 χ^2 检验，等级资料采用秩和检验，DAPK1、CD147 mRNA 表达情况的相关性采用 Pearson 相关分析。生存分析采用 Kaplan-Meier 生存曲线分析，并用 Log rank 进行检验，采用 Cox 回归分析 DAPK1、CD147 蛋白表达对生存的影响。检验水准 $\alpha = 0.05$。

结　　果

1. DAPK1、CD147 蛋白在食管鳞癌组织及癌旁正常食管上皮组织中的表达：DAPK1 主要在细胞浆中表达，在正常组织中呈高表达，而在癌组织中低表达或不表达（图 1）；CD147 主要在细胞膜中表达，在正常组织中低表达或不表达，而在癌细胞中呈高表达（图 2）；DAPK1 蛋白在食管鳞癌组织和正常鳞状上皮的阳性表达率分别为 31.3% 和 58.5%，CD147 蛋白在食管磷癌组织和正常磷状上皮的阳性表达率分别为 57.5% 和 25.0%，差异均具有统计学意义（均 $P < 0.001$）。

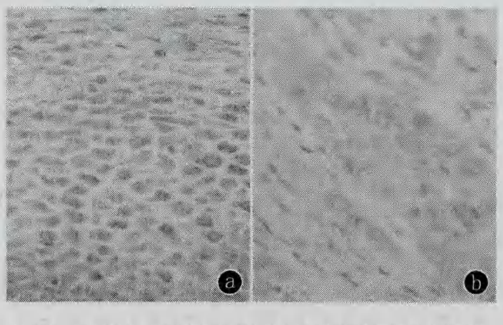

图 1　食管鳞癌组织和食管正常上皮组织中死亡相关蛋白激酶 1 蛋白的表达　免疫组化 SP 法　×400　a：食管正常上皮组织中死亡相关蛋白激酶 1 蛋白高表达；b：食管鳞癌组织中死亡相关蛋白激酶 1 蛋白低表达

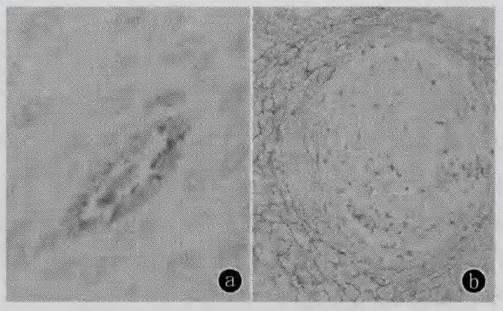

图 2　食管鳞癌组织和食管正常上皮组织中 CD147 蛋白的表达　免疫组化 SP 法　×400　a：食管正常上皮组织中 CD147 蛋白低表达；b：食管鳞癌组织中 CD147 蛋白高表达

2. DAPK1、CD147 蛋白表达与食管鳞癌临床病理特征的关系：80 例食管鳞癌中，DAPK1 蛋白的表达与患者的年龄、性别无关（均 $P > 0.05$），不同食管鳞癌浸润深度、淋巴结转移状态、TNM 分期、分化程度患者 DAPK1 蛋白表达情况不同，差异有统计学意义（Mann-Whitney U 值分别为 339.50、281.5、337.5 和 675.0，均 $P < 0.01$），不同食管鳞癌浸润深度、淋巴结转移状态、TNM 分期、分化程度患者 CD147 蛋白表达情况不同，差异有统计学意义（Mann-Whitney U 值分别为 518.0、372.0、467.0 和 934.0，均 $P < 0.01$，表 2）。

表 2　DAPK1、CD147 蛋白表达与食管鳞癌临床病理特征的关系（例）

临床病理特征	例数	DAPK1 表达 阴性	DAPK1 表达 阳性	P 值	CD147 表达 阴性	CD147 表达 阳性	P 值
年龄（岁）							
≥60	35	25	10	0.65	15	20	0.96
<60	45	30	15		19	26	
性别							
男	57	40	17	0.74	24	33	0.69
女	23	15	8		10	13	
浸润深度							
pT1	11	3	8		10	1	
pT2	13	5	8	<0.01	6	7	<0.01
pT3 和 pT4	56	47	9		18	38	
淋巴结转移							
有	39	17	22	<0.01	26	13	<0.01
无	41	38	3		8	33	
病理分期							
Ⅰ期	10	2	8		9	1	
Ⅱ期	32	18	14	<0.01	18	14	<0.01
Ⅲ期	30	28	2		5	25	
Ⅳ期	8	7	1		2	6	
分化程度							
低分化	31	29	2		7	24	
中分化	25	15	10	<0.01	11	14	0.01
高分化	24	11	13		16	8	

注：DAPK1：死亡相关蛋白激酶 1

3. DAPK1蛋白与CD147蛋白在食管鳞癌组织中表达的相关性分析：在食管鳞癌组织中DAPK1蛋白和CD147蛋白的表达水平呈负相关（$r=-0.348$，$P=0.002$）。

4. DAPK1、CD147 mRNA在食管鳞癌组织及癌旁正常食管上皮组织中的表达：半定量RT-PCR分析结果显示，DAPK1 mRNA在52例食管鳞癌组织及癌旁正常组织中的表达水平分别为0.236 ± 0.049和0.395 ± 0.058，差异有统计学意义（$P<0.01$，图3）。CD147 mRNA在52例食管鳞癌组织及癌旁正常组织中的表达水平分别为0.942 ± 0.204和0.821 ± 0.171，差异有统计学意义（$P<0.01$，图4）。

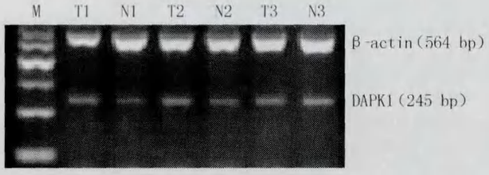

注：M：分子量标记；T：食管鳞癌组织；N：癌旁正常组织；DAPK1：死亡相关蛋白激酶1

图3　半定量RT-PCR法检测DAPK1在食管鳞癌和癌旁正常组织中的表达

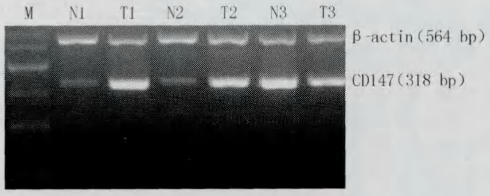

注：M：分子量标记；T：食管鳞癌组织；N：癌旁正常组织

图4　半定量RT-PCR法检测CD147 mRNA在食管鳞癌和癌旁正常组织中的表达

5. DAPK1、CD147 mRNA表达与食管鳞癌临床病理特征的关系：新鲜食管鳞癌组织中DAPK1 mRNA表达水平与淋巴结转移、分化程度和病理分期均有关（均$P<0.05$），CD147 mRNA表达水平也与淋巴结转移分化程度和病理分期有关（均$P<0.05$，表3）。

6. DAPK1、CD147蛋白表达与食管鳞癌患者预后的关系：Kaplan-Meier生存曲线分析显示，食管鳞癌患者的生存曲线在DAPK1和CD147的不同表达状态，其差异均有统计学意义（均$P<0.05$，图5、6）。Cox模型回归分析结果显示，CD147表达阳性和分化程度是影响食管鳞癌患者预后的危险因素（$RR=2.371$，$P=0.005$；$RR=0.567$，$P=0.002$）。

表3　DAPK1、CD147 mRNA表达与食管鳞癌临床病理特征的关系（$\bar{x} \pm s$）

临床病理特征	例数	DAPK1 mRNA	P值	CD147 mRNA	P值
淋巴结转移					
无	33	0.385 ± 0.065	<0.01	0.833 ± 0.172	<0.01
有	19	0.211 ± 0.041		1.055 ± 0.241	
分化程度					
低分化	11	0.121 ± 0.031		1.033 ± 0.232	
中分化	18	0.235 ± 0.045	<0.01	0.933 ± 0.191	<0.05
高分化	23	0.395 ± 0.039		0.879 ± 0.238	
病理分期					
Ⅰ期	12	0.383 ± 0.045		0.819 ± 0.167	
Ⅱ期	22	0.265 ± 0.035	<0.05	0.858 ± 0.272	<0.01
Ⅲ+Ⅳ期	18	0.133 ± 0.065		1.048 ± 0.132	

注：DAPK1：死亡相关蛋白激酶1

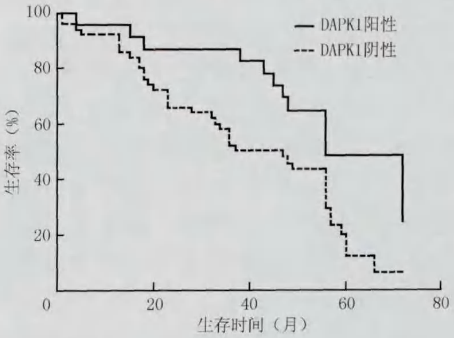

图5　死亡相关蛋白激酶1(DAPK1)表达阳性和阴性食管鳞癌患者的生存曲线

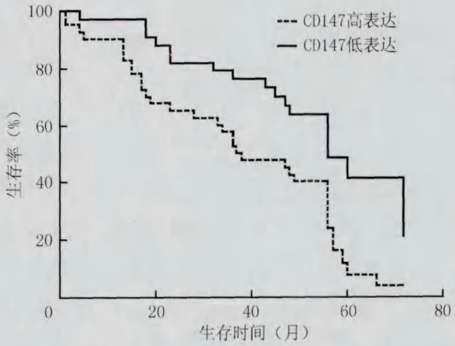

图6　CD147表达阳性和阴性食管鳞癌患者的生存曲线

讨　论

近年来，我国在食管癌的综合治疗及个体化治疗取得了一定的成绩，但由于食管壁内含有较为丰富的淋巴组织，相当一部分患者早期即可出现跳跃性或广泛淋巴结转移，患者疗效及预后仍不理想。

DAPK1作为凋亡的正调控因子，可通过$p19^{Arf}$/p53等途径抑制细胞黏附和细胞外黏附依赖的信号

分子参与肿瘤细胞凋亡、转移过程。有研究表明，p19Arf可作为 DAPK1 的酶作用底物，DAPK1 通过磷酸化 p19Arf 从而激活 p19Arf 依赖的 p53 凋亡途径[3-4]。DAPK 还可通过上调 p53-caspase 级联反应中的重要因子 Bax、Noxa、Apaf-1 等激活 p53-caspase 途径。此外，DAPK1 还参与了 Fas-FasL、由线粒体释放的凋亡刺激因子激活的内源性凋亡途径[5]。在肿瘤患者的分子流行病学和肿瘤细胞株的研究中均证实，肿瘤细胞 DAPK1 表达水平低或者不表达。临床病理资料的研究显示，DAPK 和肿瘤的发生、发展及预后有着密切的关系。Matsumoto 等[6]的研究显示，DAPK1 表达阴性的肝细胞癌患者比阳性患者血清甲胎蛋白(AFP)水平低、肿瘤分化程度高、组织的浸润和转移较少、肿瘤细胞凋亡指数高、无病生存率和总生存率高。另外，DAPK1 甲基化已经在消化道肿瘤[7]、B 细胞恶性淋巴瘤[8]、造血系统肿瘤[9]、头颈部肿瘤[10]、肺癌[11]等多种肿瘤中被证实。DAPK 启动子区域 CpG 岛的甲基化会导致 DAPK 表达的下调或者失活[12]。这使得一部分凋亡信号无法通过 DAPK 诱导细胞凋亡，从而增加部分细胞发生恶变的可能性。

CD147 是一种在多种细胞的胞膜表面表达的跨膜糖蛋白，通过诱导产生 MMPs，强化胶原蛋白酶水解作用，且可以与整合素 $\alpha3\beta1$ 和 $\alpha6\beta1$ 形成复合体，促进基底膜的降解和肿瘤细胞的移出。另外，CD147 的过表达可促进肿瘤血管内皮生长因子的大量产生，加速肿瘤血管的生成和生长。CD147 在正常组织中表达有限，甚至表达缺失，但在肿瘤组织中通常呈高表达。有研究显示，在神经胶质瘤、喉鳞癌、骨巨细胞瘤和黑色素瘤等恶性肿瘤中，CD147 均有高表达，而且其表达与肿瘤的浸润、转移关系密切[13]。研究表明，CD147 的表达强度与肿瘤的侵袭能力相关，并能反映肿瘤患者的预后，检测 CD147 表达水平可作为肿瘤侵袭、转移及预后的潜在标记物[14]。在基因靶向治疗方面，以 MMPs 的诱导剂 CD147 为靶标可能是一种有效的肿瘤治疗方式，因为通过抑制 CD147 就可以抑制 MMPs 的分泌和活性，进而遏制肿瘤的侵袭和转移。

本研究结果显示，不同的食管鳞癌浸润深度、淋巴结转移状态、TNM 分期、分化程度患者 DAPK1、CD147 蛋白的表达情况均不同(均 $P < 0.01$)。Kaplan-Meier 生存曲线显示，DAPK1 阳性表达、CD147 低表达的食管鳞癌患者预后较好。

总之，DAPK1、CD147 的表达与食管鳞癌的侵袭、转移及预后有着密切关系。随着研究深入，DAPK1、CD147 基因或将成为食管鳞癌早期发现及侵袭、转移、预后分析的遴选基因。研究 DAPK1、CD147 的分子作用机制及其参与肿瘤发生、发展的信号传导过程，探求阻断其信号传导或抑制其作用的方法，可能为未来肿瘤的治疗提供新的视野。

参 考 文 献

[1] Cohen O, Inbal B, Kissil JL, et al. DAP-kinase participates in TNF-alpha- and Fas-induced apoptosis and its function requires the death domain. J Cell Biol, 1999, 146:141-148.

[2] 许良中，杨文涛. 免疫组织化学反应结果的判断标准. 中国癌症杂志, 1996, 6:229-231.

[3] Bialik S, Bresnick AR, Kimchi A. DAP-kinase-mediated morphological changes are localization dependent and involve myosin-II phosphorylation. Cell Death Differ, 2004, 11:631-644.

[4] Kogel D, Reimertz C, Dussmann H, et al. The death associated protein (DAP) kinase homologue Dlk/ZIP kinase induces p19ARF-and p53-independent apoptosis. Eur J Cancer, 2003, 39:249-256.

[5] Cohen O, Inbal B, Kissil JL, et al. DAP-kinase participates in TNF-alpha-and Fas-induced apoptosis and its function requires the death domain. J Cell Biol, 1999, 146:141-148.

[6] Matsumoto H, Nagao M, Ogawa S, et al. Prognostic significance of death-associated protein-kinase expression in hepatocellular carcinomas. Anticancer Res, 2003, 23:1333-1341.

[7] Widschwendter A, Muller HM, Fiegl H, et al. DNA methylation in serum and tumors of cervical cancer patients. Clin Cancer Res, 2004, 10:565-571.

[8] Esteller M. Relevance of DNA methylation in the management of cancer. Lancet Oncol, 2003, 4:351-358.

[9] Reddy AN, Jiang WW, Kim M, et al. Death-associated protein kinase promoter hypermethylation in normal human lymphocytes. Cancer Res, 2003, 63:7694-7698.

[10] Takahashi T, Shivapurkar N, Reddy J, et al. DNA methylation profiles of lymphoid and hematopoietic malignancies. Clin Cancer Res, 2004, 10:2928-2935.

[11] Yamaguchi S, Asao T, Nakamura J, et al. High frequency of DAP-kinase gene promoter methylation in colorectal cancer specimens and its identification in serum. Cancer Lett, 2003, 194:99-105.

[12] Toyooka S, Toyooka KO, Miyajima K, et al. Epigenetic down-regulation of death-associated protein kinase in lung cancers. Clin Cancer Res, 2003, 9:3034-3041.

[13] Si AI, Huang L, Xu J, et al. Expression and localization of extracellular matrix metalloproteinase inducer in giant cell tumor of bone. J Cell Biochem, 2003, 89:1154-1163.

[14] Als AB, Dyrskjot L, von der Maase H, et al. Emmprin and survivin predict response and survival following cisplatin-containing chemotherapy in patients with advanced bladder cancer. Clin Cancer Res, 2007, 13:4407-4414.

(收稿日期:2010-09-21)
