# Supplementary material for: Expression of Concern: The prognostic and clinicopathologic characteristics of CD147 and esophagus cancer: A meta-analysis
Source: PLoS One. 2023 Feb 22;18(2):e0282229. doi: 10.1371/journal.pone.0282229 (PMC9946197; doi:10.1371/journal.pone.0282229)
Supplement: S1 File — (ZIP) [file pone.0282229.s001.zip › PDF of included paper/╧╦╬1⁄4┴1⁄4╜╙╡░░╫íó▓π≡ñ┴1⁄4╡░░╫íó╧╕░√═Γ╗∙╓╩╜≡╩⌠╡░░╫├╕╙╒╡╝╥≥╫╙╘┌╩│╣▄░⌐╡─▒φ┤∩.pdf]

维普资讯 http://www.cqvip.com

· 论著 ·

纤维连接蛋白、层黏连蛋白、细胞外基质金属蛋白酶诱导因子在食管癌的表达

谢玲 裴志东 马冬萍 薛琪 林勇虎 陈志军 任铁军

【摘要】 **目的** 研究纤维连接蛋白(FN)、层黏连蛋白(LN)、细胞外基质金属蛋白酶诱导因子(EMMPRIN)在食管癌的表达,探讨其临床意义。**方法** 采用免疫组织化学法对 87 例食管癌组织、30 例正常食管组织、20 例不典型增生食管组织进行 FN、LN、EMMPRIN 检测。**结果** 正常食管组织基底膜 FN、LN 染色呈连续线状;低分化癌巢周围基底膜 FN、LN 染色表现为碎片状;FN、LN 表达与食管癌病理分级、淋巴结转移有关;EMMPRIN 的弱阳性表达与食管癌淋巴结转移有关,强阳性表达与食管癌的病理分级、临床分期和淋巴结转移有关。**结论** FN、LN、EMMPRIN 表达可作为评价食管癌侵袭性及恶性程度的有用指标。

【关键词】 食管肿瘤; 纤连蛋白类; 层黏连蛋白; 金属蛋白酶类; 免疫组织化学

【中图分类号】 R735.1 【文献标识码】 A 【文章编号】 1673-422X(2008)02-0156-04

Expression of fibronectin, laminin and extracellular matrix metalloproteinase inducer in esophageal Carcinoma

XIE Ling, PEI Zhi-dong, MA Dong-ping, XUE Qi, LIN Yong-hu, CHEN Zhi-jun, REN Tie-jun. Department of Oncology, Luoyang Central Hospital, Luoyang 471000, China

【**Abstract**】 **Objective** To study the expression and clinical significance of fibronectin(FN), laminin (LN) and extracellular matrix metalloproteinase inducer(EMMPRIN) in esophageal carcinoma. **Methods** Immunohistochemical staining was used to detected the expression of FN, LN and EMMPRIN in 87 esophageal carcinoma tissues, 30 normal esophageal tissues and 20 dysplasia of esophageal tissues. **Results** In normal esophageal tissues, FN and LN in basement membranes were continuous linear; in poorly differentiated carcinomas, FN and LN in basement membranes were fragmentary. There were correlations between the expression of FN, LN and pathology grade, lymph node metastasis of esophageal carcinoma; there was correlation between the weakly positive expression of EMMPRIN and lymph node metastasis of esophageal carcinoma, there were correlations between the intensely positive expression of EMMPRIN and pathology grade, clinical stage and lymph node metastasis of esophageal carcinoma. **Conclusion** FN, LN and EMMPRIN can be used as helpful markers to evaluate the invasive ability and malignant degree of esophageal carcinoma.

【**Key words**】 Esophageal neoplasms; Fibronectins; Laminin; Metalloproteinases; Immunohistochemistry

我国是食管癌高发地区,食管癌治疗失败的主要原因是局部复发和远处转移,如能早期预测食管癌的复发和转移,可明显改善患者预后。我们采用免疫组织化学法检测纤维连接蛋白(fibronectin, FN)、层黏连蛋白(laminin, LN)、细胞外基质金属蛋白酶诱导因子(extracellar matrix metalloproteinase inducer, EMMPRIN)在食管癌中的表达,探讨与食管癌侵袭转移的关系及临床意义。

基金项目:洛阳市科技发展计划基金资助项目(051023)

作者单位:471000 河南省洛阳市中心医院肿瘤科

1 资料与方法

1.1 资料

收集本院 2003 年 4 月 1 日至 2006 年 6 月 30 日外科手术切除的食管癌病例标本 87 例,年龄在 37~76 岁之间,平均年龄 53.2 岁。病理类型均为食管鳞状细胞癌,其中Ⅰ级 25 例,Ⅱ级 50 例,Ⅲ级 12 例;Ⅱ期 19 例,Ⅲ期 68 例;淋巴结转移 35 例,无淋巴结转移 52 例;所有病例手术前均未经放疗或化疗。30 例正常食管组织标本、20 例不典型增生食管组织标本取自胃镜检查患者。所有标本均经 10% 福尔马林固定、石蜡包埋,连续切片 3 张,厚约 4~5 μm,2 张作

维普资讯 http://www.cqvip.com

免疫组织化学染色,1张作苏木素-伊红染色复查诊断。

1.2 方法

兔抗人FN多克隆抗体、鼠抗人LN单克隆抗体、超敏S-P试剂盒、联苯二胺显色剂购自福州迈新生物技术开发有限公司,兔抗人EMMPRIN多克隆抗体购自北京中杉金桥生物技术有限公司。用已知阳性切片作为阳性对照,用0.01 mol/L磷酸盐缓冲盐液代替一抗作为阴性对照,严格按S-P试剂盒免疫组化染色步骤进行。

1.3 结果判定

FN、LN阳性结果为基底膜、间质纤维组织、癌细胞的细胞质出现黄色或棕黄色物,基底膜染色结果根据染色范围及基底膜的完整程度分4级,<10%、碎片状为(-);10%~25%、碎片状为(+);26%~50%、断线状为(++);>50%、连续线状为(+++)。(-)~(+)为基底膜明显缺损,(++)~(+++)为基底膜非明显缺损。EMMPRIN以细胞膜和细胞质呈棕黄色为阳性,根据显色强度和范围分为:阴性(-):阳性染色细胞<5%或仅有与正常黏膜相同的基底层细胞染色;弱阳性(+):阳性细胞5%~50%或染色浅;强阳性(++):阳性细胞>50%或显色深。

1.4 统计学处理

用SPSS11.5统计软件对实验结果进行卡方检验、秩和检验,$P<0.05$有统计学意义。

2 结果

2.1 不同食管组织中FN、LN、EMMPRIN的表达

2.1.1 FN、LN的表达:正常食管组织基底膜FN、LN染色呈连续线状;不典型增生时基底膜FN、LN染色一般是连续的(图1a、1b),偶有断线状;高分化的食管癌癌巢周围可有完整的基底膜FN、LN染色,低分化的癌巢周围基底膜FN、LN染色为碎片状或完全缺如(图1c、1d),间质纤维组织中可见FN阳性染色,无LN阳性染色,部分癌细胞的细胞质中可见FN、LN阳性表达(图1e、1f)。

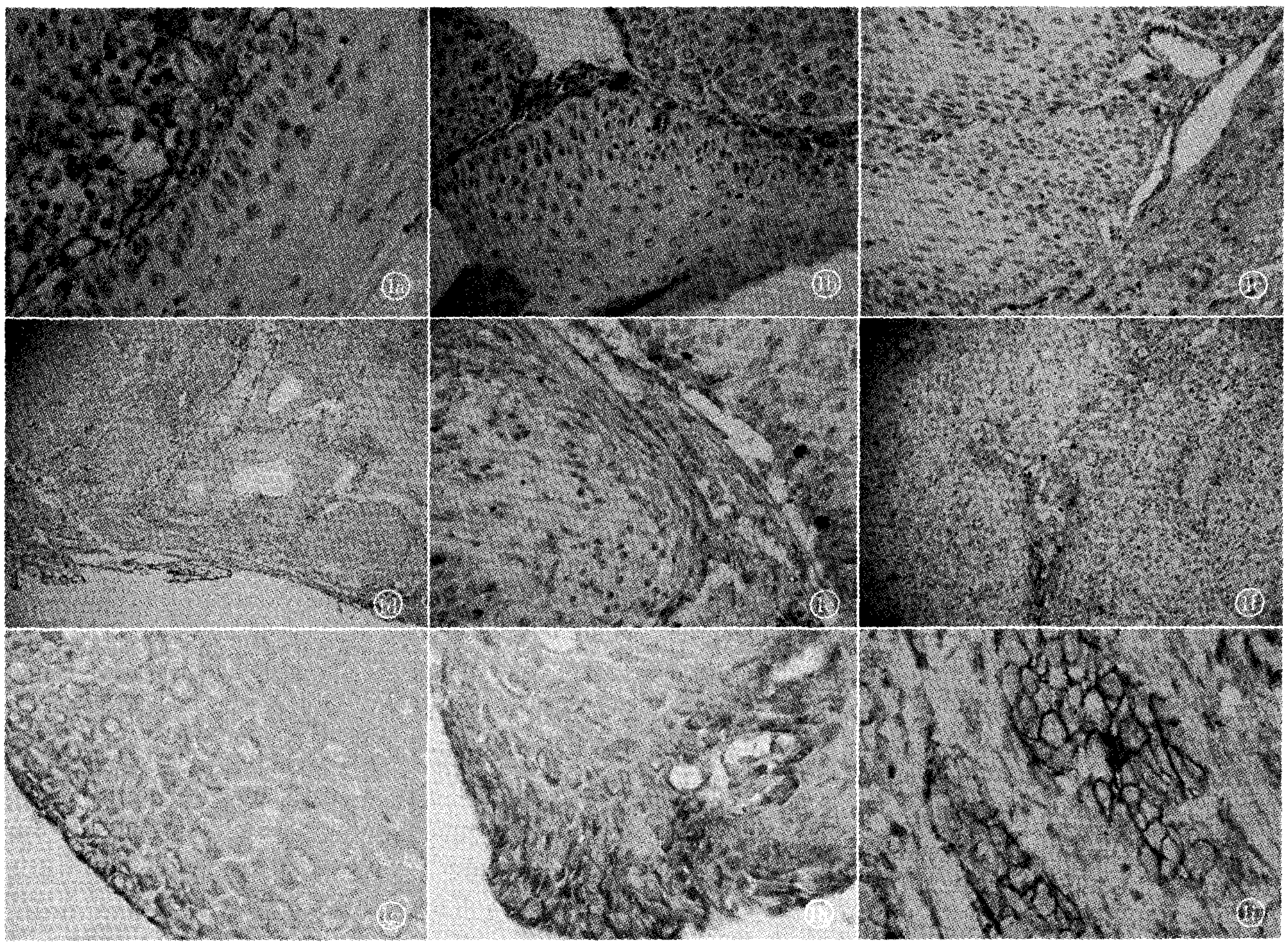

1a、1b:不典型增生食管组织FN、LN染色;1c、1d、1e、1f:食管癌FN、LN染色;1g、1h、1i:正常食管组织、不典型增生食管组织、食管癌EMMPRIN染色

图1 不同食管组织中FN、LN、EMMPRIN的表达 免疫组化S-P法

维普资讯 http://www.cqvip.com

表 1 基底膜 FN、LN 表达与食管癌的关系(例)

临床病理因素	例数	基底膜 FN		LN	
		(-)~(+)	(++)~(+++)	(-)~(+)	(++)~(+++)
病理分级					
Ⅰ级	25	6	19	2	23
Ⅱ级	50	35	15	4	46
Ⅲ级	12	11	1	9	3
临床分期					
Ⅱ期	19	16	3	15	4
Ⅲ期	68	42	26	39	29
淋巴结转移					
有	35	31	4	26	9
无	52	14	38	16	36

2.1.2 EMMPRIN 的表达：正常食管组织 EMMPRIN 阴性表达或仅见黏膜基底层细胞染色(图 1g)；不典型增生时见黏膜基底层细胞 EMMPRIN 染色(图 1h)；食管癌见细胞膜及细胞质 EMMPRIN 染色(图 1i)。正常食管组织、不典型增生食管组织和食管癌 EMMPRIN 的弱阳性表达率分别为 6.7%、55.0% 和 83.9%，强阳性表达率分别为 0、15.0% 和 52.9%，差异有统计学意义($P<0.01$)。

2.2 基底膜 FN、LN 表达与食管癌的关系

由表 1 可见，FN、LN 表达与食管癌病理分级、淋巴结转移有关($P<0.01$)，与临床分期无关($P>0.05$)。

2.3 EMMPRIN 表达与食管癌的关系

由表 2 可见，EMMPRIN 的弱阳性表达与食管癌的病理分级和临床分期无关($P>0.05$)，与淋巴结转移有关($P<0.01$)；强阳性表达与食管癌的病理分级、临床分期及淋巴结转移有关($P<0.01$)。

3 讨论

肿瘤的浸润与转移是一个多步骤、多因素参与的极其复杂的病理过程，有不同调节机制涉及这一过程，其中细胞外基质与肿瘤转移关系极为密切[1]。

目前已经证实，细胞外基质是阻止肿瘤转移的一道屏障。细胞外基质在上皮或内皮细胞的基底部，即以基底膜的形式存在，FN 和 LN 是基底膜的主要组成成分，在肿瘤发展过程中具有重要生物学意义。

表 2 EMMPRIN 表达与食管癌的关系(%)

临床病理因素	例数	EMMPRIN	
		弱阳性表达	强阳性表达
病理分级			
Ⅰ级	25	76.0	16.0
Ⅱ级	50	88.0	64.0
Ⅲ级	12	91.7	83.3
临床分期			
Ⅱ期	19	78.9	15.8
Ⅲ期	68	92.6	75.0
淋巴结转移			
有	35	91.4	80.0
无	52	67.3	23.1

FN 是具有多种功能的细胞外非胶原糖蛋白，在细胞黏附、迁移、生长和分化过程中发挥重要作用[2]，其进行性减少是人类肿瘤的特征之一[3]。体外实验中，肿瘤细胞表面 FN 减少或完全丧失均可使细胞间黏附力降低，细胞更易分离而浸润进入基底膜中。

肿瘤浸润转移时，肿瘤细胞与基底膜相互作用，尤其是细胞表面 LN 受体与 LN 的黏附是关键的一步，此后基底膜被胶原酶降解，癌细胞移行实现浸润。LN 受体可能通过增加Ⅳ型胶原蛋白的形式来促进 LN 与其结合，促进细胞转移、浸润。

基底膜由保留上皮细胞功能的癌变鳞状细胞分泌合成，癌细胞分化程度越高，其分泌能力越强，故高分化鳞状细胞癌的癌巢基底膜多完整，而低分化鳞状细胞癌的癌巢基底膜多表达为部分或全部缺失。本实验证实了癌巢基底膜 FN、LN 表达与食管癌细胞分化程度和浸润能力均相关。本实验还发现，癌巢基底膜 FN、LN 的表达与食管癌淋巴结转移之间有相关性($P<0.01$)。推测在转移过程中，癌细胞首先要突破其下方的基底膜至间质结缔组织中，再穿透脉管周边基底膜而进入脉管系统，故癌巢基底膜完整者较少出现淋巴结转移，而基底膜缺失情况下癌细胞较易穿透基底膜发生转移。

EMMPRIN 是新近发现的细胞表面黏附因子，主要表达于人类各种肿瘤细胞，介导细胞之间的黏附，具有刺激成纤维细胞分泌基质金属蛋白酶(MMP)的作用，特别是 MMP1、MMP2 和 MMP3 的合成，通过 MMP 降解细胞外基质和基底膜，促进癌细胞的侵袭和转移[4]。已有人用抗 EMMPRIN 特异性单克隆抗体注入到肝细胞癌异种移植物上，发现可显著抑制肿瘤的生长和远处转移[5]。已发现多种恶性肿瘤组织中均存在 EMMPRIN 过表达[6-7]。本实验发现，食管癌中也存在 EMMPRIN 的异常表达，不但表现在数量上，也表现在分布上。EMMPRIN 在正常食管组织仅

维普资讯 http://www.cqvip.com

有基底层细胞的表达，在食管癌中出现癌细胞的细胞膜及细胞质的阳性表达，在癌巢周边癌细胞向周围组织浸润处表达尤为强烈，肿瘤分化越差表达越强，且随着肿瘤分期的增高和淋巴结转移的存在，两者的表达均有增强的趋势，提示 EMMPRIN 的表达影响着食管癌的转移和预后。

FN、LN、EMMPRIN 表达可作为评价食管癌侵袭性及恶性程度的有用指标。转移是癌症患者死亡的主要原因，可从破坏癌细胞与细胞外基质的关系入手抑制肿瘤转移，如抑制细胞外基质的降解、抗黏附等。在今后的研究中可试用 FN 抗体、LN 抗体或封闭瘤细胞表面的 LN 受体、抗 EMMPRIN 抗体来防治恶性肿瘤复发和转移。

参 考 文 献

[1] 江忠清，朱凤川，曲军英，等．宫颈癌 MMP-9 表达与肿瘤血管生成、癌细胞增殖及侵袭转移的关系．癌症，2003，22(2):71-77.

[2] 雷振东，赵华，雷三林，等．基因 MMP-2、PTEN 和 Fn 的表达与胃癌临床病理因素的关系．生命科学研究，2006，10(3):265-269.

[3] 李辽源．纤维连接蛋白和膀胱肿瘤．国外医学．泌尿系统分册，2003，23(6):639-641.

[4] Sier CF, Zuidwijk K, Zijlmans HJ, et al. EMMPRIN-induced MMP-2 activation cascade in human cervical squamous cell carcinoma. Int J Cancer, 2006, 118(12):2991-2998.

[5] Chen ZN, Mi L, Xu J, et al. Targeting radioimmunotherapy of hepatocellular carcinoma with iodine (^{131}I) metuximab injection: clinical phase Ⅰ/Ⅱ trials. Int J Radiat Oncol Biol Phys, 2006, 65(2):435-444.

[6] Davidson B, Goldberg I, Berner A, et al. EMMPRIN (extracellular matrix metalloproteinase inducer) is a novel marker of poor outcome in serous ovarian carcinoma. Clin Exp Metastasis, 2003, 20(2):161-169.

[7] Vigneswaran N, Beckers S, Waigel S, et al. Increased EMMPRIN (CD 147) expression during oral carcinogenesis. Exp Mol Pathol, 2006, 80(2):147-159.

(收稿日期:2007-09-18 修回日期:2007-12-24)

·临床报道·

CD24 与原发性肝细胞癌侵袭转移的关系

潘群雄 庄建良 苏子剑 许荣誉 王聪仁

CD24 是一种低分子量高度糖基化的黏附分子。最近研究表明，CD24 参与肿瘤的侵袭和转移。本研究运用免疫组织化学检测肿瘤组织 CD24、增殖细胞核抗原(PCNA)表达情况。

1 资料与方法

1.1 资料

选取泉州市第一医院 2000—2004 年手术的原发性肝细胞癌手术切除标本 48 例，经 4% 中性甲醛固定，24 h 内取材，常规石蜡包埋，4 μm 厚度连续切片。所有患者术前未经放疗和化疗，所有病例均经切片病理确诊。其中男性 33 例，女性 15 例；小于 35 岁 16 例，大于 35 岁 32 例。病理分型：中-低分化 33 例，高分化 5 例。HBSAg 阴性 17 例，阳性 31 例。肿瘤直径小于 5 cm 18 例，大于 5 cm 30 例。甲胎蛋白小于 50 μg/L 17 例，大于 50 μg/L 31 例。有侵袭转移者 18 例，无侵袭转移者 30 例。

1.2 方法

1.2.1 免疫组织化学染色：CD24 浓缩型多克隆抗体购自 BioLegend，克隆号(SN3b)。按 1:50 进行稀释。PCNA 单克隆抗体(克隆号:PC10)购自福州迈新科技有限公司。用已知阳性片作阳性对照。同时用 PBS 代替第一抗体作阴性对照。

具体步骤如下：①石蜡切片常规经二甲苯脱蜡、梯度酒精水化后，用 PBS 冲洗 3 次，每次 5 min。②每张切片加 50 μl 过氧化物酶阻断溶液，以阻断内源性过氧化物酶的活性，在室温(25℃ ±2℃)下孵育 10 min；PBS 冲洗 3 次，每次 5 min。③CD24 采用 Tris-EDTA(pH 9.0)热抗原修复法-水浴法修复抗原：取一定量的 Tris-EDTA 抗原修复液于烧杯中，放入铝锅加盖，加热至锅中水沸腾，切片用蒸馏水洗 2 次之后，完全浸入 Tris-EDTA 抗原修复液中，继续加热 20 min，停止加热，从锅中取出烧杯，完全冷却后取出玻片，用 PBS 冲洗 3 次，每次 5 min。PCNA 采用柠檬酸缓冲液高温高压法：将柠檬酸盐缓冲液加热沸腾后，再将切片完全浸没于其中后加高压锅盖，继续加热至喷气，计时 1 min，停止加热，冷却至室温，取出玻片，用 PBS 冲洗 3 次，每次 5 min。④吸去多余液体，

基金项目：泉州市技术研究与开发基金资助项目(2007Z27)

作者单位：362000 泉州，福建医科大学附属泉州第一医院肿瘤外科
