# Supplementary material for: Expression of Concern: The prognostic and clinicopathologic characteristics of CD147 and esophagus cancer: A meta-analysis
Source: PLoS One. 2023 Feb 22;18(2):e0282229. doi: 10.1371/journal.pone.0282229 (PMC9946197; doi:10.1371/journal.pone.0282229)
Supplement: S1 File — (ZIP) [file pone.0282229.s001.zip › PDF of included paper/╩│╣▄┴█╫┤╧╕░√░⌐EMMPRIN╡░░╫╡─▒φ┤∩╝░┴┘┤▓╥Γ╥σ_╒┼╗▌╓╥.pdf]

·实验研究·

食管鳞状细胞癌 EMMPRIN 蛋白的表达及临床意义

张惠忠　李海刚　华平　王梅　魏益平　刘金耿　吴澄

【摘要】 目的　探讨食管鳞状细胞癌(ESCC)中细胞外基质金属蛋白酶诱导因子(EMMPRIN)蛋白的表达及临床意义。方法　免疫组织化学染色法检测 85 例 ESCC、18 例癌旁不典型增生(AH)、38 例癌旁"正常"鳞状上皮(NSEBC)和 15 例正常食管鳞状上皮(NSE)中 EMMPRIN 蛋白的表达。结果　ESCC、AH、NSEBC 和 NSE 中 EMMPRIN 蛋白的阳性率分别为 80%(68/85)、39%(7/18)、66%(25/38)和 20%(3/15);ESCC 中 EMMPRIN 蛋白阳性率高于非癌肿组织($P<0.01$, $r=0.35$)且阳性细胞分布区域不同;高、中度分化组 ESCC 中 EMMPRIN 蛋白阳性率高于低分化组($P<0.01$, $r=0.29$);ESCC 中 EMMPRIN 蛋白的表达与肿瘤浸润食管壁的深度、临床分期和淋巴结转移均无明显相关($P>0.05$)。结论　EMMPRIN 蛋白在 ESCC 组织的表达与在非癌肿组织的表达存在显著不同,且它与癌肿的组织分化程度有密切关系。

【关键词】 食管肿瘤;　金属蛋白酶类;　免疫组织化学;　转移

Expression and clinical significance of extracellular matrix metalloproteinase inducer in squamous cellular carcinoma of esophagus　*ZHANG Hui-zhong*, LI Hai-gang, HUA Ping, et al. * Department of Cardiothoracic Surgery, The Second Affiliated Hospital, Sun Yat-sen University, Guangzhou* 510120, China
Corresponding author: LI Hai-gang

【Abstract】 Objective　To investigate the expression and clinical significance of extracellular matrix metalloproteinase inducer (EMMPRIN) in squamous cellular carcinoma of esophagus (ESCC). **Methods**　The expression of EMMPRIN was detected By using immunohistochemistry in tissues of ESCC from 85 patients, of atypical hyperplasia (AH) from 18 patients, of "normal" squamous epithelium beside carcinoma (NSEBC) from 38 patients and of normal squamous epithelium (NSE) from 15 people. **Results**　The positive expression rates of EMMPRIN protein in ESCC, AH, NSEBC, NSE was 80% (68/85), 39% (7/18), 66% (25/38), 20% (3/15) respectively. The positive expression rates of EMMPRIN protein in ESCC was higher than in other non-cancer tissue ($P<0.01$, $r=0.35$) and the distribution area was different. The positive expression rates of EMMPRIN protein in well-differentiated group and moderately-differentiated group were significantly higher than in low-differentiated group ($P<0.01$, $r=0.29$). No relationship was found between the expression of EMMPRIN and the invasion, clinical stage, lymphoid metastasis ($P>0.05$). **Conclusion**　The expression of EMMPRIN protein in ESCC tissues was different from that in non-cancer tissues. The expression of EMMPRIN was closely related with the differentiation of ESCC.

【Key words】 Esophagus neoplasms;　Metalloproteinase;　Immunohistochemistry;　Metastasis

细胞外基质和基底膜的降解是恶性肿瘤浸润和转移发生过程中的重要步骤,多种酶参与此降解过程,其中金属蛋白酶的降解作用尤为重要。跨膜糖蛋白细胞外基质金属蛋白酶诱导因子(EMMPRIN)蛋白是基质金属蛋白酶(MMPs)的诱导剂,通过诱导 MMPs 的表达而影响恶性肿瘤的浸润和转移[1-3]。我们通过免疫组织化学染色检测食管鳞状细胞癌和非癌性鳞状上皮组织中 EMMPRIN 蛋白的表达情况,探讨 EMMPRIN 蛋白与食管鳞状细胞癌的发生、组织分化程度、肿瘤浸润食管壁的深度、淋巴结转移和临床分期等生物学行为的关系。

材料和方法

1. 材料:收集中山大学附属第二医院 2002 年 1 月至 2004 年 12 月行外科手术切除的食管鳞状细胞癌病例的标本 85 例。其中,男 67 例,女 18 例,年龄 35～77 岁(中位年龄 58 岁)。40 例手术时伴有淋巴结转移。所有病例手术前均未接受放化疗。85 例癌组织切片中,18 例伴有癌旁不典型增生、38 例伴有癌旁"正常"鳞状上皮(组织细胞形态上正常)。15 例正常食管

基金项目:广东省重点科技攻关项目(2003B30104)
作者单位:510120 广州,中山大学附属第二医院心胸外科(张惠忠、华平、王梅、魏益平、刘金耿、吴澄),病理科(李海刚)
通讯作者:李海刚

(C)1994-2022 China Academic Journal Electronic Publishing House. All rights reserved.　http://www.cnki.net

鳞状上皮组织取自因胃不适进行胃镜检查、食管肉眼形态正常的患者。

2.免疫组织化学染色:石蜡包埋组织切片,脱蜡水化,蒸馏水漂洗 5 min,作微波炉抗原修复。室温下冷却 30 min,磷酸盐缓冲液(PBS)漂洗 5 min,3% H_2O_2 室温下孵育 10 min,PBS 漂洗 3×5 min。滴加正常血清,37 ℃孵育 30 min。吸干血清后滴加第一抗体(即 EMMPRIN 抗体,购自北京中山公司,稀释度为 1∶70),4 ℃冰箱中过夜。第 2 天取出置室温 1 h 后,用 PBS 漂洗 3×5 min,滴加生化素标记的第二抗体,37 ℃孵育 30 min,PBS 漂洗 3×5 min。滴加链霉素卵蛋白生物素复合物,37 ℃孵育 30 min,PBS 漂洗 3×5 min,二氨基联苯胺显色 3～10 min。自来水漂洗,苏木精复染细胞核,中性树胶封片。用已知阳性的膀胱移行上皮癌切片作阳性对照,PBS 代替一抗作阴性对照。EMMPRIN 蛋白的阳性表达呈棕色位于细胞膜。

3.统计学方法:均采用秩和检验(Wilcoxon W、Mann-whitey U 和 Kruskal Wallis),Spearman 法计算相关系数。

结　果

1.非癌性鳞状上皮和食管鳞状细胞癌组织中 EMMPRIN 蛋白的表达:食管鳞状上皮、癌旁“正常”鳞状上皮、癌旁不典型增生组织 EMMPRIN 蛋白表达的阳性率分别为 20%(3/15)、66%(25/38)和 39%(7/18)。三组非癌组织的阳性细胞均局限于基底层,且数量较少,少于总细胞数的 5%(图 1～3)。

食管鳞状细胞癌组织 EMMPRIN 蛋白的阳性表达率为 80%(68/85),阳性细胞并不局限于基底层,其分布零乱、无规律性,且数量多(图 4)。在高倍镜下取 4 个不同视野各计数 200 个癌细胞,阳性细胞百分数为(32.2±26.2)%。

癌组织中 EMMPRIN 蛋白的阳性表达率明显高于正常食管鳞状上皮和癌旁不典型增生组织($P<0.05$);癌旁鳞状上皮 EMMPRIN 蛋白的阳性表达率明显高于正常食管鳞状上皮组织($P<0.05$);其余各组之间的阳性表达率差异无统计学意义($P>0.05$)。

2.EMMPRIN 蛋白表达与性别的关系:男、女性组食管鳞状细胞癌 EMMPRIN 蛋白阳性表达率分别为 79%(53/67)和 83%(15/18),差异无统计学意义($P>0.05$)。

3.EMMPRIN 蛋白表达与癌组织学分化程度的关系:高、中、低分化组食管鳞状细胞癌 EMMPRIN 蛋白阳性表达率分别为 88%(35/40)、86%(24/28)和 53%(9/17)。EMMPRIN 蛋白的表达与癌组织分化程度呈负相关($Z=-2.446, P<0.05, r=0.29$)。

4.EMMPRIN 蛋白表达与癌肿食管壁浸润深度的关系:未浸润食管壁肌层的食管鳞状细胞癌 EMMPRIN 蛋白阳性表达率为 75%(6/8);浸润肠壁浅肌层的阳性表达率为 100%(5/5);浸润肠壁深肌层的阳性表达率为 86%(10/12);浸润食管壁外层的阳性表达率为 78%(47/60)。EMMPRIN 蛋白的表达与癌浸润深度无明显相关($P>0.05$)。

5.EMMPRIN 蛋白表达与癌淋巴结转移的关系:伴淋巴结转移组食管鳞状细胞癌 EMMPRIN 蛋白阳性表达率为 83%(33/40),没有淋巴结转移组阳性表达率为 78%(35/45)。EMMPRIN 蛋白的表达与食管鳞状细胞癌的淋巴结转移无明显相关($P>0.05$)。

6.EMMPRIN 蛋白的表达与临床分期的关系:临床Ⅰ、Ⅱ、Ⅲ、Ⅳ期的食管鳞状细胞癌组织 EMMPRIN 蛋白阳性表达率分别为 80%(4/5)、79%(34/43)、81%(26/32)和 80%(4/5)。EMMPRIN 蛋白的表达与食管鳞状细胞癌的临床分期无明显相关($P>0.05$)。

讨　论

有研究结果表明,多种恶性肿瘤组织中均存在 EMMPRIN 蛋白的过度表达[1-3]。本研究结果显示,食管鳞状细胞癌中也存在 EMMPRIN 蛋白的异常表达,癌组织这种异常表达不但表现为数量上的过度表达,且表现为阳性细胞分布上的不同,即 EMMPRIN 蛋白在癌组织中呈弥漫无规律性的零乱分布,而在非癌组织中仅分布于基底层,由此提示非癌组织中只有基底层的细胞产生 EMMPRIN 蛋白,而癌组织则普遍产生 EMMPRIN 蛋白,癌与非癌组织中 EMMPRIN 蛋白的表达存在本质上的差异。本研究结果显示,癌旁“正常”鳞状上皮 EMMPRIN 蛋白的阳性表达率明显高于正常食管鳞状上皮,提示癌旁的鳞状上皮虽然形态学上仍然保持正常,但其 EMMPRIN 蛋白的表达已明显增多,只是阳性细胞的分布还与正常食管鳞状上皮相同而有别于癌组织,这反映出此时癌旁“正常”鳞状上皮可能正处于“量变”阶段。

本研究结果显示,高、中度分化组鳞状细胞癌组织 EMMPRIN 蛋白的阳性表达率明显高于低分化组,表明 EMMPRIN 蛋白的阳性表达与食管鳞状细胞癌的组织分化程度呈正相关。本研究结果还显示,食管鳞状细胞癌的食管壁的浸润深度、淋巴结转移和临床分期均与 EMMPRIN 蛋白的表达无明显相关。这可能是因为,EMMPRIN 蛋白虽为细胞外 MMPs 的诱导剂,但其诱导产生的 MMPs 对肿瘤组织的作用还受到

(C)1994-2022 China Academic Journal Electronic Publishing House. All rights reserved. http://www.cnki.net

多因素的制约，如受到组织基质金属蛋白酶抑制剂（TIMPs）的调控。

（本文图 1～4 见封三）

参 考 文 献

1 陈雷，林建华，张声. 基质金属蛋白酶-9 和组织金属蛋白酶抑制剂-1 在骨肉瘤中的表达及其意义. 中华实验外科杂志，2004，21：1522-1523.

2 肖春花，冯玉梅，李晓青，等. 基质金属蛋白酶-2 和纤维粘连蛋白在乳腺癌细胞系的定量表达. 中华实验外科杂志，2005，22：527-529.

3 杨锦建，张秋红，贾占奎，等. 基质金属蛋白酶（MMP）-9、MMP-2 mRNA 及基质金属蛋白酶抑制剂 2 mRNA 在人膀胱移行细胞癌中的表达及意义. 中华实验外科杂志，2006，23：594-595.

（收稿日期：2006-04-19）

·简报·

脑出血后成年大鼠神经干细胞移植的研究

张弩 周辉 杨林

近年来，胚胎来源的神经干细胞移植治疗脑出血的研究已有报道[1]，我们通过对大鼠脑出血模型进行成年神经干细胞移植治疗，观察出血部位移植神经干细胞的生长情况。

一、材料与方法

1. 神经干细胞的分离、培养和鉴定：成年雄性大鼠腹腔麻醉后处死，显微镜下仔细分离出海马组织。采用机械消化法，使组织体积逐渐减小直至形成单细胞悬液。加入神经干细胞全培液，置于 37 ℃，5% CO_2 培养箱中悬浮培养。5～7 d 机械分离克隆传代 1 次。第 3 代细胞大部分继续培养 3 d，少量的神经球，将其移入铺有 L-多聚赖氨酸的盖玻片上，培养液为含有 10% 胎牛血清的 DMEM/F12。3 d 后，丙酮固定。应用 nestin 抗体进行细胞免疫组织化学染色。

2. 神经干细胞的 5-溴脱氧嘧啶（BrdU）标记：BrdU 与胸腺嘧啶结构相似，BrdU 在 DNA 合成过程中能与胸腺嘧啶一样掺入其中，应用 BrdU 单克隆免疫组织化学可以检测培养细胞的增殖情况，本实验用来检测神经干细胞在移植区域的生长情况。

3. 大鼠脑出血模型制作：SD 大鼠，选右尾状核为注血部位，坐标为前囟前 0.4 mm，矢状缝旁 3 mm，距脑表面深 5 mm；用大鼠脑立体定向仪，以上面坐标为中心颅骨钻孔，以颅骨外板为零点将头皮针垂直插入脑内，进针深度为 6 mm，100 微升微量进样器取非肝素化尾动脉血 75 μl 缓慢注入尾状核内，留针 10 min 后缓慢拔针，骨蜡封骨孔后缝合头皮。

4. 大鼠神经功能评分：采用 Longa[2] 法。

5. 实验动物分组：模型制作后 3 d，将制作成功的 24 只大鼠随机分为 NSCs 移植组 12 只和对照组 12 只：移植组使用 BrdU 标记神经干细胞，浓度为 2×10^7 细胞/100 μl。原骨孔处头皮针垂直插入脑内，分别在进入 5 mm、6 mm 和 7 mm 处注入细胞悬液 10 μl，留针 10 min 后拔针。分别在移植后 1 d 和 4 周后处死，灌注固定，取脑行 BrdU 标记的检测。

二、结果

1. 来源于成年大鼠海马的神经干细胞能在体外大量增殖、长期传代。细胞的免疫组织化学染色鉴定：（1）神经球 nestin 鉴定：免疫荧光染色呈阳性，神经球贴壁生长后神经元细胞逐渐向四周迁移，表现出成熟神经细胞的形态。（2）神经球分化鉴定：神经球分化后神经元和胶质细胞特异性标志物阳性，免疫荧光染色部分细胞呈 NF 阳性。

2. 脑组织切片免疫组织化学染色：显示在血肿周围可见到 BrdU 及 nestin 阳性细胞并且在血肿灶周围聚集融合。

3. 大鼠运动功能评分：模型制作后 2 h 及移植后 1 d，二组间运动功能比较差异无统计学意义（$P>0.05$）；移植后 28 d 与模型制作后 2 h，比较差异有统计学意义（$P<0.01$），即运动功能有显著的改善，移植后 28 d 与对照组比较，差异有统计学意义（$P<0.05$）。

三、讨论

近年来，对成年神经干细胞的研究不多，主要是由于：首先，成年神经干细胞在大脑内的含量很少而且数量随年龄增长而降低；分离和纯化困难；而且体外培养存活率较低。目前只是在癫痫患者和开放性脑损伤的患者的手术中才有可能分离到神经干细胞，为其临床应用带来困难。其次，从患者体内分离的神经干细胞，并进行体外培养，需要足够数量的细胞才可能用于治疗。而且在细胞培养中，还不能完全控制神经干细胞的定向分化，同时在细胞培养时也可能出现一些遗传错误或者基因突变，因而这样的神经干细胞不适于细胞移植治疗。这些潜在的弱点将限制成体干细胞的使用。本实验通过对移植的 BrdU 标记的成年大鼠海马神经干细胞进行免疫组织化学鉴定，证实 NSCs 能在移植区成活，通过对神经系统评分显示能改善神经功能缺损的症状。

参 考 文 献

1 杨林，赵洪洋，赵甲山，等. 体外标记神经干细胞脑内移植治疗大鼠创伤性脑损伤. 中华实验外科杂志，2006，23：209-211.

2 Longa EZ, Weinstein PR, Carlson S, et al. Reversible middle cerebral artery occlusion without craniectomy in rats. Stroke, 1989, 20:84-91.

（收稿日期：2006-03-09）

基金项目：温州市科技局资助项目（Y2003A024）

作者单位：325027 浙江温州医学院附属第二医院神经外科（张弩、周辉）；华中科技大学同济医学院附属协和医院神经外科（杨林）

(C)1994-2022 China Academic Journal Electronic Publishing House. All rights reserved. http://www.cnki.net

（接上页）

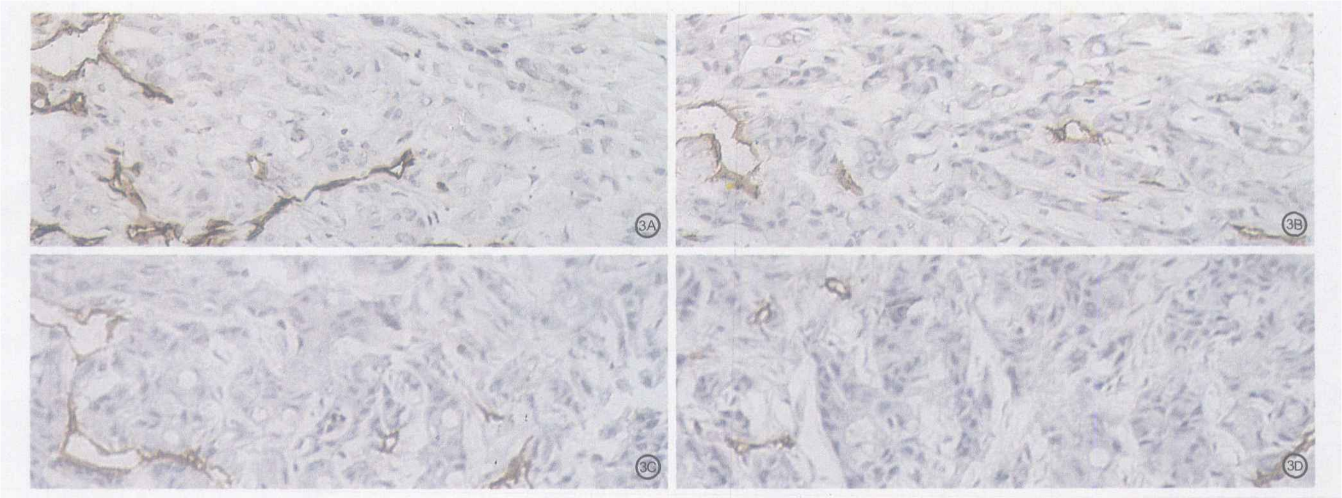

图 3A 肿瘤单位面积微血管测定 对照组 **图 3B** 肿瘤单位面积微血管测定 3TSR 组 **图 3C** 肿瘤单位面积微血管测定 Gem 组
图 3D 肿瘤单位面积微血管测定 3TSR + Gem 组

腺病毒介导的人血管抑素基因治疗胰腺癌

（正文见第 1234 页）

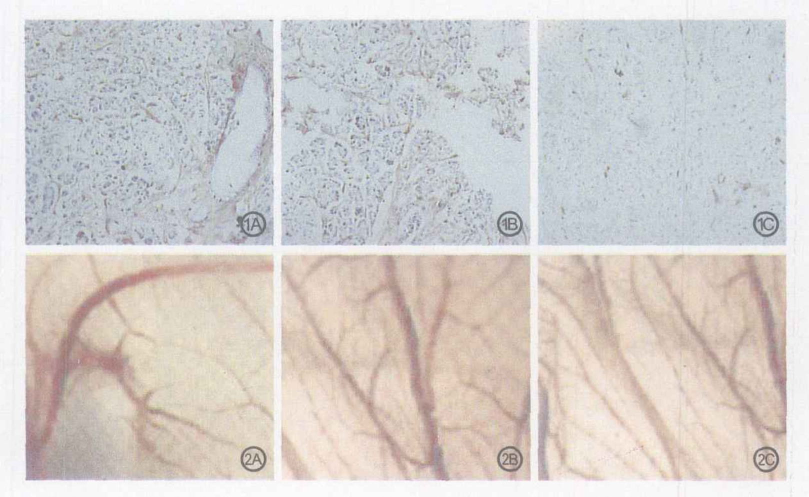

图 1 鸡胚绒毛膜尿囊膜检测实验（CAM） 1：治疗组 Ad-hA；2：对照组 Ad-LacZ；3：对照组 Ad-buffer

图 2 免疫组织化学染色观察治疗组与对照组微血管密度（PV900 法 × 100） 1：对照组 Ad-LacZ（×100）；2：对照组 Ad-buffer（×100）；3：Ad-hA 治疗组（×100）

食管鳞状细胞癌 EMMPRIN 蛋白的表达及临床意义

（正文见第 1248 页）

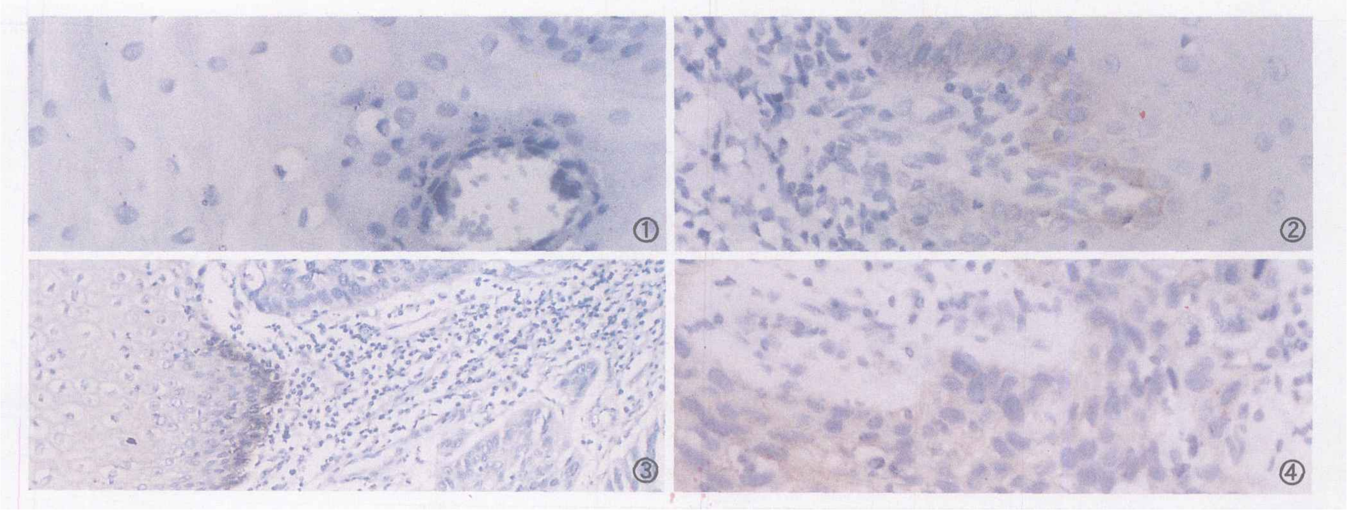

图 1 正常食管鳞状上皮组织 EMMPRIN 蛋白呈阴性表达（SP×200） **图 2** 癌旁“正常”鳞状上皮组织 EMMPRIN 蛋白的阳性表达（SP×200）
图 3 癌旁不典型增生组织 EMMPRIN 蛋白的阳性表达（SP×200） **图 4** 食管鳞状细胞癌组织 EMMPRIN 蛋白阳性表达（SP×200）

(C)1994-2022 China Academic Journal Electronic Publishing House. All rights reserved. http://www.cnki.net
