# Supplementary material for: Expression of Concern: The prognostic and clinicopathologic characteristics of CD147 and esophagus cancer: A meta-analysis
Source: PLoS One. 2023 Feb 22;18(2):e0282229. doi: 10.1371/journal.pone.0282229 (PMC9946197; doi:10.1371/journal.pone.0282229)
Supplement: S1 File — (ZIP) [file pone.0282229.s001.zip › PDF of included paper/╩│╣▄┴█╫┤╧╕░√░⌐╓╨MMP-9íóCD-147╡─▒φ┤∩╝░╥Γ╥σ_╨▄╦╔░╪.pdf]

DOI:10.13241/j.cnki.pmb.2007.01.014

食管鳞状细胞癌中 MMP-9、CD-147 的表达及意义

熊松柏 徐 义 蒋 彬 朱小星 杨 康

(第三军医大学第一附属医院胸心外科 重庆 400038)

摘要 目的: 探讨基质金属蛋白酶 9(MMP-9)和 CD-147 在食管鳞状细胞癌的表达及其与肿瘤浸润转移的关系。**方法:** 应用免疫组化 S-P 法观察 57 例食管鳞癌组织中 MMP-9、CD-147 的表达,并探讨其与食管鳞癌临床病理资料的关系。**结果:** MMP-9、CD-147 在癌组织中的阳性表达率分别为 82.46%,64.91%; 在癌旁组织中的阳性表达率分别是 29.82%,8.77%。在食管鳞癌中,MMP-9 及 CD-147 的表达均与食管鳞癌的浸润深度有关,与分化程度无明显关联;有淋巴结转移的病例阳性表达率明显高于无淋巴结转移组。**结论:** MMP-9 及 CD-147 的表达均与食管鳞癌的浸润深度及淋巴结转移有关。

关键词: 食管肿瘤;基质金属蛋白酶 -9;CD-147;免疫组化

中图分类号: R655.4 **文献标识码:** A **文章编号:** 1673-6273(2007)01-0042-02

Expression of Matrix Metalloproteinases -9 and CD-147 in Human Esophageal Squamous Cell Carcinoma

XIONG Song-bai, XU Yi, JIANG Bing, ZHU Xiao-xing, YANG Kang

(Department of Cardiothoracic Surgery, The First Affiliated Hospital, The Third Military Medical University, Chongqing 400038)

ABSTRACT Objective: To investigate the expression of matrix metalloproteinase-9 (MMP-9) and CD-147 in human esophageal squamous cell carcinoma (ESCC). **Methods:** The expression of MMP-9 and CD-147 was detected in 57 patients with ESCC by immunohistochemical S-P method. **Results:** The expression of MMP-9 and CD-147 had a significant difference between esophageal squamous cell carcinoma and esophageal epithelial cells from normal tissues adjacent to the tumours, and had a significant relevance to infiltrating depth and lymph node metastasis, but no association was observed with pathological grades. **Conclusion:** Both MMP-9 and CD-147 are all related to infiltrating depth and lymph node metastasis of esophageal squamous cell carcinoma.

Key words: Esophageal carcinoma; Matrix metalloproteinase-9; CD-147; Immuneohistochemistry

Chinese Library Classification(CLC): R775.1 Document code:E

Article ID: 1673-6273(2007)01-0042-02

食管鳞状细胞癌是消化道常见恶性肿瘤,多数患者就诊时已有深度浸润及转移,死亡率很高。细胞外基质(Extracell Matrix, ECM)是阻碍肿瘤细胞侵袭转移的天然屏障,癌细胞必须穿透 ECM 才能向外侵袭[1],因此癌细胞产生能降解细胞外基质的酶与食管鳞状细胞癌的转移密切相关。ECM 主要由Ⅳ型胶原组成,而基质金属蛋白酶 -9 (Matrix Metalloproteins9, MMP9)是降解Ⅳ型胶原最主要的酶,CD-147 由于刺激 MMP-9 的产生也有利于肿瘤细胞的转移,本研究探讨 MMP-9 及 CD-147 在食管鳞癌中的表达与临床分期,分级及预后的关系。

1 材料与方法

1.1 临床资料

本组 40 例食管鳞状细胞癌标本及癌旁正常组织标本来自第三军医大学第一附属医院心胸外科 2005 年 7 月至 2006 年 3 月手术切除组织标本,标本均经我院病理科常规病理证实。其中男 44 例,女 13 例,年龄 36~75 岁,平均 55.6 岁。病理分级:中、高分化 41 例,低分化 16 例,临床分期根据 UICC1997 年标准:Ⅰ期 3 例,Ⅱ期 46 例,Ⅲ期 8 例,15 例区域淋巴结转移。全部病例未发现有远处转移。主要症状为进行性吞咽困难。所有患者术前均未做化疗和放疗。手术时取所需标本,经 10%中性福尔马林固定,石蜡包埋,连续 4μ m 切片。

1.2 免疫组织化学方法

免疫组织化学方法选用 S-P 法,一抗为 MMP-9 及 CD-147 鼠抗人单克隆抗体,购自 SANTA CRUZ 公司。S-P 试剂盒、浓缩型 DAB 试剂盒及胃蛋白酶消化液购自北京中杉金桥生物技术有限公司。试验步骤为:石蜡切片常规脱蜡至水,3%过氧化氢孵育 5~10 分钟清除内源性抗原,蒸馏水冲洗,PBS 浸泡 3 分钟× 3 次,抗原修复(MMP-9 需要胃蛋白酶消化),PBS 浸泡 3 分钟× 3 次,滴加封闭用正常山羊血清室温孵育 15 分钟,倾去勿洗,滴加一抗 4 ℃过夜,PBS 浸泡 3 分钟× 3 次,滴加二抗,37℃孵育 15 分钟,PBS 浸泡 3 分钟× 3 次,滴加辣根酶标记链霉素工作液,DAB 显色,苏木素复染,中性树胶封片。用缓冲液 PBS 代替一抗作阴性对照,已知乳腺癌阳性标本作阳性对照。

结果判断标准:MMP-9 以胞质内出现棕黄色颗粒,CD-147 以胞膜及胞质呈棕黄色染色,强度高于背景非特异性染色者为阳性。按阳性细胞所占百分比进行分析:阴性(-):无阳性细胞染色;弱阳性(+):阳性癌细胞小于癌细胞总数 50%或显色浅;强

作者简介:熊松柏,(1975.7),男,硕士,
E-MAIL:songbaixiong@yahoo.com.cn Tel: 13389665028
通信作者:杨康,男,副教授,硕士生导师,
主要研究方向:食管癌的基础与临床。Tel:023-68754683
(收稿日期:2006-10-10 接受日期:2006-11-03)

(C)1994-2022 China Academic Journal Electronic Publishing House. All rights reserved. http://www.cnki.net

阳性(++):阳性细胞大于 50%或显色深。

1.3 统计学方法

研究中数据处理在 SPSS 13.0 统计软件包上完成。统计方法采用 x^2 检验等进行统计学处理,$P<0.05$ 认为具有统计学意义。

2 结果

2.1 组织学观察结果

食管鳞癌组织呈巢状分布，为多少不等的纤维组织分隔，癌细胞呈多角形,细胞边界较清楚,细胞核呈圆形或卵圆形,位于细胞中央,深染。根据分化程度,鳞状细胞癌可分为高分化、中分化、低分化。高分化鳞癌细胞有明显的角化现象,胞质丰富,核分裂像不多,细胞多形性不明显;低分化鳞癌细胞不见角化,癌细胞呈梭形或长椭圆形,核分裂像多,细胞多形性明显;中分化鳞癌的组织形态界于高分化和低分化鳞癌之间。

2.2 免疫组化显色结果

MMP-9 及 CD-147 在食管鳞癌及癌旁组织中的表达结果(表 1):MMP-9 及 CD-147 在癌组织中的阳性表达率分别为 82.46%,64.91%；在癌旁组织中的阳性表达率分别是 29.82%, 8.77%,MMP-9 及 CD-147 在癌组织中的阳性表达率显著高于癌旁组织的阳性表达率($P<0.01$)。

MMP-9 及 CD-147 在食管鳞癌中的表达结果见表 2。MMP-9 在低分化食管鳞癌癌细胞及中、高分化鳞癌中的阳性表达率分别为 87.5 %和 80.49%，低分化与中高分化组阳性表达率相比有显著差异($P>0.05$)。

MMP-9 及 CD-147 在食管鳞癌中的表达与食管鳞癌患者的性别、年龄、部位、分化程度等无关($P>0.05$)。

MMP-9 及 CD-147 在食管鳞癌中的表达与肿瘤浸润深度的关系见表 2:MMP-9 在黏膜及黏膜下层与肌层、外膜层的阳性表达率分别是 33.33%,85.19%有显著性差异 ($P<0.05$)。CD-147 在黏膜及黏膜下层与肌层、外膜层的阳性表达率分别是 0,68.52%有显著性差异($P<0.05$)。

MMP-9 及 CD-147 在食管鳞癌中的表达与淋巴结的转移关系(表 2):有淋巴结转移的癌组织 MMP-9 及 CD-147 的阳性表达率均明显高于无淋巴结转移组($P<0.05$)

表 1 MMP-9 与 CD-147 在食管癌组织及癌旁组织中的表达及关系

Table 1 The expression of MMP-9 and CD-147 in esophageal squamous cell carcinoma and esophageal epithelial cells from normal tissues adjacent to the tumours

	MMP-9						CD-147				
	n	-	+	++	阳性率(%)	P	-	+	++	阳性率(%)	P
癌旁组织	57	40	14	3	29.82	0.000	52	5	0	8.77	0.000
癌组织	57	10	26	21	82.46		20	17	20	64.91	

表 2 MMP-9 和 CD-147 在食管鳞癌中的表达

Table 2 The expression of MMP-9 and CD-147 in esophageal squamous cell carcinoma

		MMP-9						CD-147				
		n	-	+	++	阳性率%	P	-	+	++	阳性率%	P
性别	男	44	8	19	17	81.82	0.81	15	12	17	65.91	0.772
	女	13	2	7	4	84.62	6	5	5	3	61.54	
年龄	≥ 60 岁	27	3	12	12	88.89	0.24	7	7	13	74.07	0.169
	<60 岁	30	7	14	9	76.67	9	13	10	7	56.67	
部位	食管上、中段	32	6	14	12	81.25	0.78	12	7	13	62.5	0.666
	食管下段	25	4	12	9	84.	6	8	10	7	68	
浸润深	黏膜及黏膜下	3	2	1	0	33.33	0.02	3	0	0	0	0.016
度	肌层和外膜层	54	8	25	21	85.19	2	17	17	20	68.52	
分化程	中高分化	41	8	21	12	80.49	0.53	14	13	14	65.85	0.812
度	低分化	16	2	5	9	87.5	2	6	4	6	62.5	
淋巴结	有	15	0	5	10	93.33	0.03	1	6	8	93.33	0.026
转移	无	42	10	21	11	78.57	7	19	11	12	54.76	

3 讨论

MMP-9 作为 MMPs 家族的重要成员，是降解基膜成分Ⅳ型胶原纤维的主要酶。正常生理状态下,MMP-9 参与细胞外基质的代谢,在胚胎发育、骨组织改建、排异及损伤愈合中发挥着作用;在肿瘤组织中其活性异常增高,对肿瘤浸润及转移起着重要作用 [2]。在人体肿瘤研究中发现多种肿瘤 [3~5] 中存在 MMP-9 高表达现象。在本实验中 MMP-9 在食管癌旁组织中的表达为 29.82%,远远低于癌组织的 82.46%,且随着癌组织向食管壁浸润深度的增加，肿瘤细胞分化程度的降低，癌细胞 MMP-9 的阳性表达明显增高;而且淋巴结转移阳性组 MMP-9 表达明显高于阴性组。这与 Davies 等[6]在膀胱癌的研究中结果一致,即 MMP-9 在膀胱癌中的表达与肿瘤的浸润有关,浸润性肿瘤比浅表性肿瘤 MMP-9 表达明显增高;Bo 等[7]利用 KA II /CD82 抑制 H1299 人非小细胞癌细胞株 MMP-9 mRNA 的表

（下转第 47 页）

(C)1994-2022 China Academic Journal Electronic Publishing House. All rights reserved. http://www.cnki.net

癌中，Survivin 和 Bcl-2 可能协同发挥癌细胞的抗凋亡作用，促使结肠癌细胞的形成和扩散。

本研究发现 Survivin 的表达与 P53 的表达无显著相关性（P>0.05）。虽然 Survivin 和 P53 在细胞的增生和凋亡过程中都属于关键性的调节因子，有些报道也提示在胃癌和胰腺癌中两者的表达有显著相关性[2,5]，但是到目前为止还没有足够证据表明在大肠癌中两者在蛋白表达机制上有相互协同作用。

Survivin 的表达与肿瘤的预后有关，高表达者预后差。细胞凋亡受抑制使得肿瘤细胞得以继续存活并通过血流转移到其他部位，从而可以促进肿瘤的进展。Sarela 等[6]发现 Survivin mRNA 的高表达与患者因大肠癌复发致死率呈正相关。本研究结果显示 Survivin 与生存密切相关，Survivin 阳性组患者的 5 年生存率明显低于阴性组（P=0.001）。

4 结论

凋亡抑制基因的参与是肿瘤发生发展的一个重要环节，Survivin 作为一个独特的凋亡抑制基因，高选择性表达在肿瘤组织中，使得其有望作为一个较为普遍的肿瘤标志物用于肿瘤的早期诊断，也有望成为肿瘤靶向治疗的新靶点。检测结肠癌中 Survivin 对于临床诊治和预后判断都有重要价值。

参考文献(References)

[1] YASUMORI N, SHUICHI Y, MAKI H, et al. Differential expression of surviving in bone marrow cells from patients with acute lymphocytic leukemia and from patients with lyphocytic leukemia [J]. Leukemia Res, 2004, 28(5):487-94

[2] SWANA H, GROSSMAN D, ANTHONY J, et al. Tumor content of the anti- apoptosis molecule surviving and recurrence of bladder cancer[J]. N Engl J Med, 1999,341:452-3

[3] KAWASAKI H, ALTIERI D, LU C, et al. Inhibition of apoptosis by survivin predicts shorter survival rates in colorectal cancer [J].Cancer Res, 1998, 58:5071-4

[4] SARELA A, SCOTT N, RAMSDALE J, et al. Immunohistochemical detection of the antiapoptosis protein, surviving, predicts survival after curative resection of stage II colorectal carcinomas [J]. Ann Surg Oncol, 2001, 8:305-10

[5] LU C-D, ALTIERI D, TANIGAWA N. Expression of novel antiapoptosis gene, surviving, correlated with tumor cell apoptosis and p53 accumulation in gastric carcinomas[J]. Cancer Res, 1998,58:1808-12

[6] SARELA A, MACUDAM R, FARMERY S, et al. Expression of the antiapoptosis gene, surviving, predicts death from recurrent colorectal carcinoma [J]. Gut, 2000, 46:645-50

（上接第 44 页）

达后，癌细胞的侵袭与转移显著降低。MMP-9 作为一种基质金属蛋白酶，对肿瘤的恶性演变过程具有影响。

CD-147 属于免疫球蛋白超家族(IgSF)[8]，它是一种新的细胞表面黏附分子，介导细胞与细胞、细胞与间质的黏附作用[9]。CD-147 参与人体的多种生理活动，如胚胎着床、子宫复旧、及伤口愈合[10]等，但其在肿瘤侵袭和转移中的作用更值得关注。CD-147 作为细胞外基质金属蛋白酶刺激物，具有刺激肿瘤细胞及周围间质成纤维细胞分泌Ⅳ型胶原酶的能力，促进肿瘤细胞的转移[11]。本组结果显示：在食管癌组织中 CD-147 的阳性表达率为 64.91%，癌旁组织的阳性表达率为 8.77%，两者有显著差异（P＜0.01）；随着癌组织浸润深度的增加及淋巴结的转移 CD-147 的表达亦增强（P＜0.05），但是 CD-147 与患者性别、年龄、肿瘤生长部位、及肿瘤的分期无明显相关，提示 CD-147 的表达可能与肿瘤的转移相关。细胞外基质中 CD147 与 MMP-9 多同时表达，两种蛋白可能协同作用共同促进细胞外基质恶性细胞的增殖和侵袭转移。二者可能作为衡量肿瘤恶性程度的潜在指标，为肿瘤诊断、评价预后及化学治疗提供了一个新的思路。而 MMP 抑制剂已进入三期临床阶段，作为其上游分子的 CD147，相信阻断其表达或功能将更有效地抑制肿瘤的发展。

参考文献(References)

[1] RAY J M, STEVENSON W G. The role of matrix metalloproteinases and their in tumor invasion, metastasis and angiogenesis [J]. Eur J Respir dis, 1994, 7: 2062-2072

[2] 王璐，张丽红，李玉林，等. 基质金属蛋白酶-9 及其 mRNA 在胃癌中的表达与血管新生的关系 [J]. 中华医学杂志, 2003, 83(9): 782-786
WANG LU , ZHANG LI-HONG, LI YU-LIN, et al. Expression of MMP-9 and MMP-9 mRNA in gastric carcinoma and its correlation with angiogenesis[J]. Chinese Medical Journal, 2003, 83(9): 782-786

[3] ZENG ZS, GUILLEM JG. Distinct pattern of matrix metalloproteinase-9 and tissue inhibitor of metaloppoteinase-1 mRNA expression in human colorectal cancer [J]. Br J Cancer, 1995, 72: 575-582

[4] 张欣欣，郭永清，叶青，等. 基质金属蛋白酶 MMP-9 和 MMP-2 与鼻咽癌转移的相关性研究 [J]. 临床耳鼻咽喉科杂志,1999,13(8): 356-358
ZHANG XIN-XIN, GUO YONG-QING, YE QING, et al. The significance of matrix metalloproteinase_9 and tissue inhibitor of metalloproteinase_1 expression in laryngeal carcinoma [J]. J Clin Otorhinolaryngol(China),1999,13(8): 356-358

[5] EDWARDS JG, MCLAREN J, JONES JL, et al. Matrix metalloproteinases-2 and 9 (gelatinases A and B) expression in malignant mesotelioma and benign pleura[J]. Br J Cancer, 2 003, 88(10): 1553

[6] SHEKHAR MPV, WERDELL J, SANTNER SJ, et al. Brast stroma plays a dominant regulatory role in breast epithelial growth and differentiation; implication for tumor development and progression [J]. Cancer Res, 2001, 61(4): 1320-1326

[7] BO KEUN JEE, KOUNG MIN PARK, SIBIN SURENDRAN, et al. KAI1/CD82 suppresses tumor invasion by MMP9 inactivation via TIMP1 up-regulation in the H1299 human lung carcinoma cell line [J]. Biochem Biophys Res Commun, 2006, 34 (2) : 655~661

[8] BISWAS C, ZHANG Y, DECASTROR, et al. The human tumor cell-derived collagenase-stimulatory factor (renamed EMMPRIN) is am ember of the immunoglobulin superfamily [J]. Cancer Res, 1995, 55(2): 434-439

[9] SUZUKI S, SATO M, SENOO H, et al. Directcell-cell interaction enhances Pro-MMP-production and activatation in co-culture of laryngeal cancer cells and fibroblasts: involvement of EMMPRIN and MT1-MMP[J]. Exp Cell Res, 2004, 293(2): 259-266

[10] GUO H, LI R, ZUCKER S, et al. EMMPRIN (CD147), an inducer of matrix metalloproteinase synthesis, also binds interstitial collagenase to the tumor cells surface[J]. Cancer Res, 2000, 60(4): 888-891

[11] ERIC EG, THANH HX, ALAIN M, et al. EMMPRIN/CD147 an MMP modulator in cancer, development and tissue repair [J]. Biochimie, 2005, 87 : 361~368

(C)1994-2022 China Academic Journal Electronic Publishing House. All rights reserved. http://www.cnki.net
