# Supplementary material for: Expression of Concern: The prognostic and clinicopathologic characteristics of CD147 and esophagus cancer: A meta-analysis
Source: PLoS One. 2023 Feb 22;18(2):e0282229. doi: 10.1371/journal.pone.0282229 (PMC9946197; doi:10.1371/journal.pone.0282229)
Supplement: S1 File — (ZIP) [file pone.0282229.s001.zip › Supplementary data/Figure legend-Supplementary Begg's Plot.docx]

**Supplementary Begg’s Plot:**

Begg’s test was used to assess publication bias (P < 0.05 was considered statistically significant). If publication bias was confirmed, a trim-and-fill method developed by Duval and Tweedie was implemented to adjust for this bias.

A The publication bias between CD147 positive expression and 3-year survival rate. No publication bias was found with p=1.000.

B The publication bias between CD147 positive expression and 5-year survival rate. No publication bias was found with p=1.000.

C The publication bias between cancer and noncancer tissues. Publication bias was found with p=0.024.

D The publication bias between cancer and normal tissues.No publication bias was found with p=1.000.

E The publication bias between cancer and para-carcinoma tissues.No publication bias was found with p=0.074.

F The publication bias between cancer and hyperplastic tissues.No publication bias was found with P=0.296.

G The publication bias between CD147 expression and TNM staging. No publication bias was found with P=1.000.

H The publication bias between CD147 expression and tumor depth.No publication bias was found with P=0.536.

I The publication bias between CD147 expression and status of lymph node.Publication bias was found with P=0.016.

J The publication bias between CD147 expression and tumor differentiation.No publication bias was found with P=0.721.

K The publication bias between CD147 expression and age.No publication bias was found with P=1.000.

L The publication bias between CD147 expression and sex.No publication bias was found with P=0.902.

.
