# Supplementary material for: Expression of Concern: The prognostic and clinicopathologic characteristics of CD147 and esophagus cancer: A meta-analysis
Source: PLoS One. 2023 Feb 22;18(2):e0282229. doi: 10.1371/journal.pone.0282229 (PMC9946197; doi:10.1371/journal.pone.0282229)
Supplement: S1 File — (ZIP) [file pone.0282229.s001.zip › Supplementary data/Figure legend-Supplementary Sensitivity analysis plot.docx]

Sensitive analysis was performed to evaluate the influences of individual studies on the final effect size if the parameter has no less than three data set for parameter.The middle vertical line is the combined OR. The vertical lines on both sides are the [upper](http://cn.bing.com/dict/clientsentence?mkt=zh-CN&setLang=zh&form=BDVEHC&ClientVer=BDDTV3.5.0.4311&q=%E4%B8%8A%E4%B8%8B%E9%99%90) and [lower](http://cn.bing.com/dict/clientsentence?mkt=zh-CN&setLang=zh&form=BDVEHC&ClientVer=BDDTV3.5.0.4311&q=%E4%B8%8A%E4%B8%8B%E9%99%90) limits of 95% CI. The horizontal lines corresponding to each study is the final combined OR when removing itself.

A OR fluctuation between cancer and noncancer tissues was (8.55~10.73) and the 95%CI fluctuation was (4.87~19.43).

B OR fluctuation between cancer and normal tissues was (9.27~18.43) and the 95%CI fluctuation was (2.37~90.29).

C OR fluctuation between between cancer and para-carcinoma tissues was (10.82~15.24) and the 95%CI fluctuation was (5.83~30.30).

D OR fluctuation between cancer and hyperplastic tissues was (2.48~5.14) and the 95%CI fluctuation was (0.93~10.95).

E OR fluctuation between CD147 expression and TNM staging was (2.80~5.31) and the 95%CI fluctuation was (1.94~9.59).

F OR fluctuation between CD147 expression and tumor depth was (6.64~9.46) and the 95%CI fluctuation was (3.54~19.26).

G OR fluctuation between CD147 expression and status of lymph node was (4.22~6.50) and the 95%CI fluctuation was (1.74~16.32).

H OR fluctuation between CD147 expression and tumor differentiation was (1.33~2.01) and the 95%CI fluctuation was (0.67~3.55).

I OR fluctuation between CD147 expression and age was (0.95~1.08) and the 95%CI fluctuation was (0.63~1.82).

K OR fluctuation between CD147 expression and age was (0.64~0.80) and the 95%CI fluctuation was (0.41~1.18).
