# Supplementary material for: Expression of Concern: The prognostic and clinicopathologic characteristics of CD147 and esophagus cancer: A meta-analysis
Source: PLoS One. 2023 Feb 22;18(2):e0282229. doi: 10.1371/journal.pone.0282229 (PMC9946197; doi:10.1371/journal.pone.0282229)
Supplement: S1 File — (ZIP) [file pone.0282229.s001.zip › Supplementary data/Supplementary Table.1 NOSscore.docx]

Table.2 NOS score

| Column | Entries | First author | | | | | | | | | | | | | | | | |
| --- | --- | --- | --- | --- | --- | --- | --- | --- | --- | --- | --- | --- | --- | --- | --- | --- | --- | --- |
|  |  | ① | ② | ③ | ④ | ⑤ | ⑥ | ⑦ | ⑧ | ⑨ | ⑩ | ⑪ | ⑫ | ⑬ | ⑭ | ⑮ | 16 | 17 |
|  | Is the definition adequate | ☆ | ☆ | ☆ | ☆ | ☆ | ☆ | ☆ | ☆ | ☆ | ☆ | ☆ | ☆ | ☆ | ☆ | ☆ | ☆ | ☆ |
|  | Representativeness of the cases | ☆ | ☆ | ☆ | ☆ | ☆ | ☆ | ☆ | ☆ | ☆ | ☆ | ☆ | ☆ | ☆ | ☆ | ☆ | ☆ | ☆ |
| Section | Selection of controls |  |  |  |  |  |  |  |  |  | ☆ |  |  |  |  |  |  |  |
|  | Definition of controls | ☆ | ☆ | ☆ | ☆ | ☆ | ☆ | ☆ | ☆ | ☆ | ☆ | ☆ | ☆ | ☆ | ☆ | ☆ | ☆ | ☆ |
| Comparability | Comparability of cases and controls on the basis of the  design and analysis | ☆☆ | ☆ | ☆ | ☆ | ☆ | ☆ | ☆☆ | ☆☆ | ☆ | ☆ | ☆ | ☆ | ☆☆ | ☆ | ☆☆ | ☆ | ☆☆ |
|  | Ascertainment of exposure | ☆ | ☆ | ☆ | ☆ | ☆ | ☆ | ☆ | ☆ | ☆ |  | ☆ | ☆ | ☆ | ☆ | ☆ | ☆ | ☆ |
| Exposure | Same method of ascertainment  for cases and controls | ☆ | ☆ | ☆ | ☆ | ☆ | ☆ | ☆ | ☆ | ☆ | ☆ | ☆ | ☆ | ☆ | ☆ | ☆ | ☆ | ☆ |
|  |  |  |  |  |  |  |  |  |  |  |  |  |  |  |  |  |  |  |
|  | Non-Response rate | ☆ | ☆ | ☆ | ☆ | ☆ | ☆ | ☆ | ☆ | ☆ | ☆ | ☆ | ☆ | ☆ | ☆ | ☆ | ☆ | ☆ |
| Total scores |  | 8 | 7 | 7 | 7 | 7 | 7 | 8 | 8 | 7 | 7 | 7 | 7 | 8 | 7 | 8 | 7 | 8 |

Notes: ①.Yoshio Ishibashi,2004; ②. Zhao JH,2004; ③. Cheng, M. F,2006; ④. Zhang HZ,2006; ⑤. Xiong SongBai，2007; ⑥. Xie L，2008; ⑦. Qi Bo，2008; ⑧.Ma Guang，2009; ⑨.Chen JX2，009; ⑩. Liu HaiMing，2010; ⑪. Xiao XiangZhi，2011; ⑫. Zhu ShaoJun，2011; ⑬.Xiong LN，2011; ⑭.Zhu ,S，2011;⑮Wan ,Y，2012；Li ChangXiu，2013；Huang ,L，2015
