# Supplementary figures and images for: Expression of Concern: The prognostic and clinicopathologic characteristics of CD147 and esophagus cancer: A meta-analysis
Source: PLoS One. 2023 Feb 22;18(2):e0282229. doi: 10.1371/journal.pone.0282229 (PMC9946197; doi:10.1371/journal.pone.0282229)

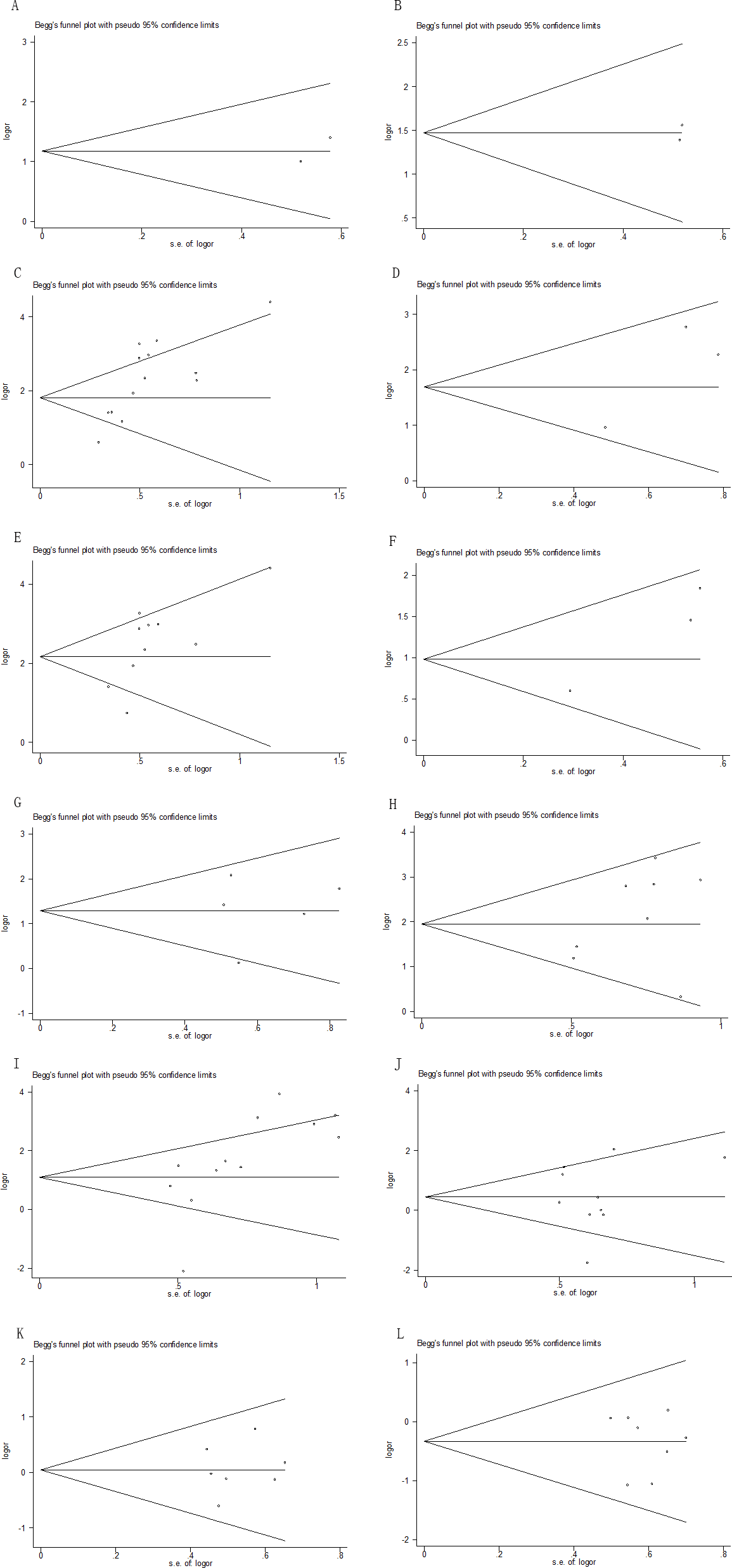

Supplement: S1 File — (ZIP) [file pone.0282229.s001.zip › Supplementary data/Supplementary Begg's Plot.tif]

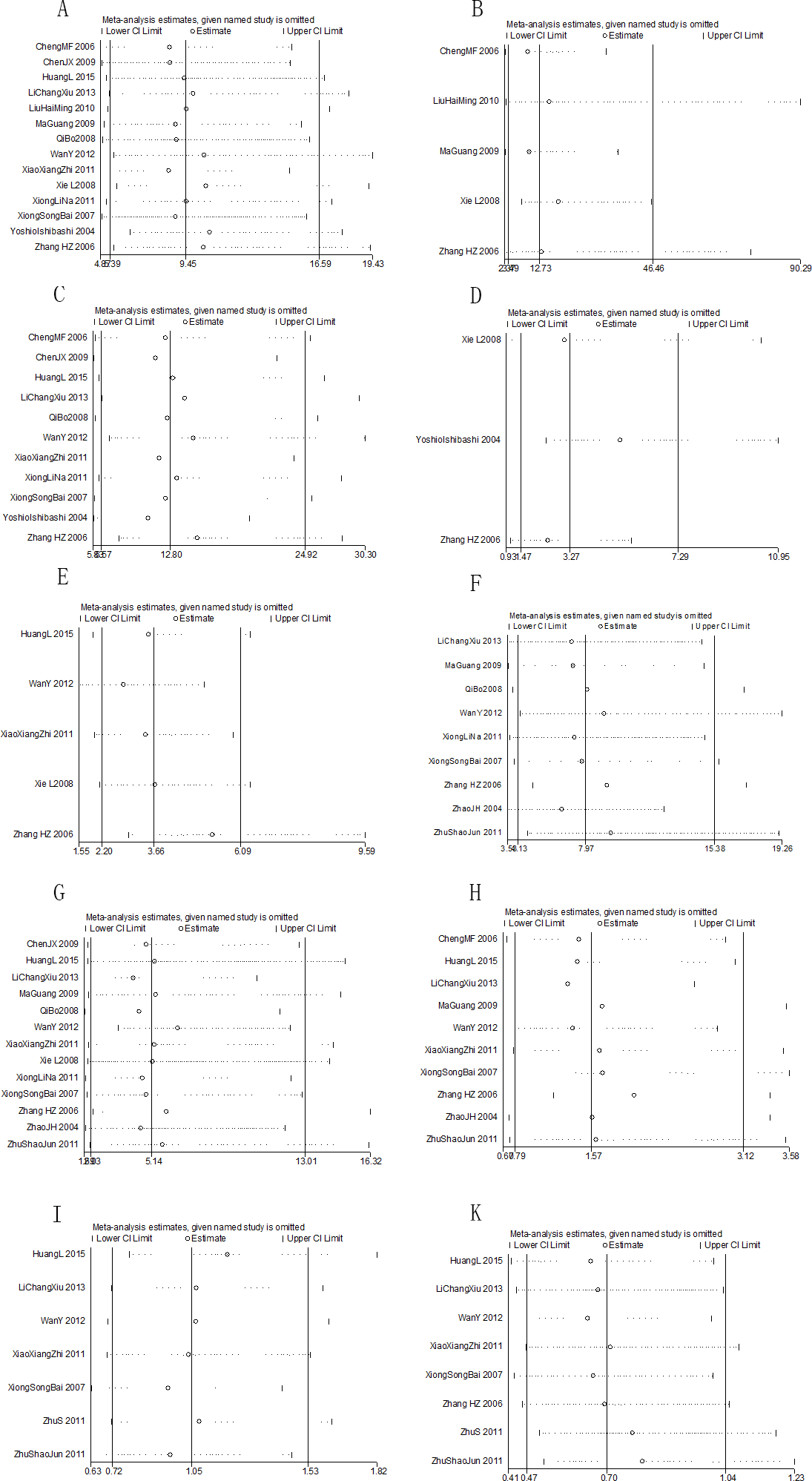

Supplement: S1 File — (ZIP) [file pone.0282229.s001.zip › Supplementary data/Supplementary Sensitivity analysis plot.tif]
